# Redirecting the Peptide Cleavage Causes Protease Inactivation

**DOI:** 10.1002/anie.202506832

**Published:** 2025-07-04

**Authors:** Christian Breuer, Jim Küppers, Anna‐Christina Schulz‐Fincke, Anna Heilos, Carina Lemke, Petra Spiwoková, Janina Schmitz, Laura Cremer, Marta Frigolé‐Vivas, Michael Lülsdorff, Matthias D. Mertens, Filip Wichterle, Miloš Apeltauer, Martin Horn, Erik Gilberg, Norbert Furtmann, Jürgen Bajorath, Ulrike Bartz, Bernd Engels, Michael Mareš, Michael Gütschow

**Affiliations:** ^1^ Pharmaceutical Institute Pharmaceutical & Medicinal Chemistry University of Bonn An der Immenburg 4 53121 Bonn Germany; ^2^ Department of Natural Sciences University of Applied Sciences Bonn‐Rhein‐Sieg von‐Liebig‐Str. 20 53359 Rheinbach Germany; ^3^ Institute of Organic Chemistry and Biochemistry Czech Academy of Sciences Flemingovo n. 2 16610 Prague Czech Republic; ^4^ Institute for Physical and Theoretical Chemistry Julius‐Maximilians‐Universität Würzburg Emil‐Fischer‐Str. 42 97074 Würzburg Germany; ^5^ Department of Life Science Informatics and Data Science B‐IT, University of Bonn Friedrich‐Hirzebruch‐Allee 6 53115 Bonn Germany

**Keywords:** Cysteine protease, Enzyme inhibition, Peptide cleavage, Suicide substrates, Transcarbamoylation

## Abstract

Cysteine and serine proteases cleave peptides through covalent catalysis by generating a transient adduct with the N‐terminal part of the substrate after releasing its C‐terminal part. We demonstrate the unique redirection of this event leading to strong enzyme inactivation. For targeting human cathepsin B, a cysteine protease of significant therapeutic importance, we designed tailored peptidomimetics with a variety of dipeptide fragments directed toward the occluding loop and equipped with numerous N‐terminal carbamate warheads. The carbamate deprotonation catalyzed by the active site thiolate initiates the redirected cleavage. The C‐terminal part of the inhibitors remains covalently attached to the protease. Hydrolysis of such carbamoyl‐enzyme complexes is catalytically unsupported rendering inhibition irreversible. This novel mechanism of action comprises a significant extension of the covalent drug space.

## Graphical abstract

Cysteine proteases catalyze substrate cleavage by a two‐step acyl transfer mechanism. Newly designed peptidic inhibitors of human cathepsin B with N‐terminal carbamate warheads undergo a redirected cleavage and generate non‐canonical covalent complexes. Representatives of this chemotype exhibit second order rate constants of inactivation greater than 10 000 M^−1^s^−1^.

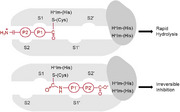

Protease substrates bind along the active site whose non‐primed (S1, S2…) or primed specificity pockets (S1′, S2′…) interact with the corresponding amino acid residues of the substrate (at positions P1, P2… or P1′, P2′…), leading to the specificity of the cleavage event. Cysteine and serine proteases catalyze the hydrolysis of peptide bonds via an acyl transfer mechanism. In the first step, and accompanied by the release of the first product, an *S*‐acyl or *O*‐acyl‐enzyme is formed as the result of the attack of the active site nucleophile at the carbonyl carbon of the scissile peptide bond. At this stage, only the non‐primed amino acid residues remain in the corresponding pockets, until the rapid, evolutionarily supported reaction with water hydrolyzes the (thio)ester bond of the protease‐substrate complex, thereby liberating the second product. A covalent protease‐substrate intermediate in which only the primed amino acid residues keep occupying the primed specificity pockets might undergo a slower hydrolysis and cause a prolonged residence time.^[^ ^1^, ^2^, ^3^ ^]^ To accomplish such a hypothetical scenario, we envisaged that a suitable peptide, addressing the primed binding region, should be equipped at its N‐terminus with an appropriate warhead. However, the typical feature of peptidic inhibitors or activity‐based probes of cysteine and serine proteases is different and comprises a C‐terminal electrophilic warhead, e.g. a phosphonate, aldehyde, sulfonylfluoride, epoxide, nitrile, acyloxymethyl ketone, or Michael acceptor.^[^ ^4^, ^5^, ^6^, ^7^, ^8^, ^9^, ^10^ ^]^ In this study, we developed peptidomimetics with an inversely oriented warhead that undergoes deprotonation by the active‐site thiolate as the key step for the covalent inactivation of the cysteine protease cathepsin B.

We considered cathepsin B as a particularly attractive protease for this approach for the following reasons. Cathepsin B shows endopeptidase and exopeptidase activity, an uncommon dual functionality among the cathepsins. In the open conformation, large endopeptidase substrates can be bound and cleaved. A special structural feature, the occluding loop, covers the primed binding region in the closed conformation of the enzyme and confers a dipeptidyl carboxypeptidase activity to cathepsin B.^[^ ^11^, ^12^, ^13^ ^]^ The two protonated histidine residues (His110 and His111) of the occluding loop interact with the C‐terminal carboxylate of exopeptidase substrates,^[^ ^11^, ^12^, ^13^ ^]^ and of epoxide‐^[^ ^14^, ^15^, ^16^, ^17^ ^]^ or nitrile‐based inhibitors.^[^ ^18^ ^]^ Hence, cathepsin B offers the opportunity for well‐defined and pronounced interactions of its primed binding region with tailored dipeptide derivatives. Targeting of the S1′ and S2′ sites of metalloproteases is known, e.g., for captopril whose N‐terminal sulfhydryl group interacts with the catalytic Zn^2+^ of angiotensin I‐converting enzyme.^[^ ^19^ ^]^ Papain‐like cysteine proteases do not exhibit a strong preference for a specific P1 amino acid,^[^ ^20^ ^]^ which allowed us to install a warhead at this position.

We designed novel cathepsin B inhibitors with P1′‐P2′ dipeptide portions and an *O*‐aryl carbamate warhead structure. To generate this library, *tert*‐butyl or methyl esters of the P2′ amino acids were coupled with Cbz‐protected P1′ amino acids. After *N*‐deprotection, the dipeptide esters were reacted with phenyl chloroformate or 4‐chlorophenyl chloroformate. The *tert*‐butyl esters of the resulting dipeptide carbamates^[^ ^21^ ^]^ were finally cleaved (Scheme S1). Solid phase chemistry on amino acid‐loaded Wang or 2‐chlorotrityl resins was carried out as well to expand this library (Schemes S2 and S3). Selected members are listed in Table 1. Besides proteinogenic amino acids, several non‐natural amino acids were incorporated, such as the β‐amino acid (*R*)‐2‐aminomethyl‐4‐methylpentanoic acid (H‐APA‐OH), obtained by an auxiliary‐supported, enantioselective route (Scheme S4), and cyclohexylglycine (H‐Chg‐OH), cyclohexylalanine (H‐Cha‐OH) and homoproline (H‐Pip‐OH). In total, 32 combinations of dipeptides were applied to establish the library of type **A** carbamates.

**Table 1:** Selected dipeptide carbamates of type **A** and cathepsin B inhibition.

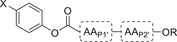						
cmpd^a)^	X	AA_P1′_	AA_P2′_	R	r.a. (%)^b)^	*k*_inac_/*K*_i_ ± SE (M^−1^s^−1^)^c)^
**1**	H	Leu	Phe	H	23	157 ± 13
**2**	Cl	Leu	Phe	*t*Bu	92	n.d.^d)^
**3**	Cl	Leu	Phe	Me	97	n.d.
**4**	Cl	Leu	Phe	H	≤2	745 ± 5
**5**	Cl	APA^e)^	Phe	H	11	108 ± 5
**6**	Cl	Chg	Phe	H	≤2	1670 ± 50
**7**	Cl	Cha	Phe	H	≤2	2010 ± 110
**8**	Cl	Leu	Pro	H	4	711 ± 40
**9**	Cl	APA	Pro	H	53	30.3 ± 4.1
**10**	Cl	Leu	Pip	H	5	933 ± 40
**11**	Cl	Chg	Pro	H	≤2	807 ± 10
**12**	Cl	Cha	Pro	H	≤2	548 ± 10
**13**	Cl	Cha	Pip	H	≤2	1240 ± 30

^a)^ H‐AA_P1′_‐OH and H‐AA_P2′_‐OH refer to the amino acids in P1′ and P2′ position.

^b)^ Residual enzymatic activity from duplicate measurements at 20 µM inhibitor concentration.

^c)^ Second‐order rate constant of enzyme inactivation.

^d)^ Not determined if the residual cathepsin B activity in the presence of 20 µM inhibitor was >70%.

^e)^ H‐APA‐OH = (*R*)‐2‐aminomethyl‐4‐methylpentanoic acid.

To get a first insight into the interaction with human cathepsin B, the inhibition of the enzyme‐catalyzed cleavage of a chromogenic peptide substrate was examined. Two methyl and ten *tert*‐butyl esters, as well as 34 carboxylic acids were initially applied at a concentration of 20 µM, and indeed, cathepsin B inhibition was observed in certain cases. Active compounds were subjected to kinetic measurements at five different concentrations (Tables 1 and S1). The progress curves revealed time‐dependent inhibition and the resulting first‐order rate constants were utilized to obtain second‐order rate constants of irreversible enzyme inhibition, *k*_inac_/*K*_i_ (Figure 1). We also investigated the inhibition of three related human cathepsins, i.e., L, K, and S, by the 46 type **A** carbamates (Table S1) and did not observe enzyme inhibition (residual activity of more than 50% at an inhibitor concentration of 20 µM or *k*_inac_/*K*_i_ values below 40 M^−1^s^−1^).

**Figure 1:**
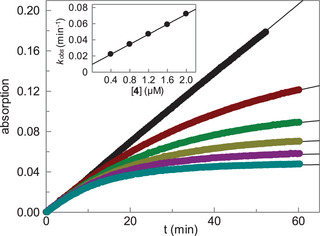
Cathepsin B‐catalyzed hydrolysis of 500 µM (= 0.45 *K*_m_) of Cbz‐Arg‐Arg‐*p*‐nitroanilide in the absence (black) or presence of increasing concentrations of carbamate **4** (from top to bottom: 0.4, 0.8, 1.2, 1.6, and 2.0 µM). Inset: A plot of first‐order rate constants *k*_obs_ (mean values of duplicate measurements) versus the inhibitor concentrations gave a *k*_inac_/*K*_i_ value of 745 ± 5 M^−1^s^−1^ using the equation *k*_obs_/[I] = (*k*_inac_/*K*_i_)/(1 + [S]/*K*_m_).

Compounds with a free carboxyl group inhibited cathepsin B to a stronger extent than analogous methyl or *tert*‐butyl esters, which were inactive in the majority of cases (e.g., **4** versus **2** and **3**, Table 1). These findings indicate an interaction of the inhibitors with the histidine residues of the occluding loop. The introduction of a chloro substituent at *para* position of the *O*‐aryl moiety improved potency (e.g., **1** versus **4**), pointing to the nucleofugality as an essential factor for enzyme inactivation. While maintaining the *para*‐chloro substitution and the C‐terminal carboxylate, the dipeptide pattern was varied in the course of this P1′‐P2′ mapping approach (Tables 1 and S1). With Pro in P2′ position, Leu in P1′ was slightly more advantageous than Cha (**8** versus **12**), but Phe in P2′ gave a better combination with Cha than with Leu in P1′ (**7** versus **4**). Such data exemplify subsite cooperativity caused by conformational plasticity within the S1′‐S2′ region.^[^ ^22^, ^23^ ^]^ An elongation of the distance between the C‐terminal carboxylate and the carbamate group by one methylene unit diminished the inhibitory potency (**4** versus **5**; **8** versus **9**). The most suitable dipeptide sequences were disclosed as Cha‐Phe, Chg‐Phe, and Cha‐Pip (**7**, **6**, and **13**).

As depicted in Table 2, we modified the carbamate part of inhibitors **4** and **8**. The nitrogen was methylated in **14** and **15**, while the carbamate motif was flipped in **16** and **17** (Scheme S5) and a urea moiety was installed in **18** and **19** (Scheme S6). In contrast to the active compounds **4** and **8**, type **B** analogs **14**–**19** were virtually inactive (Tables 2 and S2). These data strongly indicate that dipeptidic *O*‐aryl carbamates undergo an elimination reaction once they are bound in the active site of cathepsin B. This proceeds via an E1cB or E2 pathway depending on whether carbamate deprotonation and release of the phenolate leaving group occur consecutively or simultaneously. The generated peptidyl isocyanate will in turn carbamoylate the active site cysteine leading to protease inactivation (Figure 2). Hence, compounds **14**–**19** rendered inoperative, either through the inability for deprotonation (**14**, **15**) or the absence of a good leaving group (**16**–**19**). Once we had assumed the formation of a covalent thiocarbamate complex, this was detailed by covalent docking. The predicted binding mode of **8**, superimposed with the crystallographic inhibitor CA‐030, indicated a highly similar occupancy of the primed binding region with the carboxylates of both inhibitors interacting with His110 and His111 (Figure S3).

**Table 2:** Selected carbonic acid derivatives of type **B** and cathepsin B inhibition.

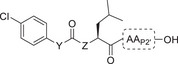					
cmpd^a)^	Y	Z	AA_P2′_	r.a. (%)^b)^	*k*_inac_/*K*_i_ ± SE (M^−1^s^−1^)^c)^
**14**	O	NMe	Phe	85	n.d.^d)^
**15**	O	NMe	Pro	≥98	n.d.
**16**	NH	O	Phe	91	n.d.
**17**	NH	O	Pro	68	10.6 ± 1.6
**18**	NH	NH	Phe	90	n.d.
**19**	NH	NH	Pro	95	n.d.

^a)^ H‐AA_P2′_‐OH refers to the amino acid in P2′ position.

^b)^ Residual enzymatic activity from duplicate measurements at an inhibitor concentration of 20 µM.

^c)^ Second‐order rate constant of enzyme inactivation.

^d)^ Not determined if the residual cathepsin B activity in the presence of 20 µM inhibitor was >70%.

**Figure 2:**
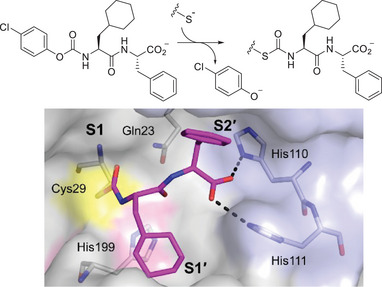
Top. Formation of an *S*‐carbamoyl enzyme through the reaction of **7** with cathepsin B. Bottom. The crystal structure of the complex of cathepsin B with **7** (magenta sticks). The enzyme is in colored surface representation with critical residues as gray sticks. The catalytic Cys29 (yellow surface) underwent carbamoylation and the inhibitor remained occupying the S1′‐S2′ binding region. His199 (pink surface) and Gln23 also participate in catalysis. Dashed lines indicate hydrogen bonds formed between the carboxylate of the inhibitor and histidine residues of the occluding loop (His110 and His111; light blue surface).

Using the Leu‐Pro dipeptide pattern, further inhibitors with modified *O*‐aryl moieties have been prepared in order to optimize the non‐primed site addressing portion (e.g., **20**–**28**, Tables 3 and S3). The key step involves a triphosgene‐promoted introduction of the CO unit (Scheme S12) to connect the dipeptide with the independently prepared (Schemes S7–S11) phenol building blocks. The subseries of type **C** carbamates comprises 21 carboxylic acids and 14 *tert*‐butyl esters, the latter being inactive against cathepsins L, K, and S (Table S3).

**Table 3:** Selected dipeptide carbamates of type **C** and cathepsin B inhibition.

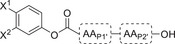						
cmpd^a)^	X^1^	X^2^	AA_P1′_	AA_P2′_	r.a. (%)^b)^	*k*_inac_/*K*_i_ ± SE (M^−1^s^−1^)^c)^
20	Cl	BnCONH	Leu	Pro	≤2	2860 ± 50
21	Ph	BnCONH	Leu	Pro	≤2	1340 ± 20
22	Cl	(Ph)_2_CHCONH	Leu	Pro	≤2	3830 ± 70
23	H	Cl	Leu	Pro	≤2	2760 ± 50
24	BnCONH	Cl	Leu	Pro	≤2	6530 ± 310
25	4‐Biphenyl‐CONH	Cl	Leu	Pro	≤2	3640 ± 60
26	(Bn)_2_NCONH	Br	Leu	Pro	≤2	2660 ± 80
27	(Ph)_2_CHCONH	Br	Leu	Pro	≤2	6000 ± 100
28	(Ph)_2_CHCONH	Cl	Leu	Pro	≤2	8180 ± 290
29	(Ph)_2_CHCONH	Cl	Leu	Phe	≤2	11300 ± 600
30	(Ph)_2_CHCONH	Cl	Cha	Phe	≤2	13800 ± 1100
31	(Bn)_2_CHCONH	Cl	Cha	Phe	≤2	23600 ± 1900

^a)^ H‐AA_P1′_‐OH and H‐AA_P2′_‐OH refer to the amino acids in P1′ and P2′ position.

^b)^ Residual enzymatic activity from duplicate measurements at an inhibitor concentration of 20 µM. Residual activity of cathepsin B at an inhibitor concentration of 20 µM.

^c)^ Second‐order rate constant of enzyme inactivation.

Introduction of a *meta*‐phenylacetamido substituent caused a pronounced inactivation (**20**, Table 3 versus **8**, Table 1). This and the following results revealed that also the structural expansion of the warhead can increase the *k*_inac_/*K*_i_ value. An exchange of the *meta* and *para* substituents demonstrated the benefit of a halo substituent in *meta* and a bulky hydrophobic group in *para* position (**20** versus **24** and **22** versus **28**). Whereas *meta*‐halo substitution clearly had an electronic effect, the hydrophobic groups did not alter the chemical reactivity of the warhead but favored the association of the non‐covalent enzyme‐inhibitor complex (**23** versus **24** and **28**). Branched residues were particularly advantageous (**24** and **25** versus **28**).

We combined the most effective fragments, i.e., the dipeptide pattern of **7** (Table 1) and the warhead part of **28** (Table 3), assembled the structure of carbamate **30** and reached a second‐order rate constant of inactivation of more than 10 000 M^−1^s^−1^. Molecular docking of the non‐covalent cathepsin B‐**30** complex suggested available space for the insertion of two methylene linkers to direct the aromatic rings deeper into the S2 and S3 pockets (Figures S4 and S5). Hence, we conceived the structure of carbamate **31**. Its convergent synthesis (Scheme 1) started with an amide coupling using an aniline whose phenolic group was protected to ensure a regioselective acylation. After deprotection, the phenolic building block was treated with triphosgene generating an in situ chloroformate. EDC‐promoted peptide coupling of the P1′ amino acid with the *tert*‐butyl ester of the P2′ amino acid, followed by hydrogenolytic *N*‐deprotection afforded the amino component for the subsequent carbamate formation. A final *tert*‐butyl ester cleavage gave **31**.

**Scheme 1:**
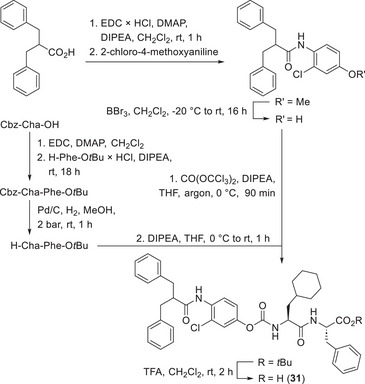
Synthesis of the dipeptidyl carbamate **31**.

Compound **31** is a selective (Table S3), irreversible (Figure S2) and highly potent (*k*_inac_/*K*_i_ = 23 600 M^−1^s^−1^) inactivator of human cathepsin B. We dissected the kinetic behavior in comparison with **7**, which shares the P1′‐P2′ portion with **31**. The optimized inhibitor **31** exhibited a more than 20‐fold lower *K*_i_ value (441 nM), a clear reflection of the improved affinity to form the non‐covalent enzyme‐inhibitor complex (Table S4 and Figure S1).

To further rationalize the mode of inhibitor‐protease interaction for this chemotype, comprehensive molecular dynamics (MD) simulations and quantum mechanics/molecular mechanics (QM/MM) calculations were performed (Figures S7–S12). They revealed the mechanistic pathway with the cathepsin‐catalyzed deprotonation of the carbamate moiety by the active site thiolate^[^ ^24^ ^]^ as the key event leading to inactivation and the crucial involvement of a water molecule in the deprotonation step.

Finally, crystallographic analysis was pursued to corroborate the transcarbamoylation reaction and fine‐tune the binding mode of our inhibitors. Two representatives were subjected to crystallization and the crystal structures of human cathepsin B in complex with the type **A** carbamate **7** (Table 1) and the optimized inhibitor **31** (Table 3) were determined at a resolution of 1.45 Å (Figures 2 and S13) and 1.70 Å (Figure S13), respectively. Atomic coordinates and experimental structure factors have been deposited in the Protein Data Bank with accession codes 8B4T and 8B5F, respectively. Both structures confirm the formation of the thiocarbamate linkage. Noteworthy, since **7** and **31** contain the same carbamoylating substructure, the complexes are highly similar with an RMSD of 0.12 and 0.09 Å for alignment of inhibitor atoms and protein Cα atoms, respectively. Hence, the different N‐terminal substructures influenced the non‐covalent binding event, but not the accommodation of the covalently attached dipeptide portion. The latter is held in place through a network of hydrogen bonds and hydrophobic interactions (Figure S13) mainly with the side chains of Val176, Leu181, Met196, His199, Trp221, and His110, His111, which compose the S1′ and S2′ subsites, respectively (Table S5 and Figure S13). The N‐terminal CO‐NH segment is hydrogen bonded to all three residues involved in catalysis, including Cys29 and Gln23 (in S1) and His199 (in S1′). The imidazole nitrogens of His110 and His111 interact with the properly positioned carboxylate (Figure 2), similarly as in case of CA‐030 (Figure S14).

Cathepsin B has been validated as an effective cancer and inflammatory biomarker and a promising drug target.^[^ ^25^, ^26^, ^27^ ^]^ Low‐molecular weight inhibitors represent valuable theranostic tool compounds^[^ ^28^, ^29^, ^30^, ^31^ ^]^ as well as potential anti‐tumor^[^ ^32^, ^33^, ^34^ ^]^ and anti‐SARS‐CoV‐2 therapeutics.^[^ ^35^, ^36^ ^]^ Our study led to the discovery of the development compound **31**, an outstandingly potent cathepsin B inactivator.

By deciphering the intriguing mechanism of interaction of this chemotype of inhibitors, a crucial polar interaction with the occluding loop was demonstrated. Strong evidence was provided that the active site cysteine thiolate acts as a base and triggers a unique enzyme‐catalyzed carbamate deprotonation event. Our inhibitors with inversely oriented warheads behave as suicide substrates, undergo cleavage and effectuate the fixation of their dipeptide portion in the primed binding region of the protease. Such a scenario has not been achieved so far and is opposite when compared to the canonical cysteine protease‐catalyzed peptide cleavage.

## Conflict of Interests

The authors declare no conflict of interest.

## Supporting information



Supporting Information

## Data Availability

The data that support the findings of this study are available in the supplementary material of this article.
