# Supplementary material for: Redirecting the Peptide Cleavage Causes Protease Inactivation
Source: Angew Chem Int Ed Engl. 2025 Jul 4;64(31):e202506832. doi: 10.1002/anie.202506832 (PMC12304826; doi:10.1002/anie.202506832)
Supplement: Supplementary file 1 — Supporting Information [file ANIE-64-e202506832-s001.pdf]

Supporting Information

Redirecting the Peptide Cleavage Causes Protease Inactivation

Christian Breuer,[a,b]◊ Jim Küppers,[a]◊ Anna-Christina Schulz-Fincke,[a] Anna Heilos,[d] Carina Lemke,[a] Petra Spiwoková,[c] Janina Schmitz,[a,b] Laura Cremer,[a] Marta Frigolé-Vivas,[a] Michael Lülsdorff,[a] Matthias D. Mertens,[a] Filip Wichterle,[c] Miloš Apeltauer,[c] Martin Horn,[c] Erik Gilberg,[a,e] Norbert Furtmann,[a,e] Jürgen Bajorath,[e] Ulrike Bartz,[b] Bernd Engels,[d] Michael Mareš,[c] and Michael Gütschow[a]*

[a] Pharmaceutical Institute, Pharmaceutical & Medicinal Chemistry, University of Bonn, An der Immenburg 4, 53121 Bonn (Germany)

[b] Department of Natural Sciences, University of Applied Sciences Bonn-Rhein-Sieg, von-Liebig-Str. 20, 53359 Rheinbach (Germany)

[c] Institute of Organic Chemistry and Biochemistry, Czech Academy of Sciences, Flemingovo n. 2, 16610 Prague (Czech Republic)

[d] Institute for Physical and Theoretical Chemistry, Julius-Maximilians-Universität Würzburg, Emil-Fischer-Str. 42, 97074 Würzburg (Germany)

[e] Department of Life Science Informatics and Data Science, B-IT, University of Bonn, Friedrich-Hirzebruch-Allee 6, 53115 Bonn (Germany)

[◊] These authors contributed equally to this work.

Table of Content

1. Enzyme Inhibition Assays and Kinetic Data of Inhibition of Cathepsins B, L, K and S... S4

Table S1. Inhibitory properties of type **A** carbamates………………………………………. S6

Table S2. Inhibitory properties of type **B** carbamates and ureas…………………………. S12

Table S3. Inhibitory properties of type **C** carbamates………………………………………. S14

Table S4. Kinetic data of cathepsin B inhibition by two selected carbamates (**7** and **31**)... S19

Figure S1. Inhibition of human cathepsin B by carbamate inhibitor **31**………………….. S20

Figure S2. Cathepsin B reactivation experiment with carbamate inhibitors **7** and **31**……. S21

2. Synthetic Schemes………………………………………………………………………. S22

Scheme S1. Solution-phase synthesis of *O*-aryl-*N*-dipeptidyl-carbamates of type **A**…….. S22

Scheme S2. Solid-phase synthesis of type **A** carbamates on a Wang resin………………. S23

Scheme S3. Solid-phase synthesis of type **A** carbamates on a 2-chlorotrityl resin……….. S23

Scheme S4. Synthesis of Fmoc-APA-OH………………………………………………… S24

Scheme S5. Synthesis of *N*-aryl-carbamates of type **B**…………………………………… S24

Scheme S6. Synthesis of *N*-aryl-ureas of type **B**………………………………………….. S25

Scheme S7. Synthesis of *N*-substituted chlorophenols…………………………………….. S25

Scheme S8. Synthesis of a biaryl-phenol………………………..…………………….. S25

Scheme S9. Synthesis of *N*-substituted bromophenols…………………………………… S26

Scheme S10. Synthesis of a 4-urea-phenol………………………………………………… S26

Scheme S11. Synthesis of a 2-benzyl-3-phenylpropanamido-substituted phenol…………. S26

Scheme S12. Synthesis of *O*-aryl-*N*-dipeptidyl-carbamates of type **C**…………………….. S27

3. Molecular Modeling……………………………………………………………………. S28

Figure S3. Covalent docking of carbamate **8** bound to the active site of human cathepsin B. Crystallographic binding mode of CA-030………………………………... S29

Figure S4. Docking of carbamate **30** bound to the active site of human cathepsin B……… S30

Figure S5. Docking of carbamate **31** bound to the active site of human cathepsin B………. S31

Figure S6. Docking of carbamate **4** bound to the active site of human cathepsin B. Covalent docking of the inhibitor after carbamoylation of the active site cysteine……….. S32

4. QM/MM Calculations…………………………………………………………..…… S33

Figure S7. Reaction scheme of the NEB calculations with a water-mediated proton transfer………………………………………………………………………..…… S36

Figure S8. Combined NEB profiles……………………………………………………….. S36

Figure S9. Reaction scheme of the carbamoylation of Cys29…………………………... S37

Figure S10. Product structure within a relaxed environment, including a water molecule. S37

Figure S11. Converged structures with a water molecule in the active site……………… S37

Figure S12. QM/MM NEB path of the carbamoylation of Cys29 through the isocyanate intermediate…………………………………………………………………………... S38

Table of Content (continuation)

5. Crystallographic Analysis……………………………………………….…….……………… S39

Figure S13. Crystal structures of the carbamate inhibitors **7** and **31** in complex with human cathepsin B and binding mode analysis…………………………………………….………. S42

Figure S14. Comparison of crystallographic binding modes of carbamate inhibitors and the inhibitor CA-030……………………………………………………………………………. S43

Table S5. Crystallographic contacts formed between human cathepsin B and inhibitors **7** and **31**………………………………………………………………………………………… S44

Table S6. X-ray data collection and refinement statistics……….……………………….. S45

6. General Methods and Synthetic Procedures……….………..………………………… S46

7. Preparation of Compounds……………………………………..……………………….. S51

7.1. Dipeptide and amino acid precursors………………………………………………. S51

7.2. Dipeptide carbamates of type A……………………………………………………... S63

7.3. Carbamates and ureas of type B…………………………………………………… S89

7.4. Phenoles and anisol precursors…………………………………………………….. S96

7.5. Dipeptide carbamates of type C……………………………………………………. S105

8. NMR Spectra……………………………………………………………….………………….. S132

9. References………………………………………………………………………………… S163

1. Enzyme Inhibition Assays and Kinetic Data of Inhibition of Cathepsins B, L, K and S

General. Human cathepsins were assayed either fluorimetrically or spectrophotometrically as noted below. The 10 mM inhibitor solutions were prepared in DMSO. The final concentration of DMSO in all assays was 2%. Non-linear regressions were conducted with GraFit 5. Residual cathepsin activity at an inhibitor concentration of 20 µM was determined from duplicate measurements based on the product formation after 60 min. Concentration dependent measurements were performed in duplicate measurements with five different inhibitor concentrations when the residual cathepsin activity in the presence of 20 µM inhibitor was below 70% (cathepsin B) or below 50% (cathepsins cathepsins L, K, and S). Progress curves were monitored for 60 min. First-order rate constants, k_{obs}, were determined by non-linear regression following the equation $[P] = v_i \times (1 - \exp(-k_{obs} \times t))/k_{obs} + d$, where [P] is the product concentration, v_i is the initial product formation rate, k_{obs} is the observed first-order rate constant, and d is the offset. Mean values of k_{obs} were plotted *versus* corresponding inhibitor concentrations [I], and second-order rate constants of inactivation, k_{inac}/K_i, were obtained by linear regression. The equation $k_{obs}/[I] = (k_{inac}/K_i)/(1 + [S]/K_m)$ was used, where [S] is the substrate concentration and K_m is the Michaelis-Menten constant. Standard errors (SE) refer to the linear regression.

Cathepsin B inhibition assay.[1] Human isolated cathepsin B (Calbiochem, Darmstadt, Germany) was assayed spectrophotometrically on a Cary 50 or 100 Bio, Varian, at 405 nm. The reactions were followed at 37 °C. Assay buffer was 100 mM sodium phosphate buffer pH 6.0, 100 mM NaCl, 5 mM EDTA, 0.01% Brij 35. A stock solution of human cathepsin B of 1.81 mg/mL or 0.47 mg/mL in 20 mM sodium acetate buffer pH 5.0, 1 mM ethylenediaminetetraactic acid (EDTA) was diluted 1:500 with assay buffer containing 5 mM dithiothreitol (DTT) and incubated for 30 min at 37 ºC. A 100 mM stock solution of the *para*-nitroaniline (pNA)-based chromogenic substrate Cbz-Arg-Arg-pNA was prepared in DMSO. The final concentration of the substrate was 500 µM (= 0.45 K_m). Into a cuvette containing 960 µL assay buffer, inhibitor solution and DMSO in a total volume of 15 µL, and 5 µL of the substrate solution were added and mixed. The reaction was initiated by adding 20 µL of the enzyme solution.

Cathepsin L inhibition assay.[1] Human isolated cathepsin L (Enzo Life Sciences, Lörrach, Germany) was assayed spectrophotometrically on a Cary 50 or 100 Bio, Varian, at 405 nm. The reactions were followed at 37 °C. Assay buffer was 100 mM sodium phosphate buffer pH 6.0, 100 mM NaCl, 5 mM EDTA, and 0.01% Brij 35. A stock solution of human cathepsin L of 135 µg/mL in 20 mM malonate buffer pH 5.5, 400 mM NaCl, and 1 mM EDTA was diluted 1:100 with assay buffer containing 5 mM DTT and incubated for 30 min at 37 ºC. A 10 mM stock solution of the chromogenic substrate Cbz-Phe-Arg-pNA was prepared in DMSO. The final concentration of the substrate was 100 µM (= 5.88 K_m). Into a cuvette containing 940 µL assay buffer, inhibitor solution and DMSO in a total volume of 10 µL, and 10 µL of the substrate solution were added and mixed. The reaction was initiated by adding 40 µL of the cathepsin L solution.

Cathepsin K inhibition assay.[1] Human recombinant cathepsin K (Enzo Life Sciences, Lörrach, Germany) was assayed fluorimetrically on a Monaco Safas spectrofluorometer flx or a Fluostar OPTIMA plate reader (BMG Labtech, Ortenberg, Germany). The wavelength for excitation was 360 nm and for emission 440 nm. The reactions were followed at 25 °C. A human cathepsin K stock solution of 23 µg/mL in 50 mM sodium acetate pH 5.5, 50 mM NaCl, 0.5 mM EDTA, 5 mM DTT was diluted 1:100 with assay buffer (100 mM sodium citrate pH 5.0, 100 mM NaCl, 1 mM EDTA, 0.01% CHAPS) containing 5 mM DTT and incubated for 30 min at 37 ºC. A 10 mM stock solution of the 7-amino-4-methyl-coumarin (AMC)-based fluorogenic substrate Cbz-Leu-Arg-AMC was prepared in DMSO. The final concentration of the substrate was 40 µM (= 13.3 K_m). When using the spectrofluorometer, into a cuvette containing 960 µL assay buffer, inhibitor solution and DMSO in a total volume of 16 µL, and 4 µL of the substrate solution were added and mixed. The reaction was initiated by adding 20 µL of the cathepsin K solution. When using the plate reader, into a well containing 192 µL assay buffer, inhibitor solution and DMSO in a total volume of 3.2 µL and 0.8 µL of the substrate solution were added and mixed. The reaction was initiated by adding 4 µL of the cathepsin K solution.

Cathepsin S inhibition assay.[2] Human recombinant cathepsin S (Enzo Life Sciences, Lörrach, Germany) was assayed fluorimetrically on a Monaco Safas spectrofluorometer flx or a Fluostar OPTIMA plate reader (BMG Labtech, Ortenberg, Germany). The wavelength for excitation was 360 nm and for emission 440 nm. The reactions were followed at 25 °C. Assay buffer was 100 mM sodium phosphate buffer pH 6.0, 100 mM NaCl, 5 mM EDTA, and 0.01% Brij 35. A stock solution of human cathepsin S of 70 µg/mL in 100 mM MES buffer, pH 6.5, 1 mM EDTA, 50 mM L-cysteine, 10 mM DTT, 0.5% Triton X-100 and 30% glycerol was diluted 1:100 with a 50 mM sodium phosphate buffer pH 6.5, 50 mM NaCl, 2 mM EDTA, 0.01% Triton X-100 and 5 mM DTT and incubated for 60 min at 37 °C. A 10 mM stock solution of the fluorogenic substrate Cbz-Phe-Arg-AMC was prepared in DMSO. The assay was performed with a final substrate concentration of 40 µM (= 0.74 K_m). When using the spectrofluorometer, into a cuvette containing 920 µL assay buffer, inhibitor solution and DMSO in a total volume of 16 µL, and 4 µL of the substrate solution were added and mixed. The reaction was initiated by adding 60 µL of the cathepsin S solution. When using the plate reader, into a well containing 188 µL assay buffer, inhibitor solution and DMSO in a total volume of 3.2 µL and 0.8 µL of the substrate solution were added and mixed. The reaction was initiated by adding 12 µL of the cathepsin S solution.

Table S1. Inhibitory properties of type **A** carbamates.

Cmpd		Residual activity @ 20 µM (%)[a] or k_{inac}/K_i ± SE ($M^{-1}s^{-1}$)[b]			
		Cat B[c]	Cat L[d]	Cat K[d]	Cat S[d]
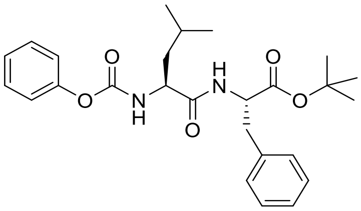		93	95	96	≥ 98
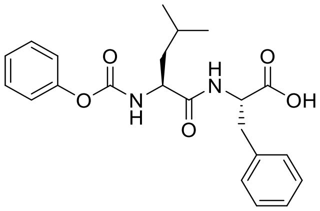	**1**	157 ± 13	94	77	97
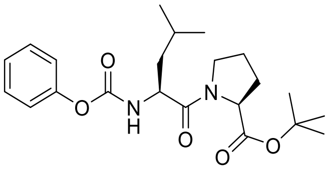		78	95	91	≥ 98
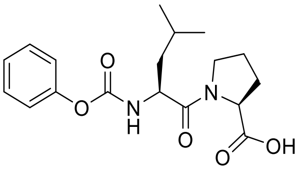		131 ± 12	94	95	≥ 98
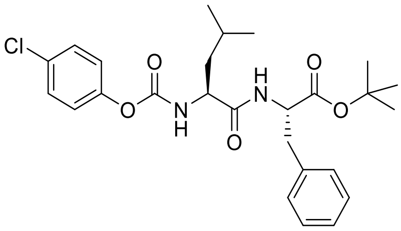	**2**	92	96	93	≥ 98
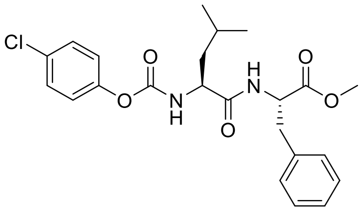	**3**	97	85	≥ 98	79
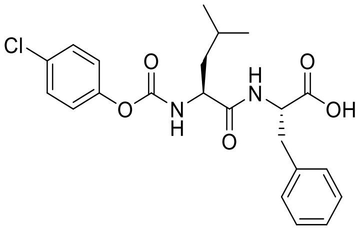	**4**	745 ± 5	92	92	83

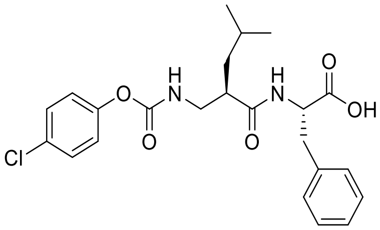	**5**	108 ± 5	90	70	10.2 ± 1.7
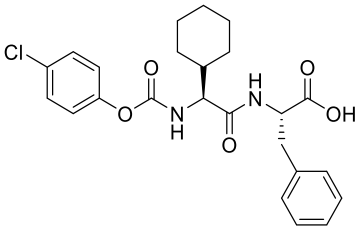	**6**	1670 ± 50	90	71	≥ 98
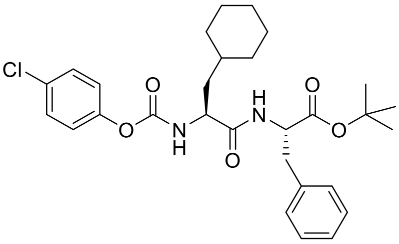		96	93	54	64
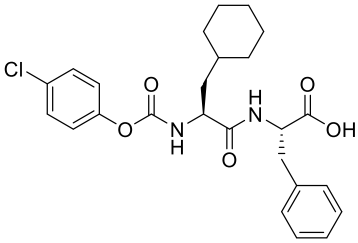	**7**	2010 ± 110	86	60	72
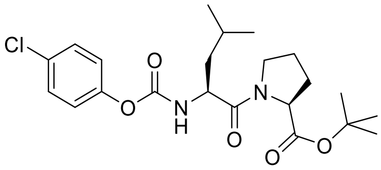		112 ± 4	83	93	≥ 98
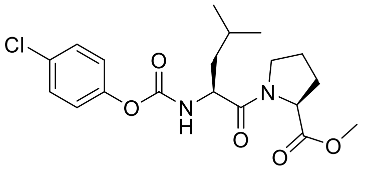		12.8 ± 0.4	84	94	92
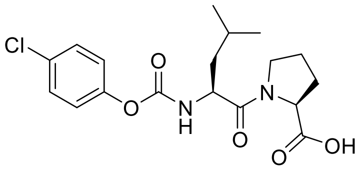	**8**	711 ± 40	97	91	≥ 98
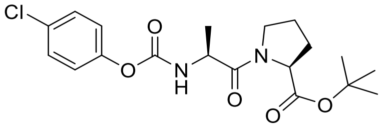		17.1 ± 1.5	96	≥ 98	≥ 98
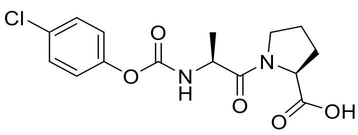		21.9 ± 0.7	≥ 98	97	93

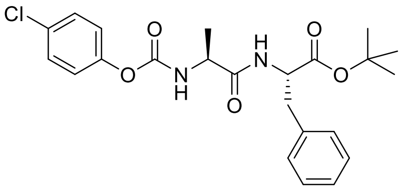	≥ 98	≥ 98	93	74
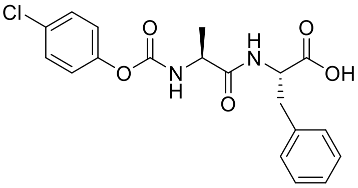	16.5 ± 0.6	≥ 98	95	82
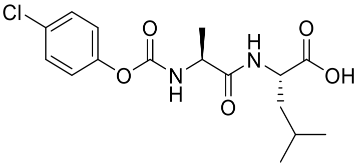	13.3 ± 0.4	95	95	75
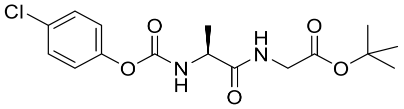	≥ 98	97	93	≥ 98
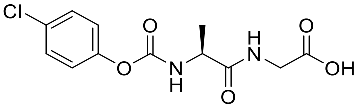	95	94	95	≥ 98
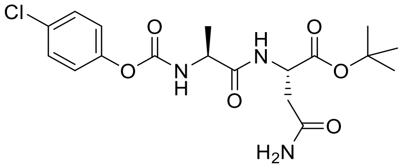	≥ 98	97	90	≥ 98
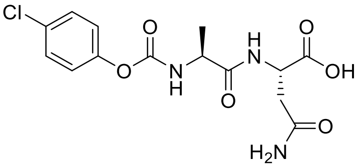	≥ 98	96	≥ 98	≥ 98
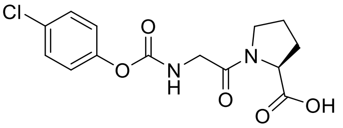	92	97	≥ 98	97
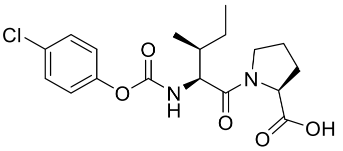	307 ± 19	≥ 98	91	≥ 98
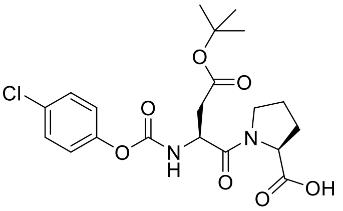	63.8 ± 2.9	≥ 98	91	97

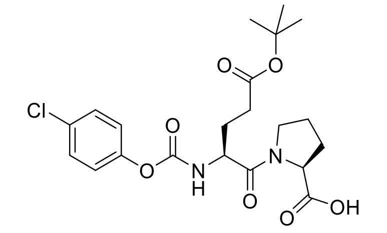		100 ± 1	≥ 98	81	97
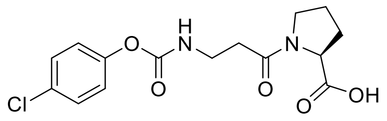		83	≥ 98	≥ 98	≥ 98
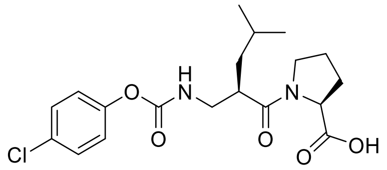	**9**	30.3 ± 4.1	95	85	92
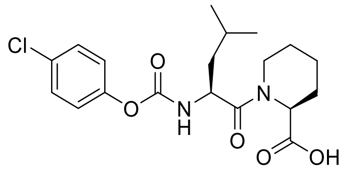	**10**	933 ± 40	91	93	≥ 98
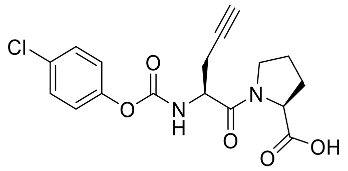		33.4 ± 0.6	97	≥ 98	≥ 98
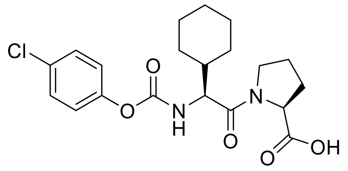	**11**	807 ± 10	91	84	88
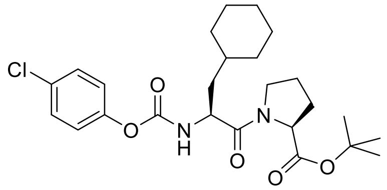		39.6 ± 2.2	87	38.6 ± 2.2	69
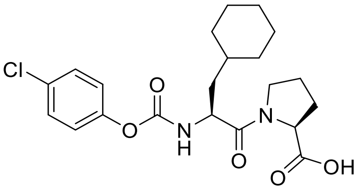	**12**	548 ± 10	97	88	83
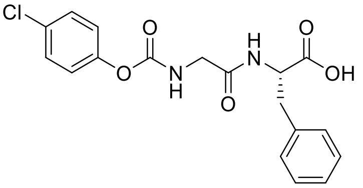		93	90	≥ 98	68
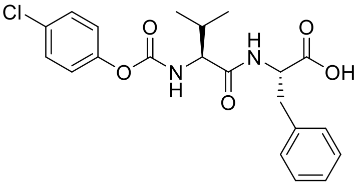		170 ± 30	97	96	≥ 98

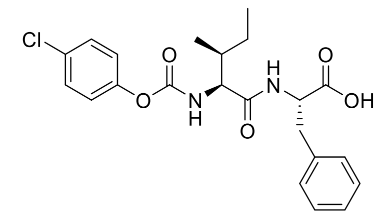	274 ± 3	97	72	≥ 98
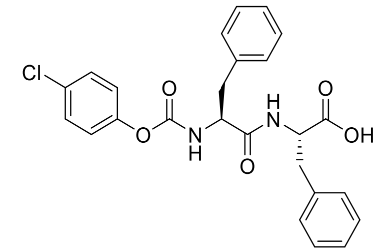	174 ± 26	91	84	6.96 ± 1.04
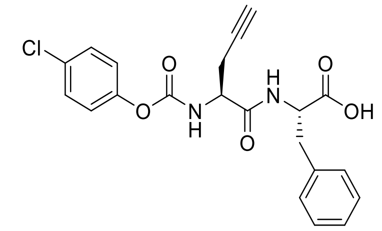	23.2 ± 0.4	96	97	82
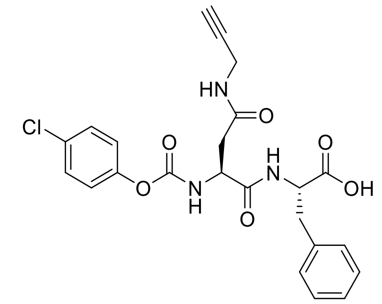	12.8 ± 1.6	≥ 98	93	92
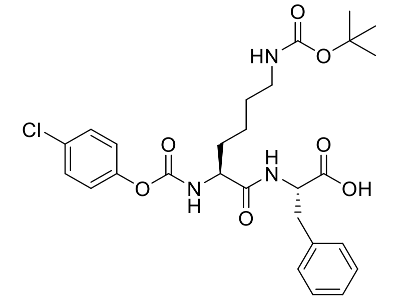	95.7 ± 2.9	95	97	90
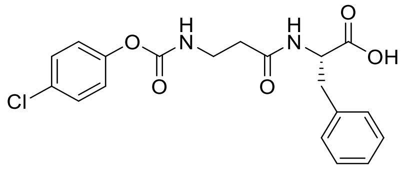	76	97	≥ 98	55
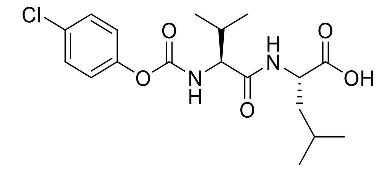	76.9 ± 4.4	97	≥ 98	84
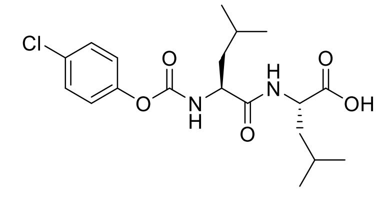	131 ± 19	95	85	93

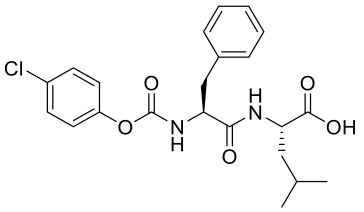		136 ± 9	96	89	88
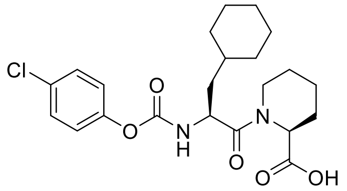	**13**	1240 ± 30	90	95	96

[a] Values without standard errors refer to the percentage residual cathepsin activity from duplicate measurements at an inhibitor concentration of 20 µM. The reactions were followed for 60 min. [b] Values with standard errors refer to the second-order rate constant of enzyme inactivation from duplicate measurements with five different inhibitor concentrations. Progress curves were monitored for 60 min. For details, see chapter Enzyme Inhibition Assays. [c] Concentration dependent measurements were not performed when the residual activity of cathepsin B in the presence of 20 µM inhibitor was above 70%. [d] Concentration dependent measurements were not performed when the residual activity of cathepsins L, K, and S in the presence of 20 µM inhibitor were above 50%.

Table S2. Inhibitory properties of type **B** carbamates and ureas.

Cmpd		Residual activity @ 20 µM (%)[a] or k_{inac}/K_i ± SE ($M^{-1}s^{-1}$)[b]			
		Cat B[c]	Cat L[d]	Cat K[d]	Cat S[d]
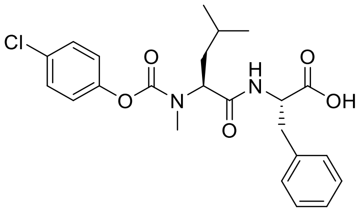	**14**	85	91[e]	76	68[e]
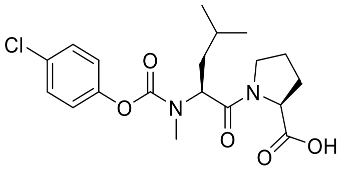	**15**	≥ 98	97	91	≥ 98
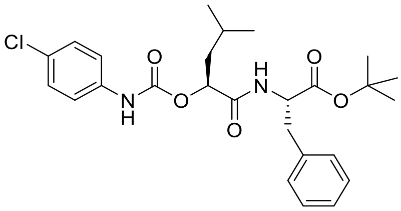		71[e]	69[e]	388 ± 54	88[e]
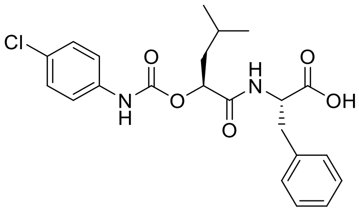	**16**	91	95	185 ± 25	56
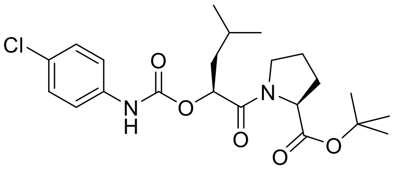		96	99	86	99
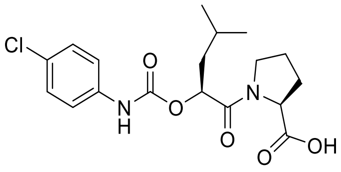	**17**	10.6 ± 1.6	94	95	93
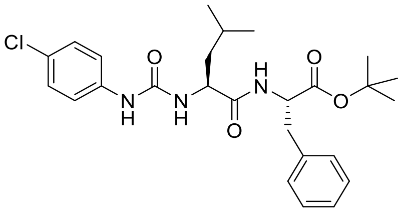		85	76	296 ± 43	94

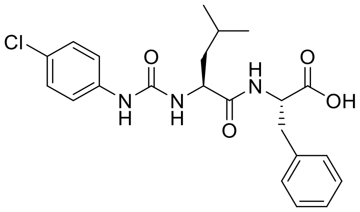	**18**	90	94	87	78
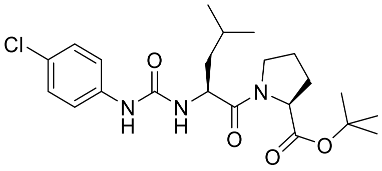		93	94	83	≥ 98
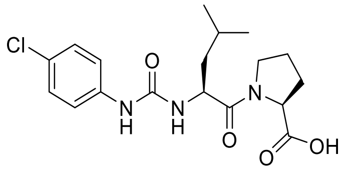	**19**	95	96	92	≥ 98

[a] Values without standard errors refer to the percentage residual cathepsin activity from duplicate measurements at an inhibitor concentration of 20 µM. The reactions were followed for 60 min. [b] Values with standard errors refer to the second-order rate constant of enzyme inactivation from duplicate measurements with five different inhibitor concentrations. Progress curves were monitored for 60 min. For details, see chapter Enzyme Inhibition Assays. [c] Concentration dependent measurements were not performed when the residual activity of cathepsin B in the presence of 20 µM inhibitor was above 70%. [d] Concentration dependent measurements were not performed when the residual activity of cathepsins L, K, and S in the presence of 20 µM inhibitor were above 50%. [e] The inhibitor was not soluble at an assay concentration of 20 µM. The IC_{50} values from duplicate measurements with two different, lower inhibitor concentrations were determined according to $IC_{50} = [I]/((P_0/P_1)-1)$, where [I] is the inhibitor concentration, P_0 is the product formation of the control (100%), and P_1 is the product formation in the presence of the inhibitor. The equation $P_1 = P_0/((20\ \mu M/IC_{50})+1)$ was then used to calculate the approximated percentage product formations at [I] = 20 µM and the mean value was listed in the Table.

Table S3. Inhibitory properties of type **C** carbamates.

Cmpd	Residual activity @ 20 μM (%)[a] or $k_{inac}/K_i \pm$ SE ($M^{-1}s^{-1}$)[b]			
	Cat B[c]	Cat L[d]	Cat K[d]	Cat S[d]
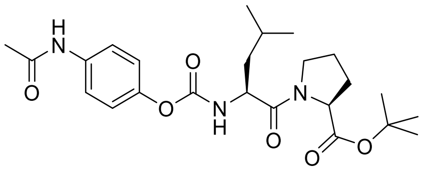	9.44 ± 0.07	88	≥ 98	≥ 98
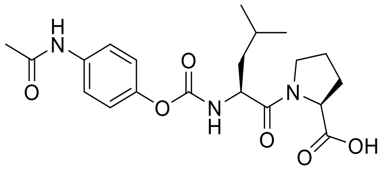	78.9 ± 3.0	88	92	≥ 98
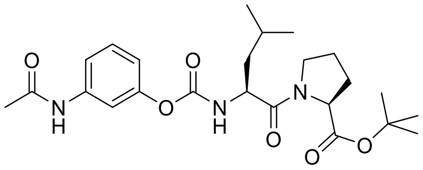	90	97	≥ 98	87
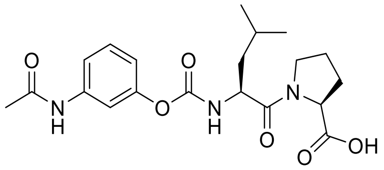	375 ± 25	92	≥ 98	≥ 98
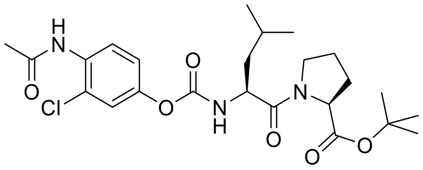	243 ± 22	61.9 ± 3.8	≥ 98	89
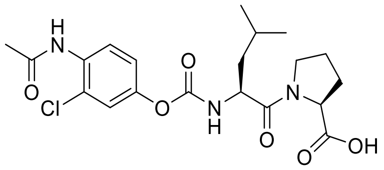	2680 ± 160	94	≥ 98	≥ 98
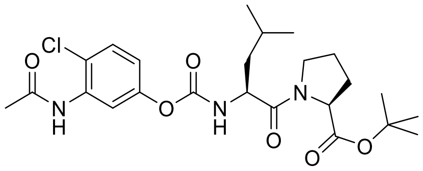	108 ± 9	76	76	57
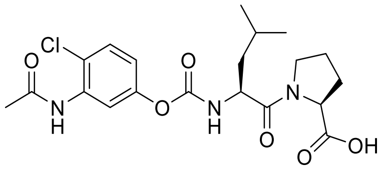	1440 ± 80	97	≥ 98	≥ 98

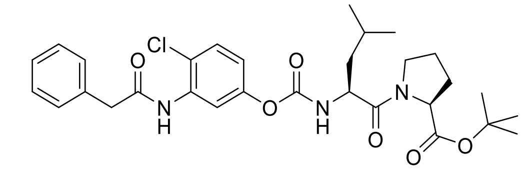		4.38 ± 0.16	96	60	91
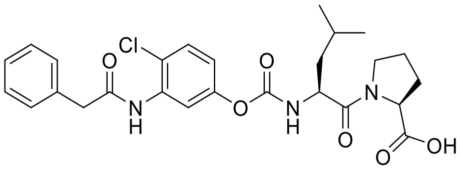	**20**	2860 ± 50	96	82	93
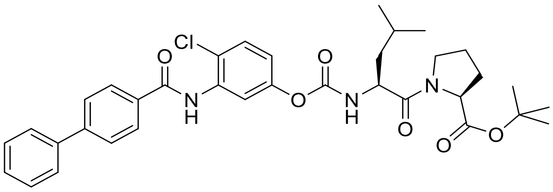		518 ± 51	83[e]	59[e]	89
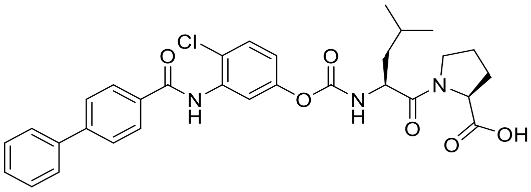		3440 ± 130	69[e]	79	66[e]
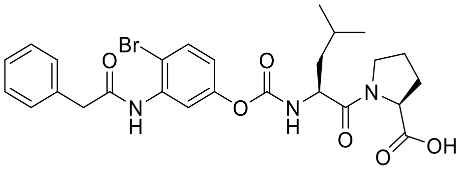		4240 ± 50	91	61	74
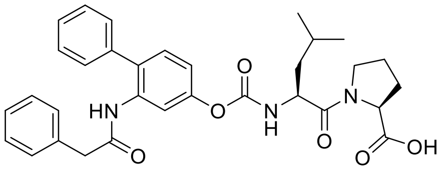	**21**	1340 ± 10	92	73	89
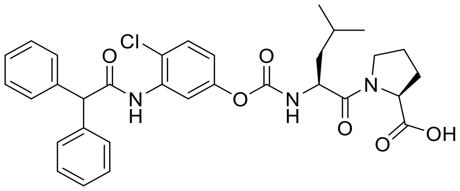	**22**	3830 ± 70	59[e]	87	87
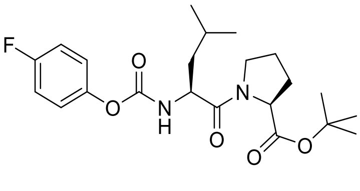		92	97	≥ 98	93
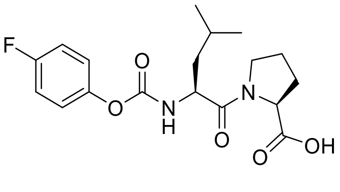		26.5 ± 1.7	≥ 98	≥ 98	≥ 98

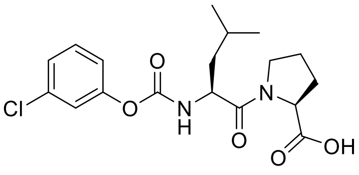	**23**	2760 ± 50	92	87	89
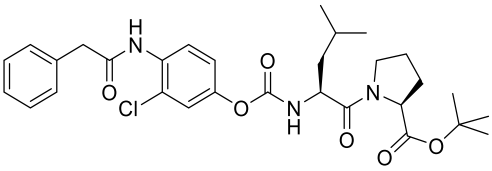		44.7 ± 1.4	88	83	95
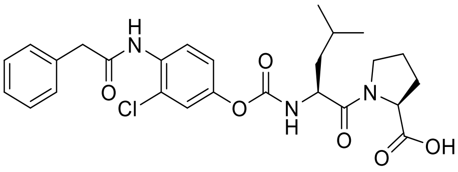	**24**	6530 ± 310	97	95	90
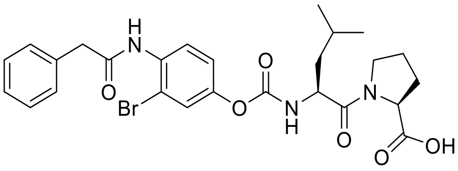		1900 ± 10	90	82	77
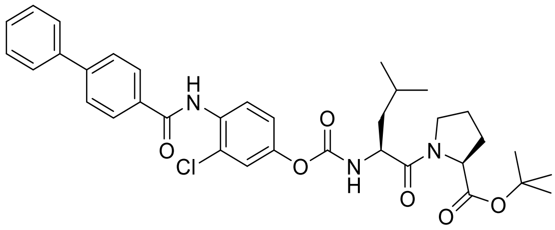		618 ± 37	90[e]	117 ± 36	62[e]
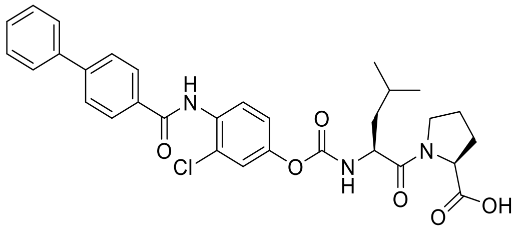	**25**	3640 ± 60	94	76[e]	53[e]
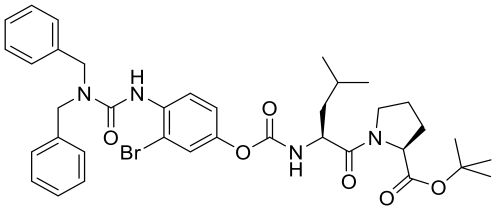		118 ± 31	71[e]	54[e]	62
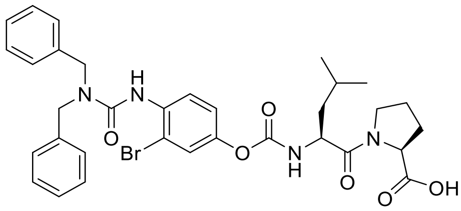	**26**	2660 ± 80	70[e]	83	59

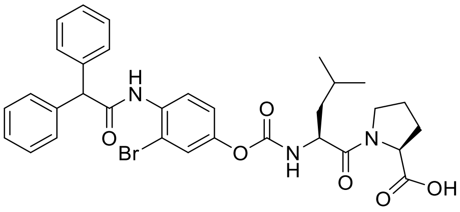	**27**	6000 ± 100	81	90	60
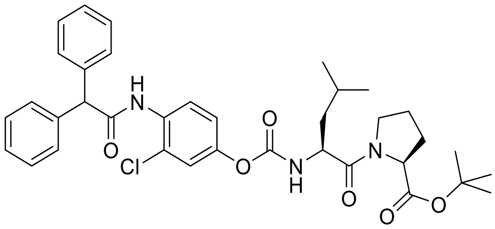		592 ± 23	92[e]	24.4 ± 7.2	74[e]
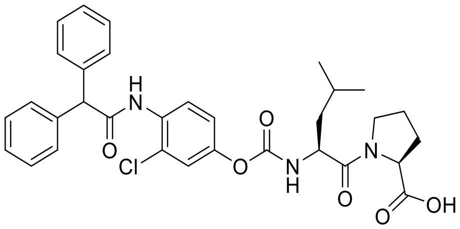	**28**	8180 ± 290	62[e]	83	58
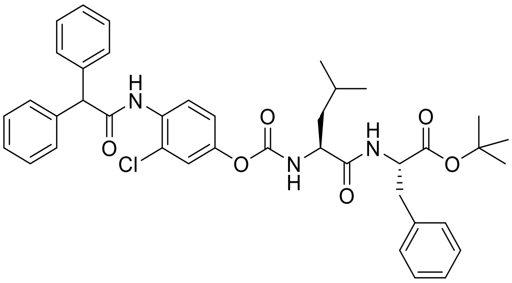		13.7 ± 80	68[e]	48[e]	62[e]
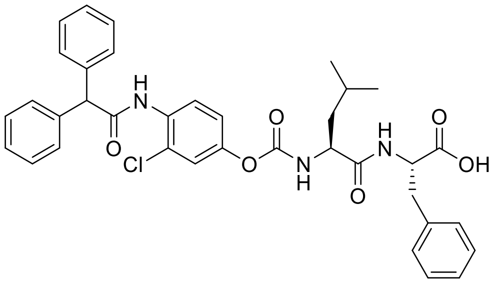	**29**	11,300 ± 600	70[e]	8[e,f]	63[e]
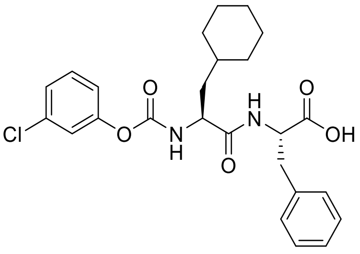		4700 ± 200	81[e]	455 ± 34	70.8 ± 13.5
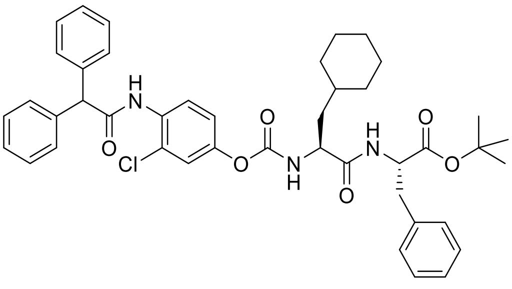		184 ± 2	54[e]	61[e]	45[e,f]

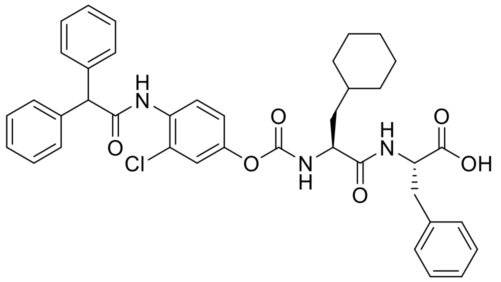	**30**	13,800 ± 1100	64[e]	3490 ± 30	42[e,f]
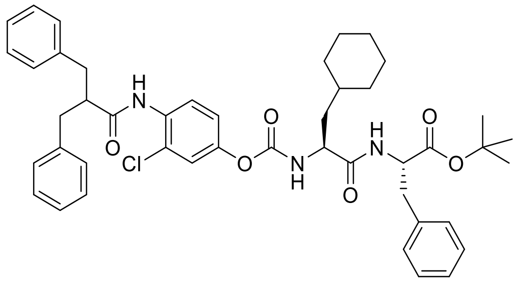		70[e]	68[e]	42[e,f]	19.7 ± 3.5
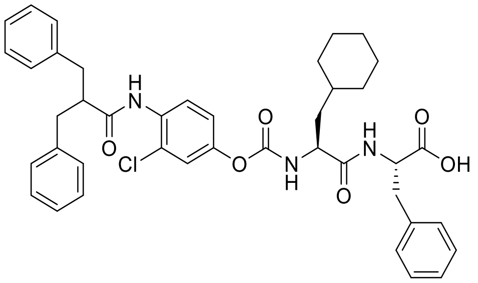	**31**	23,600 ± 1900	76[e]	23.1 ± 1.4	46[e,f]

[a] Values without standard errors refer to the percentage residual cathepsin activity from duplicate measurements at an inhibitor concentration of 20 µM. The reactions were followed for 60 min. [b] Values with standard errors refer to the second-order rate constant of enzyme inactivation from duplicate measurements with five different inhibitor concentrations. Progress curves were monitored for 60 min. For details, see chapter Enzyme Inhibition Assays. [c] Concentration dependent measurements were not performed when the residual activity of cathepsin B in the presence of 20 µM inhibitor was above 70%. [d] Concentration dependent measurements were not performed when the residual activity of cathepsins L, K, and S in the presence of 20 µM inhibitor were above 50%. [e] The inhibitor was not soluble at an assay concentration of 20 µM. The IC_{50} values from duplicate measurements with two different, lower inhibitor concentrations were determined according to $IC_{50} = [I]/((P_0/P_1)-1)$, where [I] is the inhibitor concentration, P_0 is the product formation of the control (100%), and P_1 is the product formation in the presence of the inhibitor. The equation $P_1 = P_0/((20\ \mu M/IC_{50})+1)$ was then used to calculate the approximated percentage product formations at [I] = 20 µM and the mean value was listed in the Table. [f] No time-dependent inhibition; $k_{obs} < 5\times10^{-5}\ s^{-1}$ at [I] = 5 µM.

Table S4. Kinetic data of cathepsin B inhibition by two selected carbamates (**7** and **31**).

Compd		k_{inac}/K_i ± SE ($M^{-1}s^{-1}$)[a]	k_{inac} (s^{-1})[c]	K_i ± SE (µM)[b]
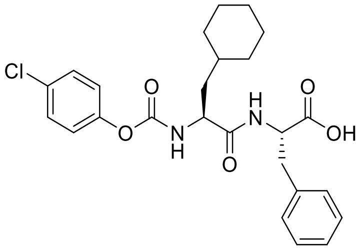	**7**	2010 ± 110	0.0200	9.96 ± 4.83 µM
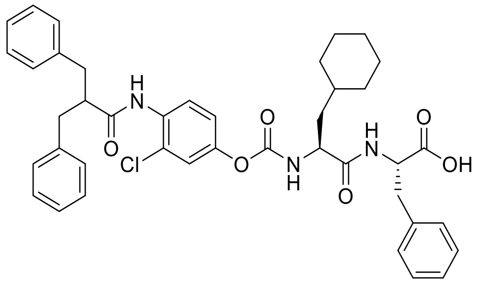	**31**	23,600 ± 1900	0.0104	0.441 ± 0.185

[a] Second-order rate constant of enzyme inactivation from duplicate measurements with five different inhibitor concentrations. Progress curves were monitored for 60 min. For details, see chapter Enzyme Inhibition Assays. First-order rate constants, k_{obs}, were determined by non-linear regression following the equation $[P] = v_i \times (1 - \exp(-k_{obs} \times t))/k_{obs} + d$, where [P] is the product concentration, v_i the initial product formation rate, k_{obs} is the observed first-order rate constant and d the offset. Mean values of k_{obs} were plotted *versus* corresponding inhibitor concentrations [I], and second-order rate constants of inactivation, k_{inac}/K_i, were obtained by linear regression. The equation $k_{obs}/[I] = (k_{inac}/K_i)/(1 + [S]/K_m)$ was used, where [S] is the substrate concentration and K_m is the Michaelis-Menten constant. Standard errors (SE) refer to the linear regression. [b] From non-linear regression of the time-dependent measurements using the equation $[P] = v_i \times (1 - \exp(-k_{obs} \times t))/k_{obs} + d$, values for the initial product formation rate, v_i, were obtained. These were plotted *versus* the inhibitor concentrations. To achieve K_i values, the equation $v_i = v_0/(1 + ([I]/K_i \times (1+[S]/K_m))$ was used for non-linear regression, where v_0 is the initial rate in the absence of inhibitor. Standard errors (SE) refer to the non-linear regression. [c] Values for k_{inac} were calculated from k_{inac}/K_i values and separately obtained K_i values.

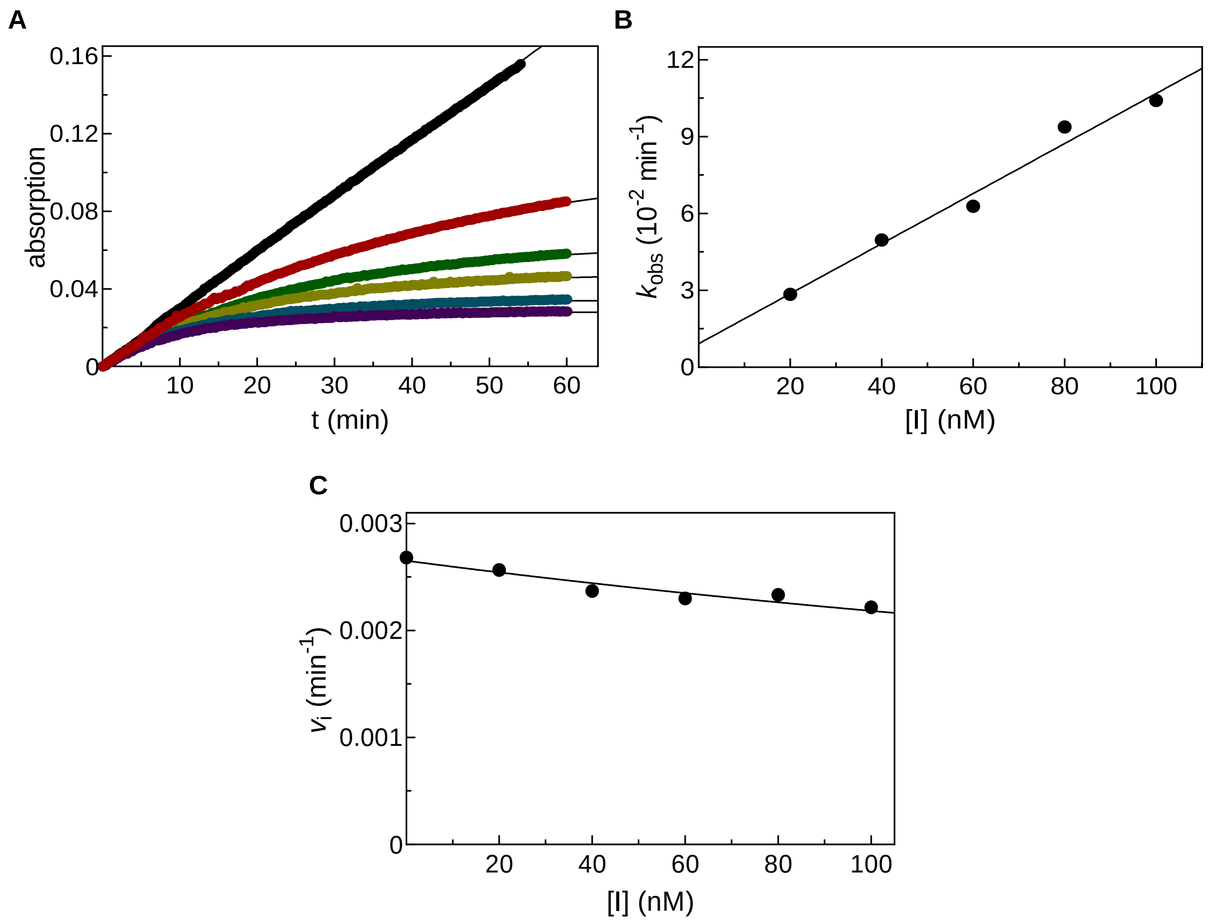

Figure S1. Inhibition of human cathepsin B by carbamate inhibitor **31**, see Table S4. (**A**) Progress curves were followed for 60 min in the absence (black) and presence of increasing concentrations of **31** (from top to bottom: 20 nM, 40 nM, 60 nM, 80 nM, 100 nM). Non-linear regression was performed using the equation [P] = $v_i \times (1 - \exp(-k_{obs} \times t))/k_{obs} + d$, where [P] is the product concentration, v_i the initial product formation rate, k_{obs} is the observed first-order rate constant and d the offset. (**B**) A plot of the first-order rate constants k_{obs} (mean values of duplicate measurements) *versus* the inhibitor concentrations [I] and linear regression according to the equation $k_{obs}/[I] = (k_{inac}/K_i)/(1 + [S]/K_m)$ was performed to obtain k_{inac}/K_i. (**C**) Values for the initial product formation rate, v_i, were plotted *versus* the inhibitor concentrations and non-linear regression according to $v_i = v_0/(1 + ([I]/K_i \times (1+[S]/K_m))$ was carried out to separately obtain K_i.

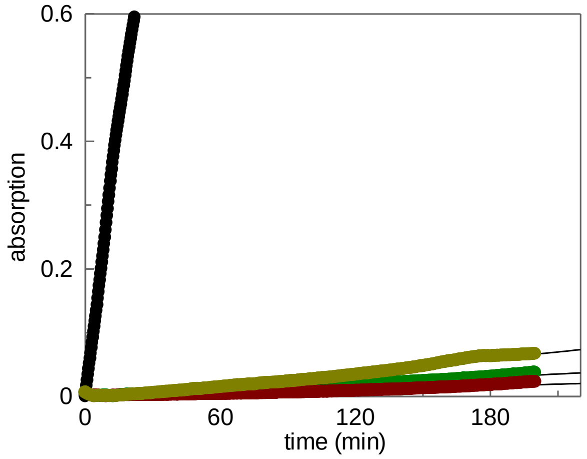

Figure S2. Reactivation experiment of human cathepsin B after incubation with the carbamate inhibitors **7** and **31**. Cathepsin B (38 µg/mL) was incubated under assay conditions for 30 min with • DMSO, • 5 µM of compound **7**, or • 0.5 µM of compound **31**. After removal of assay buffer, DMSO and excessive inhibitor by spin-filter centrifugation, product formation was followed in the presence of Cbz-Arg-Arg-pNA as substrate for 200 min. A negative control (•) containing only substrate and no enzyme was used to measure spontaneous decay of the substrate.

2. Synthetic Schemes

Scheme S1. Solution-phase synthesis of *O*-aryl-*N*-dipeptidyl-carbamates of type **A**.

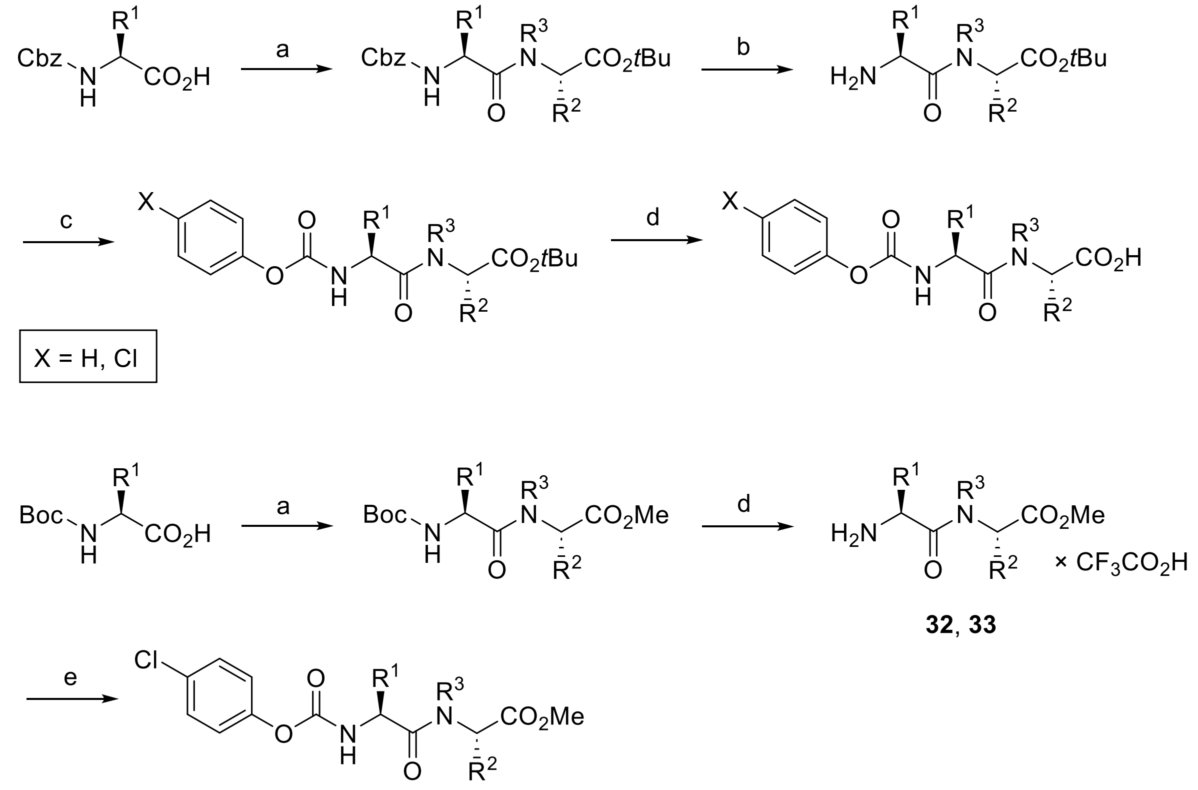

R^1 is the side chain of the P1′ amino acid. R^2 is the side chain of the P2′ amino acid. R^3 is either H or forms with R^2 the cyclic structure of proline. Reagents and conditions (H-AA-OH: amino acid): a) 1. HATU or EDC/DMAP, DIPEA, rt or *N*-methylmorpholine, $ClCO_2$*i*Bu, -25 °C, CH_2Cl_2, 2. H-AA-O*t*Bu (× HCl); b) Pd/C, H_2, MeOH, 30 psi, rt; c) $ClCO_2$-Ar, TEA, THF, rt; d) TFA, CH_2Cl_2, rt; e) $ClCO_2$-C_6H_4(4-Cl), TEA, THF, rt.

Scheme S2. Solid-phase synthesis of type **A** carbamates on a Wang resin.

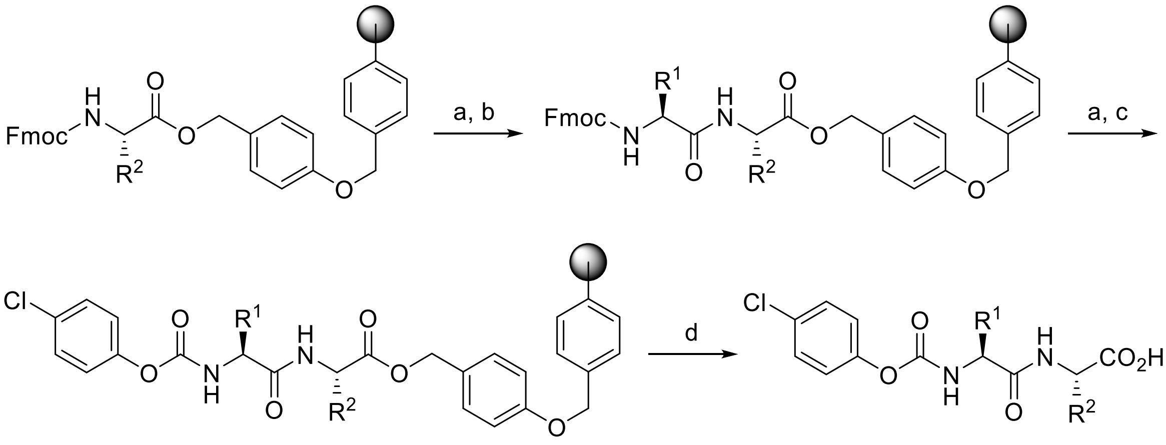

R^1 is the side chain of the P1′ amino acid. R^2 is the side chain of the P2′ amino acid. Reagents and conditions (H-AA-OH: amino acid): a) 20% piperidine, DMF, rt; b) Fmoc-AA-OH, HBTU, HOBt, DIPEA, DMF, rt; c) $ClCO_2$-C_6H_4(4-Cl), DIPEA, CH_2Cl_2, rt; d) 95% TFA, 2.5% TIPS, 2.5% CH_2Cl_2, rt.

Scheme S3. Solid-phase synthesis of type **A** carbamates on a 2-chlorotrityl resin.

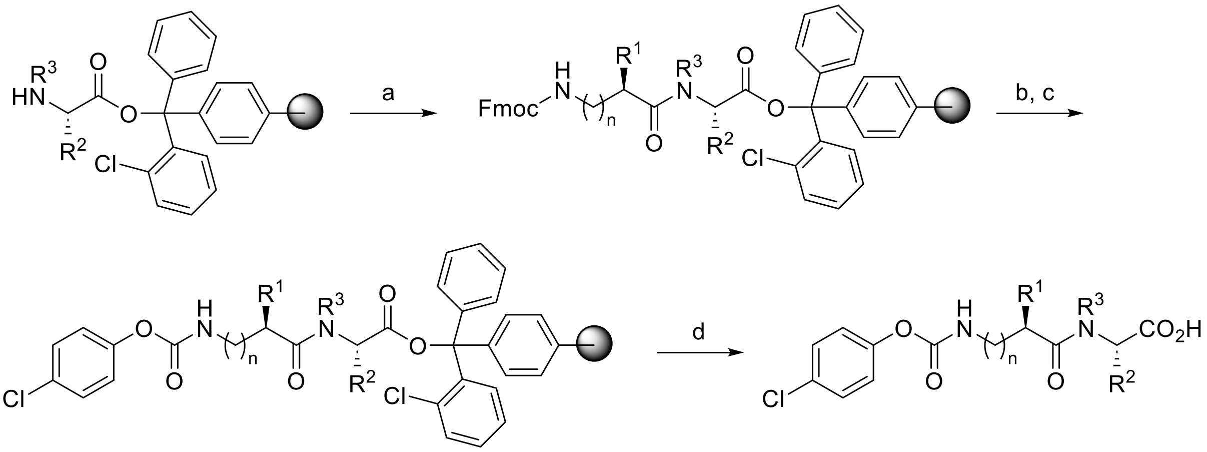

R^1 is the side chain of the P1′ amino acid. R^2 is the side chain of the P2′ amino acid. R^3 is either H or forms with R^2 the cyclic structure of proline or homoproline. For α-amino acids at the P1′ position, n = 0. For β-amino acids at the P1′ position, n = 1. Reagents and conditions (H-AA-OH: amino acid): a) Fmoc-AA-OH, HBTU, HOBt, DIPEA, DMF, rt; b) 20% piperidine, DMF, rt; c) $ClCO_2$-C_6H_4(4-Cl), DIPEA, CH_2Cl_2, rt; d) 2% TFA, 2% TIPS, 96% CH_2Cl_2, rt.

Scheme S4. Synthesis of Fmoc-APA-OH.

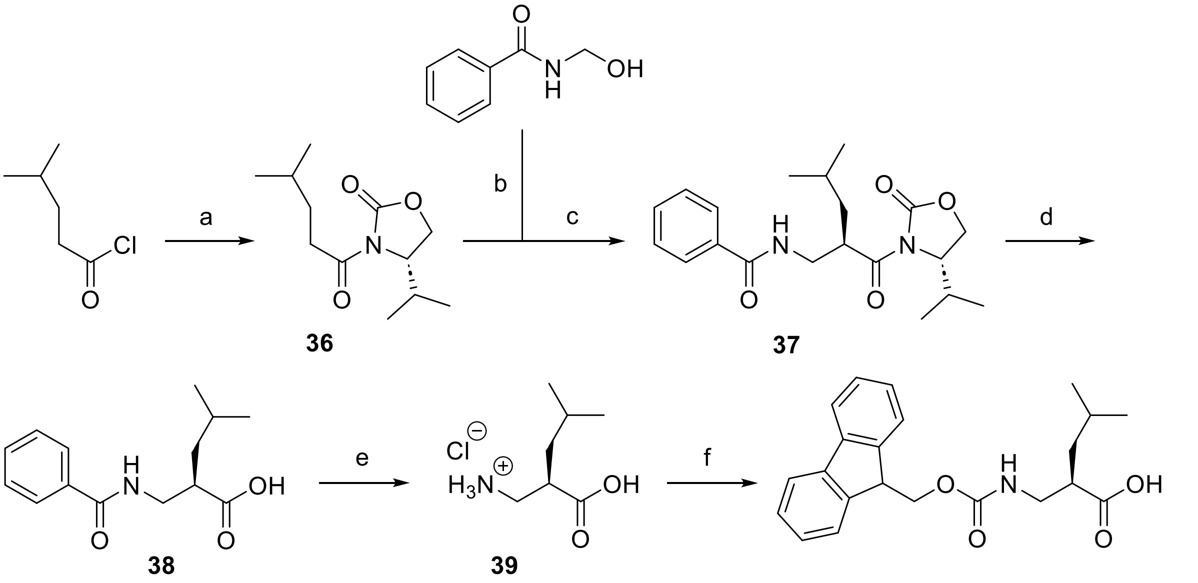

Reagents and conditions: a) (*S*)-4-isopropyloxazolidin-2-one, *n*-BuLi, THF, -78 °C to rt; b) $SOCl_2$, CH_2Cl_2, rt; c) $TiCl_4$, TEA, CH_2Cl_2, 0 °C; d) H_2O_2, LiOH × H_2O, THF / H_2O, 0 °C; e) HCl / AcOH / H_2O, reflux; f) Fmoc-OSu, TEA, 1,4-dioxane, H_2O, rt.

Scheme S5. Synthesis of *N*-aryl-carbamates of type **B**.

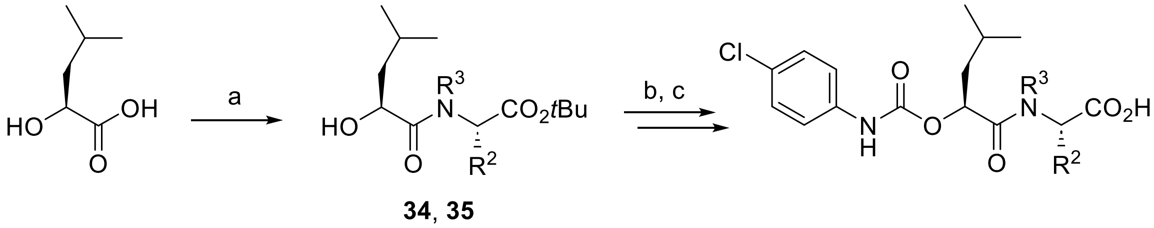

R^2 is the side chain of the P2′ amino acid. R^3 is either H or forms with R^2 the cyclic structure of proline. Reagents and conditions (H-AA-OH: amino acid): a) HOBt × H_2O, EDC × HCl, H-AA-O*t*Bu × HCl, TEA, DMF, rt; b) 4-Cl-C_6H_4-NCO, toluene, reflux; c) TFA, CH_2Cl_2, rt.

Scheme S6. Synthesis of *N*-aryl-ureas of type **B**.

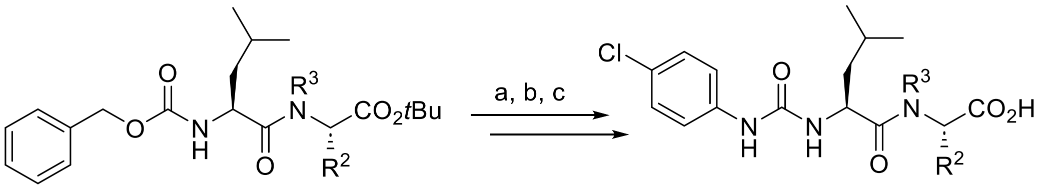

R^2 is the side chain of the P2′ amino acid. R^3 is either H or forms with R^2 the cyclic structure of proline. Reagents and conditions: a) Pd/C, H_2, MeOH, 30 psi, rt; b) 4-Cl-C_6H_4-NCO, CH_2Cl_2, rt; c) TFA, CH_2Cl_2, rt.

Scheme S7. Synthesis of *N*-substituted chlorophenols.

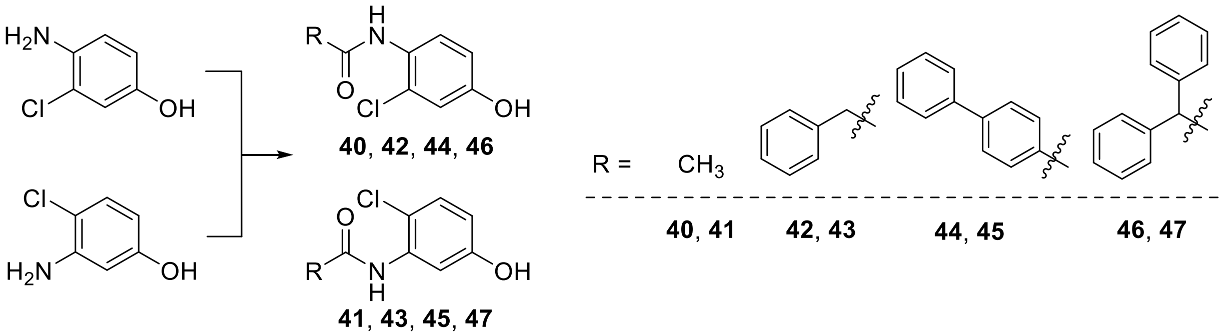

Reagents and conditions: 1. RCO_2H, *N*-methylmorpholine, $ClCO_2$*i*Bu, -25 °C or HATU, DIPEA, rt, CH_2Cl_2, 2. 4-amino-3-chlorophenol or 3-amino-4-chlorophenol.

Scheme S8. Synthesis of a biaryl-phenol.

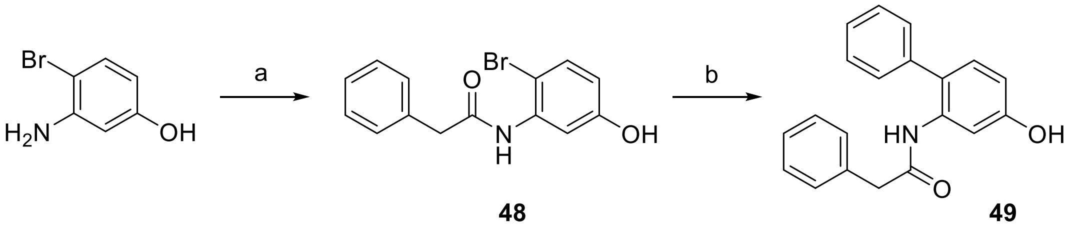

Reagents and conditions: a) 1. $BnCO_2H$, *N*-methylmorpholine, $ClCO_2$*i*Bu, Cl_2CH_2, -25 °C, 2. 3-amino-4-bromophenol; b) $PhB(OH)_2$, Na_3PO_4, $Pd(PPh_3)_4$, 1,4-dioxane, H_2O, 95 °C.

Scheme S9. Synthesis of *N*-substituted bromophenols.

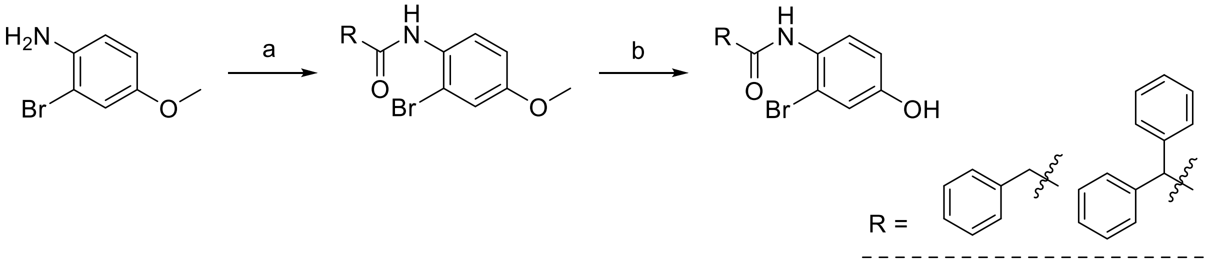

50 **51**

Reagents and conditions: a) RCO_2H, HBTU, DIPEA, THF, rt; b) BBr_3, CH_2Cl_2, -20 °C to rt.

Scheme S10. Synthesis of a 4-ureido-phenol.

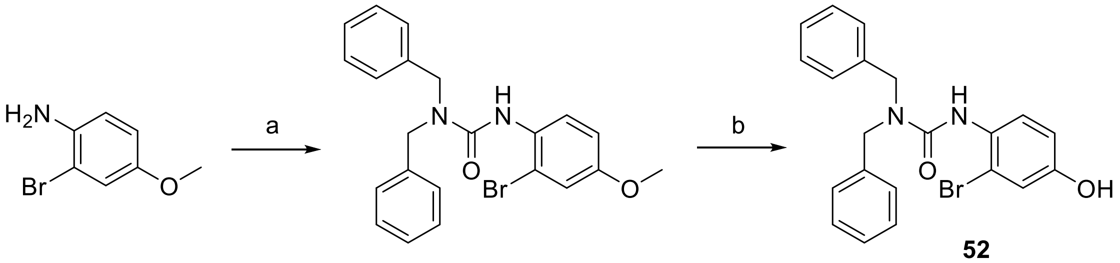

52

Reagents and conditions: a) 1. $ClCO_2$-C_6H_4(4-NO_2), DIPEA, CH_2Cl_2, -25 °C, 2. Bn_2NH, rt; b) BBr_3, CH_2Cl_2, -20 °C to rt.

Scheme S11. Synthesis of a 2-benzyl-3-phenylpropanamido-substituted phenol.

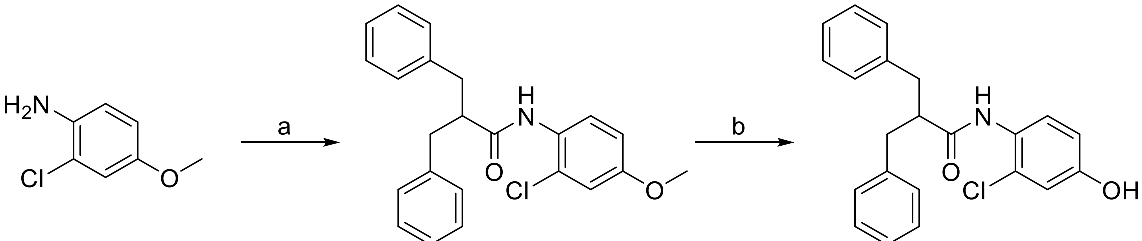

53

Reagents and conditions: a) Bn_2CHCO_2H, EDC × HCl, DMAP, DIPEA, CH_2Cl_2, rt; b) BBr_3, CH_2Cl_2, -20 °C to rt.

Scheme S12. Synthesis of *O*-aryl-*N*-dipeptidyl-carbamates of type **C**.

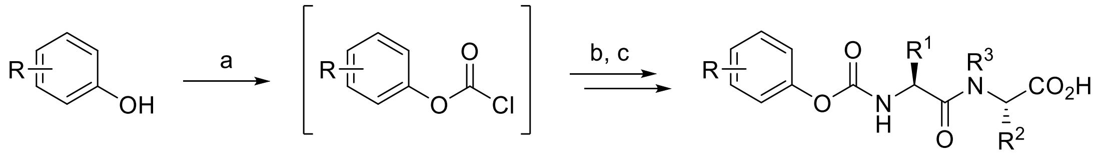

R^1 is the side chain of the P1′ amino acid. R^2 is the side chain of the P2′ amino acid. R^3 is either H or forms with R^2 the cyclic structure of proline. Reagents and conditions (H-AA-OH: amino acid): a) Triphosgene, DIPEA, THF, argon, 0 °C; b) H-AA-AA-O*t*Bu, DIPEA, rt; c) TFA, CH_2Cl_2, rt.

3. Molecular Modeling

The X-ray structure of human cathepsin B (PDB ID: 1CSB)[3] bound to the epoxide-derived inhibitor CA-030 was obtained from the RCSB Protein Data Bank.[4] The structure was used as a template for covalent ligand docking and modeling studies that were performed with the Molecular Operating Environment (MOE 2018.0101).[5] Prior to docking calculations, the bound inhibitor and crystallographic water molecules were removed from the template structure. The protein structure was prepared using MOE implementations to add hydrogens and atomic partial charges, correct orientations of Asn/Gln/His and optimize the Hydrogen-bond network (pH 6.0). To model the CatB-dipeptidyl-thiocarbamate adduct, the covalent docking module of MOE was utilized by the application of default postprocessing protocols. Based on a reaction/transformation placement methodology, the covalent bond between the reactive carbamate moiety of the ligand and the thiol group of Cys29 was formed. Possible binding modes of non-covalently bound inhibitor structures were generated with the general docking functionality of MOE. Putative binding modes of (covalently) bound inhibitors were selected after visual inspection of high-scoring docking poses.

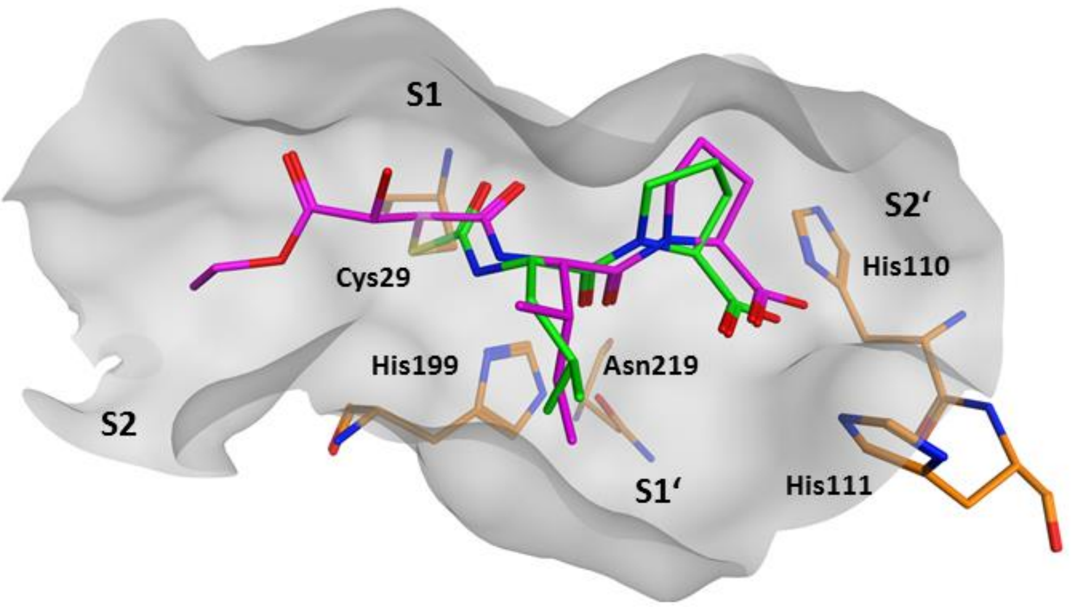

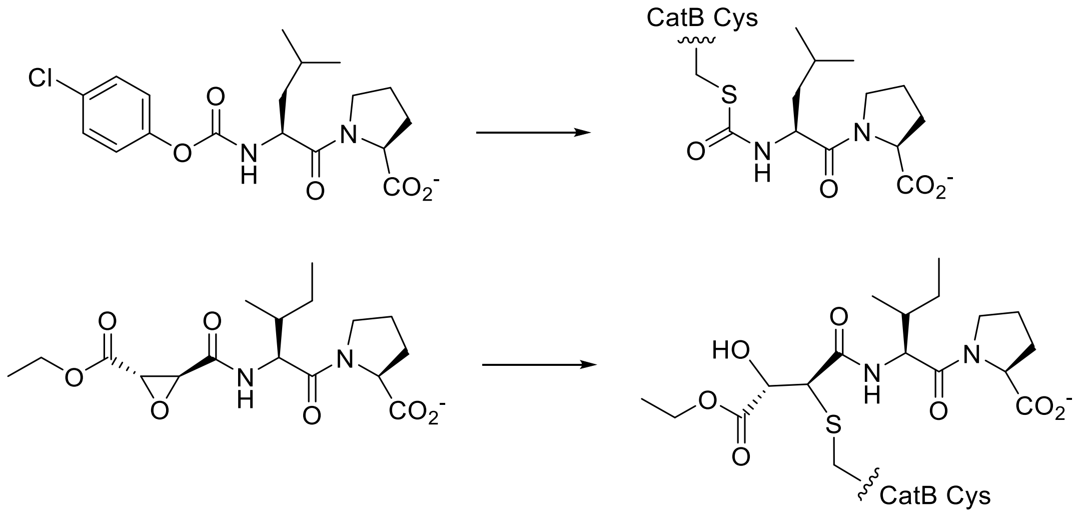

Figure S3. Top. Covalent docking of the carbamate **8** (green) bound to the active site of human cathepsin B. Crystallographic binding mode of CA-030 (magenta). Selected amino acids residues of the protein are shown (orange; PDB-ID: 1CSB).[3] Bottom. Formation of covalent enzyme-inhibitor complexes in the course of inactivation of cathepsin B by CA-030 and carbamate **8**.

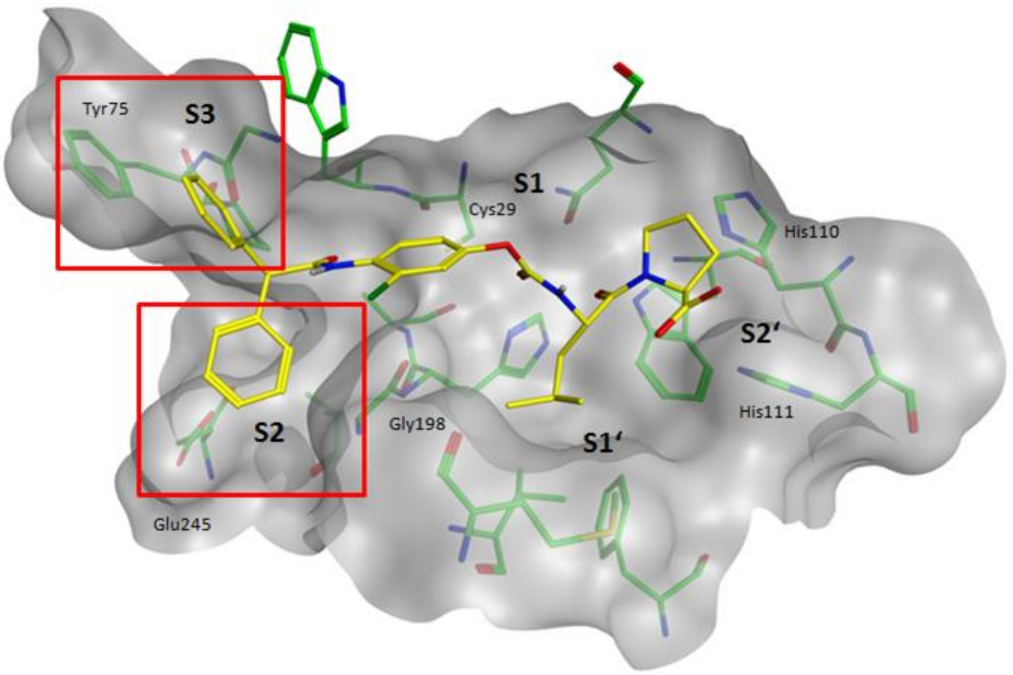

Figure S4. Docking of the carbamate **30** (yellow) bound to the active site of human cathepsin B. Selected amino acids residues of the protein are shown (green; PDB-ID: 1CSB).[3] Red rectangles indicate space for structural expansion.

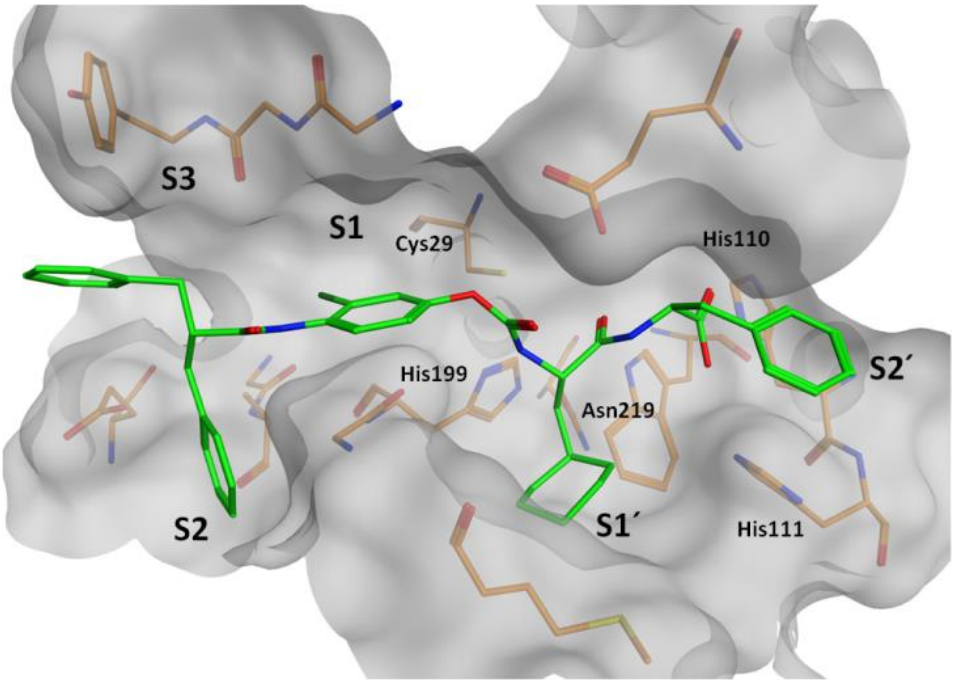

Figure S5. Docking of the carbamate **31** (green) bound to the active site of human cathepsin B. Selected amino acids residues of the protein are shown (orange; PDB-ID: 1CSB).[3]

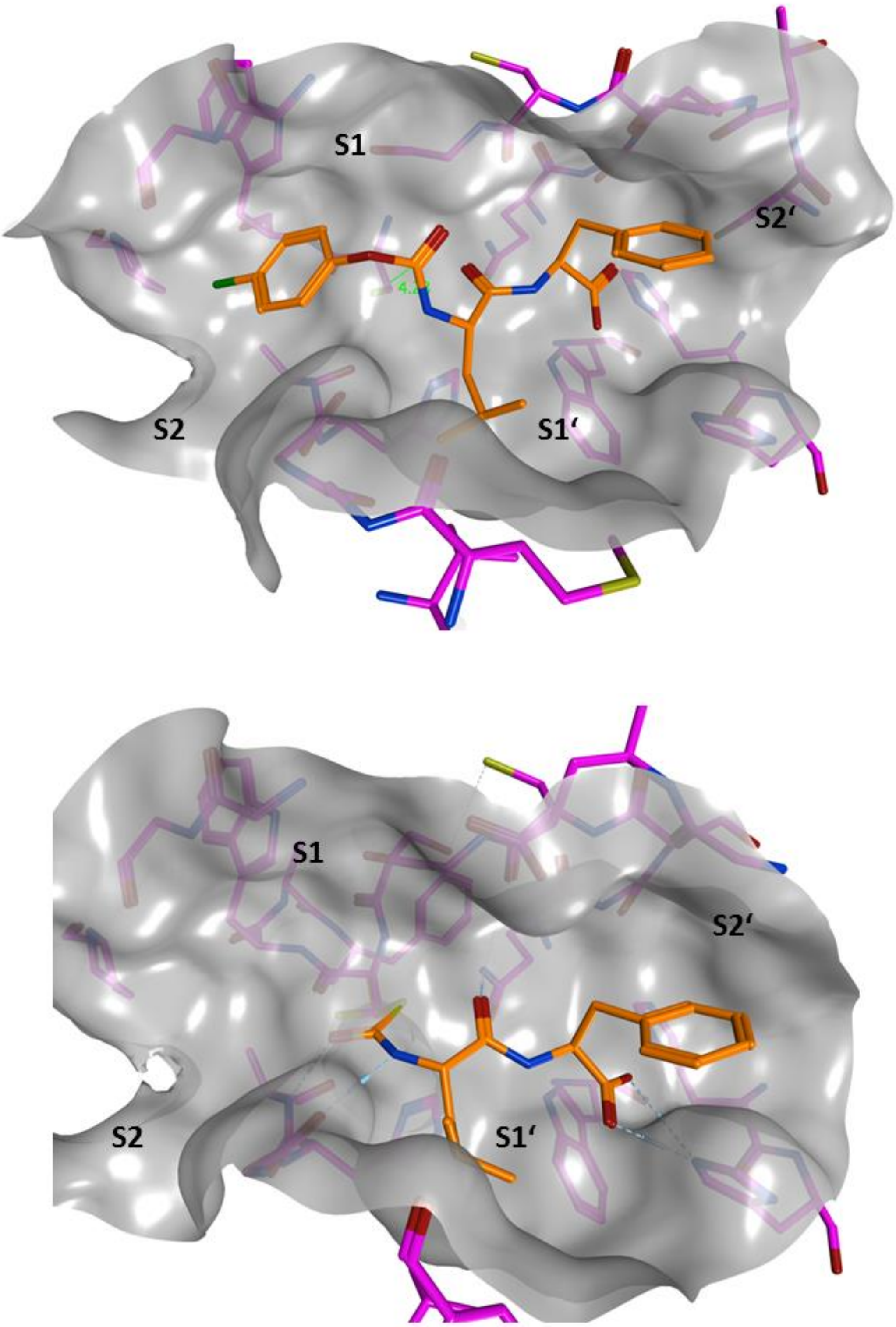

Figure S6. Top. Docking of the carbamate **4** (orange) bound to the active site of human cathepsin B. Bottom. Covalent docking of the inhibitor (orange) after carbamoylation of the active site cysteine. Selected amino acids residues of the protein are shown (magenta; PDB-ID: 1HUK).[6]

4. QM/MM Calculations

Computational methods. All molecular dynamics (MD) simulations were performed using the AMBER program package.[7] The applied force fields were ff99SB[8] and ff14SB.[9] GAFF[10] in combination with the program antechamber[11] was used for the parametrization of the ligands. An octahedral TIP3P[12] water shell with a radius of 30 Å was added around the parameterized system with help of the module AMBERTOOLS 1.4.[7] Spherical boundary conditions were applied to the system and the net charge was neutralized by the addition of sodium ions. A procedure containing three distinct calculation steps was applied to all systems in preparation for the actual MD run. First, a geometry optimization of only the water shell was performed, followed by an optimization of the whole system. After that, a short MD simulation, running over 400 ps with time steps of 1 fs, was performed. The protein was kept within weak constraints during the simulation and the temperature was gradually increased from 0 K to 300 K. The simulation times of the subsequent MD calculations were adapted to the respective problem. The specific data is given in the respective sections. For temperature controlling, a Langevin thermostat was used. The SHAKE algorithm was applied to bonds that involved a hydrogen atom.

Quantum mechanics (QM) calculations were carried out as follows. All quantum mechanical ab-initio calculations were performed, using the Turbomole program package.[13] For the simulation of a solvent surrounding, the implicit solvation model COSMO[14] was used. Semi-empirical calculations were performed using the MNDO 7.0 program package.[15]

Quantum mechanics/molecular mechanics (QM/MM) calculations were carried out as follows. The program ChemShell 3.6[16] was used as an interface within the performed QM/MM calculations. All systems calculated on a QM/MM level were taken from preceding MD calculations and therefore included the TIP3P water shell and the neutralizing sodium ions. The applied boundary treatment included electrostatic embedding in combination with the charge shift method and the link atom approach. All QM/MM optimizations were performed using the DL-FIND module[17] of ChemShell with an active area of 10 Å around the reaction center of the enzyme. The rest of the system was kept fixed during the calculations. For all QM/MM calculations, the MM part was calculated with the AMBER program package and the 14SB force field. The QM calculations were performed with the Turbomole 6.6 and 7.1 program package. For the Nudged Elastic Band (NEB) calculations, the same boundary treatment as described above was utilized. The calculations were performed with different counts of frames, reaching from 7 up to 17.

The programs used for the analysis and visualization of the results were OriginPro 2016,[18] and VMD 1.9.1.[19]

General. The molecular docking results of inhibitor **4** to the active site of human cathepsin B (Figure S6) were taken as a basis for theoretical investigations on the proposed mechanism of the enzyme-inhibitor interaction. Both the noncovalent Michaelis-Menten complex and the covalent thiocarbamate complex were included in the calculations. The mechanisms of the reaction of carbamate inhibitors with the active-site residues of human cathepsin B were investigated using different computational approaches including NEB calculations as well as reaction profiles and potential energy surfaces. Initially, various reaction pathways of the carbamate inhibitor in the active site of cathepsin B were evaluated via thermodynamic calculations (SCS-MP2/aug-cc-pVDZ with COSMO; ϵ=78.93). For this purpose, a model system was set up, containing only the side chains of histidine and cysteine (the backbone was replaced by a methyl residue) as well as the warhead of the inhibitor. After preliminary calculations, alternative mechanisms could be excluded leading to the remaining feasible one that describes an elimination reaction according to an E1cB mechanism with a proton transfer from cysteine to the phenolate leaving group and a subsequent carbamoylation of cysteine. The model system was then enlarged to the whole enzyme including a docked inhibitor structure.

Calculations about the stabilization of the phenolate anion. The stabilization of the phenolate leaving group was examined. A MD simulation with a frozen QM area containing the dissociated inhibitor, represented by its charge-density, was performed in order to recognize stabilizing interactions. The enzymatic environment did not undergo significant changes, whereas a small water shell surrounded the anion. One water molecule was observed to diffuse into the active site. However, a quantification of the stabilizing effect was not possible, as the structural changes of the environment around the active site dominated the energy of the compared systems. A fixation of the surrounding amino acids lead to a destabilization of the structure with the water inside the active site by 10.8 kcal/mol, as the adjacent fixed molecules could not adapt to the water molecule. Next, the protonation of the formed phenolate through Cys29 was investigated using density functional theory (DFT) methods. The deprotonation of the inhibitor was assumed to be the first step of the reaction with the subsequent dissociation and protonation of phenolate. The deprotonation was computed using a reaction profile (B3LYP/aug-cc-pVDZ with COSMO; ϵ=78.93) with the help of a potential energy surface (B3LYP/aug-cc-pVDZ with COSMO; ϵ=78.93). The resulting pathway has an energy barrier of 28.8 kcal/mol. In an attempt to investigate if a reaction path with a lower barrier exists, the possibility of the three steps (deprotonation, dissociation and protonation of phenolate) occurring simultaneously was investigated via multiple potential energy surfaces (B3LYP/aug-cc-pVDZ with COSMO; ϵ=78.93). However, the resulting reaction barrier still remained at 32.9 kcal/mol.

Calculations of the water-mediated inhibitor cleavage. NEB calculations were performed for the system with a water-mediated proton transfer (Figure S7). The calculation describing the whole E1cB elimination including the protonation of the phenolate (**NEB12_wat**) did not converge, as the structural changes during the reaction are too drastic. The two calculations NEB1_wat and NEB2_wat did however converge. Figure S8 depicts the resulting reaction path in one normalized coordinate with equidistant

marks (one for each frame of the calculation). The reaction barrier of the deprotonation (15.3 kcal/mol) is similar to the respective barrier calculated from the potential energy surfaces. The barrier for the dissociation is now significantly lower at 9.8 kcal/mol and the reaction path does not include an isolated hydronium ion. The small barriers at 0.2 and 0.7 on the reaction coordinate (Figure S8) result from a reorientation of the water molecule. The water-mediated proton transfer between the inhibitor and Cys29 occurs as a concerted reaction. The abstraction of the proton ($r(O_AH_1)$) changes simultaneously to the protonation of Cys29 ($r(S\text{-}H_{A1})$). After cysteine deprotonation, His199 rotates away from the sulfur atom towards the recognition unit of the inhibitor (r(S-His) in Figure S8). At 0.7-0.9 of the reaction coordinate, the protonation of phenolate ($r(O_1\text{-}H_{A2})$) takes place simultaneously to the dissociation of the inhibitor ($r(C_1O_1)$). The results of the NEB calculation show that the reaction depends on a number of different coordinates and is partially concerted. The inclusion of a water molecule leads to a significant reduction of the reaction barriers and results in an exothermic reaction with a barrier of only 15.3 kcal/mol.

Calculations on the conversion of the inhibitor-derived isocyanate. Starting from structure **W3_wat** (Figure S7), structure **W3** (Figure S9) was formed through the removal of phenol from the system and the exclusion of the water molecule from the active site. Starting from this structure, QM/MM potential energy surfaces (PM3 as QM method) were calculated in order to generate the structures **W4** and **W5** (Figure S9) with the additional water molecule inside the active site. All structures converged in a subsequent unconstrained QM/MM optimization with B3LYP/cc-pVDZ as QM method. The simulations of W5 (Figure S10) resulted in a converged product structure within a relaxed environment. Figure S11 illustrates the structures that are taken for the following NEB calculations. NEB calculations were performed at the QM/MM level of theory in order to investigate the carbamoylation of Cys29 through the isocyanate intermediate. One computation included the whole mechanism, starting from structure **W3** with structure **W5** as the reaction product. Based on results of a first NEB calculation, a second calculation was carried out, going from **W4** to **W5'** (Figure S12). The resulting reaction pathways (**W3** to **W4** and **W4** to **W5'**) were again combined in one normalized axis with equidistant marks (one per frame). The attack of the nucleophilic sulfur at the isocyanate carbon has a reaction barrier of 5.1 kcal/mol. During this step, the water molecule already starts approaching to His199. The reaction has an enthalpy of -13.0 kcal/mol and segues nearly barrier-free into the next step, a water bridged proton transfer. The deprotonation of His199 and the protonation of the inhibitor-derived fragment through a water molecule occurs simultaneously and results in the product **W5'**.

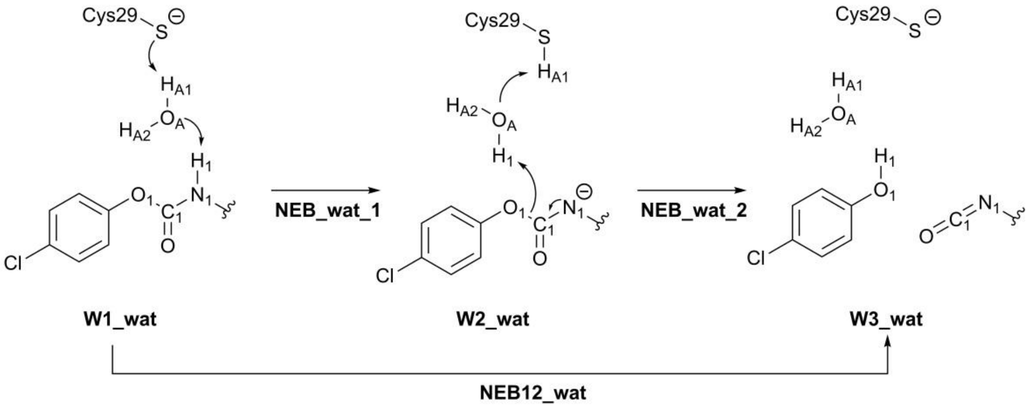

Figure S7. Reaction scheme of the performed NEB calculations with a water-mediated proton transfer.

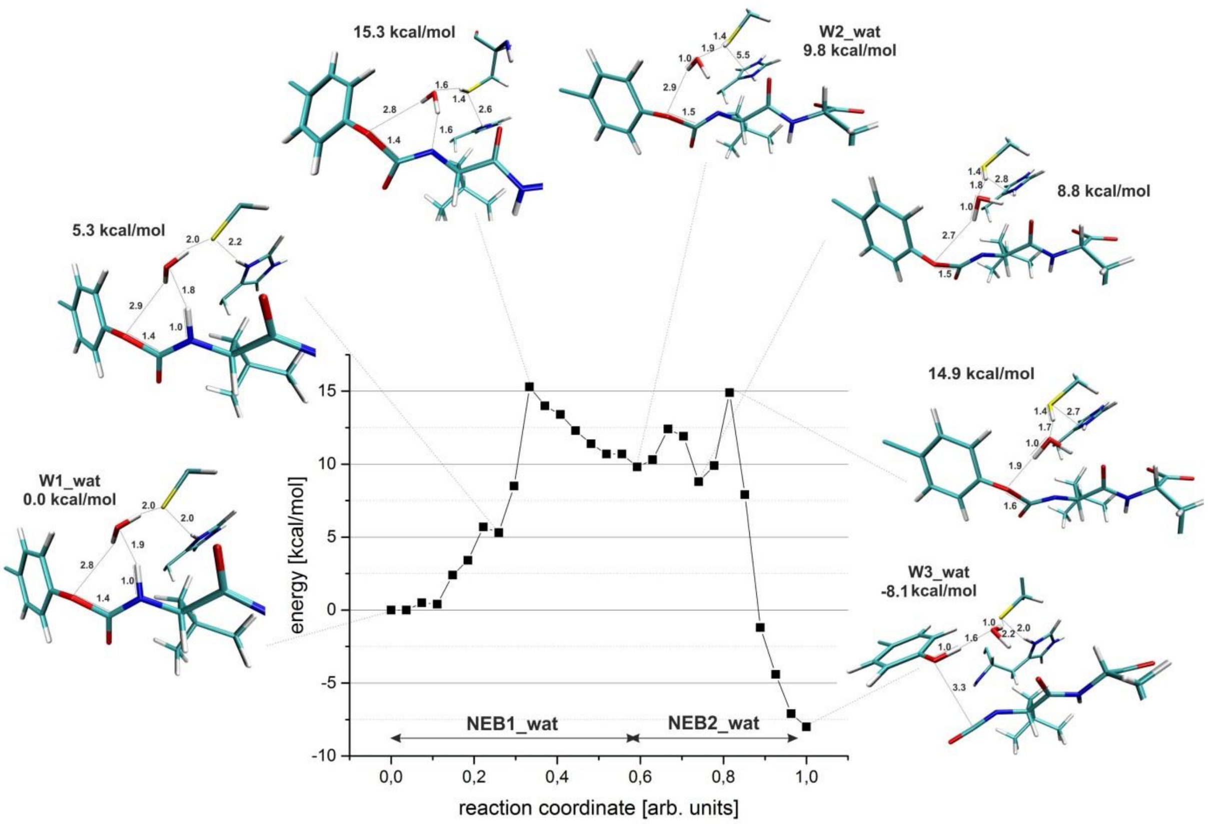

Figure S8. NEB path (B3LYP/cc-pVDZ as QM method) of the reaction from **W1_wat** to **W3_wat**. NEB profiles from **W1_wat** to **W2_wat** and from **W2_wat** to **W3_wat** were combined in one reaction coordinate, the x-axis therefore contains equidistant marks, one for each frame of the calculation.

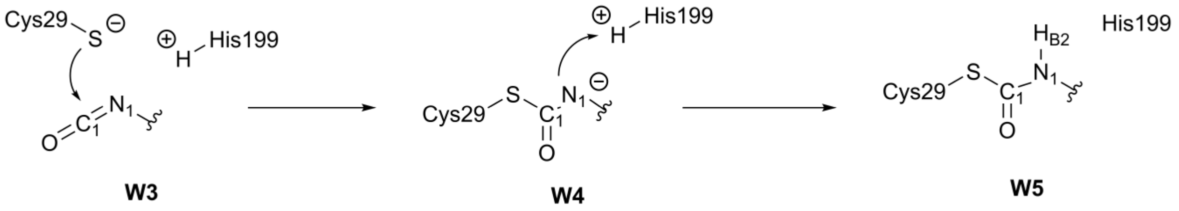

Figure S9. Reaction scheme of the carbamoylation of Cys29 with an isocyanate and His199.

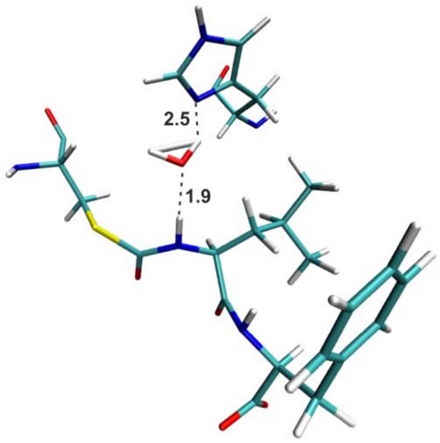

Figure S10. Product structure **W5** within a relaxed environment, including a water molecule in the active site. The distances are given in Å.

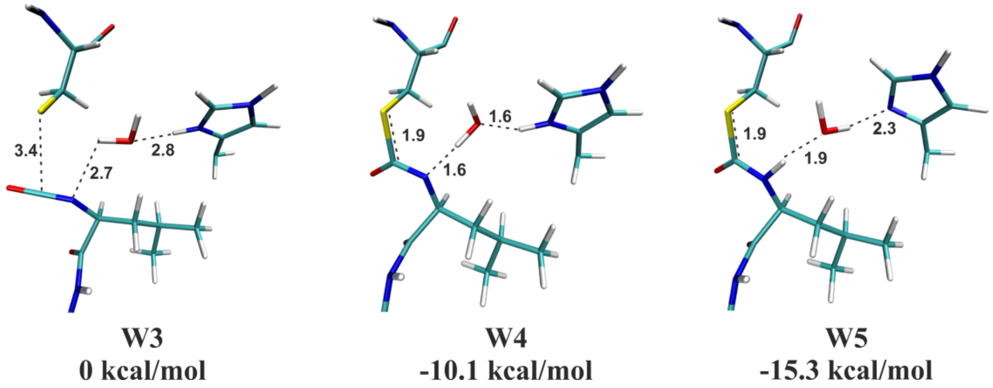

Figure S11. Converged structures **W3**, **W4** and **W5** with a water molecule in the active site.

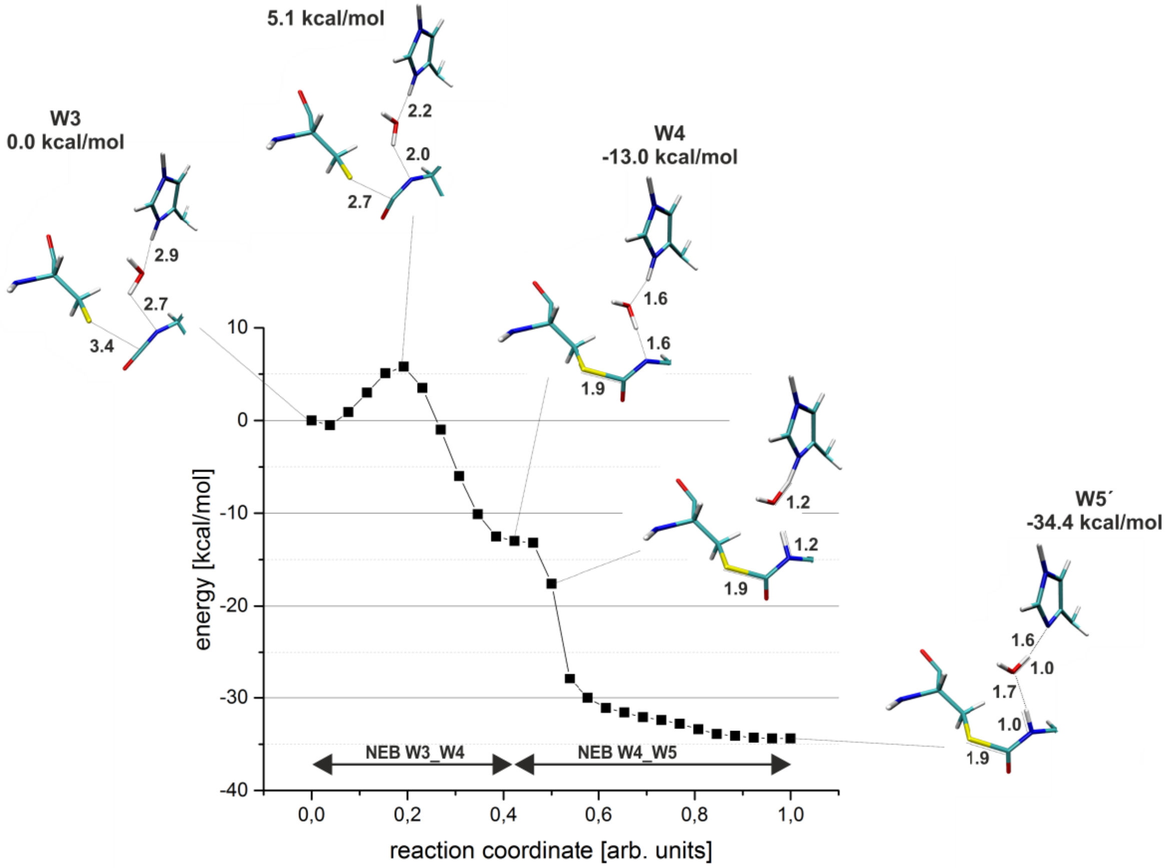

Figure S12. QM/MM NEB path (B3LYP/cc-pVDZ asQMmethod) of the carbamoylation of Cys29 through the isocyanate intermediate. NEB profiles from **W3** to **W4** and from **W4** to **W5** were combined in one reaction coordinate. The x-axis therefore contains equidistant marks, one for each frame of the calculation.

5. Crystallographic Analysis

Crystal structures of human cathepsin B in complex with two carbamate inhibitors and binding mode analysis. Recombinant human cathepsin B was crystallized in complex with inhibitors **7** and **31**. The final crystallographic models contain residues 0–254, including mature enzyme residues 1–254 and the residue 0 from an incompletely processed propeptide. The structures were refined to resolutions of 1.45 Å for the **7** complex and 1.70 Å for the **31** complex. The electron density map used for modeling of inhibitors was of high quality for both structures (Figure S13). The root-mean-square deviation (RMSD) alignment of the Cα atoms of the cathepsin B structures was 0.09 Å, and no significant differences in the protein structures were observed. The RMSD alignment of all atoms of bound inhibitors **7** and **31**, which share the same carbamoylating dipeptide structure, was 0.12 Å indicating their analogous conformation in the active site.

The inhibitors **7** and **31** form the covalent complex with cathepsin B through its catalytic Cys29, and the covalent bond between the reacted warhead of the inhibitor and Cys29 was clearly demonstrated by electron density (Figure S13). The active site cleft contains the catalytic dyad of Cys29 and His199 (supported by Gln23 forming the oxyanion hole). These three residues form hydrogen bonds with the reacted warhead or its close vicinity (the inhibitor segment –CO–NH–) in the subsites S1 (Cys29 and Gln23) and S1′ (His199). The dipeptide part of inhibitors occupies the subsites S1′ and S2′ of cathepsin B, which are bordered by the occluding loop, an insertion of 23 residues (residues 104–126)[20] that is a characteristic of cathepsins B.

The P1′ residue of cyclohexyl alanine forms a hydrogen bond through the peptide carbonyl to the side chain nitrogen of Trp221. The hydrophobic S1′ pocket in the R domain of the enzyme is filled by the cyclohexyl moiety forming hydrophobic interactions with Val176, Leu181, Met196, and Trp221. Higher B-factors (Figure S14B) suggest certain flexibility of the cyclohexyl ring, which is also in line with differences in the network of contacts of this ring in **7** and **31** with the S1′ pocket (Table S5).

The P2′ phenylalanine of the inhibitors occupies a shallow S2′ pocket in the L domain of the enzyme. The flexible phenyl ring is oriented towards the wall formed by residues Gly121 to Asp124 in the occluding loop, which is pushed out of the active site, while residues Gly121 to Cys119 form a number of contacts with the phenyl ring but no electrostatic/hydrophobic interactions. An important part of the occluding loop are two histidine residues, His110 and His111, which interact with the C-terminus of substrates and are responsible for the exopeptidase specificity of cathepsin B. We found that the C-terminal carboxy group of the inhibitors **7** and **31** interacts specifically with His110 and His111 via two hydrogen bonds. The same pattern of hydrogen bonds has previously been observed for the C-terminal carboxy group of a cathepsin B-specific inhibitor, CA-030, in complex with human cathepsin B (PDB ID: 1CSB).[3]

In conclusion, although the inhibitors **7** and **31** differ in their western (leaving) parts, their crystallographic analysis confirmed the same carbamoylating dipeptide structure in the active site of cathepsin B. The leaving groups of **7** and **31** released during the reaction of the inhibitors with the

catalytic Cys29 residue were not identified in the crystal structures of the complexes. The carbamoylating dipeptide parts possess an analogous binding mode and form an almost identical network of interactions in the S1′ and S2′ subsites of cathepsin B.

Preparation of recombinant human cathepsin B. A nonglycosylated mutant (Ser115→Ala) of the human cathepsin B zymogen (Uniprot accession P0785) was expressed using the pPICZαA vector in the yeast *Pichia pastoris* analogously as described previously.[21] The yeast medium was harvested 3 days after induction, concentrated by ultrafiltration, and buffer exchanged to 20 mM Bis-Tris propane pH 6.5. The zymogen was purified on a Mono Q 5/50 GL column (GE Healthcare Bio-Sciences, Uppsala, Sweden) equilibrated with 20 mM Bis-Tris propane pH 6.5 and eluted using a linear gradient of 0–0.5 M NaCl. The zymogen was activated in 100 mM sodium acetate pH 4.5, 2.5 mM DTT for 1 h at 25 °C under an argon atmosphere to prevent the active site cysteine residue from oxidation. Mature cathepsin B was incubated with a 4-fold molar excess of the inhibitor **7** or **31** for 8 h under the same conditions. The conversion to the mature enzyme was monitored using SDS-PAGE, and the complexation reaction was monitored using the activity assay.[22] The complexes were buffer exchanged to 20 mM sodium acetate pH 5.5, containing 20 μM inhibitor and concentrated to 7.2 and 9.2 mg/mL for the **7** and **31** complexes, respectively.

Protein crystallization and data collection. Crystals were obtained by vapor diffusion in a hanging drop. Drops consisting of 1 μL of protein solution and 1 μL of reservoir solution were equilibrated against 0.5 mL of reservoir solution at 18°C. Crystals of the **7** complex were grown for 5 days from 100 mM sodium acetate pH 5.5, 20% polyethylene glycol 2000 monomethyl ether, and crystals of the **31** complex from 0.1 M sodium acetate pH 5.5, 30% polyethylene glycol 5000 monomethyl ether for 5 days. The obtained needle-shaped crystals were flash-cooled by plunging into liquid nitrogen with cryoprotection (100 mM sodium acetate pH 5.5, 35% polyethylene glycol 750 monomethyl ether). Diffraction data were collected at 100 K on MX 14.1 operated by the Joint Berlin MX-Laboratory at the BESSY II electron storage ring in Berlin-Adlershof, Germany.[23] Diffraction data were processed using the XDS suite of programs.[24] Crystal parameters and data collection statistics are given in Table S6.

Structure determination, refinement, and analysis. The inhibitor complexes were crystallized in the orthorhombic space group $P2_12_12_1$ with one molecule in the asymmetric unit and a solvent content of ~41%. The structures were determined by molecular replacement with the program Molrep[25] from the CCP4 package[26] using the structure of uncomplexed cathepsin B as a search model (PDB ID: 2IPP) for the complex of **7**, which was subsequently used as a search model for the complex of **31**. Model refinement was carried out using the program REFMAC 5.8.0258 from the CCP4 package,[26] interspersed with manual adjustments using Coot.[27] The structures were refined using data to a resolution of 1.45 and 1.70 Å for the complexes of **7** and **31**, respectively. The geometric restraints for inhibitors **7** and **31** were generated by the program Libcheck.[26] The model was validated using the wwPDB Validation Server[28] and MolProbity.[29] Final refinement statistics are given in Table S6. Atomic coordinates and structure factors have been deposited in the Protein Data Bank with accession

codes 8B4T for the **7** complex and 8B5F for the **31** complex. The inhibitor-enzyme interactions were analyzed using the programs CONTACT[26] and PLIP.[30] All figures showing structural presentations were prepared with the program PyMOL 2.5.0 (Schrödinger, New York, USA).

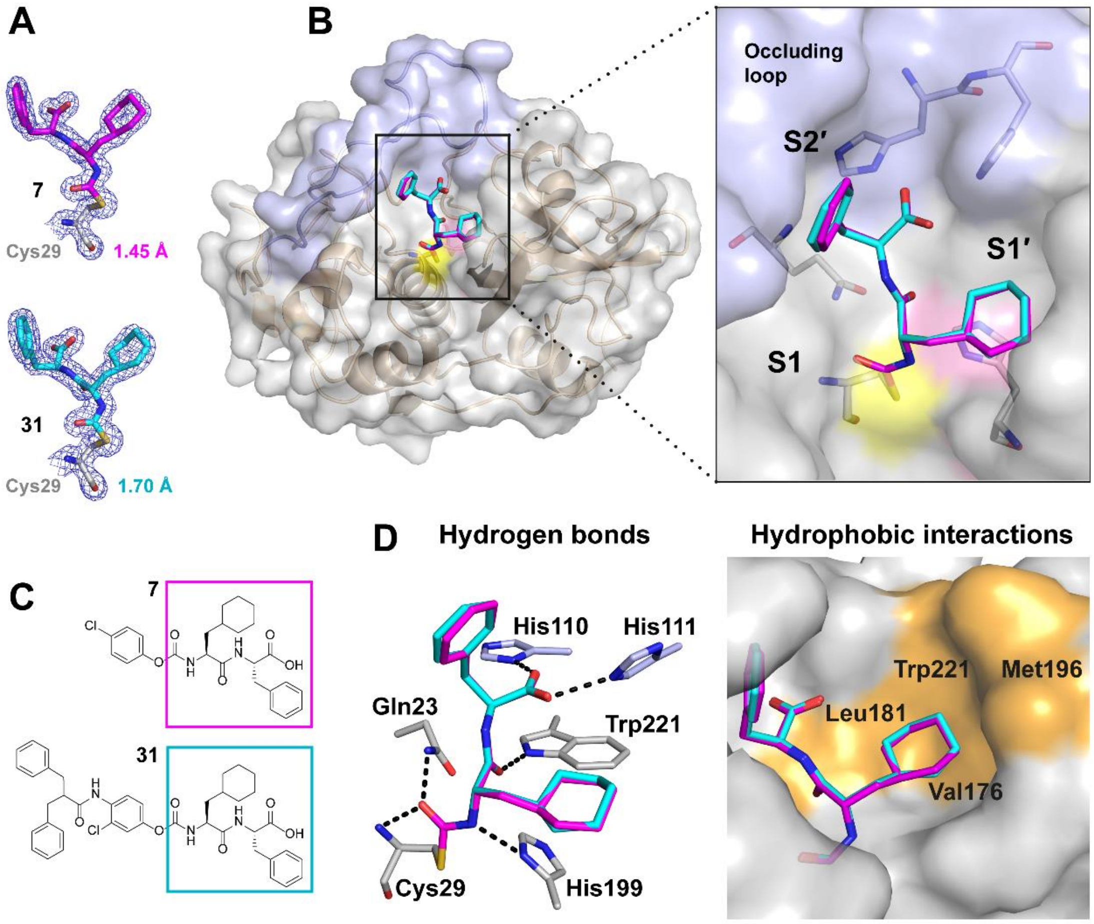

Figure S13. Crystal structures of the carbamate inhibitors **7** and **31** in complex with human cathepsin B and binding mode analysis. (**A**) Electron density maps for the inhibitors and catalytic Cys29 are contoured to 1.0 σ. (**B**) Overall crystal structure of human cathepsin B (surface representation) in complex with inhibitors (sticks representation). The catalytic residues Cys29 (yellow) and His199 (pink) are highlighted in the active site (boxed), and the occluding loop (residues 104–126) is colored light blue. The zoomed view of the cathepsin B active site shows the covalent thiocarbamate complex formed between Cys29 and the eastern part of the inhibitors occupying the subsites S1′ and S2′; the reacted warhead is bound in the S1 subsite. Side chains are presented for the catalytic residues and two binding histidine residues, His110 and His111. (**C**) The chemical structures of inhibitors **7** and **31**. The carbamoylating dipeptide parts of the inhibitors are boxed. (**D**) In the left panel, hydrogen bonds between cathepsin B (gray) and **7** (magenta) or **31** (cyan) are represented by dashed lines (black). Specific interactions are provided by His110 and His111 from the cathepsin B occluding loop. In the right panel, the cathepsin B residues that form hydrophobic interactions (the distance cutoff set to 4.2 Å) are highlighted in orange in the surface representation. Heteroatoms have standard color coding (O, red; N, blue; S, yellow).

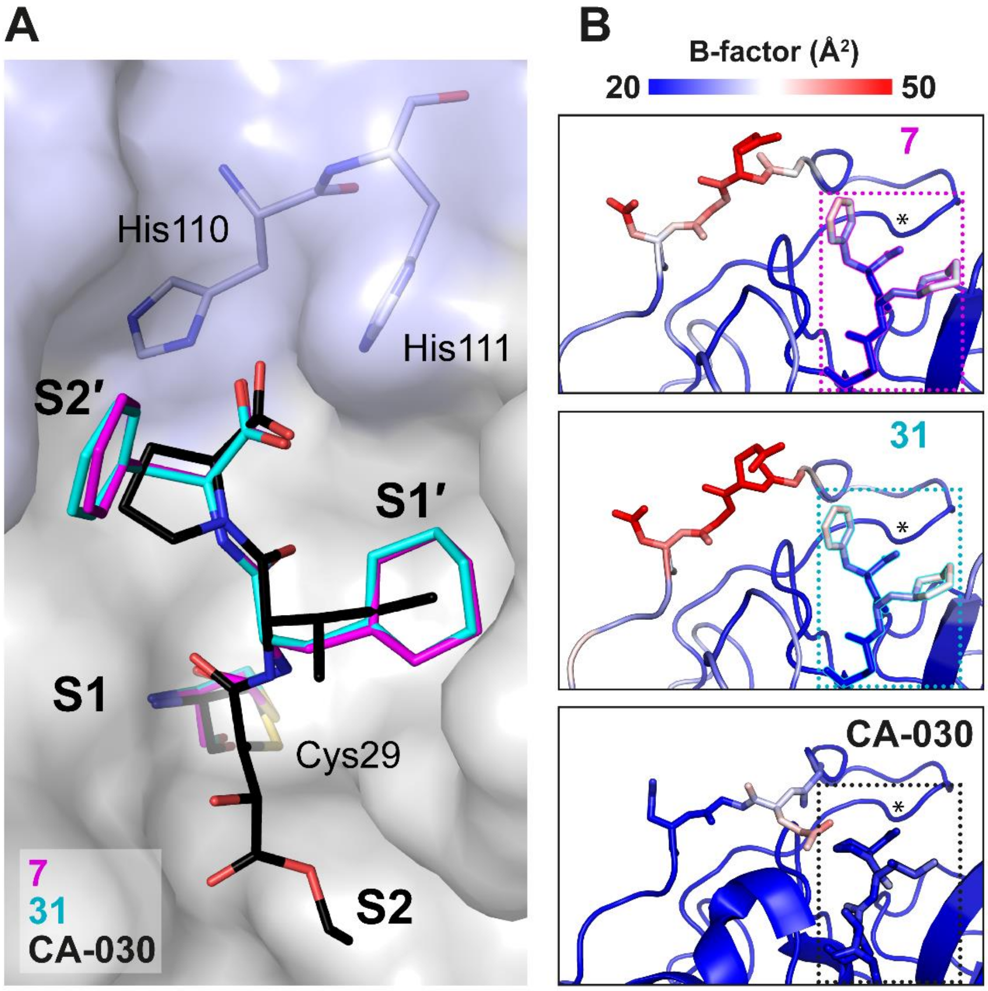

Figure S14. Comparison of the crystallographic binding modes of carbamate inhibitors and the inhibitor CA-030 (PDB-ID: 1CSB). (**A**) Superposition of the inhibitors **7** (magenta), **31** (cyan), and CA-030 (black) in the active site of human cathepsin B. In the occluding loop (light blue), residues His110 and His111 (sticks representation) are responsible for the specific interaction of all three inhibitors with cathepsin B. The enzyme subsites and the catalytic Cys29 are indicated. (**B**) Conformational flexibility of the occluding loop and inhibitors in the active site. Structures are colored according to atomic B-factor values, ranging from blue to red (low to high flexibility, respectively). The highest B-factors indicating flexibility are observed for the occluding loop segment Gly121–Asp124 (in sticks representation), which is pushed out of the active site in the complexes of **7** and **31** by the phenyl moiety of the inhibitor P2′ residue. The inhibitor is boxed, and the position of His110 and His111 residues is marked by an asterisk. Heteroatoms have standard color coding (O, red; N, blue; S, yellow).

Table S5. Crystallographic contacts formed between human cathepsin B and inhibitors **7** and **31**.[a]

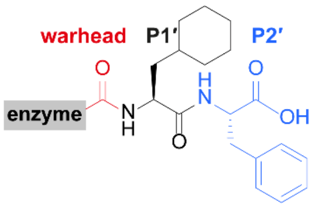

Positions	Cmpd **7**			Cmpd **31**		
	Residue	Contacts	H-bond	Residue	Contacts	H-bond
**P2′**	Gln23	4		Gln23	4	
	Gly24	1		Gly24	1	
	Cys26	3		Cys26	4	
				Gly27	1	
	**His110**	9	**1**	**His110**	9	**1**
	**His111**	7	**1**	**His111**	7	**1**
	Cys119	3		Cys119	3	
	Thr120	9		Thr120	8	
	Gly121	5		Gly121	5	
	Trp221	9		Trp221	9	
**P1′**	Gln23	4		Gln23	4	
	Cys29	3		Cys29	3	
	Val176	6		Val176	4	
	Leu181	1		Leu181	1	
	Met196	8		Met196	9	
	Gly197	6		Gly197	5	
	Gly198	2		Gly198	2	
	**His199**	8	**1**	**His199**	7	**1**
	**Trp221**	8	**1**	**Trp221**	8	**1**
**warhead**	**Gln23**	4	**1**	**Gln23**	4	**1**
	Gly27	5		Gly27	5	
	Ser28	3		Ser28	3	
	**Cys29**	7	**1**	**Cys29**	7	**1**
	His199	2		His199	2	

[a] The list of crystallographic contacts formed between human cathepsin B and inhibitors **7** and **31** was generated using the program CONTACT.[26] The distance cutoffs were set to 4.2 Å for all contacts and 3.3 Å for hydrogen bonds (H-bonds). The enzyme residues interacting in the individual inhibitor positions are specified (fragmentation of the inhibitors into the P1' and P2' positions and the warhead is shown). For each enzyme residue, total number of contacts is indicated including H-bonds (residues forming H-bonds are in bold).

Table S6. X-ray data collection and refinement statistics.

	Complex with **7**[a]	Complex with **31**[a]
**Data collection statistics**		
Wavelength (Å)	0.918	0.918
Temperature (K)	100	100
Space group	$P2_12_12_1$	$P2_12_12_1$
a, b, c (Å)	30.75 81.55 93.47	30.57 81.57 93.52
α, β, γ (°)	90.00 90.00 90.00	90.00 90.00 90.00
Resolution (Å)	50.00–1.45 (1.54–1.45)	50.00–1.70 (1.80–1.70)
Number of unique reflections	42360 (6631)	26540 (4187)
Multiplicity	13.0 (12.5)	12.9 (12.9)
Completeness (%)	98.8 (96.9)	99.9 (99.7)
R_{merge}[b] (%)	17.6 (312.0)	24.7 (316.0)
Average I/σ (I)	11.36 (0.78)	9.34 (0.83)
$CC_{1/2}$ (%)[c]	99.8 (39.7)	99.7 (33.0)
Wilson B (Å^2)	24.9	28.9
**Refinement statistics**		
Resolution range (Å)	46.73–1.45 (1.49–1.45)	46.76–1.70 (1.74–1.70)
Number of reflections in working set	40259 (2825)	25215 (1769)
Number of reflections in test set	2101 (148)	1328 (94)
R value[d] (%)	17.7 (38.7)	17.6 (35.2)
R_{free} value[e] (%)	20.8 (40.4)	20.5 (32.1)
Number of molecules in AU[f]	1	1
Number of atoms in AU[f] protein/inhibitor/solvent	2090/25/231	2013/25/190
Average ADP[g] for protein/inhibitor/solvent (Å^2)	20.8/26.7/31.2	24.5/28.8/32.9
RMSD bond length (Å)	0.013	0.011
RMSD bond angle (°)	1.80	1.67
Ramachandran plot statistics[h]		
Favored regions (%)	95.5	96.3
Allowed regions (%)	4.5	3.7
PDB code	8B4T	8B5F

[a] The numbers in the parentheses refer to the highest-resolution shell. [b] R_{merge}= $100\sum_{hkl}\sum_i|I_i(hkl) - \langle I(hkl)\rangle|/\sum_{hkl}\sum_i I_i(hkl)$, where $I_i(hkl)$ is an individual intensity of the ith observation of the reflection *hkl* and ‹I(*hkl*)› is the average intensity of the reflection *hkl* with summation over all data. [c] $CC_{1/2}$ (%) is the percentage of correlation between intensities from random half-datasets.[31] [d] R value = $||F_o| - |F_c||/|F_o|$, where F_o and F_c are the observed and calculated structure factors, respectively. [e] R_{free} is equivalent to the R value but is calculated for up to 5% of the reflections chosen at random and omitted from the refinement process.[32] [f] AU, asymmetric unit. [g] ADP, atomic displacement parameter, formally B-factor. [h] As determined by MolProbity.[25]

6. General Methods and Synthetic Procedures

General. Reagents and solvents were obtained from abcr (Karlsruhe, Germany), Acros (Geel, Belgium), Alfa Aesar (Karlsruhe, Germany), Bachem (Bubendorf, Switzerland), Calbiochem (Darmstadt, Germany), Carbolution Chemicals (Saarbrücken, Germany), Enzo Life Sciences (Lörrach, Germany), Fluka (Taufkirchen, Germany), Fluorochem (Hadfield, United Kingdom), IRIS Biotech (Marktredwitz, Germany), Merck (Darmstadt, Germany), Sigma-Aldrich (Steinheim, Germany) and TCI (Eschborn, Germany). Dichloromethane was dried according to a standard procedure with P_2O_5, all other dry solvents were purchased from Acros (Geel, Belgium) and used without further treatment.

Thin-layer chromatography (TLC) was carried out on Merck aluminum silica gel plates with 60 F_{254} indicator. Detection was performed with UV light at 254 nm or 366 nm. For visualization of the spots, Ninhydrin reagent (0.20 g in 100 mL EtOH) was used. Preparative column chromatography was performed using Merck silica gel 60 (63-200 mesh). Semi-preparative HPLC purifications were carried out on a system of the SMARTLINE series (Knauer, Germany) equipped with two S-1800 pumps with 100 mL pump heads including a two-canal online degasser as well as a static mixing chamber SmartMix 350, an assistant 6000 comprising an S-100 feed pump as well as an electrical injection valve with 2000 µL sample loop, an S-2550 UV detector and an electrical 16-port-1-canal switch valve for sample collection. Data were acquired and processed with ChromGate Software 3.3.2 Agilent Technologies. As the stationary phase, the Eurospher II 100-5 C18 (5 µm, column size 250 × 16 mm, Knauer, Germany) connected to a precolumn (30 × 16 mm), was applied. The preparation of the sample solution (mg/mL) and the composition of the mobile phase throughout the purification including the duration time (min) are as indicated for each compound. Each run was performed at room temperature after injection of a definite volume (µL) of the unfiltrated sample solution at a specific flow rate (mL/min), and detection was carried out at a particular wavelength (nm). Melting points were determined on a Büchi 510 oil bath apparatus and were uncorrected.

^{1}H NMR (600 MHz or 500 MHz) and ^{13}C NMR (150 MHz or 125 MHz) spectra were recorded on a Bruker Avance III-600 MHz or Bruker Avance DRX-500 MHz instrument at 30 °C using DMSO-d_6 or at 25 °C using $CDCl_3$ as solvent. Chemical shifts are reported in ppm relative to the remaining protons of the deuterated solvent used as an internal standard (DMSO-d_6: 2.49 / 39.7 ppm; $CDCl_3$: 7.24 / 77.0 ppm). Coupling constants (J) are given in Hertz and spin multiplicities are given as s (singlet), d (doublet), t (triplet), q (quartet), quint (quintet) and m (multiplet).

HPLC (diode array detection; DAD) chromatograms and MS (electrospray ionization; ESI) spectra were recorded on an API 2000 mass spectrometer (AB Sciex, Darmstadt, Germany) coupled with an Agilent HP1100 HPLC system using an EC50/2 Nucleodur C18 Gravity 3 µm column (Macherey-Nagel, Düren, Germany). The purity of the tested compounds was determined monitoring the UV absorption from 220 nm to 400 nm on the described system using a procedure as follows: samples were dissolved (1 mg/mL) in MeOH or MeCN, respectively, containing 2mM $NH_4^+CH_3COO^-$. Then, 8 µL of the sample solution was injected into the column at 25 °C. The mobile phase was a mixture of H_2O containing

2 mM $NH_4^+CH_3COO^-$ (A) and MeOH or MeCN, respectively, containing 2 mM $NH_4^+CH_3COO^-$ (B). Elution was performed following a gradient of A/B (90:10) to (0:100) in 10 min, then (0:100) to 20 min at a flow rate of 300 µL/min.

Quadrupole time-of-flight high-resolution mass spectrometry using electrospray ionization (ESI-Q/TOF-HRMS) spectra were recorded on a microTOF-Q mass spectrometer (Bruker, Köln, Germany) coupled with an HPLC Dionex Ultimate 3000 (Thermo Scientific, Braunschweig, Germany) using an EC50/2 Nucleodur C18 Gravity 3 µm column (Macherey-Nagel, Düren, Germany). Samples were dissolved (0.6 mg/ml) in MeCN. Then, 1 µL of the sample solution was injected into the column at 25 °C. The mobile phase was a mixture of H_2O containing 2 mM $NH_4^+CH_3COO^-$ (A) and MeCN (B). Elution was performed following a gradient of A/B (90:10) for 1 min, then (90:10) to (0:100) in 9 min and (0:100) to 20 min at a flow rate of 300 µL/min.

General procedure for mixed anhydride-mediated dipeptide formation (GP1). Cbz-Leu-OH (2.65 g, 10.0 mmol) was dissolved in dry CH_2Cl_2 (100 mL) and cooled to -25 °C. *N*-Methylmorpholine (11.0–22.0 mmol) and isobutyl chloroformate (1.50 g, 11.0 mmol) were added and the reaction mixture was stirred for 45 min. Upon precipitation of *N*-methylmorpholine hydrochloride, an amine / ammonium chloride *tert*-butyl ester (10.0 mmol) was added. The mixture was stirred and allowed to warm to room temperature (rt) within 16 h. The reaction mixture was washed with 10% $KHSO_4$ (2 × 50 mL) and sat. aq. $NaHCO_3$ (2 × 50 mL). The solvent was dried over Na_2SO_4 and evaporated. The crude product was purified by silica gel column chromatography using petroleum ether / EtOAc (3:1) as eluent.

General procedure for HATU-mediated dipeptide formation (GP2). Cbz-Ala-OH (0.44 g, 2.00 mmol) was dissolved in dry CH_2Cl_2 (30 mL). Subsequently, an amine / ammonium chloride *tert*-butyl ester (2.00 mmol), 1-[bis(dimethylamino)methylene]-1*H*-1,2,3-triazolo[4,5-*b*]pyridinium 3-oxide hexafluorophosphate (HATU; 0.76 g, 2.00 mmol) and *N*,*N*-diisopropylethylamine (DIPEA; 4.00–6.00 mmol) were added and the solution was stirred at rt for 16 h. After evaporation of the solvent, the crude product was dissolved in EtOAc (30 mL) and washed with 10% $KHSO_4$ (2 × 30 mL), sat. aq. $NaHCO_3$ (2 × 30 mL) and brine (30 mL). The solvent was dried over Na_2SO_4 and evaporated. Purification of the compounds was carried out as noted.

General procedure for EDC-mediated dipeptide formation (GP3). Cbz-Cha-OH (1.00 g, 3.00 mmol) was dissolved in dry CH_2Cl_2 (25 mL). 1-Ethyl-3-(3-dimethylaminopropyl)carbodiimide (EDC; 0.56 g, 3.60 mmol) and 4-dimethylaminopyridine (DMAP; 18 mg, 0.15 mmol) were added and the reaction mixture was stirred at rt for 5 min. Upon addition of an amine / ammonium chloride *tert*-butyl ester (3.00 mmol) and DIPEA (0.78 g, 6.00 mmol), the mixture was stirred at rt for 16 h. After evaporation of the solvent, the residue was dispersed between in H_2O (60 mL) and EtOAc (3 × 30 mL). The combined organic layers were washed with 10% $KHSO_4$ (2 × 30 mL), sat. aq. $NaHCO_3$ (2 × 30 mL) and brine (30 mL). The solvent was dried over Na_2SO_4 and evaporated. The crude product was purified by column chromatography on silica gel using petroleum ether / EtOAc (1:1) as eluent.

General procedure for HATU-mediated dipeptide methyl ester formation (GP4). Boc-Leu-OH × H_2O (0.50 g, 2.00 mmol) was dissolved in dry CH_2Cl_2 (30 mL). H-Pro-OMe × HCl (0.33 g, 2.00 mmol) or H-Phe-OMe × HCl (0.43 g, 2.00 mmol), HATU (0.76 g, 2.00 mmol) and DIPEA (0.76 g, 6.00 mmol) were added. The reaction mixture was stirred at rt for 16 h. The solvent was evaporated and the residue was redissolved in EtOAc (30 mL). The organic layer was washed with sat. aq. $NaHCO_3$ (2 × 30 mL), water (30 mL), 10% $KHSO_4$ (2 × 30 mL) and brine (30 mL) and dried over Na_2SO_4. After evaporation of the solvent, purification of the residue was carried out as noted.

General procedure for TFA-mediated *N*-Boc deprotection (GP5). The Boc-protected dipeptide methyl ester (1.00 mmol) was dissolved in dry CH_2Cl_2 (30 mL) and trifluoroacetic acid (TFA; 6 mL) was added. The reaction mixture was stirred at rt for 2 h. The solvent was evaporated at < 1 mbar. The product was characterized without purification.

General procedure for *O*-aryl-*N*-dipeptidyl carbamate methyl ester formation (GP6). The dipeptidic ammonium trifluoroacetate (0.50 mmol) was dissolved in dry THF (5 mL). 4-chlorophenyl chloroformate (0.11 g, 0.55 mmol) and triethylamine (TEA; 0.17 g, 1.65 mmol) were added. After stirring at rt for 2 h, the solvent was evaporated. The residue was dispersed between water (30 mL) and EtOAc (30 mL). The aqueous layer was extracted with EtOAc (2 × 30 mL). The combined organic layers were washed with brine (2 × 30 ml) and dried over Na_2SO_4. After evaporation of the solvent, the crude product was purified by column chromatography on silica gel using CH_2Cl_2 / EtOAc (9.5:0.5) as eluent.

General procedure for *O*-aryl-*N*-dipeptidyl carbamate formation (GP7). The approproiate Cbz-protected dipeptide *tert*-butyl ester (2.00 mmol) was dissolved in dry MeOH (15 mL), treated with Pd/C (10 wt%) and hydrogenated for 1 h at 30 psi and rt. After filtration over celite and evaporation of methanol, the obtained amine (0.60–2.00 mmol) was redissolved in THF (20–30mL) and TEA (0.60–2.00 mmol). Upon addition of 4-chlorophenyl chloroformate or phenyl chloroformate (0.66–2.20 mmol), the reaction mixture was stirred at rt for 2 h. Subsequently, the solvent was evaporated and the residue was suspended in H_2O (30 mL) and extracted with EtOAc (3 × 30 mL). The organic layers were combined and washed with brine (30 mL). After drying of the organic layer over Na_2SO_4 and evaporation of the solvent, the crude product was purified by column chromatography on silica gel using mixtures of petroleum ether / EtOAc as noted.

General procedure for *O*-aryl-*N*-dipeptidyl carbamate *tert*-butyl ester cleavage (GP8). The carbamate *tert*-butyl ester (0.10–0.30 mmol) was dissolved in dry CH_2Cl_2 (10 mL) and treated with TFA (2 mL). After stirring for 2 h at rt, the solvent was evaporated. In need of a subsequent purification of the product, the residue was subjected to column chromatography on silica gel using EtOAc or mixtures of petroleum ether / EtOAc as noted plus 1% AcOH as modifier.

General procedure for solid phase carbamate formation (GP9). The respective Fmoc-protected Wang resin-bound amino acid (0.50 mmol) was suspended in piperidine (2 mL) and DMF (8 mL). Shaking was carried out continuously at 300 rpm. After 30 min, the resin was washed in a standard

procedure with DMF (3 × 10 mL), CH_2Cl_2 (3 × 10 mL), MeOH (3 × 10 mL), again CH_2Cl_2 (3 × 10 mL) and finally dried. The deprotected Wang resin-bound amino acid or a 2-chlorotrityl resin-bound amino acid (0.50 mmol), respectively, was suspended in DMF (5 mL) and DIPEA (0.26 g, 2.00 mmol). The respective Fmoc-protected amino acid (1.00 mmol), [benzotriazol-1-yloxy(dimethylamino)methylidene]-dimethylazanium hexafluorophosphate (HBTU; 0.38 g, 1.00 mmol) and 1*H*-1,2,3-benzotriazol-1-ol (HOBt; 0.15 g, 1.00 mmol) were suspended in DMF (5 mL), treated for 2 min in an ultrasonic bath and added to the resin-bound amino acid. After shaking for 2 h, the resin was washed and dried. The Fmoc-protected resin-bound dipeptide was then suspended in DMF (8 mL) and piperidine (2 mL) and shaking was continued for 30 min. The solvent was filtered off and Fmoc-deprotection conditions were repeated once. The resin was washed and dried. The *N*-deprotected resin-bound dipeptide was suspended in CH_2Cl_2 (8 mL) and DIPEA (0.13 g, 1.00 mmol). After 5 min, 4-chlorophenyl chloroformate (0.19 g, 1.00 mmol) was added and the reaction mixture was shaken for 2 h. The solvent was filtered off and the resin was washed and dried. The resin-bound dipeptidic carbamate was shaken for 1 h in a mixture of TFA, triisopropylsilane (TIPS) and CH_2Cl_2 (10 mL, [0.2:0.2:9.6] for 2-chlorotrityl resin or [9.5:0.25:0.25] for Wang resin). The product-containing solution was filtered off and collected and the cleavage conditions were applied to the resin once again. After evaporation of the solvent, the residue was purified. Unless stated otherwise, column chromatography on silica gel was performed using eluents as noted plus 1% AcOH as a modifier.

General procedure for mixed anhydride-mediated preparation of amidophenols (GP10). The respective carboxylic acid (2.20–8.80 mmol) was dissolved in dry CH_2Cl_2 (25–40 mL) and cooled to -25 °C. Subsequently, *N*-methylmorpholine (2.20–8.80 mmol) and isobutyl chloroformate (2.20–8.80 mmol) were added. The solution was stirred for 30 min. The respective 3-amino-4-halophenol (2.00–8.00 mmol) was applied as a solid or a solution of 2-chloro-4-hydroxyanilinium chloride (0.72 g, 4.00 mmol) and *N*-methylmorpholine (0.40 g, 4.00 mmol) in CH_2Cl_2 (40 mL) was added, respectively. The reaction mixture was stirred for 4 h whilst warming to rt. Upon evaporation of CH_2Cl_2, the residue was dissolved in EtOAc (100 mL) and washed with sat. aq. $NaHCO_3$ (50 mL), 10% $KHSO_4$ (50 mL) and brine (50 mL). The organic layer was dried over Na_2SO_4 and evaporated. The crude product was purified by recrystallization or column chromatography on silica gel using petroleum ether / EtOAc (2:1) as eluent.

General procedure for uronium salt-mediated preparation of amidoanisoles and amidophenols (GP11). The respective carboxylic acid (3.00–5.00 mmol) was dissolved in dry THF (40 mL). Subsequently, HBTU (3.00–5.00 mmol), DIPEA (4.50–10.0 mmol) and the corresponding halogenated aminophenol / hydroxyanilinium chloride or anisidine (3.00–5.00 mmol), respectively, were added and the solution was stirred at rt for 16 h. After evaporation of THF, the crude product was dissolved in EtOAc (40 mL) and washed with 10% $KHSO_4$ (3 × 40 mL) and brine (40 ml). The solvent was dried over Na_2SO_4 and evaporated. Purification of the compounds was carried out by recrystallization or column chromatography on silica gel as noted.

General procedure for the cleavage of aromatic methoxy groups (GP12). The anisole (1.00–2.00 mmol) was dissolved in dry CH_2Cl_2 (30–50 mL) and cooled to -20 °C. Upon dropwise addition of BBr_3 (3.00–6.00 mmol), the reaction mixture was stirred for 16 h while warming to rt. Quenching of remaining BBr_3 was achieved by addition of ice water (50 mL). After addition of CH_2Cl_2 (50 mL), the organic layer was washed with sat. aq. $NaHCO_3$ (2 × 50 mL), dried over Na_2SO_4 and evaporated. The crude product was purified by column chromatography on silica gel using mixtures of petroleum ether / EtOAc as noted.

General procedure for *O*-aryl-*N*-dipeptidyl carbamate formation (GP13). Triphosgene (0.15 g, 0.50 mmol) was dissolved in dry THF (15 mL) under argon atmosphere and cooled to 0 °C. DIPEA (0.19 g, 1.5 mmol) was added to release phosgene (3 eq.) from the triphosgene. Immediately, an appropriate phenol (0.50 mmol), dissolved in THF (15 mL), was added to the solution dropwise over 30 min generating a phenylchloroformate intermediate, which was not isolated. The reaction mixture was stirred for 1 h at 0 °C. Hydrogenolysis of a Cbz-protected dipeptide *tert*-butyl ester (0.75 mmol) using Pd/C (10 wt%) and H_2 at 30 psi for 1 h at rt in dry MeOH (20 mL) afforded the desired dipeptidic amine component for the final reaction step. Upon filtration over celite and evaporation of methanol, the amine was redissolved in THF (15 mL) and DIPEA (0.19 g, 1.5 mmol) and added to the reaction mixture containing the phenylchloroformate. Whilst stirring for 1 h and warming up to rt, the desired carbamate was generated. Subsequently, THF was evaporated and the residue was dissolved in CH_2Cl_2 (50 mL) and washed whit 10% $KHSO_4$ (2 × 50 mL). After drying of the organic layer over Na_2SO_4 and evaporation of the solvent, the crude product was purified by column chromatography on silica gel as noted.

7. Preparation of Compounds

7.1. Dipeptide and amino acid precursors

Cbz-leucyl-phenylalanine *tert*-butyl ester

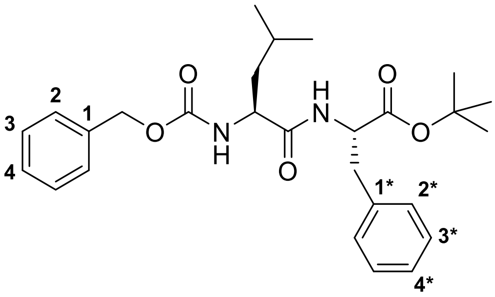

The synthesis was carried out according to GP1 using H-Phe-O*t*Bu × HCl (2.58 g) and *N*-methylmorpholine (2.23 g, 22.0 mmol). Column chromatography on silica gel afforded Cbz-Leu-Phe-O*t*Bu[33] as a white solid (3.31 g, 7.06 mmol, 71%). mp 123–125 °C; ^{1}H NMR (500 MHz, DMSO-d_6) δ 0.84 (2 × d, ^3J = 6.6 Hz, 6H, CH(C<u>H</u>$_3$)$_{2,Leu}$), 1.30 (s, 9H, C(C<u>H</u>$_3$)$_3$), 1.34–1.43 (m, 2H, CHC<u>H</u>$_{2,Leu}$), 1.53–1.62 (m, 1H, C<u>H</u>(CH$_3$)$_{2,Leu}$), 2.88–3.00 (m, 2H, CHC<u>H</u>$_{2,Phe}$), 4.02–4.09 (m, 1H, NHC<u>H</u>$_{Phe}$), 4.34 (q, ^3J = 8.9 Hz, 1H, NHC<u>H</u>$_{Leu}$), 5.01 (s, 2H, PhC<u>H</u>$_2$O), 7.16–7.36 (m, 11H, 2-<u>H</u>, 3-<u>H</u>, 4-<u>H</u>, 2*-<u>H</u>, 3*-<u>H</u>, 4*-<u>H</u>, OCON<u>H</u>), 8.12 (d, ^3J = 7.5 Hz, 1H, N<u>H</u>CH$_{Phe}$); ^{13}C NMR (125 MHz, DMSO-d_6) δ 21.61, 23.07, 24.25 (<u>C</u>H(<u>C</u>H$_3$)$_{2,Leu}$), 27.62 (C(<u>C</u>H$_3$)$_3$), 36.85 (CH<u>C</u>H$_{2,Phe}$), 40.93 (CH<u>C</u>H$_{2,Leu}$), 53.02, 54.14 (NH<u>C</u>H$_{Phe}$, NH<u>C</u>H$_{Leu}$), 65.45 (Ph<u>C</u>H$_2$O), 80.69 (<u>C</u>(CH$_3$)$_3$), 126.54 (<u>C</u>-4*), 127.87 (<u>C</u>-4), 127.74, 128.21, 128.42, 129.31 (<u>C</u>-2, <u>C</u>-3, <u>C</u>-2*, <u>C</u>-3*), 137.19, 137.31 (<u>C</u>-1, <u>C</u>-1*), 155.91 (O<u>C</u>ONH), 170.45, 172.32 (CH<u>C</u>O); LC/MS (ESI): H_2O/MeOH, 90:10 to 0:100; *m/z* 469.3 [M + H]$^+$; 97% purity; $C_{27}H_{36}N_2O_5$.

Boc-leucyl-phenylalanine methyl ester

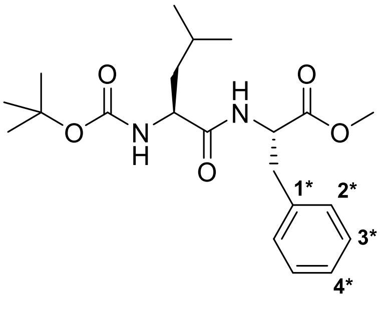

The compound was synthesized according to GP4. The crude product was purified by recrystallization from EtOAc to yield Boc-Leu-Phe-OMe as a white solid (0.40 g, 51%). mp 68–70 °C; ^{1}H NMR (500 MHz, DMSO-d_6) δ 0.82 (2 × d, ^3J = 6.6 Hz, 6H, CH(C<u>H</u>$_3$)$_2$), 1.21–1.41 (m, 11H, C(C<u>H</u>$_3$)$_3$, CHC<u>H</u>$_{2,Leu}$), 1.47–1.55 (m, 1H, C<u>H</u>(CH$_3$)$_{2,Leu}$), 2.93 (dd, ^2J = 13.9 Hz, ^3J = 8.7 Hz, 1H, CHC<u>H</u>$_{2,Phe}$), 3.02 (dd, ^2J = 13.9 Hz, ^3J = 5.7 Hz, 1H, CHC<u>H</u>$_{2,Phe}$), 3.57 (s, 3H, OC<u>H</u>$_3$), 3.95 (dt, ^3J = 5.7 Hz, ^3J = 9.1 Hz, 1H, NHC<u>H</u>$_{Leu}$), 4.48 (dt, ^3J = 5.7 Hz, ^3J = 8.3 Hz, 1H, NHC<u>H</u>$_{Phe}$), 6.75 (d, ^3J = 8.6 Hz, 1H, OCON<u>H</u>), 7.16–7.21 (m, 3H, 2*-<u>H</u>, 4*-<u>H</u>), 7.22–7.28 (m, 2H, 3*-<u>H</u>), 8.07 (d, ^3J = 7.7 Hz, 1H, N<u>H</u>CH$_{Phe}$); ^{13}C NMR (125 MHz, DMSO-d_6) δ 21.75, 22.94, 24.25 (<u>C</u>H(<u>C</u>H$_3$)$_2$), 28.29 (C(<u>C</u>H$_3$)$_3$), 36.76 (CH<u>C</u>H$_{2,Phe}$), 40.93

($CH\underline{C}H_{2,Leu}$), 51.89 ($O\underline{C}H_3$), 52.79, 53.35 ($NH\underline{C}H_{Leu}$, $NH\underline{C}H_{Phe}$), 78.11 ($\underline{C}(CH_3)_3$), 126.59 ($\underline{C}$-4*), 128.28 ($\underline{C}$-2*), 129.18 ($\underline{C}$-3*), 137.16 ($\underline{C}$-1*), 155.22 ($O\underline{C}ONH$), 171.92, 172.62 ($CH\underline{C}O$); LC/MS (ESI): H_2O/MeOH, 90:10 to 0:100; *m/z* 393.3 $[M + H]^+$, 410.5 $[M + NH_4]^+$; 100% purity; $C_{21}H_{32}N_2O_5$.

Leucyl-phenylalanine methylester trifluoroacetate (32)

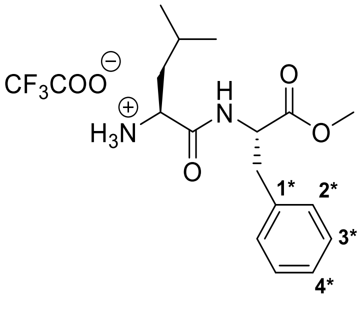

The compound was synthesized according to GP5 using Boc-Leu-Phe-OMe (0.39 g) to obtain TFA × H-Leu-Phe-OMe a white solid (0.39 g, 96%). mp 162–164 °C; ^{1}H NMR (500 MHz, DMSO-d_6) δ 0.88 (2 × d, ^3J = 6.2 Hz, 6H, $CH(C\underline{H}_3)_2$), 1.47–1.57 (m, 2H, $CHC\underline{H}_{2,Leu}$), 1.61–1.68 (m, 1H, $C\underline{H}(CH_3)_{2,Leu}$), 2.98 (dd, ^2J = 14.1 Hz, ^3J = 8.6 Hz, 1H, $CHC\underline{H}_{2,Phe}$), 3.07 (dd, ^2J = 14.0 Hz, ^3J = 5.9 Hz, 1H, $CHC\underline{H}_{2,Phe}$), 3.59 (s, 3H, $OC\underline{H}_3$), 3.73–3.79 (m, 1H, $NH_3C\underline{H}_{Leu}$), 4.51–4.58 (m, 1H, $NHC\underline{H}_{Phe}$), 7.20–7.26 (m, 3H, 2*-$\underline{H}$, 4*-$\underline{H}$), 7.26–7.33 (m, 2H, 3*-$\underline{H}$), 8.10 (d, ^3J = 5.2 Hz, 3H, $N\underline{H}_3$), 8.91 (d, ^3J = 7.4 Hz, 1H, $N\underline{H}CH_{Phe}$); ^{13}C NMR (125 MHz, DMSO-d_6) δ 21.83, 22.86, 23.49 ($\underline{C}H(\underline{C}H_3)_2$), 36.48 ($CH\underline{C}H_{2,Phe}$), 50.76 ($O\underline{C}H_3$), 52.10, 53.99 ($NH\underline{C}H_{Leu}$, $NH\underline{C}H_{Phe}$), 126.83 ($\underline{C}$-4*), 128.48 ($\underline{C}$-2*), 129.13 ($\underline{C}$-3*), 136.94 ($\underline{C}$-1*), 169.43 ($CH\underline{C}O$), 171.38 ($\underline{C}OOCH_3$); one signal ($CH\underline{C}H_{2,Leu}$) is obscured by the solvent peak; LC/MS (ESI): H_2O/MeOH, 90:10 to 0:100; *m/z* 293.0 $[M + H]^+$; 97% purity; $C_{16}H_{24}N_2O_3 \cdot C_2HF_3O_2$.

Cbz-leucyl-proline *tert*-butyl ester

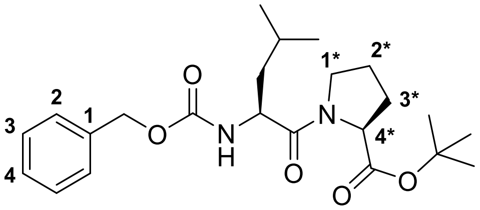

The synthesis was carried out according to GP1 using H-Pro-O*t*Bu (1.71 g) and *N*-methylmorpholine (1.11 g, 11.0 mmol). Column chromatography on silica gel afforded Cbz-Leu-Pro-O*t*Bu as a colorless oil (3.06 g, 7.30 mmol, 73%). ^{1}H NMR (600 MHz, DMSO-d_6) δ 0.88 (2 × d, ^3J = 6.7 Hz, 6H, $CH(C\underline{H}_3)_{2,Leu}$), 1.21–1.31 (m, 1H, $CHC\underline{H}_{2,Leu}$), 1.36 (s, 9H, $C(C\underline{H}_3)_3$), 1.45–1.52 (m, 1H, $CHC\underline{H}_{2,Leu}$), 1.63–1.71 (m, 1H, $C\underline{H}(CH_3)_{2,Leu}$), 1.73–1.80 (m, 1H, 2*-$\underline{H}$), 1.88–1.95 (m, 2H, 2*-$\underline{H}$, 3*-$\underline{H}$), 2.10–2.18 (m, 1H, 3*-$\underline{H}$), 3.47 (dt, ^2J = 9.4 Hz, ^3J = 6.8 Hz, 1H, 1*-$\underline{H}$), 3.67 (dt, ^2J = 9.5 Hz, ^3J = 6.9 Hz, 1H, 1*-$\underline{H}$), 4.17 (dd, ^3J = 4.9 Hz, ^3J = 8.5 Hz, 1H, 4*-$\underline{H}$), 4.23–4.30 (m, 1H, $NHC\underline{H}_{Leu}$), 4.99 (s, 2H, $PhC\underline{H}_2O$), 7.30–7.37 (m, 5H, 2-$\underline{H}$, 3-$\underline{H}$, 4-$\underline{H}$), 7.49 (d, ^3J = 8.1 Hz, 1H, $OCON\underline{H}$); ^{13}C NMR (150 MHz, DMSO) δ 21.42, 23.25, 24.27 ($\underline{C}H(\underline{C}H_3)_{2,Leu}$), 24.74 ($\underline{C}$-2*), 27.70 ($C(\underline{C}H_3)_3$), 28.66 ($\underline{C}$-3*), 40.23 ($CH\underline{C}H_{2,Leu}$),

46.45 (C-1*), 50.86 (NHCH$_{Leu}$), 59.55 (C-4*), 65.50 (PhCH$_2$O), 80.37 (C(CH$_3$)$_3$), 127.82 (C-2), 127.92 (C-4), 128.47 (C-3), 137.21 (C-1), 156.19 (OCONH), 170.79, 171.05 (CHCO); LC/MS (ESI): H_2O/MeOH, 90:10 to 0:100; *m/z* 419.4 [M + H]$^+$; 100% purity; $C_{23}H_{34}N_2O_5$.

Boc-leucyl-proline methyl ester

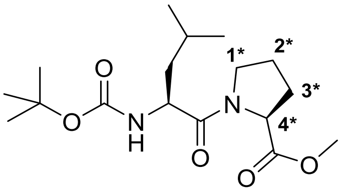

The compound was synthesized according to GP4. The product was purified by column chromatography on silica gel using petroleum ether / EtOAc (7:3) as eluent to give Boc-Leu-Pro-OMe as a colorless oil (0.46 g, 67%). ^{1}H NMR (600 MHz, CHCl$_3$) δ 0.95 (2 × d, ^3J = 6.6 Hz, 6H, CH(CH$_3$)$_2$), 1.39 (s, 9H, C(CH$_3$)$_3$), 1.45–1.49 (m, 2H, CHCH$_{2,Leu}$), 1.71–1.78 (m, 1H, CH(CH$_3$)$_{2,Leu}$), 1.94–2.04 (m, 3H, 2*-H, 3*-H), 2.16–2.22 (m, 1H, 3*-H), 3.54–3.62 (m, 1H, 1*-H), 3.69 (s, 3H, OCH$_3$), 3.72–3.78 (m, 1H, 1*-H), 4.45 (dt, ^3J = 5.6 Hz, ^3J = 8.8 Hz, 1H, NHCH$_{Leu}$), 4.50 (dd, ^3J = 4.6 Hz, ^3J = 8.4 Hz, 1H, 4*-H), 5.09 (d, ^3J = 9.2 Hz, 1H, NH); ^{13}C NMR (150 MHz, CDCl$_3$) δ 21.78, 23.36 (CH(CH$_3$)$_2$), 24.53, 24.88 (C-2*, CH(CH$_3$)$_2$), 28.32 (C(CH$_3$)$_3$), 28.94 (C-3*), 41.92 (CHCH$_{2,Leu}$), 46.70 (C-1*), 50.27 (NHCH$_{Leu}$), 52.19 (COOCH$_3$), 58.66 (C-4*), 79.51 (C(CH$_3$)$_3$), 155.70 (OCONH), 171.87 (CON), 172.51 (COOCH$_3$); LC/MS (ESI): H_2O/MeOH, 90:10 to 0:100; *m/z* 343.0 [M + H]$^+$; 100% purity; $C_{17}H_{30}N_2O_5$.

Leucyl-proline methylester trifluoroacetate (33)

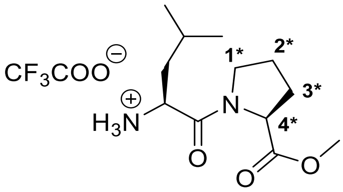

The compound was synthesized according to GP5 using Boc-Leu-Pro-OMe (0.34 g) to obtain TFA × H-Leu-Pro-OMe as a yellowish oil (0.33 g, 92%). ^{1}H NMR (600 MHz, DMSO-d_6) δ 0.93 (2 × d, ^3J = 6.5 Hz, 6H, CH(CH$_3$)$_2$), 1.48–1.60 (m, 2H, CH$_2$CH(CH$_3$)$_2$, 1.73–1.80 (m, 1H, CH(CH$_3$)$_2$), 1.81–1.87 (m, 1H, 2*-H), 1.88–1.92 (m, 1H, 2*-H), 1.93–2.00 (m, 1H, 3*-H), 2.18–2.27 (m, 1H, 3*-H), 3.42 (dt, ^2J = 9.9 Hz, ^3J = 7.2 Hz, 1H, 1*-H), 3.61 (s, 3H, OCH$_3$), 3.73–3.78 (m, 1H, 1*-H), 4.38 (dd, ^3J = 6.0, ^3J = 8.4 Hz, 1H, 4*-H), 8.15 (d, ^3J = 6.1 Hz, 3H, NH$_3$); NH$_3$CH$_{Leu}$ is obscured; ^{13}C NMR (150 MHz, DMSO-d_6) δ 21.47, 23.16, 23.54 (CH(CH$_3$)$_2$), 24.94 (C-2*), 28.71 (C-3*), 46.77 (C-1*), 49.60 (NH$_3$CH$_{Leu}$), 52.13 (COOCH$_3$), 58.90 (C-4*), 168.08 (CON), 171.90 (COOCH$_3$); one signal (CHCH$_{2,Leu}$) is obscured by the solvent peak; LC/MS (ESI): H_2O/MeOH, 90:10 to 0:100; *m/z* 243.3 [M + H]$^+$; 93% purity; $C_{12}H_{22}N_2O_3 \cdot C_2HF_3O_2$.

Cbz-cyclohexylalanyl-phenylalanine *tert*-butyl ester

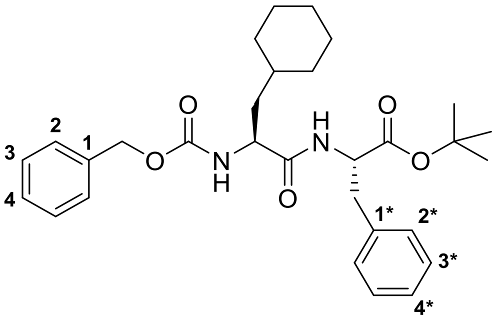

The synthesis was carried out according to GP3 using H-Phe-O*t*Bu × HCl (0.77 g). Column chromatography on silica gel afforded Cbz-Cha-Phe-O*t*Bu as a white solid (0.99 g, 1.95 mmol, 65%). mp 88–91 °C; ^{1}H NMR (500 MHz, DMSO-*d*$_6$) δ 0.78–0.88 (m, 2H, CH(C<u>H</u>$_2$)$_5$), 1.03–1.20 (m, 4H, C<u>H</u>(C<u>H</u>$_2$)$_5$), 1.30 (s, 9H, C(C<u>H</u>$_3$)$_3$), 1.36–1.42 (m, 2H, CH(C<u>H</u>$_2$)$_5$), 1.60–1.69 (m, 5H, NHCHC<u>H</u>$_{2,Cha}$, CH(C<u>H</u>$_2$)$_5$), 2.89–2.98 (m, 2H, CHC<u>H</u>$_{2,Phe}$), 4.08 (dt, ^3J = 6.1 Hz, ^3J = 9.0 Hz, 1H, NHC<u>H</u>$_{Phe}$), 4.33 (q, ^3J = 7.4 Hz, 1H, NHC<u>H</u>$_{Cha}$), 4.97–5.05 (m, 2H, PhC<u>H</u>$_2$O), 7.16–7.22 (m, 3H, 2*-<u>H</u>, 4*-<u>H</u>), 7.23–7.27 (m, 2H, 3*-<u>H</u>), 7.28–7.35 (m, 6H, 2-<u>H</u>, 3-<u>H</u>, 4-<u>H</u>, OCON<u>H</u>), 8.09 (d, ^3J = 7.6 Hz, 1H, N<u>H</u>CH$_{Phe}$); ^{13}C NMR (125 MHz, DMSO-*d*$_6$) δ 25.72, 25.91, 26.21 (CH(<u>C</u>H$_2$)$_5$), 27.65 (C(<u>C</u>H$_3$)$_3$), 31.87, 33.34, 33.57 (<u>C</u>H(<u>C</u>H$_2$)$_5$), 36.89 (CH<u>C</u>H$_{2,Phe}$), 52.39 (NH<u>C</u>H$_{Phe}$), 54.15 (NH<u>C</u>H$_{Cha}$), 65.42 (Ph<u>C</u>H$_2$O), 80.72 (<u>C</u>(CH$_3$)$_3$), 126.56 (<u>C</u>-4*), 127.87 (<u>C</u>-4), 127.69, 128.23, 128.44, 129.33 (<u>C</u>-2, <u>C</u>-3, <u>C</u>-2*, <u>C</u>-3*), 137.28, 137.32 (<u>C</u>-1, <u>C</u>-1*), 155.93 (O<u>C</u>ONH), 170.49, 172.45 (CH<u>C</u>O). One signal (NHCH<u>C</u>H$_{2,Cha}$) is obscured by the DMSO solvent signal; LC/MS (ESI): H_2O/MeOH, 90:10 to 0:100; *m/z* 509.1 [M + H]$^+$; 100% purity; $C_{30}H_{40}N_2O_5$.

Cbz-alanyl-proline *tert*-butyl ester

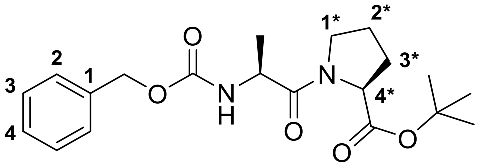

The synthesis was carried out according to GP2 using H-Pro-O*t*Bu (0.34 g) and DIPEA (0.52 g, 4.00 mmol). Column chromatography on silica gel using petroleum ether / EtOAc (3:7) as eluent afforded Cbz-Ala-Pro-O*t*Bu[34] as a colorless oil (0.55 g, 1.46 mmol, 73%). ^{1}H NMR (500 MHz, DMSO-*d*$_6$) δ 1.19 (d, ^3J = 7.3 Hz, 3H, CHC<u>H</u>$_{3,Ala}$), 1.36 (s, 9H, C(C<u>H</u>$_3$)$_3$), 1.74–1.80 (m, 1H, 2*-<u>H</u>), 1.87–1.93 (m, 2H, 2*-<u>H</u>, 3*-<u>H</u>), 2.10–2.17 (m, 1H, 3*-<u>H</u>), 3.50 (dt, ^2J = 9.8 Hz, ^3J = 6.8 Hz, 1H, 1*-<u>H</u>), 3.63 (dt, ^2J = 9.8 Hz, ^3J = 6.8 Hz, 1H, 1*-<u>H</u>), 4.17 (dd, ^3J = 4.7 Hz, ^3J = 8.5 Hz, 1H, 4*-<u>H</u>), 4.30 (quint, ^3J = 7.1 Hz, 1H, NHC<u>H</u>$_{Ala}$), 4.97 (d, ^2J = 12.6 Hz, 1H, PhC<u>H</u>$_2$O), 5.01 (d, ^2J = 12.6 Hz, 1H, PhC<u>H</u>$_2$O), 7.29–7.37 (m, 5H, 2-<u>H</u>, 3-<u>H</u>, 4-<u>H</u>), 7.45 (d, ^3J = 7.3 Hz, 1H, OCON<u>H</u>); ^{13}C NMR (125 MHz, DMSO-*d*$_6$) δ 16.80 (CH<u>C</u>H$_{3,Ala}$), 24.71 (<u>C</u>-2*), 27.72 (C(<u>C</u>H$_3$)$_3$), 28.63 (<u>C</u>-3*), 46.41 (<u>C</u>-1*), 48.03 (NH<u>C</u>H$_{Ala}$), 59.49 (<u>C</u>-4*), 65.45 (Ph<u>C</u>H$_2$O), 80.34 (<u>C</u>(CH$_3$)$_3$), 127.90 (<u>C</u>-4), 127.81, 128.46 (<u>C</u>-2, <u>C</u>-3), 137.21 (<u>C</u>-1), 155.77

(OCONH), 170.94, 171.03 (CHCO); LC/MS (ESI): H_2O/MeOH, 90:10 to 0:100; *m/z* 377.3 $[M + H]^+$; 99% purity; $C_{20}H_{28}N_2O_5$.

Cbz-alanyl-phenylalanine *tert*-butyl ester

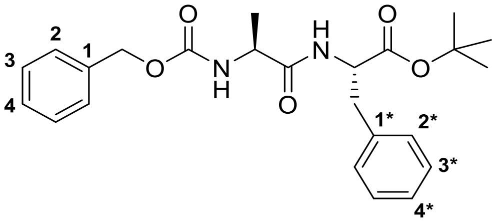

The synthesis was carried out according to GP2 using H-Phe-O*t*Bu × HCl (0.52 g) and DIPEA (0.78 g, 6.00 mmol). Column chromatography on silica gel using petrolum ether / EtOAc (1:1) as eluent afforded Cbz-Ala-Phe-O*t*Bu[35] as a white solid (0.76 g, 1.78 mmol, 89%). mp 118–120 °C; ^{1}H NMR (500 MHz, DMSO-*d*$_6$) δ 1.17 (d, ^3J = 7.3 Hz, 3H, $CHCH_{3,Ala}$), 1.30 (s, 9H, $C(CH_3)_3$), 2.89–2.98 (m, 2H, $CHCH_{2,Phe}$), 4.07 (quint, ^3J = 7.3 Hz, 1H, $NHCH_{Ala}$), 4.33 (q, 1H, ^3J = 7.3 Hz, $NHCH_{Phe}$), 5.00 (s, 2H, $PhCH_2O$), 7.18–7.31 (m, 11H, 2-H, 3-H, 4-H, 2*-H, 3*-H, 4*-H, OCONH), 8.08 (d, ^3J = 7.6 Hz, 1H, $NHCH_{Phe}$); ^{13}C NMR (125 MHz, DMSO-*d*$_6$) δ 18.33 ($CHCH_{3,Ala}$), 27.64 ($C(CH_3)_3$), 36.90 ($CHCH_{2,Phe}$), 49.90, 54.20 ($NHCH_{Ala}$, $NHCH_{Phe}$), 65.46 ($PhCH_2O$), 80.75 ($C(CH_3)_3$), 126.57 (C-4*), 127.88 (C-4), 127.81, 128.24, 128.44, 129.34 (C-2, C-3, C-2*, C-3*), 137.15, 137.27 (C-1, C-1*), 155.68 (OCONH), 170.44, 172.54 (CHCO); LC/MS (ESI): H_2O/MeOH, 90:10 to 0:100; *m/z* 427.4 $[M + H]^+$; 100% purity; $C_{24}H_{30}N_2O_5$.

Cbz-alanyl-leucine *tert*-butyl ester

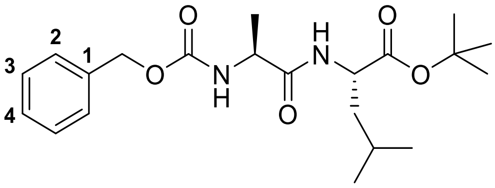

The synthesis was carried out according to GP2 using H-Leu-O*t*Bu × HCl (0.45 g) and DIPEA (0.78 g, 6.00 mmol). Column chromatography on silica gel using petrolum ether / EtOAc (1:1) as eluent afforded Cbz-Ala-Leu-O*t*Bu as a white solid (0.66 g, 1.68 mmol, 84%). mp 90–92 °C, lit.[36] mp 90–92 °C; ^{1}H NMR (500 MHz, DMSO-*d*$_6$) δ 0.86 (2 × d, ^3J = 6.7 Hz, 6H, $CH(CH_3)_{2,Leu}$), 1.20 (d, ^3J = 7.3 Hz, 3H, $CHCH_{3,Ala}$), 1.37 (s, 9H, $C(CH_3)_3$), 1.43–1.53 (m, 2H, $CHCH_{2,Leu}$), 1.59–1.66 (m, 1H, $CH(CH_3)_{2,Leu}$), 4.06–4.14 (m, 2H, $NHCH_{Ala}$, $NHCH_{Leu}$), 5.00 (s, 2H, $PhCH_2O$), 7.29–7.34 (m, 6H, 2-H, 3-H, 4-H, OCONH), 7.99 (d, ^3J = 7.6 Hz, 1H, $NHCH_{Leu}$); ^{13}C NMR (125 MHz, DMSO-*d*$_6$) δ 18.36 ($CHCH_{3,Ala}$), 21.60, 22.82, 24.37 ($CH(CH_3)_{2,Leu}$), 27.72 ($C(CH_3)_3$), 49.81, 51.17 ($NHCH_{Ala}$, $NHCH_{Leu}$), 65.41 ($PhCH_2O$), 80.45 ($C(CH_3)_3$), 127.87 (C-4), 127.80, 128.43 (C-2, C-3), 137.19 (C-1), 155.69 (OCONH), 171.65, 172.62 (CHCO), one signal ($CHCH_{2,Leu}$) is obscured by the DMSO solvent signal; LC/MS (ESI): H_2O/MeOH, 90:10 to 0:100; *m/z* 393.4 $[M + H]^+$; 100% purity; $C_{21}H_{32}N_2O_5$.

Cbz-alanyl-glycine *tert*-butyl ester

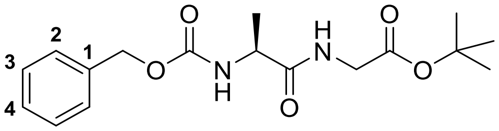

The synthesis was carried out according to GP2 using H-Gly-O*t*Bu × HCl (0.34 g) and DIPEA (0.78 g, 6.00 mmol). Column chromatography on silica gel using petrolum ether / EtOAc (1:1) as eluent afforded Cbz-Ala-Gly-O*t*Bu[37] as an opaque resin (0.28 g, 0.83 mmol, 42%). ^{1}H NMR (500 MHz, DMSO-d_6) δ 1.22 (d, ^3J = 7.3 Hz, 3H, $CHC\underline{H}_{3,Ala}$), 1.39 (s, 9H, $C(C\underline{H}_3)_3$), 3.65 (dd, ^2J = 17.3 Hz, ^3J = 5.7 Hz, 1H, $NHC\underline{H}_{2,Gly}$), 3.74 (dd, ^2J = 17.3 Hz, ^3J = 6.0 Hz, 1H, $NHC\underline{H}_{2,Gly}$), 4.07 (quint, ^3J = 7.3 Hz, 1H, $NHC\underline{H}_{Ala}$), 4.99 (d, ^2J = 12.6 Hz, 1H, $PhC\underline{H}_2O$), 5.03 (d, ^2J = 12.6 Hz, 1H, $PhC\underline{H}_2O$), 7.29–7.41 (m, 6H, 2-$\underline{H}$, 3-$\underline{H}$, 4-$\underline{H}$, $OCON\underline{H}$), 8.13 (t, ^3J = 5.9 Hz, 1H, $N\underline{H}CH_{2,Gly}$); ^{13}C NMR (125 MHz, DMSO-d_6) δ 18.33 ($CH\underline{C}H_{3,Ala}$), 27.84 ($C(\underline{C}H_3)_3$), 41.50 ($NH\underline{C}H_{2,Gly}$), 50.04 ($NH\underline{C}H_{Ala}$), 65.51 ($Ph\underline{C}H_2O$), 80.69 ($\underline{C}(CH_3)_3$), 127.85, 127.89, 128.44 ($\underline{C}$-2, $\underline{C}$-3, $\underline{C}$-4), 137.15 ($\underline{C}$-1), 155.77 ($O\underline{C}ONH$), 168.96, 172.98 ($CH\underline{C}O$); LC/MS (ESI): H_2O/MeOH, 90:10 to 0:100; *m/z* 337.2 $[M + H]^+$; 100% purity; $C_{17}H_{24}N_2O_5$.

Cbz-alanyl-asparagine *tert*-butyl ester

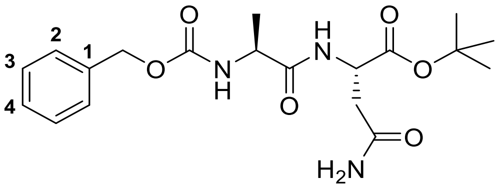

The synthesis was carried out according to GP2 using H-Asn-O*t*Bu × HCl (0.45 g) and DIPEA (0.78 g, 6.00 mmol). Column chromatography on silica gel was not required to obtain Cbz-Ala-Asn-O*t*Bu as a white solid (0.67 g, 1.70 mmol, 85%). mp 120–124 °C, lit.[38] mp 156–158 °C; ^{1}H NMR (500 MHz, DMSO-d_6) δ 1.20 (d, ^3J = 7.0 Hz, 3H, $CHC\underline{H}_{3,Ala}$), 1.36 (s, 9H, $C(C\underline{H}_3)_3$), 2.41–2.52 (m, 2H, $CHC\underline{H}_{2,Asn}$), 4.06 (quint, ^3J = 7.3 Hz, 1H, $NHC\underline{H}_{Ala}$), 4.43 (q, ^3J = 6.6 Hz, 1H, $NHC\underline{H}_{Asn}$), 4.99 (d, ^2J = 12.7 Hz, 1H, $PhC\underline{H}_2O$), 5.02 (d, ^2J = 12.7 Hz, 1H, $PhC\underline{H}_2O$), 6.87 (s, 1H, $CON\underline{H}_2$), 7.29–7.37 (m, 6H, $CON\underline{H}_2$, 2-$\underline{H}$, 3-$\underline{H}$, 4-$\underline{H}$), 7.40 (d, ^3J = 7.9 Hz, 1H, $OCON\underline{H}$), 8.03 (d, ^3J = 7.9 Hz, 1H, $N\underline{H}CH_{Asn}$); ^{13}C NMR (125 MHz, DMSO-d_6) δ 18.35 ($CH\underline{C}H_{3,Ala}$), 27.68 ($C(\underline{C}H_3)_3$), 36.81 ($CH\underline{C}H_{2,Asn}$), 49.51, 49.94 ($NH\underline{C}H_{Ala}$, $NH\underline{C}H_{Asn}$), 65.46 ($Ph\underline{C}H_2O$), 80.53 ($\underline{C}(CH_3)_3$), 127.80, 128.44 ($\underline{C}$-2, $\underline{C}$-3, $\underline{C}$-4), 137.14 ($\underline{C}$-1), 155.71 ($O\underline{C}ONH$), 170.35, 171.16, 172.37 ($CH\underline{C}O$, $CH_2\underline{C}O$); LC/MS (ESI): H_2O/MeOH, 90:10 to 0:100; *m/z* 394.4 $[M + H]^+$; 100% purity; $C_{19}H_{27}N_3O_6$.

Cbz-cyclohexylalanyl-proline *tert*-butyl ester

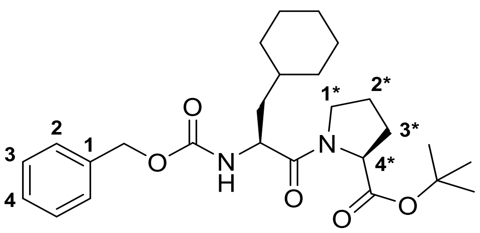

The synthesis was carried out according to GP3 using H-Pro-O*t*Bu × HCl (0.62 g). Column chromatography on silica gel afforded Cbz-Cha-Pro-O*t*Bu as a colorless resin (1.22 g, 2.66 mmol, 89%). ^{1}H NMR (500 MHz, DMSO-*d*$_6$) δ 0.85–0.95 (m, 2H, CH(C<u>H</u>$_2$)$_5$), 1.09–1.22 (m, 4H, C<u>H</u>(C<u>H</u>$_2$)$_5$), 1.36 (s, 9H, C(C<u>H</u>$_3$)$_3$), 1.42–1.50 (m, 1H, CH(C<u>H</u>$_2$)$_5$), 1.58–1.65 (m, 5H, CH(C<u>H</u>$_2$)$_5$, NHCHC<u>H</u>$_2$), 1.73–1.80 (m, 2H, NHCHC<u>H</u>$_2$, 2*-<u>H</u>), 1.89–1.95 (m, 2H, 2*-<u>H</u>, 3*-<u>H</u>), 2.10–2.16 (m, 1H, 3*-<u>H</u>), 3.44 (dt, ^2J = 9.5 Hz, ^3J = 6.7 Hz, 1H, 1*-<u>H</u>), 3.64 (dt, ^2J = 9.5 Hz, ^2J = 6.9 Hz, 1H, 1*-<u>H</u>), 4.17 (dd, ^3J = 4.7 Hz, ^3J = 8.6 Hz, 1H, 4*-<u>H</u>), 4.24–4.32 (m, 1H, NHC<u>H</u>$_{Cha}$), 4.96–5.03 (m, 2H, PhC<u>H</u>$_2$O), 7.30–7.35 (m, 5H, 2-<u>H</u>, 3-<u>H</u>, 4-<u>H</u>), 7.46 (d, ^3J = 8.2 Hz, 1H, OCON<u>H</u>); ^{13}C NMR (125 MHz, DMSO-*d*$_6$) δ 24.71 (<u>C</u>-2*), 25.69, 25.93, 26.15 (CH(<u>C</u>H$_2$)$_5$), 27.70 (C(<u>C</u>H$_3$)$_3$), 28.62 (<u>C</u>-3*), 31.71, 33.53, 33.58 (<u>C</u>H(<u>C</u>H$_2$)$_5$), 38.26 (NHCH<u>C</u>H$_{2,Cha}$), 46.42 (<u>C</u>-1*), 50.17 (NH<u>C</u>H$_{Cha}$), 59.52 (<u>C</u>-4*), 65.46 (Ph<u>C</u>H$_2$O), 80.35 (<u>C</u>(CH$_3$)$_3$), 127.88 (<u>C</u>-4), 127.76, 128.44 (<u>C</u>-2, <u>C</u>-3), 137.25 (<u>C</u>-1), 156.19 (O<u>C</u>ONH), 170.87, 171.06 (CH<u>C</u>O); LC/MS (ESI): H_2O/MeOH, 90:10 to 0:100; *m/z* 459.2 [M + H]$^+$; 92% purity; $C_{26}H_{38}N_2O_5$.

Cbz-(*N*-methyl)leucyl-phenylalanine *tert*-butyl ester

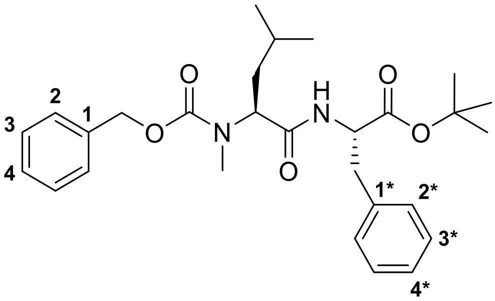

Cbz-(*N*Me)Leu-OH (0.838 g, 3.0 mol), EDC × HCl (0.633 g, 3.3 mmol) and DMAP (18 mg, 0.15 mmol) were suspended in dry CH_2Cl_2 (40 mL) and stirred at rt for 15 min. H-Phe-O*t*Bu × HCl (0.773 g, 3.0 mmol) in DMF (20 mL) was added to the reaction mixture and stirred at rt overnight. The solvent was evaporated and the residue was dissolved in EtOAc (60 mL). The organic phase was extracted with 10% $KHSO_4$ (3 × 50 mL), sat. aq. $NaHCO_3$ (3 × 50 mL), brine (1 × 50 mL) and dried with Na_2SO_4. The solvent was evaporated and the residue was purified by column chromatography on silica gel using petroleum ether / EtOAc (1:1) as eluent to obtain a colorless oil as a mixture of isomers (0.434 g, 0.90 mmol, 30%). ^{1}H NMR (500 MHz, DMSO-*d*$_6$, mixture of rotamers) δ 0.72–0.90 (m, 6H, CH(C<u>H</u>$_3$)$_{2,Leu}$), 1.27–1.41 (m, 1H, CHC<u>H</u>$_{2,Leu}$), 1.33 (s, 9H, C(C<u>H</u>$_3$)$_3$), 1.42–1.61 (m, 2H, CHC<u>H</u>$_{2,Leu}$, C<u>H</u>(CH$_3$)$_{2,Leu}$), 2.57–2.66 (m, 3H, N(C<u>H</u>$_3$)), 2.88–2.96 (m, 1H, CHC<u>H</u>$_{2,Phe}$), 2.97–3.04 (m, 1H, CHC<u>H</u>$_{2,Phe}$), 4.30–4.39 (m, 1H, C<u>H</u>CH$_{2,Phe}$), 4.46–4.71 (m, 1H, N(CH$_3$)C<u>H</u>$_{Leu}$), 4.90–5.18 (m, 2H, PhC<u>H</u>$_2$O), 7.13–7.26, 7.27–

7.41 (each m, 10H, 2-$\underline{H}$, 3-$\underline{H}$, 4-$\underline{H}$, 2*-$\underline{H}$, 3*-$\underline{H}$, 4*-$\underline{H}$), 8.18 (s, 1H, N$\underline{H}$); ^{13}C NMR (125 MHz, DMSO-*d*$_6$, mixture of rotamers) δ 21.61, 23.13, 24.53 ($\underline{C}H(\underline{C}H_3)_{2,Leu}$), 27.68 ($C(\underline{C}H_3)_3$), 29.57 ($N(\underline{C}H_3)$), 36.43, 37.27 ($CH\underline{C}H_{2,Phe}$, $CH\underline{C}H_{2,Leu}$), 54.13 ($N(CH_3)\underline{C}H_{Leu}$), 56.04 ($\underline{C}HCH_{2,Phe}$), 66.49 ($Ph\underline{C}H_2O$), 80.73 ($\underline{C}(CH_3)_3$), 126.47, 127.52, 127.90, 128.20, 128.48, 129.22 ($\underline{C}$-2, $\underline{C}$-3, $\underline{C}$-4, $\underline{C}$-2*, $\underline{C}$-3*, $\underline{C}$-4*), 137.07, 137.61 ($\underline{C}$-1, $\underline{C}$-1*), 156.16 ($O\underline{C}ON(CH_3)$), 170.52, 170.72 ($CH\underline{C}O$); LC/MS (ESI): H_2O/MeCN, 90:10 to 0:100; *m/z* 483.3 $[M + H]^+$; 92% purity; $C_{28}H_{38}N_2O_5$.

Cbz-(*N*-methyl)leucyl-proline *tert*-butyl ester

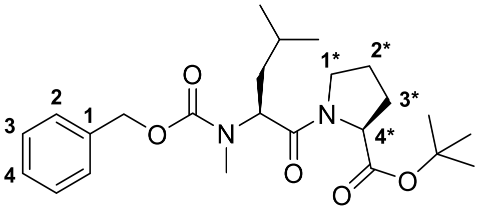

Cbz-(*N*Me)Leu-OH (0.98 g, 3.50 mmol) was dissolved in CH_2Cl_2 (25 mL). EDC (0.62 g, 4.00 mmol) and DMAP (21 mg, 0.18 mmol) were added and the reaction mixture was stirred at rt for 5 min. Upon addition of H-Pro-O*t*Bu (0.60 g, 3.50 mmol) dissolved in CH_2Cl_2 (25 mL), the solution was stirred at rt for 16 h. After evaporation of the solvent, the residue was dispensed in H_2O (50 mL) and EtOAc (3 × 50 mL). The combined organic layers were washed with 10% $KHSO_4$ (30 mL), sat. aq. $NaHCO_3$ (30 mL) and brine (30 mL). The solvent was dried over Na_2SO_4 and evaporated to obtain Cbz-(*N*Me)Leu-Pro-O*t*Bu (1.02 g, 2.36 mmol, 67%) as a colorless oil. ^{1}H NMR (500 MHz, DMSO-*d*$_6$, mixture of rotamers) δ 0.72–0.91 (m, 6H, $CH(C\underline{H}_3)_{2,Leu}$), 1.35–1.38 (m, 9H, $C(C\underline{H}_3)_3$), 1.43–1.58 (m, 3H, $CHC\underline{H}_{2,Leu}$, $C\underline{H}(CH_3)_{2,Leu}$), 1.68–1.80 (m, 1H, 2*-$\underline{H}$), 1.83–1.92 (m, 1H, 2*-$\underline{H}$), 2.00–2.20 (m, 1H, 3*-$\underline{H}$), 2.53–2.81 (m, 3H, $N(C\underline{H}_3)$), 3.23–3.37 (m, 1H, 3*-$\underline{H}$), 3.48–3.51 (m, 1H, 1*-$\underline{H}$), 4.11–4.16 (m, 1H, 1*-$\underline{H}$), 4.46–4.73 (m, 1H, 4*-$\underline{H}$), 4.81–4.98 (m, 1H, $N(CH_3)C\underline{H}_{Leu}$), 5.02–5.18 (m, 2H, $PhC\underline{H}_2O$), 7.29–7.40 (m, 5H, 2-$\underline{H}$, 3-$\underline{H}$, 4-$\underline{H}$); ^{13}C NMR (125 MHz, DMSO-*d*$_6$, signals of the main rotamer are listed) δ 22.22, 23.24, 24.66 ($\underline{C}H(\underline{C}H_3)_{2,Leu}$), 24.85 ($\underline{C}$-2*), 28.00 ($C(\underline{C}H_3)_3$), 28.95 ($\underline{C}$-3*), 29.74 ($N(\underline{C}H_3)$), 37.47 ($CH\underline{C}H_{2,Leu}$), 46.84 ($\underline{C}$-1*), 54.13 ($N(CH_3)\underline{C}H_{Leu}$), 59.88 ($\underline{C}$-4*), 67.00 ($Ph\underline{C}H_2O$), 80.70 ($\underline{C}(CH_3)_3$), 127.89, 128.50, 128.84 ($\underline{C}$-2, $\underline{C}$-3, $\underline{C}$-4), 137.30 ($\underline{C}$-1), 156.39 ($O\underline{C}ON(CH_3)$), 169.47, 171.24 ($CH\underline{C}O$); LC/MS (ESI): H_2O/MeOH, 90:10 to 0:100; *m/z* 433.8 $[M + H]^+$; 99% purity; $C_{24}H_{36}N_2O_5$.

(*S*)-2-Hydroxy-4-methylpentanoyl-phenylalanine *tert*-butyl ester (34)

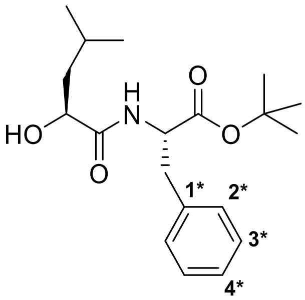

(*S*)-2-Hydroxy-4-methylpentanoic acid (0.40 g, 3.00 mmol), HOBt × H_2O (0.43 g, 3.15 mmol), EDC × HCl (0.60 g, 3.15 mmol) and TEA (0.96 g, 9.50 mmol) were suspended in dry DMF (40 ml) and stirred at rt for 30 min. A solution of H-Phe-O*t*Bu × HCl (0.77 g, 3.00 mmol) in DMF (20 ml) was added to the reaction mixture that was stirred at rt for 18 h. The solvent was evaporated and the residue was redissolved in EtOAc (60 ml). The organic phase was washed with 10% citric acid (3 × 50 ml), sat. aq. $NaHCO_3$ (3 × 50 ml) and brine (50 ml) and dried over Na_2SO_4. Evaporation of the solvent resulted in intermediate **35** as a yellow solid (0.78 g, 2.32 mmol, 77%). mp 61–62 °C. ^{1}H NMR (500 MHz, DMSO-*d*$_6$) δ 0.83 (d, ^3J = 6.6 Hz, 6H, CH(C<u>H</u>$_3$)$_2$), 1.25–1.36 (m, 11H, C(C<u>H</u>$_3$)$_3$, OCHC<u>H</u>$_2$), 1.62–1.74 (m, 1H, C<u>H</u>(CH$_3$)$_2$), 2.95–3.03 (m, 2H, CHC<u>H</u>$_{2,Phe}$), 3.81–3.86 (m, 1H, OC<u>H</u>CH$_2$), 4.40–4.46 (m, 1H, NHC<u>H</u>$_{Phe}$), 5.44 (d, ^3J = 5.7 Hz, 1H, O<u>H</u>), 7.16–7.22 (m, 3H, 2*-<u>H</u>, 4*-<u>H</u>), 7.24–7.29 (m, 2H, 3*-<u>H</u>), 7.67 (d, ^3J = 7.9 Hz, 1H, N<u>H</u>CH$_{Phe}$); ^{13}C NMR (125 MHz, DMSO-*d*$_6$) δ 21.62, 23.78, 23.92 (<u>C</u>H(<u>C</u>H$_3$)$_2$), 27.68 (C(<u>C</u>H$_3$)$_3$), 37.07 (CH<u>C</u>H$_{2,Phe}$), 43.56 (OCH<u>C</u>H$_2$), 53.19 (NH<u>C</u>H$_{Phe}$), 69.52 (O<u>C</u>HCH$_2$), 81.09 (<u>C</u>(CH$_3$)$_3$), 126.61 (<u>C</u>-4*), 128.24, 129.36 (<u>C</u>-2*, <u>C</u>-3*), 137.12 (<u>C</u>-1*), 170.51, 174.25 (CH<u>C</u>O); LC/MS (ESI): H_2O/MeOH, 90:10 to 0:100; *m/z* 336.0 [M + H]$^+$; 96% purity; $C_{19}H_{29}NO_4$.

(*S*)-2-Hydroxy-4-methylpentanoyl-proline *tert*-butyl ester (35)

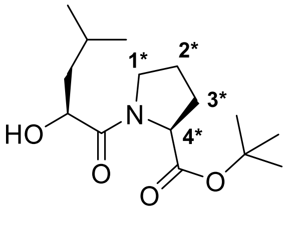

(*S*)-2-Hydroxy-4-methylpentanoic acid (0.40 g, 3.00 mmol), HOBt × H_2O (0.43 g, 3.15 mmol), EDC × HCl (0.60 g, 3.15 mmol) and TEA (0.96 g, 9.50 mmol) were suspended in dry DMF (50 ml) and stirred at rt for 30 min. A solution of H-Pro-O*t*Bu × HCl (0.62 g, 3.00 mmol) in DMF (20 ml) was added to the reaction mixture that was stirred at rt for 18 h. The solvent was evaporated and the residue was redissolved in EtOAc (60 ml). The organic layer was washed with 10% $KHSO_4$ (3 × 50 ml), sat. aq. $NaHCO_3$ (3 × 50 ml) and brine (50 ml) and dried over Na_2SO_4. After evaporation of EtOAc, the crude product was purified by column chromatography on silica gel using petrolum ether / EtOAc (1:1) as eluent to obtain **34** as a colorless oil (0.56 g, 1.96 mmol, 65%). ^{1}H NMR (500 MHz, DMSO-*d*$_6$) δ 0.90 (2 × d, ^3J = 6.6 Hz, 6H, CH(C<u>H</u>$_3$)$_2$), 1.32–1.39 (m, 11H, C(C<u>H</u>$_3$)$_3$, OCHC<u>H</u>$_2$), 1.72–1.92 (m, 4H

$C\underline{H}(CH_3)_2$, 2*-$\underline{H}$, 3*-$\underline{H}$), 2.10–2.18 (m, 1H, 3*-$\underline{H}$), 3.39 (dt, ^2J = 9.6 Hz, ^3J = 6.7 Hz, 1H, 1*-$\underline{H}$), 3.64 (dt, ^2J = 10.1 Hz, ^3J = 6.7 Hz, 1H, 1*-$\underline{H}$), 4.14–4.21 (m, 2H, 4*-$\underline{H}$, $OC\underline{H}CH_2$), 4.60 (d, ^3J = 7.6 Hz, 1H, $O\underline{H}$); ^{13}C NMR (125 MHz, DMSO-d_6) δ 21.61, 23.57, 24.08 ($\underline{C}H(\underline{C}H_3)_2$), 24.77 ($\underline{C}$-2*), 27.70 ($C(\underline{C}H_3)_3$), 28.53 ($\underline{C}$-3*), 42.90 ($OCH\underline{C}H_2$), 46.19 ($\underline{C}$-1*), 59.56 ($\underline{C}$-4*), 67.30 ($O\underline{C}HCH_2$), 80.29 ($\underline{C}(CH_3)_3$), 171.11, 172.52 ($CH\underline{C}O$); LC/MS (ESI): H_2O/MeOH, 90:10 to 0:100; *m/z* 286.0 $[M + H]^+$; 94% purity; $C_{15}H_{27}NO_4$.

N-(4-Methylpentanoyl)-(S)-4-isopropyloxazolidin-2-one (36)

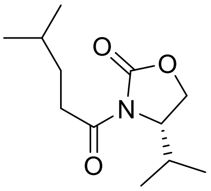

(*S*)-4-isopropyloxazolidin-2-one (6.46 g, 50.0 mmol) was dissolved in anhydrous THF (100 mL) and added dropwise over 1 h to a solution of n-butyllithium (20 mL, 50.0 mmol, 2.5M in hexane) cooled to -78 °C. Stirring was continued for 30 min at -78 °C. 4-Methylpentanoyl chloride (8.92 g, 66.0 mmol) was dissolved in anhydrous THF (25 mL) and added dropwise to the reaction mixture within 15 min. After 30 min and warming up to rt, sat. aq. NH_4Cl (45 mL) was added. The organic solvent was evaporated and the residue was quenched with H_2O (25 mL). The aqueous solution was extracted with CH_2Cl_2 (3 × 65 mL) and the combined organic layer was washed with 1M NaOH (65 mL), H_2O (65 mL), brine (65 mL) and dried over Na_2SO_4. After evaporation of the solvent, the crude residue was purified by column chromatography on silica gel using petrolum ether / EtOAc (6:1) to obtain a colorless oil (10.8 g, 95%). ^1H NMR (500 MHz, DMSO-d_6) δ 0.77 (d, ^3J = 6.9 Hz, 3H, $C\underline{H}_3$), 0.84 (d, ^3J = 7.0 Hz, 3H, $C\underline{H}_3$), 0.86 (d, ^3J = 1.6 Hz, 3H, $C\underline{H}_3$), 0.87 (d, ^3J = 1.6 Hz, 3H, $C\underline{H}_3$), 1.38–1.50 (m, 2H, $C\underline{H}_2CH_2CO$), 1.51–1.59 (m, 1H, $CH_2C\underline{H}(CH_3)_2$), 2.11–2.20 (m, 1H, $C\underline{H}(CH_3)_2$), 2.69–2.77 (m, 1H, $CH_2C\underline{H}_2CO$), 2.86–2.94 (m, 1H, $CH_2C\underline{H}_2CO$), 4.25 (dd, ^2J = 8.8 Hz, ^3J = 3.0 Hz, 1H, $C\underline{H}_2O$), 4.27–4.31 (m, 1H, $C\underline{H}_2O$), 4.32–4.36 (m, 1H, $NC\underline{H}$); ^{13}C NMR (125 MHz, DMSO-d_6) δ 14.73, 17.60 ($CH(\underline{C}H_3)_2$), 22.30, 22.32 ($CH_2CH(\underline{C}H_3)_2$), 27.23 ($CH_2\underline{C}H(CH_3)_2$), 28.35 ($\underline{C}H(CH_3)_2$), 32.88 ($CH_2\underline{C}H_2CO$), 33.10 ($\underline{C}H_2CH_2CO$), 57.99 ($N\underline{C}H$), 63.48 ($\underline{C}H_2O$), 154.08 ($\underline{C}OO$), 172.74 ($CH_2\underline{C}O$); LC/MS (ESI): H_2O/MeOH, 90:10 to 0:100; *m/z* 228.0 $[M + H]^+$; 99% purity; $C_{12}H_{21}NO_3$.

N-((R)-2-Benzamidomethyl-4-methylpentanoyl)-(S)-4-isopropyloxazolidin-2-one (37)

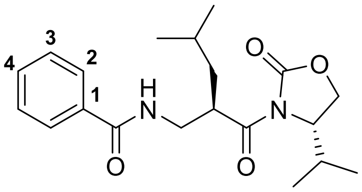

N-(Chloromethyl)benzamide (11.4 g, 50.0 mmol) was dissolved in anhydrous CH_2Cl_2 (200 mL) and cooled to 0 °C. $TiCl_4$ (9.49 g, 50.0 mmol) was added slowly and the resulting yellow suspension was

stirred for 15 min. Subsequent addition of TEA (5.06 g, 50.0 mmol) gave a dark red solution that was stirred for 45 min at 0 °C. Subsequently, compound **36** (10.2 g, 60.0 mmol) was added and the mixture was stirred for 1 h at 0 °C. After addition of sat. aq. NH_4Cl (175 mL), the organic layer was washed with 1M HCl (175 mL). The acidic aqueous layer was extracted with CH_2Cl_2 (200 mL). The combined organic layer was dried over Na_2SO_4. Upon evaporation of the solvent, the crude residue was purified by column chromatography on silica gel using petrolum ether / EtOAc (7:3) to obtain a colorless oil (14.3 g, 79%). ^{1}H NMR (500 MHz, DMSO-d_6) δ 0.63 (d, ^3J = 6.9 Hz, 3H, CH_3), 0.79 (d, ^3J = 7.1 Hz, 3H, CH_3), 0.86 (d, ^3J = 6.3 Hz, 3H, CH_3), 0.88 (d, ^3J = 6.3 Hz, 3H, CH_3), 1.26–1.34 (m, 1H, $CH_2CH(CH_3)_2$), 1.52–1.64 (m, 2H, $CH_2CH(CH_3)_2$), 2.12–2.20 (m, 1H, $CH(CH_3)_2$), 3.40–3.48 (m, 1H, $NHCH_2$), 3.50–3.54 (m, 1H, $NHCH_2$), 4.16–4.21 (m, 1H, $NHCH_2CH$), 4.23 (dd, ^2J = 9.1 Hz, ^3J = 2.8 Hz, 1H, CH_2O), 4.29 (dd, ^2J = 9.0 Hz, ^3J = 8.2 Hz, 1H, CH_2O), 4.37 (m, 1H, NCH), 7.39–7.45 (m, 2H, 3-H), 7.45–7.51 (m, 1H, 4-H), 7.75–7.80 (m, 2H, 2-H), 8.45 (t, ^3J = 5.9 Hz, 1H, NH); ^{13}C NMR (125 MHz, DMSO-d_6) δ 14.45, 17.68 ($CH(CH_3)_2$), 22.61, 22.87 ($CH_2CH(CH_3)_2$), 25.51, 27.99 ($CH_2CH(CH_3)_2$, $CH(CH_3)_2$), 39.14 ($CH_2CH(CH_3)_2$), 40.86, 41.53 ($NHCH_2CH$), 58.27 (NCH), 63.16 (CH_2O), 127.25 (C-2), 128.24 (C-3), 131.15 (C-4), 134.52 (C-1), 153.70 (COO), 166.32 (PhCO), 174.71 (CHCO); LC/MS (ESI): H_2O/MeOH, 90:10 to 0:100; *m/z* 361.1 $[M + H]^+$; 94% purity; $C_{20}H_{28}N_2O_4$.

(*R*)-2-Benzamidomethyl-4-methylpentanoic acid (Bz-APA-OH; 38)

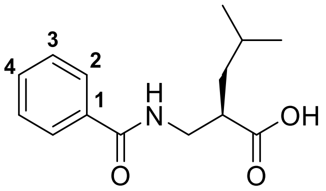

Compound **37** (9.01 g, 25.0 mmol) was dissolved in THF / H_2O (4:1, 125 mL) and cooled to 0 °C. H_2O_2 (30% aq., 100 mmol) and LiOH × H_2O (1.68 g, 40 mmol) were added and the reaction mixture was stirred for 1 h at 0 °C. Subsequently, sat. aq. $NaHSO_3$ (20 mL) was added. Upon washing of the reaction mixture with CH_2Cl_2 (3 × 100 mL), the aqueous layer was acidified to pH 2 with 6M HCl and extracted with EtOAc (3 × 100 mL). The EtOAc layer was dried over Na_2SO_4, filtered and evaporated to dryness to yield white needles (3.62 g, 58%). mp 90–92 °C; lit.[39] mp 90–91 °C; ^{1}H NMR (500 MHz, DMSO-d_6) δ 0.86 (2 × d, ^3J = 6.6 Hz, 6H, $CH(CH_3)_2$), 1.22–1.30 (m, 1H, $CHCH_2CH$), 1.44–1.51 (m, 1H, $CHCH_2CH$), 1.54–1.60 (m, 1H, $CH(CH_3)_2$), 2.66–2.73 (m, 1H, $NHCH_2CH$), 3.30–3.35 (m, 1H, $NHCH_2CH$), 3.36–3.43 (m, 1H, $NHCH_2CH$), 7.42–7.46 (m, 2H, 3-H), 7.48–7.53 (m, 1H, 4-H), 7.79–7.83 (m, 2H, 2-H), 8.52 (t, ^3J = 5.8 Hz, 1H, NH), 12.15 (s, 1H, COOH); ^{13}C NMR (125 MHz, DMSO-d_6) δ 22.75, 23.84 ($CH(CH_3)_2$), 26.62 ($CH(CH_3)_2$), 39.75 ($CHCH_2CH$), 42.59 ($NHCH_2CH$), 44.09 ($NHCH_2CH$), 128.00 (C-2), 129.08 (C-3), 131.95 (C-4), 135.35 (C-1), 167.20 (PhCO), 176.70 (COOH); LC/MS (ESI): H_2O/MeOH, 90:10 to 0:100; *m/z* 250.3 $[M + H]^+$; 100% purity; $C_{14}H_{19}NO_3$.

(*R*)-2-Carboxy-4-methylpentylammonium chloride (**39**)

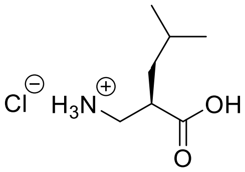

Compound **38** (2.49 g, 10.0 mmol) was dissolved in 37% HCl / AcOH / H_2O (20:10:10, 40 mL) and stirred under reflux for 48 h. After cooling to rt, the reaction mixture was diluted with H_2O (25 mL) and washed with Et_2O (3 × 150 mL). The aqueous layer was evaporated at < 1 mbar to give a white solid (1.56 g, 86%). mp 192–194 °C; ^{1}H NMR (600 MHz, DMSO-d_6) δ 0.86 (d, ^3J = 2.7 Hz, 3H, $C\underline{H}_3$), 0.87 (d, ^3J = 2.8 Hz, 3H, $C\underline{H}_3$), 1.34 (m, 1H, $CHC\underline{H}_2CH$), 1.44 (m, 1H, $CHC\underline{H}_2CH$), 1.53–1.63 (m, 1H, $C\underline{H}(CH_3)_2$), 2.67–2.73 (m, 1H, $NH_3CH_2C\underline{H}$), 2.78 (dd, ^2J = 12.8 Hz, ^3J = 5.0 Hz, 1H, $NH_3C\underline{H}_2$), 2.94 (dd, ^2J = 12.8 Hz, ^3J = 8.2 Hz, 1H, $NH_3C\underline{H}_2$), 8.18 (s, 3H, $N\underline{H}_3$), 12.71 (s, 1H, $COO\underline{H}$); ^{13}C NMR (150 MHz, DMSO-d_6) δ 22.15, 22.65, 25.44 ($\underline{C}H(\underline{C}H_3)_2$), 38.63 ($CH\underline{C}H_2CH$), 40.00, 40.98 ($NH_3\underline{C}H_2\underline{C}H$), 174.81 ($\underline{C}OOH$); LC/MS (ESI): H_2O/MeOH, 90:10 to 0:100; *m/z* 146.0 $[M + H]^+$; $C_7H_{15}NO_2 \cdot HCl$.

2-(Fmoc-aminomethyl)-4-methylpentanoic acid (Fmoc-APA-OH)

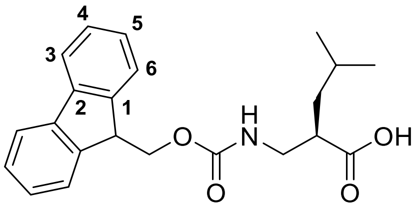

A solution of **39** (0.91 g, 5.00 mmol), Fmoc-succinimide (1.86 g, 5.50 mmol) and TEA (1.67 g, 2.29 mL, 16.5 mmol) in 1,4-dioxane / H_2O (2:1, 22.5 mL) was stirred at rt for 8 h. Subsequently, the solvent was evaporated and the residue was dispersed between EtOAc (150 mL) and 0.1N aq. sodium citrate (150 mL). The aqueous layer was extracted with EtOAc. The combined organic layer was washed with H_2O (75 mL) and dried over Na_2SO_4. Upon evaporation of the solvent, the crude residue was purified by column chromatography on silica gel using petrolum ether / EtOAc (1:1) as eluent to give Fmoc-APA-OH as a white solid (0.70 g, 38%). mp 134–136 °C; lit.[40] mp 134–135 °C; ^{1}H NMR (500 MHz, DMSO-d_6) δ 0.85 (2 × d, ^3J = 5.8 Hz, 6H, $CH(C\underline{H}_3)_2$), 1.21 (m, 1H, $CHC\underline{H}_2CH$), 1.41 (m, 1H, $CHC\underline{H}_2CH$), 1.50–1.60 (m, 1H, $C\underline{H}(CH_3)_2$), 2.51–2.55 (m, 1H, $NHCH_2C\underline{H}$), 3.01–3.07 (m, 1H, $NHC\underline{H}_2$), 3.12–3.21 (m, 1H, $NHC\underline{H}_2$), 4.20 (t, ^3J = 7.0 Hz, 1H, $C\underline{H}CH_2O$), 4.26 (d, ^3J = 7.1 Hz, 2H, $CHC\underline{H}_2O$), 7.31 (t, ^3J = 7.3 Hz, 2H, 5-$\underline{H}$), 7.40 (t, ^3J = 7.4 Hz, 3H, 4-$\underline{H}$, $N\underline{H}$), 7.68 (dd, ^4J = 3.0 Hz, ^3J = 7.9 Hz, 2H, 6-$\underline{H}$), 7.87 (d, ^3J = 7.5 Hz, 2H, 3-$\underline{H}$), 12.17 (s, 1H, $COO\underline{H}$); ^{13}C NMR (125 MHz, DMSO-d_6) δ 21.99, 23.07 ($CH(\underline{C}H_3)_2$), 25.80 ($\underline{C}H(CH_3)_2$), 38.70 ($CH\underline{C}H_2CH$), 42.87, 43.71 ($NH\underline{C}H_2\underline{C}H$), 46.84 ($\underline{C}HCH_2O$), 65.53 ($\underline{C}H_2O$), 120.21 ($\underline{C}$-6), 125.32 ($\underline{C}$-5), 127.16, 127.72 ($\underline{C}$-3, $\underline{C}$-4), 140.84, 143.99 ($\underline{C}$-1, $\underline{C}$-2), 156.25 ($O\underline{C}ONH$), 175.84 ($\underline{C}OOH$); LC/MS (ESI): H_2O/MeOH, 90:10 to 0:100; *m/z* 368.3 $[M + H]^+$, 385.2 $[M + NH_4]^+$; 98% purity; $C_{22}H_{25}NO_4$.

7.2. Dipeptide carbamates of type A

Phenoxycarbonyl-leucyl-phenylalanine *tert*-butyl ester

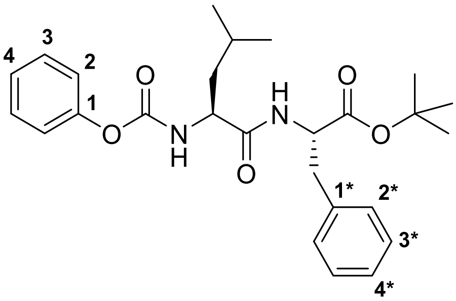

The synthesis was carried out according to GP7 using Cbz-Leu-Phe-O*t*Bu (0.94 g) for the hydrogenation. Subsequently, H-Leu-Phe-O*t*Bu (0.33 g, 1.00 mmol) was reacted with phenyl chloroformate (0.17 g, 1.10 mmol) to generate the desired carbamate. Column chromatography on silica gel using petrolum ether / EtOAc (4:1) as eluent afforded the product as a white solid (0.37 g, 0.82 mmol, 82%). mp 75–79 °C; ^{1}H NMR (500 MHz, DMSO-d_6) δ 0.87-0.91 (2 × d, ^{3}J = 6.6 Hz, 6H, $CH(C\underline{H}_3)_{2,Leu}$), 1.31 (s, 9H, $C(C\underline{H}_3)_3$), 1.39–1.52 (m, 2H, $CHC\underline{H}_{2,Leu}$), 1.63–1.68 (m, 1H, $C\underline{H}(CH_3)_{2,Leu}$,), 2.91–3.00 (m, 2H, $CHC\underline{H}_{2,Phe}$), 4.09 (dt, ^{3}J = 4.7 Hz, ^{3}J = 9.2 Hz, 1H, 1H, $NHC\underline{H}_{Phe}$), 4.34–4.38 (q, ^{3}J = 7.4 Hz, 1H, $NHC\underline{H}_{Leu}$), 7.05 (d, ^{3}J = 7.8 Hz, 2H, 2-$\underline{H}$), 7.17–7.27 (m, 6H, 4-$\underline{H}$, 2*-$\underline{H}$, 3*-$\underline{H}$, 4*-$\underline{H}$), 7.36 (t, ^{3}J = 7.6 Hz, 2H, 3-$\underline{H}$), 7.82 (d, ^{3}J = 8.5 Hz, 1H, $OCON\underline{H}$), 8.24 (d, ^{3}J = 7.6 Hz, 1H, $N\underline{H}CH_{Phe}$); ^{13}C NMR (125 MHz, DMSO-d_6) δ 21.66, 23.09, 24.34 ($\underline{C}H(\underline{C}H_3)_{2,Leu}$), 27.64 ($C(\underline{C}H_3)_3$), 36.83 ($CH\underline{C}H_{2,Phe}$), 40.86 ($CH\underline{C}H_{2,Leu}$), 53.19, 54.23 ($NH\underline{C}H_{Phe}$, $NH\underline{C}H_{Leu}$), 80.72 ($\underline{C}(CH_3)_3$), 121.70 ($\underline{C}$-2), 125.03 ($\underline{C}$ 4), 126.55 ($\underline{C}$-4*), 128.24 ($\underline{C}$-2*), 129.32, 129.36 ($\underline{C}$-3, $\underline{C}$-3*), 137.34 ($\underline{C}$-1*), 151.20 ($\underline{C}$-1), 154.19 ($O\underline{C}ONH$), 170.48, 172.04 ($CH\underline{C}O$); LC/MS (ESI): H_2O/MeOH, 90:10 to 0:100; *m/z* 455.3 $[M + H]^+$; 98% purity; Q-TOF: HRMS (ESI): *m/z* $[M + H]^+$ calcd. for $C_{26}H_{34}N_2O_5$: 455.2540, found: 455.2543.

Phenoxycarbonyl-leucyl-phenylalanine (1)

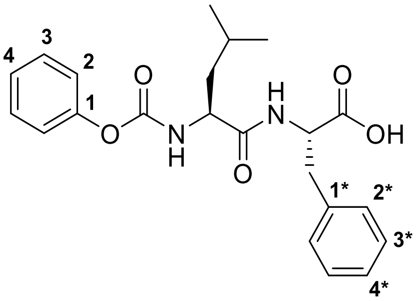

The synthesis was carried out according to GP8 using the corresponding *tert*-butyl ester (91 mg, 0.20 mmol). Column chromatography on silica gel using EtOAc as eluent afforded the product as a white solid (46 mg, 0.12 mmol, 58%). mp 146–149 °C; ^{1}H NMR (500 MHz, DMSO-d_6) δ 0.87 (2 × d, ^{3}J = 6.6 Hz, 6H, $CH(C\underline{H}_3)_{2,Leu}$), 1.37–1.49 (m, 2H, $CHC\underline{H}_{2,Leu}$), 1.60–1.66 (m, 1H, $C\underline{H}(CH_3)_{2,Leu}$), 2.92 (dd, ^{2}J = 13.9 Hz, ^{3}J = 8.9 Hz, 1H, $CHC\underline{H}_{2,Phe}$), 3.06 (dd, ^{2}J = 13.9 Hz, ^{3}J = 5.0 Hz, 1H, $CHC\underline{H}_{2,Phe}$), 4.06

(dt, ^3J = 4.8 Hz, ^3J = 9.5 Hz, 1H, NHC$\underline{H}_{Phe}$), 4.45 (dt, ^3J = 5.1 Hz, ^3J = 8.3 Hz, 1H, NHC$\underline{H}_{Leu}$), 7.05 (d, ^3J = 7.6 Hz, 2H, 2-$\underline{H}$), 7.17–7.24 (m, 6H, 4-$\underline{H}$, 2*-$\underline{H}$, 3*-$\underline{H}$, 4*-$\underline{H}$), 7.36 (t, ^3J = 7.6 Hz, 2H, 3-$\underline{H}$), 7.81 (d, ^3J = 8.5 Hz, 1H, OCON$\underline{H}$), 8.11 (d, ^3J = 7.85 Hz, 1H, N$\underline{H}$CH$_{Phe}$), 12.67 (s, 1H, COO$\underline{H}$); ^{13}C NMR (125 MHz, DMSO-d_6) δ 21.66, 23.08, 24.30 ($\underline{C}$H($\underline{C}$H$_3$)$_{2,Leu}$), 36.77 (CH$\underline{C}$H$_{2,Phe}$), 40.74 (CH$\underline{C}$H$_{2,Leu}$), 53.38 (NH$\underline{C}$H$_{Phe}$, NH$\underline{C}$H$_{Leu}$), 121.70 ($\underline{C}$-2), 125.03 ($\underline{C}$-4), 126.50 ($\underline{C}$-4*), 128.25 ($\underline{C}$-2*), 129.26, 129.36 ($\underline{C}$-3, $\underline{C}$-3*), 137.57 ($\underline{C}$-1*), 151.18 ($\underline{C}$-1), 154.15 (O$\underline{C}$ONH), 171.97, 172.87 (CH$\underline{C}$O); LC/MS (ESI): H_2O/MeOH, 90:10 to 0:100; *m/z* 399.2 [M + H]$^+$; 97% purity; Q-TOF: HRMS (ESI): *m/z* [M + H]$^+$ calcd. for $C_{22}H_{26}N_2O_5$: 399.1914, found: 399.1911.

Phenoxycarbonyl-leucyl-proline *tert*-butyl ester

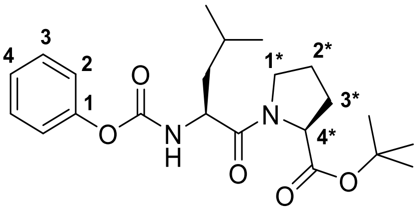

The synthesis was carried out according to GP7 using Cbz-Leu-Pro-O*t*Bu (0.84 g) for the hydrogenation. Subsequently, H-Leu-Pro-O*t*Bu (0.17 g, 0.60 mmol) was reacted with phenyl chloroformate (0.11 g, 0.70 mmol) to generate the desired carbamate. Column chromatography on silica gel using petrolum ether / EtOAc (4:1) as eluent afforded the product as a white solid (0.18 g, 0.45 mmol, 75%). mp 73–76 °C; ^{1}H NMR (500 MHz, DMSO-d_6) δ 0.93 (2 × d, ^3J = 6.6 Hz, 6H, CH(C$\underline{H}_3$)$_{2,Leu}$), 1.37 (s, 9H, C(C$\underline{H}_3$)$_3$), 1.40–1.45 (m, 1H, CHC$\underline{H}_{2,Leu}$), 1.54–1.60 (m, 1H, CHC$\underline{H}_{2,Leu}$), 1.71–1.81 (m, 2H, C$\underline{H}$(CH$_3$)$_{2,Leu}$, 2*-$\underline{H}$), 1.91–1.94 (m, 2H, 2*-$\underline{H}$, 3*-$\underline{H}$), 2.11–2.18 (m, 1H, 3*-$\underline{H}$), 3.48 (dt, ^2J = 9.5 Hz, ^3J = 6.6 Hz, 1H, 1*-$\underline{H}$), 3.67 (dt, ^2J = 9.5 Hz, ^3J = 6.9 Hz, 1H, 1*-$\underline{H}$), 4.20 (dd, ^3J = 5.1 Hz, ^3J = 8.5 Hz, 1H, 4*-$\underline{H}$), 4.29–4.33 (m, 1H, NHC$\underline{H}_{Leu}$), 7.06 (d, ^3J = 7.6 Hz, 2H, 2-$\underline{H}$), 7.18 (t, ^3J = 7.3 Hz, 1H, 4-$\underline{H}$), 7.36 (t, ^3J = 8.5 Hz, 2H, 3-$\underline{H}$), 8.00 (d, ^3J = 8.2 Hz, 1H, OCON$\underline{H}$); ^{13}C NMR (125 MHz, DMSO-d_6) δ 21.45, 23.23, 24.33 ($\underline{C}$H($\underline{C}$H$_3$)$_{2,Leu}$), 24.73 ($\underline{C}$-2*), 27.70 (C($\underline{C}$H$_3$)$_3$), 28.65 ($\underline{C}$-3*), 46.48 ($\underline{C}$-1*), 51.12 (NH$\underline{C}$H$_{Leu}$), 59.59 ($\underline{C}$-4*), 80.40 ($\underline{C}$(CH$_3$)$_3$), 121.73 ($\underline{C}$-2), 125.06 ($\underline{C}$-4), 129.35 ($\underline{C}$-3), 151.15 ($\underline{C}$-1), 154.49 (O$\underline{C}$ONH), 170.44, 171.00 (CH$\underline{C}$O), one signal (CH$\underline{C}$H$_{2,Leu}$) is obscured by the DMSO solvent signal; LC/MS (ESI): H_2O/MeOH, 90:10 to 0:100; *m/z* 405.5 [M + H]$^+$; 95% purity; Q-TOF: HRMS (ESI): *m/z* [M + H]$^+$ calcd. for $C_{22}H_{32}N_2O_5$: 405.2384, found: 405.2384.

Phenoxycarbonyl-leucyl-proline

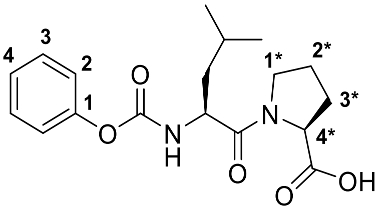

The synthesis was carried out according to GP8 using the corresponding *tert*-butyl ester (101 mg, 0.25 mmol). Column chromatography on silica gel using EtOAc as eluent afforded the product as a white solid (81 mg, 0.23 mmol, 93%). mp 55–58 °C; ^{1}H NMR (500 MHz, DMSO-d_6) δ 0.92 (2 × d, ^3J = 6.6 Hz, 6H, CH(CH_3)$_{2,Leu}$), 1.40–1.46 (m, 1H, CHC$H_{2,Leu}$), 1.52–1.58 (m, 1H, CHC$H_{2,Leu}$), 1.72–1.77 (m, 1H, CH(CH$_3$)$_{2,Leu}$), 1.79–1.85 (m, 1H, 2*-H), 1.90–1.94 (m, 2H, 2*-H, 3*-H), 2.11–2.18 (m, 1H, 3*-H), 4.27 (dd, ^3J = 4.7 Hz, ^3J = 8.8 Hz, 1H, 4*-H), 4.29–4.34 (m, 1H, NHCH_{Leu}), 7.06 (d, ^3J = 7.9 Hz, 2H, 2-H), 7.18 (t, ^3J = 7.3 Hz, 1H, 4-H), 7.36 (t, ^3J = 7.6 Hz, 2H, 3-H), 7.98 (d, ^3J = 7.9 Hz, 1H, OCONH), two signals (1*-H) are obscured by the H_2O signal, one signal (COOH) is not visible; ^{13}C NMR (125 MHz, DMSO-d_6) δ 21.55, 23.23, 24.27 (CH(CH$_3$)$_{2,Leu}$), 24.74 (C-2*), 28.66 (C-3*), 46.51 (C-1*), 51.07 (NHCH_{Leu}), 58.68 (C-4*), 121.75 (C-2), 125.09 (C-4), 129.37 (C-3), 151.15 (C-1), 154.48 (OCONH), 170.48 (CHCO), 173.31 (COOH), one signal (CHC$H_{2,Leu}$) is obscured by the DMSO solvent signal; LC/MS (ESI): H_2O/MeOH, 90:10 to 0:100; *m/z* 349.2 [M + H]$^+$; 99% purity; Q-TOF: HRMS (ESI): *m/z* [M + H]$^+$ calcd. for $C_{18}H_{24}N_2O_5$: 349.1758, found: 349.1753.

4-Chlorophenoxycarbonyl-leucyl-phenylalanine *tert*-butyl ester (2)

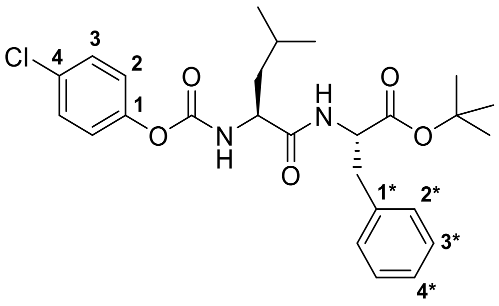

The synthesis was carried out according to GP7 using Cbz-Leu-Phe-O*t*Bu (0.94 g) for the hydrogenation. Subsequently, H-Leu-Phe-O*t*Bu (0.43 g, 1.30 mmol) was reacted with 4-chlorophenyl chloroformate (0.28 g, 1.45 mmol) to generate the desired carbamate. Column chromatography on silica gel using petrolum ether / EtOAc (4:1) as eluent afforded the product as a white solid (0.58 g, 1.18-mmol, 91%). mp 105–108 °C; ^{1}H NMR (500 MHz, DMSO-d_6) δ 0.88 (2 × d, ^3J = 6.6 Hz, 6H, CH(CH_3)$_{2,Leu}$), 1.31 (s, 9H, C(CH_3)$_3$), 1.41–1.52 (m, 2H, CHC$H_{2,Leu}$), 1.62–1.68 (m, 1H, CH(CH$_3$)$_{2,Leu}$), 2.91–3.00 (m, 2H, CHC$H_{2,Phe}$), 4.09 (dt, ^3J = 5.1 Hz, ^3J = 9.2 Hz, 1H, NHCH_{Phe}), 4.35 (q, ^3J = 7.4 Hz, 1H, NHCH_{Leu}), 7.09 (d, ^3J = 8.8 Hz, 2H, 2-H), 7.18–7.25 (m, 5H, 2*-H, 3*-H, 4*-H), 7.42 (d, ^3J = 8.8 Hz, 2H, 3-H), 7.91 (d, ^3J = 8.5 Hz, 1H, OCONH), 8.27 (d, ^3J = 7.6 Hz, 1H, NHCH_{Phe}); ^{13}C NMR (125 MHz, DMSO-d_6) δ 21.64, 23.09, 24.32 (CH(CH$_3$)$_{2,Leu}$), 27.64 (C(CH$_3$)$_3$), 36.80 (CHC$H_{2,Phe}$), 40.81 (CHC$H_{2,Leu}$), 53.19, 54.25 (NHCH_{Phe}, NHCH_{Leu}), 80.71 (C(CH$_3$)$_3$), 123.54 (C-2), 126.55 (C-4*), 128.23 (C-2*), 129.25 (C-4), 129.27 (C-3, C-3*), 137.34 (C-1*), 149.98 (C-1), 153.84 (OCONH), 170.47, 171.93 (CHCO); LC/MS (ESI): H_2O/MeOH, 90:10 to 0:100; *m/z* 489.3 [M + H]$^+$; 100% purity; Q-TOF: HRMS (ESI): *m/z* [M + H]$^+$ calcd. for $C_{26}H_{33}ClN_2O_5$: 489.2151, found: 489.2126.

4-Chlorophenoxycarbonyl-leucyl-phenylalanine methyl ester (3)

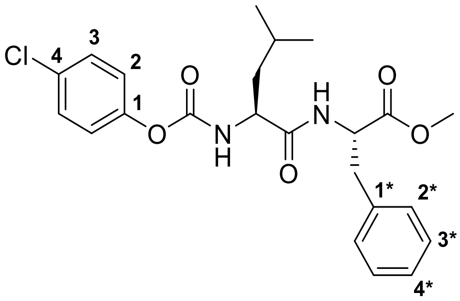

The compound was synthesized according to GP6 using **33** (0.20 g) to obtain a white solid (76 mg, 17%). mp 134–136 °C; ^{1}H NMR (500 MHz, DMSO-d_6) δ 0.88 (2 × d, ^3J = 6.6 Hz, 6H, CH(C$\underline{H}_3$)$_2$), 1.36–1.50 (m, 2H, CHC$\underline{H}_{2,Leu}$), 1.58–1.70 (m, 1H, C$\underline{H}$(CH$_3$)$_{2,Leu}$), 2.95 (dd, ^2J = 13.8 Hz, ^3J = 8.9 Hz, 1H, CHC$\underline{H}_{2,Phe}$), 3.03 (dd, ^2J = 13.8 Hz, ^3J = 5.9 Hz, 1H, CHC$\underline{H}_{2,Phe}$), 3.58 (s, 3H, OC$\underline{H}_3$), 4.04–4.14 (m, 1H, NHC$\underline{H}_{Leu}$), 4.45–4.52 (m, 1H, NHC$\underline{H}_{Phe}$), 7.07–7.11 (m, 2H, 2-$\underline{H}$), 7.14–7.22 (m, 3H, 2*-$\underline{H}$, 4*-$\underline{H}$), 7.22–7.27 (m, 2H, 3*-$\underline{H}$), 7.40–7.44 (m, 2H, 3-$\underline{H}$), 7.90 (d, ^3J = 8.4 Hz, 1H, OCON$\underline{H}$), 8.37 (d, ^3J = 7.6 Hz, 1H, N$\underline{H}$CH$_{Phe}$); ^{13}C NMR (125 MHz, DMSO-d_6) δ 21.68, 23.04, 24.28 ($\underline{C}$H($\underline{C}$H$_3$)$_2$), 36.64 (CH$\underline{C}$H$_{2,Phe}$), 40.68 (CH$\underline{C}$H$_{2,Leu}$), 51.92 (O$\underline{C}$H$_3$), 53.22, 53.61 (NH$\underline{C}$H$_{Leu}$, NH$\underline{C}$H$_{Phe}$), 123.53 ($\underline{C}$-2), 126.63 ($\underline{C}$-4*), 128.32 ($\underline{C}$-2*), 129.09 ($\underline{C}$-4), 129.18, 129.28 ($\underline{C}$-3, $\underline{C}$-3*), 137.22 ($\underline{C}$ 1*), 149.96 ($\underline{C}$-1), 153.80 (O$\underline{C}$ONH), 171.91, 172.07 (CH$\underline{C}$O); LC/MS (ESI): H_2O/MeOH, 90:10 to 0:100; *m/z* 447.2 [M + H]$^+$, 464.2 [M + NH$_4$]$^+$; 95% purity; Q-TOF: HRMS (ESI): *m/z* [M + H]$^+$ calcd. for $C_{23}H_{27}ClN_2O_5$: 447.1681, found: 447.1671.

4-Chlorophenoxycarbonyl-leucyl-phenylalanine (4)

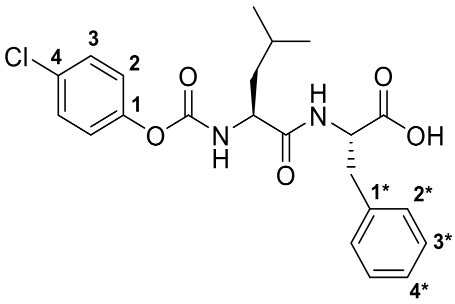

The synthesis was carried out according to GP8 using the corresponding *tert*-butyl ester (122 mg, 0.25 mmol). Column chromatography on silica gel using EtOAc as eluent afforded the product as a white solid (68 mg, 0.16 mmol, 63%). mp 168–173 °C; ^{1}H NMR (500 MHz, DMSO-d_6) δ 0.87 (2 × d, ^3J = 6.6 Hz, 6H, CH(C$\underline{H}_3$)$_{2,Leu}$), 1.38–1.49 (m, 2H, CHC$\underline{H}_{2,Leu}$), 1.60–1.65 (m, 1H, C$\underline{H}$(CH$_3$)$_{2,Leu}$), 2.92 (dd, ^2J = 13.9 Hz, ^3J = 8.8 Hz, 1H, CHC$\underline{H}_{2,Phe}$), 3.06 (dd, ^2J = 13.9 Hz, ^3J = 5.4 Hz, 1H, CHC$\underline{H}_{2,Phe}$), 4.06 (dt, ^3J = 4.8 Hz, ^3J = 9.2 Hz, 1H, NHC$\underline{H}_{Phe}$), 4.45 (dt, ^3J = 5.1 Hz, ^3J = 8.5 Hz, 1H, NHC$\underline{H}_{Leu}$), 7.09 (d, ^3J = 8.8 Hz, 2H, 2-$\underline{H}$), 7.19–7.25 (m, 5H, 2*-$\underline{H}$, 3*-$\underline{H}$, 4*-$\underline{H}$), 7.42 (d, ^3J = 8.8 Hz, 2H, 3-$\underline{H}$), 7.90 (d, ^3J = 8.5 Hz, 1H, OCON$\underline{H}$), 8.15 (d, ^3J = 7.9 Hz, 1H, N$\underline{H}$CH$_{Phe}$), 12.68 (s, 1H, COO$\underline{H}$); ^{13}C NMR (125 MHz, DMSO-d_6) δ 21.63, 23.07, 24.28 ($\underline{C}$H($\underline{C}$H$_3$)$_{2,Leu}$), 36.74 (CH$\underline{C}$H$_{2,Phe}$), 40.68 (CH$\underline{C}$H$_{2,Leu}$), 53.39 (NH$\underline{C}$H$_{Phe}$, NH$\underline{C}$H$_{Leu}$), 123.53 ($\underline{C}$-2), 126.50 ($\underline{C}$-4*), 128.24 ($\underline{C}$-2*), 129.25 ($\underline{C}$-4), 129.26 ($\underline{C}$-3,

C-3*), 137.57 (C-1*), 149.96 (C-1), 153.80 (OCONH), 171.87 (CHCO$_{Leu}$), 172.86 (COOH); LC/MS (ESI): H_2O/MeOH, 90:10 to 0:100; *m/z* 433.2 [M + H]$^+$; 96% purity; Q-TOF: HRMS (ESI): *m/z* [M + H]$^+$ calcd. for $C_{22}H_{25}ClN_2O_5$: 433.1525, found: 433.1538.

(*R*)-2-(4-Chlorophenoxycarbonylaminomethyl)-4-methylpentanoyl-phenylalanine (5)

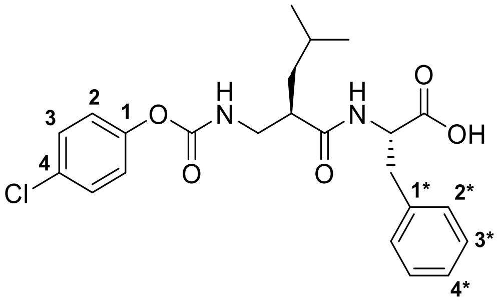

The compound was synthesized on a 2-chlorotrityl resin according to GP9 using Fmoc-APA-OH (0.37 g). The product was obtained after recrystallization from EtOAc as yellow crystals (88 mg, 39%). mp 184–186 °C; ^{1}H NMR (600 MHz, DMSO-d_6) δ 0.82 (2 × d, ^3J = 6.4 Hz, 3H, C(CH_3)$_2$), 1.08–1.15 (m, 1H, CH_2CH(CH_3)$_2$), 1.36–1.43 (m, 1H, CH_2CH(CH_3)$_2$), 1.44–1.51 (m, 1H, CH(CH_3)$_2$), 2.53–2.59 (m, 1H, NHCH_2CH), 2.93 (dd, ^2J = 14.0 Hz, ^3J = 9.3 Hz, 1H, CHC$H_{2,Phe}$), 3.01 (t, ^3J = 6.5 Hz, 2H, NHCH_2CH), 3.05 (dd, ^2J = 14.0 Hz, ^3J = 5.2 Hz, 1H, CHC$H_{2,Phe}$), 4.44–4.50 (m, 1H, NHCH_{Phe}), 7.07–7.12 (m, 2H, 2-H), 7.14–7.25 (m, 5H, 2*-H, 3*-H, 4*-H), 7.39–7.45 (m, 2H, 3-H), 7.54 (t, ^3J = 5.9 Hz, 1H, OCONH), 8.26 (d, ^3J = 8.0 Hz, 1H, NHCH$_{Phe}$), 12.60 (br s, 1H, COOH); ^{13}C NMR (150 MHz, DMSO-d_6) δ 22.52, 23.75, 25.65 (CH(CH_3)$_2$), 37.01 (CHC$H_{2,Phe}$), 39.28 (CH_2CH(CH_3)$_2$), 43.72, 44.21 (NHCH_2CH, NHCH_2CH), 53.65 (NHCH$_{Phe}$), 123.99 (C-2), 126.74 (C-4*), 128.56 (C-2*), 129.37 (C-4), 129.46, 129.55 (C-3, C-3*), 138.24 (C-1*), 150.36 (C-1), 154.44 (OCONH), 173.46 (CONH), 173.83 (COOH); LC/MS (ESI): H_2O/MeOH, 90:10 to 0:100; *m/z* 447.1 [M + H]$^+$; 97% purity; Q-TOF: HRMS (ESI): *m/z* [M + H]$^+$ calcd. for $C_{23}H_{27}ClN_2O_5$: 447.1681, found: 447.1672.

4-Chlorophenoxycarbonyl-cyclohexylglycyl-phenylalanine (6)

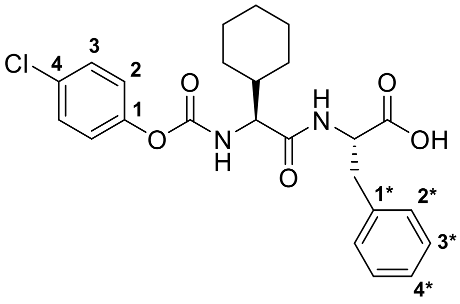

The compound was synthesized on a 2-chlorotrityl resin according to GP9 using Fmoc-Chg-OH (0.38 g). The product was purified by column chromatography on silica gel using EtOAc / MeOH (9:1) to obtain white solid (45 mg, 20%). mp > 250 °C; ^{1}H NMR (600 MHz, DMSO-d_6) δ 0.91–0.99 (m, 2H, CH(CH_2)$_5$), 1.03–1.12 (m, 3H, CH(CH_2)$_5$), 1.45–1.51 (m, 1H, CH(CH_2)$_5$), 1.54–1.66 (m, 5H, CH(CH_2)$_5$), 2.89 (dd, ^2J = 13.8 Hz, ^3J = 8.2 Hz, 1H, CHC$H_{2,Phe}$), 3.08 (dd, ^2J = 13.8 Hz, ^3J = 4.9

Hz, 1H, $CHC\underline{H}_{2,Phe}$), 3.80 (t, ^3J = 8.1 Hz, 1H, $NHC\underline{H}_{Chg}$), 4.30–4.37 (m, 1H, $NHC\underline{H}_{Phe}$), 7.11 (d, ^3J = 8.5 Hz, 2H, 2-$\underline{H}$), 7.14–7.22 (m, 5H, 2*-$\underline{H}$, 3*-$\underline{H}$, 4*-$\underline{H}$), 7.41 (d, ^3J = 8.4 Hz, 2H, 3-$\underline{H}$), 7.80–7.84 (m, 1H, $OCON\underline{H}$), 7.86 (d, ^3J = 9.1 Hz, 1H, $N\underline{H}CH_{Phe}$), one signal ($COO\underline{H}$) is not visible; ^{13}C NMR (150 MHz, DMSO-d_6) δ 21.40, 25.73, 25.79, 25.91, 28.24, 29.21 ($\underline{C}H(\underline{C}H_2)_5$)), 37.40 ($CH\underline{C}H_{2,Phe}$), 54.36 ($NH\underline{C}H_{Phe}$), 60.42 ($NH\underline{C}H_{Chg}$), 123.72 ($\underline{C}$-2), 126.16 ($\underline{C}$-4*), 128.01 ($\underline{C}$-2*), 129.15 ($\underline{C}$-4), 129.32 ($\underline{C}$-3), 129.51 ($\underline{C}$-3*), 138.40 ($\underline{C}$-1*), 150.07 ($\underline{C}$-1), 154.11 ($O\underline{C}ONH$), 170.13 ($\underline{C}ONH$), 173.76 ($\underline{C}OOH$); LC/MS (ESI): H_2O/MeOH, 90:10 to 0:100; *m/z* 459.2 $[M + H]^+$, 476.2 $[M + NH_4]^+$; 96% purity; Q-TOF: HRMS (ESI): *m/z* $[M + H]^+$ calcd. for $C_{24}H_{27}ClN_2O_5$: 459.1681, found: 459.1654.

4-Chlorophenoxycarbonyl-cyclohexylalanyl-phenylalanine *tert*-butyl ester

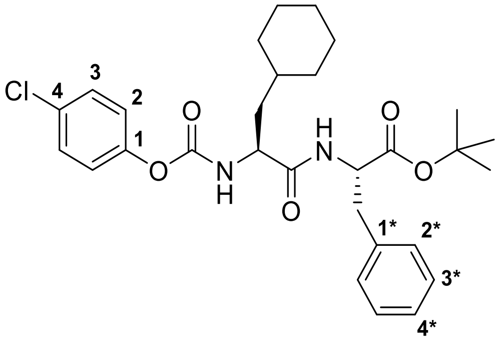

The synthesis was carried out according to GP7 using Cbz-Cha-Phe-O*t*Bu (1.02 g) for the hydrogenation. Subsequently, H-Cha-Phe-O*t*Bu (0.67 g, 1.80 mmol) was reacted with 4-chlorophenyl chloroformate (0.38 g, 2.00 mmol) to generate the desired carbamate. Column chromatography on silica gel using petrolum ether / EtOAc (4:1) as eluent afforded the product as a colorless resin (0.74 g, 1.39 mmol, 77%). ^1H NMR (500 MHz, DMSO-d_6) δ 0.84–0.93 (m, 2H, $CH(C\underline{H}_2)_5$), 1.12–1.20 (m, 4H, $C\underline{H}(C\underline{H}_2)_5$), 1.31 (s, 9H $C(C\underline{H}_3)_3$), 1.44–1.48 (m, 2H, $CH(C\underline{H}_2)_5$), 1.60–1.72 (m, 5H, $CH(C\underline{H}_2)_5$, $NHCHC\underline{H}_{2,Cha}$), 2.90–3.00 (m, 2H, $CHC\underline{H}_{2,Phe}$), 4.11 (q, ^3J = 7.8 Hz, 1H, $NHC\underline{H}_{Phe}$), 4.35 (q, ^3J = 7.4 Hz, 1H, $NHC\underline{H}_{Cha}$), 7.06–7.11 (m, 2H, 2-$\underline{H}$), 7.19–7.26 (m, 5H, 2*-$\underline{H}$, 3*-$\underline{H}$, 4*-$\underline{H}$), 7.40–7.43 (m, 2H, 3-$\underline{H}$), 7.89 (d, ^3J = 8.5 Hz, 1H, $OCON\underline{H}$), 8.24 (d, ^3J = 7.5 Hz, 1H, $N\underline{H}CH_{Phe}$); ^{13}C NMR (125 MHz, DMSO-d_6) δ 25.73, 25.88, 26.21 ($CH(\underline{C}H_2)_5$), 27.65 ($C(\underline{C}H_3)_3$), 31.92, 33.31, 33.60 ($\underline{C}H(\underline{C}H_2)_5$), 36.82 ($CH\underline{C}H_{2,Phe}$), 52.56, 54.23 ($NH\underline{C}H_{Cha}$, $NH\underline{C}H_{Phe}$), 80.71 ($\underline{C}(CH_3)_3$), 123.52 ($\underline{C}$-2), 126.56 ($\underline{C}$-4*), 128.24 ($\underline{C}$-2*), 129.09 ($\underline{C}$-4), 129.28, 129.31 ($\underline{C}$-3, $\underline{C}$-3*), 137.34 ($\underline{C}$-1*), 149.99 ($\underline{C}$-1), 153.85 ($O\underline{C}ONH$), 170.50, 172.04 ($CH\underline{C}O$), one signal ($NHCH\underline{C}H_{2,Cha}$) is obscured by the DMSO solvent signal; LC/MS (ESI): H_2O/MeCN, 90:10 to 0:100; *m/z* 529.2 $[M + H]^+$; 100% purity; Q-TOF: HRMS (ESI): *m/z* $[M + H]^+$ calcd. for $C_{29}H_{37}ClN_2O_5$: 529.2464, found: 529.2455.

4-Chlorophenoxycarbonyl-cyclohexylalanyl-phenylalanine (7)

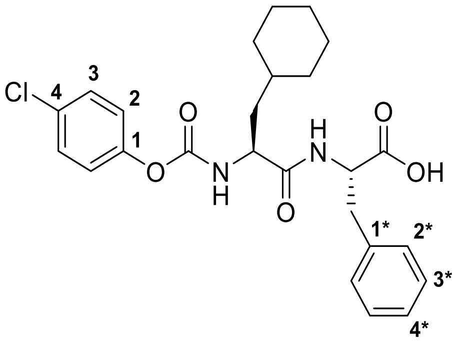

The synthesis was carried out according to GP8 using the corresponding *tert*-butyl ester (159 mg, 0.3 mmol). Column chromatography on silica gel using EtOAc as eluent afforded the product as a white solid (123 mg, 0.26 mmol, 87%). mp 158–160 °C; ^{1}H NMR (600 MHz, $CDCl_3$) δ 0.82–0.95 (m, 2H, $CH(C\underline{H}_2)_5$), 1.08–1.20 (m, 4H, $C\underline{H}(C\underline{H}_2)_5$), 1.28–1.37 (m, 1H, $CH(C\underline{H}_2)_5$), 1.48–1.54 (m, 1H, $CH(C\underline{H}_2)_5$), 1.58–1.66 (m, 4H, $CH(C\underline{H}_2)_5$, $NHCHC\underline{H}_{2,Cha}$), 1.72 (m, 1H, $NHCHC\underline{H}_{2,Cha}$), 3.06 (dd, ^2J = 13.8 Hz, ^3J = 6.1 Hz, 1H, $CHC\underline{H}_{2,Phe}$), 3.20 (dd, ^2J = 13.6 Hz, ^3J = 5.0 Hz, 1H, $CHC\underline{H}_{2,Phe}$), 4.24 (dt, ^3J = 6.5 Hz, ^3J = 7.5 Hz, 1H, $NHC\underline{H}_{Phe}$), 4.86 (dt, ^3J = 6.1 Hz, 1H, $NHC\underline{H}_{Cha}$), 5.84 (d, ^3J = 8.0 Hz, 1H, $OCON\underline{H}$), 6.58 (d, ^3J = 7.1 Hz, 1H, $N\underline{H}CH_{Phe}$), 7.02 (d, ^3J = 8.5 Hz, 2H, 2-$\underline{H}$), 7.13 (d, ^3J = 6.9 Hz, 2H, 2*-$\underline{H}$), 7.19–7.23 (m, 3H, 3*-$\underline{H}$, 4*-$\underline{H}$), 7.29 (d, ^3J = 8.4 Hz, 2H, 3-$\underline{H}$), one signal ($COO\underline{H}$) is not visible; ^{13}C NMR (150 MHz, $CDCl_3$) δ 25.97, 26.06, 26.28 ($CH(\underline{C}H_2)_5$), 32.67, 33.43, 33.89 ($\underline{C}H(\underline{C}H_2)_5$), 37.34 ($CH\underline{C}H_{2,Phe}$), 39.82 ($NHCH\underline{C}H_{2,Cha}$), 52.94, 53.15 ($NH\underline{C}H_{Cha}$, $NH\underline{C}H_{Phe}$), 122.84 ($\underline{C}$-2), 127.24 ($\underline{C}$-4*), 128.64, 129.32, 129.35 ($\underline{C}$-3, $\underline{C}$-2*, $\underline{C}$-3*), 130.81 ($\underline{C}$-4), 135.39 ($\underline{C}$-1*), 149.30 ($\underline{C}$-1), 154.16 ($O\underline{C}ONH$), 172.17 ($CH\underline{C}O_{Cha}$), 175.22 ($\underline{C}OOH$); LC/MS (ESI): H_2O/MeCN, 90:10 to 0:100; *m/z* 473.2 $[M + H]^+$; 95% purity; Q-TOF: HRMS (ESI): *m/z* $[M + H]^+$ calcd. for $C_{25}H_{29}ClN_2O_5$: 473.1838, found: 473.1833.

4-Chlorophenoxycarbonyl-leucyl-proline *tert*-butyl ester

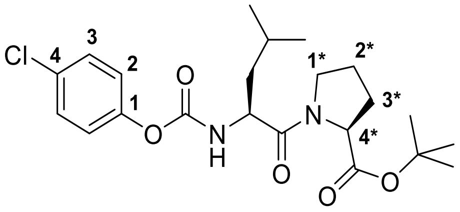

The synthesis was carried out according to GP7 using Cbz-Leu-Pro-O*t*Bu (0.84 g) for the hydrogenation. Subsequently, H-Leu-Pro-O*t*Bu (0.17 g, 0.60 mmol) was reacted with 4-chlorophenyl chloroformate (0.13 g, 0.70 mmol) to generate the desired carbamate. Column chromatography on silica gel using petrolum ether / EtOAc (4:1) as eluent afforded the product as a colorless oil (0.24 g, 0.54 mmol, 90%). ^{1}H NMR (500 MHz, DMSO-*d*$_6$) δ 0.92 (d, ^3J = 6.6 Hz, 6H, $CH(C\underline{H}_3)_{2,Leu}$), 1.37 (s, 9H, $C(C\underline{H}_3)_3$), 1.40–1.45 (m, 1H, $CHC\underline{H}_{2,Leu}$), 1.54–1.60 (m, 1H, $CHC\underline{H}_{2,Leu}$), 1.71–1.81 (m, 2H, $C\underline{H}(CH_3)_{2,Leu}$, 2*-$\underline{H}$), 1.90–1.94 (m, 2H, 2*-$\underline{H}$, 3*-$\underline{H}$), 2.11–2.18 (m, 1H, 3*-$\underline{H}$), 3.48 (dt, ^2J = 9.5 Hz, ^3J = 6.6 Hz, 1H, 1*-$\underline{H}$), 3.66 (dt, ^2J = 9.8 Hz, ^3J = 6.9 Hz, 1H, 1*-$\underline{H}$), 4.20 (dd, ^3J = 5.1 Hz, ^3J = 8.5 Hz, 1H, 4*-$\underline{H}$), 4.28–4.32 (m,

1H, $NHC\underline{H}_{Leu}$), 7.10 (d, ^3J = 8.5 Hz, 2H, 2-$\underline{H}$), 7.41 (d, ^3J = 8.8 Hz, 2H, 3-$\underline{H}$), 8.09 (d, ^3J = 7.9 Hz, 1H, $OCON\underline{H}$); ^{13}C NMR (125 MHz, DMSO-d_6) δ 21.39, 23.20, 24.30 ($\underline{C}H(\underline{C}H_3)_{2,Leu}$), 24.71 ($\underline{C}$-2*), 27.68 ($C(\underline{C}H_3)_3$), 28.62 ($\underline{C}$-3*), 46.46 ($\underline{C}$-1*), 51.16 ($NH\underline{C}H_{Leu}$), 59.57 ($\underline{C}$-4*), 80.39 ($\underline{C}(CH_3)_3$), 123.55 ($\underline{C}$-2), 129.24 ($\underline{C}$-3, $\underline{C}$-4), 149.91 ($\underline{C}$-1), 154.13 ($O\underline{C}ONH$), 170.30, 170.96 ($CH\underline{C}O$), one signal ($CH\underline{C}H_{2,Leu}$) is obscured by the DMSO solvent signal; LC/MS (ESI): H_2O/MeOH, 90:10 to 0:100; *m/z* 439.2 $[M + H]^+$; 90% purity; Q-TOF: HRMS (ESI): *m/z* $[M + H]^+$ calcd. for $C_{22}H_{31}ClN_2O_5$: 439.1994, found: 439.1997.

4-Chlorophenoxycarbonyl-leucyl-proline methyl ester

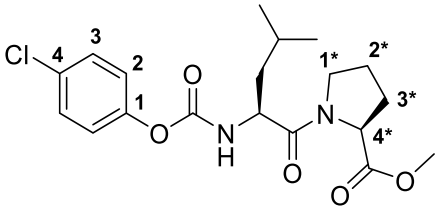

The compound was synthesized according to GP6 using compound **32** (0.18 g) to obtain a white solid (75 mg, 38%). mp 98–100 °C; ^{1}H NMR (600 MHz, $CDCl_3$) δ 0.98 (2 × d, ^3J = 6.6 Hz, 6H, $CH(C\underline{H}_3)_{2,Leu}$), 1.54–1.61 (m, 2H, $CHC\underline{H}_{2,Leu}$), 1.78–1.84 (m, 1H, $C\underline{H}(CH_3)_{2,Leu}$), 1.95–2.02 (m, 2H, 2*-$\underline{H}$), 2.02–2.09 (m, 1H, 3*-$\underline{H}$), 2.17–2.24 (m, 1H, 3*-$\underline{H}$), 3.57–3.62 (m, 1H, 1*-$\underline{H}$), 3.71 (s, 3H, $OC\underline{H}_3$), 3.73–3.76 (m, 1H, 1*-$\underline{H}$), 4.52–4.58 (m, 2H, $NHC\underline{H}_{Leu}$, 4*-$\underline{H}$), 5.72 (d, ^3J = 8.9 Hz, 1H, $OCON\underline{H}$), 7.01–7.05 (m, 2H, 2-$\underline{H}$), 7.25–7.30 (m, 2H, 3-$\underline{H}$); ^{13}C NMR (150 MHz, $CDCl_3$) δ 21.70, 23.38, 24.57 ($\underline{C}H(\underline{C}H_3)_2$), 24.87 ($\underline{C}$-2*), 28.94 ($\underline{C}$-3*), 41.87 ($CH\underline{C}H_{2,Leu}$), 46.79 ($\underline{C}$-1*), 51.09 ($NH\underline{C}H_{Leu}$), 52.28 ($O\underline{C}H_3$), 58.74 ($\underline{C}$-4*), 122.94 ($\underline{C}$-2), 129.25 ($\underline{C}$-3), 130.67 ($\underline{C}$-4), 149.40 ($\underline{C}$-1), 154.27 ($O\underline{C}ONH$), 171.13 ($\underline{C}ON$), 172.29 ($\underline{C}OOCH_3$); LC/MS (ESI): H_2O/MeOH, 90:10 to 0:100; *m/z* 397.2 $[M + H]^+$; 99% purity; Q-TOF: HRMS (ESI): *m/z* $[M + H]^+$ calcd. for $C_{19}H_{25}ClN_2O_5$: 397.1525, found: 397.1504.

4-Chlorophenoxycarbonyl-leucyl-proline (8)

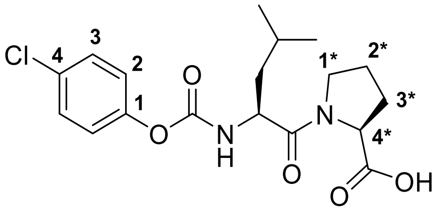

The compound was synthesized on a 2-chlorotrityl resin according to GP9 using Fmoc-Leu-OH (0.35 g). Column chromatography on silica gel using EtOAc afforded the product as a white solid (77 mg, 40%). The material was dissolved in water (20 mL) and subjected to lyophilization. mp 82–84 °C.

The synthesis was carried out as well according to GP8 using the corresponding *tert*-butyl ester (53 mg, 0.12 mmol). Column chromatography on silica gel using EtOAc as eluent afforded the product as a white solid (42 mg, 0.11 mmol, 91%). mp 110–112 °C; ^{1}H NMR (500 MHz, DMSO-d_6) δ 0.92 (2 × d, ^3J = 6.6 Hz, 6H, $CH(C\underline{H}_3)_{2,Leu}$), 1.40–1.46 (m, 1H, $CHC\underline{H}_{2,Leu}$), 1.51–1.57 (m, 1H, $CHC\underline{H}_{2,Leu}$), 1.70–

1.76 (m, 1H, CH$(CH_3)_{2,Leu}$), 1.78–1.85 (m, 1H, 2*-H), 1.89–1.94 (m, 2H, 2*-H, 3*-H), 2.11–2.18 (m, 1H, 3*-H), 3.47–3.52 (m, 1H, 1*-H), 3.67 (dt, ^2J = 9.5 Hz, ^3J = 6.9 Hz, 1H, 1*-H), 4.26 (dd, ^3J = 4.8 Hz, ^3J = 8.8 Hz, 2H, 4*-H), 4.28–4.33 (m, 1H, $NHCH_{Leu}$), 7.11 (d, ^3J = 8.5 Hz, 2H, 2-H), 7.41 (d, ^3J = 8.5 Hz, 2H, 3-H), 8.08 (d, ^3J = 7.6 Hz, 1H, OCONH); ^{13}C NMR (125 MHz, DMSO-d_6) δ 21.54, 23.23, 24.26 ($CH(CH_3)_{2,Leu}$), 24.75 (C-2*), 28.66 (C-3*), 46.52 (C-1*), 51.14 ($NHCH_{Leu}$), 58.69 (C-4*), 123.61 (C-2), 129.29 (C-3, C-4), 149.95 (C-1), 154.14 (OCONH), 170.35 ($CHCO_{Leu}$), 173.29 (COOH), one signal ($CHCH_{2,Leu}$) is obscured by the DMSO solvent signal; LC/MS (ESI): H_2O/MeOH, 90:10 to 0:100; *m/z* 383.2 $[M + H]^+$; 95% purity; Q-TOF: HRMS (ESI): *m/z* $[M + H]^+$ calcd. for $C_{18}H_{23}ClN_2O_5$: 383.1368, found: 383.1369.

4-Chlorophenoxycarbonyl-alanyl-proline *tert*-butyl ester

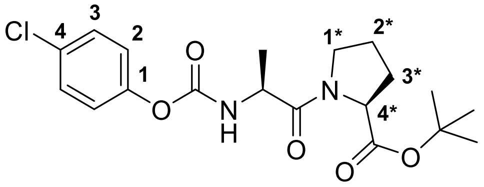

The synthesis was carried out according to GP7 using Cbz-Ala-Pro-O*t*Bu (0.49 g, 1.30 mmol) for the hydrogenation. Subsequently, H-Ala-Pro-O*t*Bu (0.24 g, 1.00 mmol), TEA (0.11 g, 1.10 mmol) and 4-chlorophenyl chloroformate (0.21 g, 1.10 mmol) were used for the reaction. Column chromatography on silica gel using petrolum ether / EtOAc (3:2) as eluent afforded the desired carbamate as a white solid (0.27 g, 0.68 mmol, 68%). mp 120–124 °C; ^{1}H NMR (500 MHz, DMSO-d_6) δ 1.27 (d, ^3J = 6.9 Hz, 3H, $CHH_{3,Ala}$), 1.37 (s, 9H, $C(CH_3)_3$), 1.74–1.81 (m, 1H, 2*-H), 1.88–1.93 (m, 2H, 2*-H, 3*-H), 2.10–2.18 (m, 1H, 3*-H), 3.51 (dt, ^2J = 9.8 Hz, ^3J = 6.8 Hz, 1H, 1*-H), 3.62 (dt, ^2J = 9.8 Hz, ^3J = 6.9 Hz, 1H, 1*-H), 4.19 (dd, ^3J = 4.7 Hz, ^3J = 8.8 Hz, 1H, 4*-H), 4.35 (quint, ^3J = 7.1 Hz, 1H, $NHCH_{Ala}$), 7.10 (d, ^3J = 8.9 Hz, 2H, 2-H), 7.41 (d, ^3J = 8.8 Hz, 2H, 3-H), 8.07 (d, ^3J = 7.3 Hz, 1H, OCONH); ^{13}C NMR (125 MHz, DMSO-d_6) δ 16.66 ($CHCH_{3,Ala}$), 24.72 (C-2*), 27.72 ($C(CH_3)_3$), 28.63 (C-3*), 46.45 (C-1*), 48.43 ($NHCH_{Ala}$), 59.53 (C-4*), 80.37 ($C(CH_3)_3$), 123.61 (C-2), 129.27 (C-3, C-4), 149.91 (C-1), 153.73 (OCONH), 170.52, 171.98 (CHCO); LC/MS (ESI): H_2O/MeOH, 90:10 to 0:100; *m/z* 397.2 $[M + H]^+$; 96% purity; Q-TOF: HRMS (ESI): *m/z* $[M + H]^+$ calcd. for $C_{19}H_{25}ClN_2O_5$: 397.1525, found: 397.1519.

4-Chlorophenoxycarbonyl-alanyl-proline

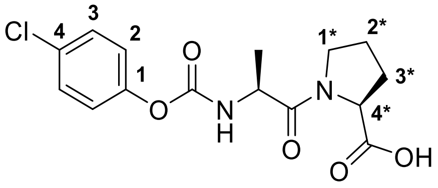

The synthesis was carried out according to GP8 using the corresponding *tert*-butyl ester (0.12 g, 0.30 mmol). Column chromatography on silica gel afforded the product as a white solid (85 mg, 0.25 mmol, 83%). mp 143–147 °C; ^{1}H NMR (500 MHz, DMSO-d_6) δ 1.25 (d, ^3J = 6.9 Hz, 3H, $CHH_{3,Ala}$),

1.80–1.86 (m, 1H, 2*-$\underline{H}$), 1.88–1.94 (m, 2H, 2*-$\underline{H}$, 3*-$\underline{H}$), 2.10–2.17 (m, 1H, 3*-$\underline{H}$), 3.53 (dt, ^{2}J = 9.8 Hz, ^{3}J = 6.8 Hz, 1H, 1*-$\underline{H}$), 3.63 (dt, ^{2}J = 10.1 Hz, ^{3}J = 7.0 Hz, 1H, 1*-$\underline{H}$), 4.26 (dd, ^{3}J = 4.6 Hz, ^{3}J = 8.7 Hz, 1H, 4*-$\underline{H}$), 4.35 (quint, ^{3}J = 7.1 Hz, 1H, NHC$\underline{H}_{Ala}$), 7.09–7.12 (m, 2H, 2-$\underline{H}$), 7.39–7.42 (m, 2H, 3-$\underline{H}$), 8.06 (d, ^{3}J = 7.6 Hz, 1H, OCON$\underline{H}$), 12.43 (s, 1H, COO$\underline{H}$); ^{13}C NMR (125 MHz, DMSO-*d*$_6$) δ 16.70 (CH$\underline{C}H_{3,Ala}$), 24.72 ($\underline{C}$-2*), 28.65 ($\underline{C}$-3*), 46.46 ($\underline{C}$-1*), 48.41 (NH$\underline{C}H_{Ala}$), 58.67 ($\underline{C}$-4*), 123.62 ($\underline{C}$-2), 129.28 ($\underline{C}$-3, $\underline{C}$-4), 149.93 ($\underline{C}$-1), 153.72 (O$\underline{C}$ONH), 170.52 (CH$\underline{C}$O), 173.30 ($\underline{C}$OOH); LC/MS (ESI): H_2O/MeOH, 90:10 to 0:100; *m/z* 341.3 $[M + H]^+$; 100% purity; Q-TOF: HRMS (ESI): *m/z* $[M + H]^+$ calcd. for $C_{15}H_{17}ClN_2O_5$: 341.0899, found: 341.0902.

4-Chlorophenoxycarbonyl-alanyl-phenylalanine *tert*-butyl ester

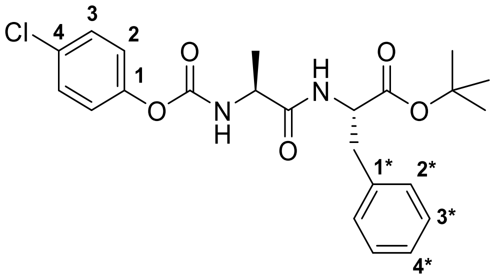

The synthesis was carried out according to GP7 using Cbz-Ala-Phe-O*t*Bu (0.60 g, 1.40 mmol) for the hydrogenation. Subsequently, H-Ala-Phe-O*t*Bu (0.29 g, 1.00 mmol), TEA (0.11 g, 1.10 mmol) and 4-chlorophenyl chloroformate (0.21 g, 1.10 mmol) were used for the reaction. Column chromatography on silica gel using petrolum ether / EtOAc (7:3) as eluent afforded the desired carbamate as a colorless oil (0.30 g, 0.66 mmol, 67%). ^{1}H NMR (600 MHz, $CDCl_3$) δ 1.38–1.43 (m, 12H, C(C$\underline{H}_3$)$_3$, CHC$\underline{H}_{3,Ala}$), 3.05–3.12 (m, 2H, CHC$\underline{H}_{2,Phe}$), 4.23 (quint, ^{3}J = 7.1 Hz, 1H, NHC$\underline{H}_{Ala}$), 4.72 (dt, ^{3}J = 5.9 Hz, ^{3}J = 7.7 Hz, 1H, NHC$\underline{H}_{Phe}$), 5.68 (d, ^{3}J = 7.4 Hz, 1H, OCON$\underline{H}$), 6.30 (d, ^{3}J = 7.7 Hz, 1H, N$\underline{H}$CH$_{Phe}$), 7.04 (d, ^{3}J = 8.7 Hz, 2H, 2-$\underline{H}$), 7.12 (d, ^{3}J = 7.1 Hz, 2H, 2*-$\underline{H}$), 7.19–7.27 (m, 3H, 3*-$\underline{H}$, 4*-$\underline{H}$), 7.29 (d, ^{3}J = 8.8 Hz, 2H, 3-$\underline{H}$); ^{13}C NMR (150 MHz, $CDCl_3$) δ 18.82 (CH$\underline{C}H_{3,Ala}$), 27.95 (C($\underline{C}H_3$)$_3$), 37.86 (CH$\underline{C}H_{2,Phe}$), 50.58, 53.61 (NH$\underline{C}H_{Ala}$, NH$\underline{C}H_{Phe}$), 82.68 ($\underline{C}$(CH$_3$)$_3$), 122.89 ($\underline{C}$-2), 127.08 ($\underline{C}$-4*), 128.45, 129.30, 129.48 ($\underline{C}$-3, $\underline{C}$-2*, $\underline{C}$-3*), 130.74 ($\underline{C}$-4), 135.82 ($\underline{C}$-1*), 149.33 ($\underline{C}$-1), 153.62 (O$\underline{C}$ONH), 170.16, 171.14 (CH$\underline{C}$O); LC/MS (ESI): H_2O/MeOH, 90:10 to 0:100; *m/z* 447.3 $[M + H]^+$; 100% purity; Q-TOF: HRMS (ESI): *m/z* $[M + H]^+$ calcd. for $C_{23}H_{27}ClN_2O_5$: 447.1681, found: 447.1676.

4-Chlorophenoxycarbonyl-alanyl-phenylalanine

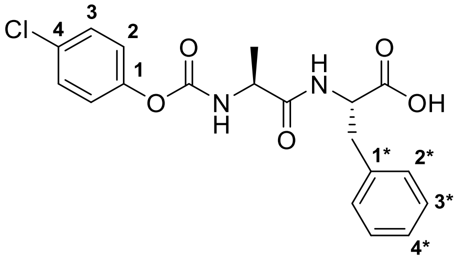

The synthesis was carried out according to GP8 using the corresponding *tert*-butyl ester (0.22 g, 0.50 mmol). Column chromatography on silica gel was not required to obtain the product as a white solid (0.18 g, 0.47 mmol, 96%). mp 169–174 °C; ^{1}H NMR (500 MHz, DMSO-d_6) δ 1.23 (d, ^{3}J = 7.0 Hz, 3H, $CHH_{3,Ala}$), 2.92 (dd, ^{2}J = 13.9 Hz, ^{3}J = 8.5 Hz, 1H, $CHH_{2,Phe}$), 3.05 (dd, ^{2}J = 13.9 Hz, ^{3}J = 5.1 Hz, 1H, $CHH_{2,Phe}$), 4.10 (quint, ^{3}J = 7.3 Hz, 1H, $NHCH_{Ala}$), 4.41–4.46 (m, 1H, $NHCH_{Phe}$), 7.10 (d, ^{3}J = 8.9 Hz, 2H, 2-H), 7.17–7.26 (m, 5H, 2*-H, 3*-H, 4*-H), 7.41 (d, ^{3}J = 8.9 Hz, 2H, 3-H), 7.93 (d, ^{3}J = 7.6 Hz, 1H, OCONH), 8.12 (d, ^{3}J = 7.9 Hz, 1H, $NHCH_{Phe}$); ^{13}C NMR (125 MHz, DMSO-d_6) δ 18.14 ($CHCH_{3,Ala}$), 36.75 ($CHCH_{2,Phe}$), 50.21, 53.50 ($NHCH_{Ala}$, $NHCH_{Phe}$), 123.59 (C-2), 126.53 (C-4), 129.13 (C-4*), 128.27, 129.25, 129.27 (C-3, C-2*, C-3*), 137.52 (C-1*), 149.92 (C-1), 153.55 (OCONH), 172.12, 172.84 (CHCO); LC/MS (ESI): H_2O/MeOH, 90:10 to 0:100; *m/z* 391.2 $[M + H]^+$; 99% purity; Q-TOF: HRMS (ESI): *m/z* $[M + H]^+$ calcd. for $C_{19}H_{19}ClN_2O_5$: 391.1055, found: 391.1057.

4-Chlorophenoxycarbonyl-alanyl-leucine *tert*-butyl ester

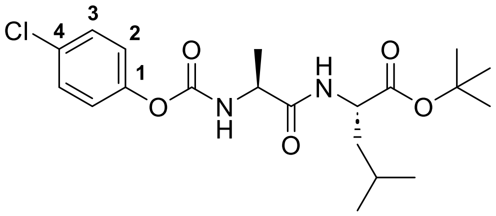

The synthesis was carried out according to GP7 using Cbz-Ala-Leu-O*t*Bu (0.79 g) for the hydrogenation. Subsequently, H-Ala-Leu-O*t*Bu (0.52 g, 2.00 mmol), TEA (0.20 g, 2.20 mmol) and 4-chlorophenyl chloroformate (0.42 g, 2.20 mmol) were used for the reaction. Column chromatography on silica gel using petrolum ether / EtOAc (7:3) as eluent afforded the desired carbamate as a colorless semisolid (0.70 g, 1.70 mmol, 85%). ^{1}H NMR (500 MHz, DMSO-d_6) δ 0.84 (2 × d, ^{3}J = 7.4 Hz, 6H, $CH(CH_3)_{2,Leu}$), 1.27 (d, ^{3}J = 6.9 Hz, 3H, $CHCH_{3,Ala}$), 1.35 (s, 9H, $C(CH_3)_3$), 1.37–1.45 (m, 1H, $CHCH_{2,Leu}$), 1.65–1.72 (m, 1H, $CHCH_{2,Leu}$), 1.97–2.05 (m, 1H, $CH(CH_3)_{2,Leu}$), 4.14 (q, ^{3}J = 6.9 Hz, 1H, $NHCH_{Ala}$), 4.41 (dd, *J* = 4.4, 11.3 Hz, 1H, $NHCH_{Leu}$), 6.72–6.78 (m, 2H, 2-H), 7.14–7.20 (m, 2H, 3-H), 8.31 (s, 1H, OCONH), 9.63 (s, 1H, $NHCH_{Leu}$); ^{13}C NMR (125 MHz, DMSO-d_6) δ 17.59 ($CHCH_{3,Ala}$), 20.99, 23.19, 24.59 ($CH(CH_3)_{2,Leu}$), 27.64 ($C(CH_3)_3$), 36.43 ($CHCH_{2,Leu}$), 50.67, 51.77 ($NHCH_{Ala}$, $NHCH_{Leu}$), 81.44 ($C(CH_3)_3$), 117.06 (C-2), 122.45 (C-4), 129.25 (C-3), 155.95 (C-1), 156.74 (OCONH), 168.39, 174.82 (CHCO), LC/MS (ESI): H_2O/MeCN, 90:10 to 0:100; *m/z* 356.8 $[M - C_4H_9]^+$; 95% purity; $C_{20}H_{29}ClN_2O_5$.

4-Chlorophenoxycarbonyl-alanyl-leucine

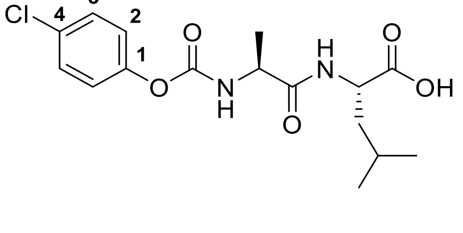

The synthesis was carried out according to GP8 using the corresponding *tert*-butyl ester (0.25 g, 0.60 mmol). Column chromatography on silica gel was not required to obtain the product as a colorless oil (0.20 g, 0.56 mmol, 93%). ^{1}H NMR (500 MHz, DMSO-d_6) δ 0.86 (2 × d, ^3J = 6.6 Hz, 6H, CH(C$\underline{H}_3$)$_{2,Leu}$), 1.27 (d, ^3J = 6.9 Hz, 3H, CHC$\underline{H}_{3,Ala}$), 1.50–1.54 (m, 2H, CHC$\underline{H}_{2,Leu}$), 1.59–1.67 (m, 1H, C$\underline{H}$(CH$_3$)$_{2,Leu}$), 4.11 (quint, ^3J = 7.3 Hz, 1H, NHC$\underline{H}_{Ala}$), 4.20–4.25 (m, 1H, NHC$\underline{H}_{Leu}$), 6.74–7.12 (d, ^3J = 8.8 Hz, 2H, 2-$\underline{H}$), 7.41 (d, ^3J = 8.8 Hz, 2H, 3-$\underline{H}$), 7.94 (d, ^3J = 7.6 Hz, 1H, OCON$\underline{H}$), 8.07 (d, ^3J = 7.9 Hz, 1H, N$\underline{H}$CH$_{Leu}$); ^{13}C NMR (125 MHz, DMSO-d_6) δ 18.11 (CH$\underline{C}$H$_{3,Ala}$), 21.49, 22.95, 24.38 ($\underline{C}$H($\underline{C}$H$_3$)$_{2,Leu}$), 50.12, 50.34 (NH$\underline{C}$H$_{Ala}$, NH$\underline{C}$H$_{Leu}$), 123.60 ($\underline{C}$-2), 129.11 ($\underline{C}$-4), 129.26 ($\underline{C}$-3), 149.97 ($\underline{C}$-1), 153.60 (O$\underline{C}$ONH), 172.19, 174.05 (CH$\underline{C}$O), one signal (CH$\underline{C}$H$_{2,Leu}$) is obscured by the DMSO solvent signal; LC/MS (ESI): H_2O/MeOH, 90:10 to 0:100; *m/z* 357.2 [M + H]$^+$; 98% purity; Q-TOF: HRMS (ESI): *m/z* [M + H]$^+$ calcd. for $C_{16}H_{21}ClN_2O_5$: 357.1212, found: 357.1207.

4-Chlorophenoxycarbonyl-alanyl-glycine *tert*-butyl ester

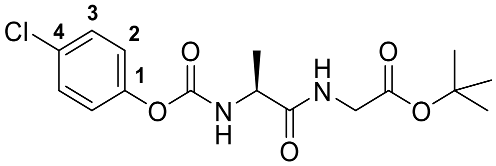

The synthesis was carried out according to GP7 using Cbz-Ala-Gly-O*t*Bu (0.27 g, 0.80 mmol) for the hydrogenation. Subsequently, H-Ala-Gly-O*t*Bu (0.12 g, 0.60 mmol), TEA (71 mg, 0.70 mmol) and 4-chlorophenyl chloroformate (0.13 g, 0.70 mmol) were used for the reaction. Column chromatography on silica gel using petrolum ether / EtOAc (1:1) as eluent afforded the desired carbamate as a white solid (0.14 g, 0.39 mmol, 65%). mp 105–110 °C; ^{1}H NMR (600 MHz, CDCl$_3$) δ 1.44–1.47 (m, 12H, C(C$\underline{H}_3$)$_3$, CHC$\underline{H}_{3,Ala}$), 3.92 (d, ^3J = 5.1 Hz, 2H, NHC$\underline{H}_{2,Gly}$), 4.31 (quint, ^3J = 7.2 Hz, 1H, NHC$\underline{H}_{Ala}$), 5.74 (d, ^3J = 7.6 Hz, 1H, OCON$\underline{H}$), 6.40–6.45 (m, 1H, N$\underline{H}$CH$_{2,Gly}$), 7.03–7.08 (m, 2H, 2-$\underline{H}$), 7.26–7.30 (m, 2H, 3-$\underline{H}$); ^{13}C NMR (150 MHz, CDCl$_3$) δ 18.72 (CH$\underline{C}$H$_{3,Ala}$), 28.02 (C($\underline{C}$H$_3$)$_3$), 42.03 (NH$\underline{C}$H$_{2,Gly}$), 50.52 (NH$\underline{C}$H$_{Ala}$), 82.62 ($\underline{C}$(CH$_3$)$_3$), 122.92 ($\underline{C}$-2), 129.31 ($\underline{C}$ 3), 130.79 ($\underline{C}$-4), 149.30 ($\underline{C}$-1), 153.84 (O$\underline{C}$ONH), 168.64, 171.80 (CH$\underline{C}$O, CH$_2\underline{C}$O); LC/MS (ESI): H_2O/MeOH, 90:10 to 0:100; *m/z* 357.3 [M + H]$^+$; 98% purity; Q-TOF: HRMS (ESI): *m/z* [M + H]$^+$ calcd. for $C_{16}H_{21}ClN_2O_5$: 357.1212, found: 357.1206.

4-Chlorophenoxycarbonyl-alanyl-glycine

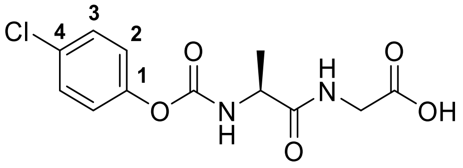

The synthesis was carried out according to GP8 using the corresponding *tert*-butyl ester (0.14 g, 0.40 mmol). Column chromatography on silica gel afforded the product as a white solid (102 mg, 0.34 mmol, 85%). mp 165–168 °C; ^{1}H NMR (500 MHz, DMSO-d_6) δ 1.28 (d, ^3J = 7.3 Hz, 3H, CHC$\underline{H}_{3,Ala}$),

3.73 (dd, ^2J = 17.5 Hz, ^3J = 6.0 Hz, 1H, NHC$\underline{H}_{2,Gly}$), 3.80 (dd, ^2J = 17.3 Hz, ^3J = 6.0 Hz, 1H, NHC$\underline{H}_{2,Gly}$), 4.11 (quint, ^3J = 7.3 Hz, 1H, NHC$\underline{H}_{Ala}$), 7.12–7.15 (m, 2H, 2-$\underline{H}$), 7.39–7.43 (m, 2H, 3-$\underline{H}$), 8.01 (d, ^3J = 7.9 Hz, 1H, OCON$\underline{H}$), 8.21 (t, ^3J = 5.7 Hz, 1H, N$\underline{H}$CH$_{2,Gly}$), 12.53 (br s, 1H, COO$\underline{H}$); ^{13}C NMR (125 MHz, DMSO-d_6) δ 18.19 (CH$\underline{C}$H$_{3,Ala}$), 40.82 (NH$\underline{C}$H$_{2,Gly}$), 50.26 (NH$\underline{C}$H$_{Ala}$), 123.69 ($\underline{C}$-2), 129.27 ($\underline{C}$-3, $\underline{C}$-4), 149.99 ($\underline{C}$-1), 153.73 (O$\underline{C}$ONH), 171.26, 172.50 (CH$\underline{C}$O, CH$_2\underline{C}$O); LC/MS (ESI): H_2O/MeOH, 90:10 to 0:100; *m/z* 318.1 [M + NH_4]$^+$; 99% purity; Q-TOF: HRMS (ESI): *m/z* [M + H]$^+$ calcd. for $C_{12}H_{13}ClN_2O_5$: 301.0586, found: 301.0584.

4-Chlorophenoxycarbonyl-alanyl-asparagine *tert*-butyl ester

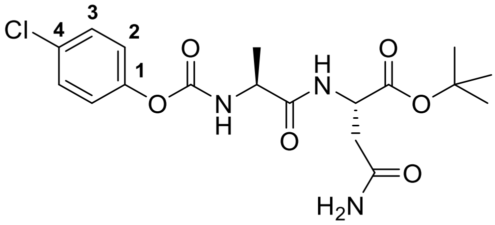

The synthesis was carried out according to GP7 using Cbz-Ala-Asn-O*t*Bu (0.63 g, 1.60 mmol) for the hydrogenation. Subsequently, H-Ala-Asn-O*t*Bu (0.31 g, 1.20 mmol), TEA (0.13 g, 1.30 mmol) and 4-chlorophenyl chloroformate (0.25 g, 1.30 mmol) were used for the reaction. Column chromatography on silica gel using petrolum ether / EtOAc (3:7) as eluent afforded the desired carbamate as a white solid (0.17 g, 0.41 mmol, 34%). mp 164–166 °C; ^{1}H NMR (600 MHz, $CDCl_3$) δ 1.44 (s, 12H, C(C$\underline{H}_3$)$_3$, CHC$\underline{H}_{3,Ala}$), 2.70 (dd, ^2J = 15.8 Hz, ^3J = 4.1 Hz, 1H, CHC$\underline{H}_{2,Asn}$), 2.88 (dd, ^2J = 16.1 Hz, ^3J = 4.7 Hz, 1H, CHC$\underline{H}_{2,Asn}$), 4.31 (quint, ^3J = 6.8 Hz, 1H, NHC$\underline{H}_{Ala}$), 4.66–4.70 (m, 1H, NHC$\underline{H}_{Asn}$), 5.69 (s, 1H, CON$\underline{H}_2$), 5.77 (s, 1H, CON$\underline{H}_2$), 5.93 (d, ^3J = 7.3 Hz, 1H, OCON$\underline{H}$), 7.04 (d, ^3J = 8.5 Hz, 2H, 2-$\underline{H}$), 7.27 (d, ^3J = 8.3 Hz, 3H, 3-$\underline{H}$, N$\underline{H}$CH$_{Asn}$); ^{13}C NMR (150 MHz, $CDCl_3$) δ 18.97 (CH$\underline{C}$H$_{3,Ala}$), 27.87 (C($\underline{C}$H$_3$)$_3$), 37.00 (CH$\underline{C}$H$_{2,Asn}$), 49.42, 50.63 (NH$\underline{C}$H$_{Ala}$, NH$\underline{C}$H$_{Asn}$), 82.68 ($\underline{C}$(CH$_3$)$_3$), 122.96 ($\underline{C}$-2), 129.29 ($\underline{C}$-3), 130.73 ($\underline{C}$-4), 149.33 ($\underline{C}$-1), 153.75 (O$\underline{C}$ONH), 169.54, 171.93 (CH$\underline{C}$O, CH$_2\underline{C}$O); LC/MS (ESI): H_2O/MeOH, 90:10 to 0:100; *m/z* 414.1 [M + H]$^+$; 97% purity; Q-TOF: HRMS (ESI): *m/z* [M + H]$^+$ calcd. for $C_{18}H_{24}ClN_3O_6$: 414.1426, found: 414.1421.

4-Chlorophenoxycarbonyl-alanyl-asparagine

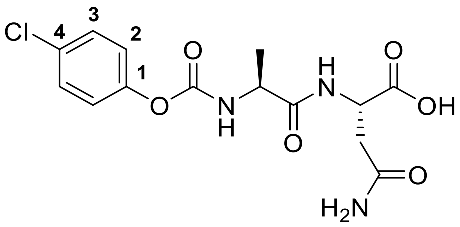

The synthesis was carried out according to GP8 using the corresponding *tert*-butyl ester (0.12 g, 0.30 mmol). Column chromatography on silica gel afforded the product as a white solid (69 mg, 0.19 mmol, 64%). mp 135–139 °C; ^{1}H NMR (500 MHz, DMSO-d_6) δ 1.26 (d, ^3J = 7.3 Hz, 3H, CHC$\underline{H}_{3,Ala}$), 2.47–2.57 (m, 2H, CHC$\underline{H}_{2,Asn}$), 4.10 (quint, ^3J = 7.3 Hz, 1H, NHC$\underline{H}_{Ala}$), 4.51 (q, ^3J = 6.6 Hz, 1H,

NHCH$_{Asn}$), 6.89 (s, 1H, CONH$_2$), 7.13 (d, ^3J = 8.8 Hz, 2H, 2-H), 7.35 (s, 1H, CONH$_2$), 7.41 (d, ^3J = 8.8 Hz, 2H, 3-H), 8.02 (d, ^3J = 7.9 Hz, 1H, OCONH), 8.09 (d, ^3J = 8.3 Hz, 1H, NHCH$_{Asn}$); ^{13}C NMR (125 MHz, DMSO-d_6) δ 18.08 (CHCH$_{3,Ala}$), 36.72 (CHCH$_{2,Asn}$), 48.82, 50.23 (NHCH$_{Ala}$, NHCH$_{Asn}$), 123.71 (C-2), 129.18 (C-4), 129.28 (C-3), 149.98 (C-1), 153.69 (OCONH), 171.42, 171.93, 172.78 (CHCO, CH$_2$CO); LC/MS (ESI): H_2O/MeOH, 90:10 to 0:100; *m/z* 358.2 [M + H]$^+$; 99% purity; Q-TOF: HRMS (ESI): *m/z* [M + H]$^+$ calcd. for $C_{14}H_{17}ClN_3O_6$: 358.0800, found: 358.0799.

4-Chlorophenoxycarbonyl-glycyl-proline

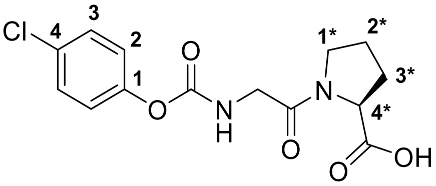

The compound was synthesized on a 2-chlorotrityl resin according to GP9 using Fmoc-Gly-OH (0.30 g). The product was purified by column chromatography on silica gel using EtOAc / MeOH (9:1) to obtain a colorless oil (92 mg, 56%). ^{1}H NMR (500 MHz, DMSO-d_6) δ 1.82–1.86 (m, 1H, 2*-H), 1.88–1.95 (m, 2H, 2*-H, 3*-H), 2.06–2.17 (m, 1H, 3*-H), 3.81–3.98 (m, 2H, CH$_{2,Gly}$), 4.25 (dd, ^3J = 3.7 Hz, ^3J = 8.9 Hz, 1H, 4*-H), 7.10–7.15 (m, 2H, 2-H), 7.40–7.44 (m, 2H, 3-H), 7.90 (t, ^3J = 6.0 Hz, 1H, OCONH), two signals (1*-H) are obscured by the water peak, one signal (COOH) is not visible; ^{13}C NMR (125 MHz, DMSO-d_6) δ 24.51 (C-2*), 28.78 (C-3*), 42.90 (CH$_{2,Gly}$), 45.63 (C-1*), 58.81 (C-4*), 123.60 (C-2), 129.16 (C-4), 129.34 (C-3), 150.06 (C-1), 154.62 (OCONH), 166.83 (CON), 173.42 (COOH); LC/MS (ESI): H_2O/MeOH, 90:10 to 0:100; *m/z* 326.9 [M + H]$^+$; 99% purity; Q-TOF: HRMS (ESI): *m/z* [M + H]$^+$ calcd. for $C_{14}H_{15}ClN_2O_5$: 327.0742, found: 327.0743.

4-Chlorophenoxycarbonyl-isoleucyl-proline

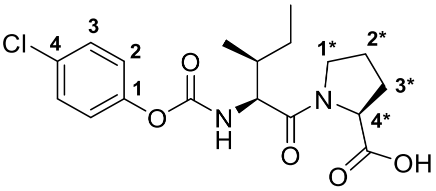

The compound was synthesized on a 2-chlorotrityl resin according to GP9 using Fmoc-Ile-OH (0.35 g). The product was obtained after recrystallization from EtOAc as a white solid (90 mg, 47%). mp 117–118 °C; ^{1}H NMR (600 MHz, DMSO-d_6) δ 0.85 (t, ^3J = 7.3 Hz, 3H, CH$_2$CH$_3$), 0.93 (d, ^3J = 6.6 Hz, 3H, CHCH$_3$), 1.15–1.19 (m, 1H, CH$_2$CH$_3$), 1.57–1.60 (m, 1H, CH$_2$CH$_3$), 1.79–1.92 (m, 4H, CHCH$_2$CH$_3$, 2*-H, 3*-H), 2.11–2.16 (m, 1H, 3*-H), 3.56–3.60 (m, 1H, 1*-H), 3.72–3.76 (m, 1H, 1*-H), 4.11 (t, ^3J = 8.8 Hz, 1H, 4*-H), 4.25–4.27 (m, 1H, NHCH$_{Ile}$), 7.11 (d, ^3J = 8.8 Hz, 2H, 2-H), 7.40 (d, ^3J = 8.8 Hz, 2H, 3-H), 8.11 (d, ^3J = 7.0 Hz, 1H, OCONH), 12.43 (br s, 1H, COOH); ^{13}C NMR (150 MHz, DMSO-d_6) δ 10.80 (CH$_2$CH$_3$), 14.81 (CHCH$_3$), 24.45 (CH$_2$CH$_3$), 24.69 (C-2*), 28.88 (C-3*), 36.11 (CHCH$_3$), 47.06 (C-1*), 56.86 (C-4*), 58.73 (NHCH$_{Ile}$), 123.68 (C-2), 129.17 (C-4), 129.31 (C-

3), 149.96 (C-1), 154.26 (OCONH), 170.10 (CON), 173.37 (COOH); LC/MS (ESI): H_2O/MeOH, 90:10 to 0:100; *m/z* 383.2 $[M + H]^+$; 99% purity; Q-TOF: HRMS (ESI): *m/z* $[M + H]^+$ calcd. for $C_{18}H_{23}ClN_2O_5$: 383.1368, found: 383.1362.

4-Chlorophenoxycarbonyl-(β-*tert*-butyl)aspartyl-proline

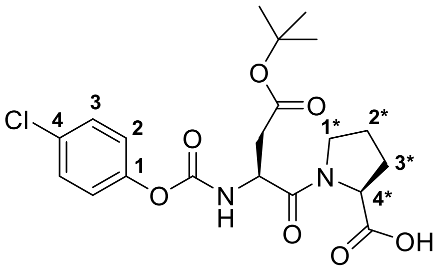

The compound was synthesized on a 2-chlorotrityl resin according to GP9 using Fmoc-(β-*t*Bu)Asp-OH (0.41 g). Column chromatography on silica gel using EtOAc afforded the product as a white solid (77 mg, 35%). The material was dissolved in water (20 mL) and subjected to lyophilization. mp 77–78 °C; ^{1}H NMR (500 MHz, DMSO-d_6) δ 1.40 (s, 9H, $C(CH_3)_3$), 1.82–1.95 (m, 3H, 2*-H, 3*-H), 2.10–2.17 (m, 1H, 3*-H), 2.52 (dd, ^2J = 16.4 Hz, ^3J = 8.2 Hz, 1H, CH_2CO_2tBu), 2.65 (dd, ^2J = 16.4 Hz, ^3J = 6.3 Hz, 1H, CH_2CO_2tBu), 3.60–3.63 (m, 2H, 1*-H), 4.23–4.25 (m, 1H, 4*-H), 4.64–4.68 (m, 1H, NHCH), 7.11 (d, ^3J = 8.9 Hz, 2H, 2-H), 7.41 (d, ^3J = 8.8 Hz, 2H, 3-H), 8.29 (d, ^3J = 8.5 Hz, 1H, OCONH); one signal (COOH) is not visible; ^{13}C NMR (125 MHz, DMSO-d_6) δ 24.55 (C-2*), 27.77 ($C(CH_3)_3$), 28.73 (C-3*), 37.23 (CH_2CO_2tBu), 46.54 (C-1*), 49.66 (NHCH), 58.90 (C-4*), 80.56 ($C(CH_3)_3$), 123.56 (C-2), 129.16 (C-4), 129.33 (C-3), 149.84 (C-1), 153.78 (OCONH), 168.52, 168.98 (CON, CO_2tBu), 173.13 (COOH); LC/MS (ESI): H_2O/MeOH, 90:10 to 0:100; *m/z* 441.3 $[M + H]^+$; 100% purity; Q-TOF: HRMS (ESI): *m/z* $[M + H]^+$ calcd. for $C_{20}H_{25}ClN_2O_7$: 441.1423, found: 441.1407.

4-Chlorophenoxycarbonyl-(γ-*tert*-butyl)glutamyl-proline

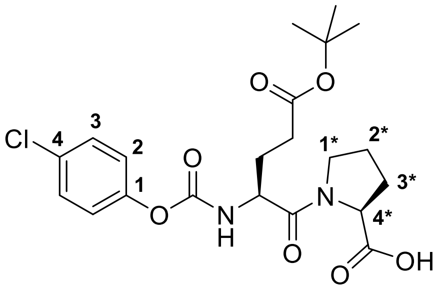

The compound was synthesized on a 2-chlorotrityl resin according to GP9 using Fmoc-(γ-*t*Bu)Glu-OH (0.43 g). Column chromatography on silica gel using EtOAc afforded the product as a white solid (84 mg, 37%). The material was dissolved in water (20 mL) and subjected to lyophilization. mp 58–59 °C; ^{1}H NMR (600 MHz, DMSO-d_6) δ 1.39 (s, 9H, $C(CH_3)_3$), 1.74–1.77 (m, 1H, 2*-H), 1.82–1.86 (m, 1H, 2*-H), 1.88–1.93 (m, 3H, 3*-H, $CHCH_{2,Glu}$), 2.11–2.17 (m, 1H, 3*-H), 2.37 (t, ^3J = 7.3 Hz, 2H, CH_2CO_2tBu), 3.57–3.66 (m, 2H, 1*-H), 4.25–4.28 (m, 1H, 4*-H), 4.33–4.36 (m, 1H, $NHCH_{Glu}$), 7.11

(d, ^3J = 9.0 Hz, 2H, 2-$\underline{H}$), 7.41 (d, ^3J = 9.0 Hz, 2H, 3-$\underline{H}$), 8.11 (d, ^3J = 7.7 Hz, 1H, OCON$\underline{H}$), 12.43 (br s, 1H, COO$\underline{H}$); ^{13}C NMR (150 MHz, DMSO-d_6) δ 24.65 ($\underline{C}$-2*), 26.45 ($CH\underline{C}H_{2,Glu}$), 27.92 ($C(\underline{C}H_3)_3$), 28.73 ($\underline{C}$-3*), 30.66 ($\underline{C}H_2CO_2t$Bu), 46.64 ($\underline{C}$-1*), 51.66 ($NH\underline{C}H_{Glu}$), 58.68 ($\underline{C}$-4*), 79.95 ($\underline{C}(CH_3)_3$), 123.65 ($\underline{C}$-2), 129.23 ($\underline{C}$-4), 129.32 ($\underline{C}$-3), 149.90 ($\underline{C}$-1), 154.04 (O$\underline{C}$ONH), 169.73 ($\underline{C}$ON), 171.99 ($\underline{C}O_2t$Bu), 173.31 ($\underline{C}$OOH); LC/MS (ESI): H_2O/MeOH, 90:10 to 0:100; *m/z* 455.2 $[M + H]^+$; 98% purity; Q-TOF: HRMS (ESI): *m/z* $[M + H]^+$ calcd. for $C_{21}H_{27}ClN_2O_7$: 455.1580, found: 455.1568.

4-Chlorophenoxycarbonyl-β-alanyl-proline

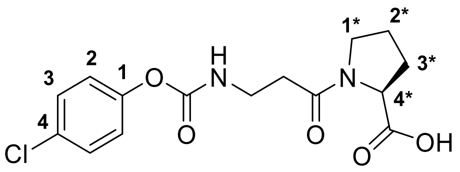

The compound was synthesized on a 2-chlorotrityl resin according to GP9 using Fmoc-β-Ala-OH (0.31 g). The product was purified by column chromatography on silica gel using EtOAc / MeOH (9:1) to obtain a colorless resin (70 mg, 41%). ^{1}H NMR (500 MHz, DMSO-d_6) δ 1.78–1.94 (m, 3H, 2*-$\underline{H}$, 3*-$\underline{H}$), 2.08–2.18 (m, 1H, 3*-$\underline{H}$), 2.50–2.58 (m, 2H, $NHCH_2C\underline{H}_{2,ß\text{-}Ala}$), 3.27 (dt, ^3J = 5.4 Hz, ^3J = 7.1 Hz, 2H, $NHC\underline{H}_2CH_{2,ß\text{-}Ala}$), 4.22 (dd, ^3J = 3.8 Hz, ^3J = 8.8 Hz, 1H, 4*-$\underline{H}$), 7.10–7.15 (m, 2H, 2-$\underline{H}$), 7.38–7.43 (m, 2H, 3-$\underline{H}$), 7.70 (t, ^3J = 5.7 Hz, 1H, OCON$\underline{H}$), two signals (1*-$\underline{H}$) are obscured by the water peak, one signal (COO$\underline{H}$) is not visible; ^{13}C NMR (125 MHz, DMSO-d_6) δ 24.46 ($\underline{C}$-2*), 29.04 ($\underline{C}$-3*), 33.83 ($NHCH_2\underline{C}H_{2,ß\text{-}Ala}$), 36.75 ($NH\underline{C}H_2CH_{2,ß\text{-}Ala}$), 46.66 ($\underline{C}$-1*), 58.43 ($\underline{C}$-4*), 123.75 ($\underline{C}$-2), 129.13 ($\underline{C}$-4), 129.28 ($\underline{C}$-3), 150.03 ($\underline{C}$-1), 154.06 (O$\underline{C}$ONH), 169.02 ($\underline{C}$ON), 173.60 ($\underline{C}$OOH); LC/MS (ESI): H_2O/MeOH, 90:10 to 0:100; *m/z* 340.8 $[M + H]^+$; 100% purity; Q-TOF: HRMS (ESI): *m/z* $[M + H]^+$ calcd. for $C_{15}H_{17}ClN_2O_5$: 341.0899, found: 341.0892.

(*R*)-2-(4-Chlorophenoxycarbonylaminomethyl)-4-methylpentanoyl-proline (9)

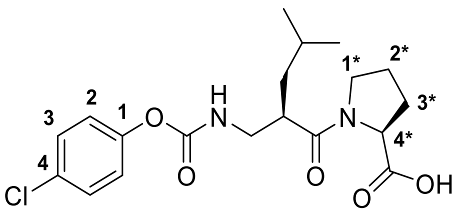

The compound was synthesized on a 2-chlorotrityl resin according to GP9 using Fmoc-APA-OH (0.37 g). The crude product was dissolved in MeOH (101 mg/mL) and purified by semi-preparative HPLC using an isocratic elution with MeCN / H_2O (50:50) for 15 min (250 µL; 16.0 mL/min; 230 nm). Subsequent volatile evaporation and lyophilization afforded the product as a white solid (50 mg, 25%). mp 98–100 °C; ^{1}H NMR (500 MHz, DMSO-d_6) δ 0.85 (2 × d, ^3J = 6.7 Hz, 6H, $CH(C\underline{H}_3)_2$), 1.13–1.20 (m, 1H, $C\underline{H}_2CH(CH_3)_2$), 1.46–1.53 (m, 1H, $C\underline{H}_2CH(CH_3)_2$), 1.56–1.64 (m, 1H, $C\underline{H}(CH_3)_2$), 1.79–1.87 (m, 1H, 2*-$\underline{H}$), 1.88–1.96 (m, 2H, 2*-$\underline{H}$, 3*-$\underline{H}$), 2.11–2.19 (m, 1H, 3*-$\underline{H}$), 2.87–2.92 (m, 1H, $NHCH_2C\underline{H}$), 3.00–3.07 (m, 1H, $NHC\underline{H}_2CH$), 3.11–3.18 (m, 1H, $NHC\underline{H}_2CH$), 3.66 (dt, ^2J

= 9.8 Hz, ^3J = 6.7 Hz, 1H, 1*-H), 4.24 (dd, ^3J = 4.8 Hz, ^3J = 8.6 Hz, 1H, 4*-H), 7.07–7.15 (m, 2H, 2-H), 7.38–7.44 (m, 2H, 3-H), 7.88 (t, ^3J = 5.8 Hz, 1H, OCONH), 12.30 (br s, 1H, COOH); one signal (1*-H) is obscured by the water peak; ^{13}C NMR (125 MHz, DMSO-d_6) δ 22.52, 23.19, 24.65 ($CH(CH_3)_2$), 25.22 (C-2*), 28.95 (C-3*), 39.12 ($CH_2CH(CH_3)_2$), 40.88 ($NHCH_2CH$), 43.27 ($NHCH_2CH$), 46.85 (C-1*), 58.61 (C-4*), 123.65 (C-2), 129.16 (C-4), 129.30 (C-3), 149.93 (C-1), 154.27 (OCONH), 172.07 (CON), 173.50 (COOH); LC/MS (ESI): H_2O/MeOH, 90:10 to 0:100; *m/z* 397.0 $[M + H]^+$; 100% purity; Q-TOF: HRMS (ESI): *m/z* $[M + H]^+$ calcd. for $C_{19}H_{25}ClN_2O_5$: 397.1525, found: 397.1515.

4-Chlorophenoxycarbonyl-leucyl-homoproline (10)

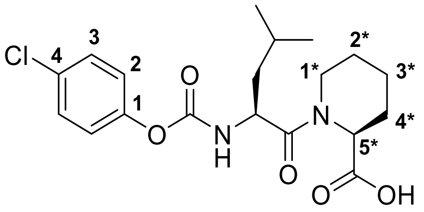

The compound was synthesized on a 2-chlorotrityl resin according to GP9 using Fmoc-Leu-OH (0.35 g). The crude product was dissolved in MeOH (186 mg/mL) and purified by semi-preparative HPLC using an isocratic elution with MeCN / H_2O (50:50) plus 0.1% TFA for 20 min (100 µL; 15.0 mL/min; 230 nm). Subsequent volatile evaporation and lyophilization afforded the product as a white solid (24 mg, 12%). mp 93–95 °C; ^{1}H NMR (500 MHz, DMSO-d_6) δ 0.90 (2 × d, ^3J = 6.7 Hz, 6H, $CH(CH_3)_{2,Leu}$), 1.21–1.30 (m, 1H, $CHCH_{2,Leu}$), 1.32–1.58 (m, 3H, 2*-H, 3*-H), 1.59–1.77 (m, 4H, 3*-H, 4*-H, $CHCH_{2,Leu}$, $CH(CH_3)_{2,Leu}$), 2.09–2.14 (m, 1H, 4*-H), 3.15 (dt, ^2J = 13.0 Hz, ^3J = 2.9 Hz, 1H, 1*-H), 3.78–3.83 (m, 1H, 1*-H), 4.52–4.59 (m, 1H, $NHCH_{Leu}$), 5.10 (dd, ^3J = 2.3 Hz, ^3J = 6.1 Hz, 1H, 5*-H), 7.05–7.16 (m, 2H, 2-H), 7.38–7.44 (m, 2H, 3-H), 8.06 (d, ^3J = 8.1 Hz, 1H, OCONH), 12.77 (br s, 1H, COOH); ^{13}C NMR (125 MHz, DMSO-d_6) δ 20.79 (C-3*), 21.52, 23.24, 24.38 ($CH(CH_3)_{2,Leu}$), 25.06 (C-2*), 26.40 (C-4*), 42.93 (C-1*), 49.52 ($NHCH_{Leu}$), 51.76 (C-5*), 123.59 (C-2), 129.15 (C-4), 129.30 (C-3), 149.94 (C-1), 154.03 (OCONH), 171.36 (CON), 172.42 (COOH), one signal ($CHCH_{2,Leu}$) is obscured by the solvent peak; LC/MS (ESI): H_2O/MeOH, 90:10 to 0:100; *m/z* 397.0 $[M + H]^+$; 100% purity; Q-TOF: HRMS (ESI): *m/z* $[M + H]^+$ calcd. for $C_{19}H_{25}ClN_2O_5$: 397.1525, found: 397.1508.

4-Chlorophenoxycarbonyl-propargylglycyl-proline

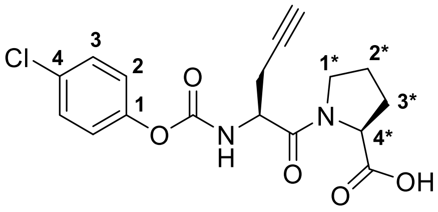

The compound was synthesized on a 2-chlorotrityl resin according to GP9 using Fmoc-Pra-OH (0.34 g). The product was purified by column chromatography on silica gel using EtOAc

/ MeOH (9:1) to obtain a white solid (95 mg, 52%). mp 148–150 °C; ^{1}H NMR (600 MHz, $CDCl_3$) δ 1.97–2.08 (m, 2H, 2*-H), 2.09 (t, ^{3}J = 2.6 Hz, 1H, C≡CH), 2.12–2.24 (m, 2H, 3*-H), 2.61–2.69 (m, 1H, $CHH_{2,Pra}$), 2.71–2.78 (m, 1H, $CHH_{2,Pra}$), 3.75 (dd, ^{3}J = 6.0 Hz, ^{3}J = 7.6 Hz, 2H, 1*-H), 4.58 (dd, ^{3}J = 4.3 Hz, ^{3}J = 8.3 Hz, 1H, 4*-H), 4.70–4.76 (m, 1H, $NHCH_{Pra}$), 6.19 (d, ^{3}J = 8.6 Hz, 1H, NH), 7.00–7.10 (m, 2H, 2-H), 7.27–7.32 (m, 2H, 3-H), one signal (COOH) is not visible; ^{13}C NMR (150 MHz, $CDCl_3$) δ 22.55 ($CHCH_{2,Pra}$), 24.83 (C-2*), 28.29 (C-3*), 47.69 (C-1*), 51.25 ($NHCH_{Pra}$), 59.48 (C-4*), 71.83 (C≡CH), 78.00 (C≡CH), 122.91 (C-2), 129.37 (C-3), 130.96 (C-4), 149.24 (C-1), 153.94 (OCONH), 170.29 (CON), 173.92 (COOH); LC/MS (ESI): H_2O/MeOH, 90:10 to 0:100; *m/z* 364.9 $[M + H]^+$; 100% purity; Q-TOF: HRMS (ESI): *m/z* $[M + H]^+$ calcd. for $C_{17}H_{17}ClN_2O_5$: 365.0899, found: 365.0904.

4-Chlorophenoxycarbonyl-cyclohexylglycyl-proline (11)

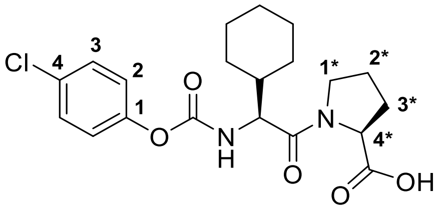

The compound was synthesized on a 2-chlorotrityl resin according to GP9 using Fmoc-Chg-OH (0.38 g). The product was purified by column chromatography on silica gel using EtOAc / MeOH (9:1) to obtain white solid (72 mg, 35%). mp 125–127 °C; ^{1}H NMR (500 MHz, DMSO-d_6) δ 0.97–1.07 (m, 2H, $CH(CH_2)_5$), 1.11–1.19 (m, 4H, $CH(CH_2)_5$), 1.56–1.76 (m, 5H, $CH(CH_2)_5$), 1.79–1.89 (m, 3H, 2*-H, 3*-H), 2.04–2.13 (m, 1H, 3*-H), 3.53–3.60 (m, 1H, 1*-H), 3.67–3.74 (m, 1H, 1*-H), 4.10 (t, ^{3}J = 8.6 Hz, 1H, $NHCH_{Chg}$), 4.25 (dd, ^{3}J = 4.3 Hz, ^{3}J = 8.6 Hz, 1H, 4*-H), 7.08–7.13 (m, 2H, 2-H), 7.37–7.43 (m, 2H, 3-H), 7.99 (d, ^{3}J = 8.4 Hz, 1H, OCONH), one signal (COOH) is not visible; ^{13}C NMR (125 MHz, DMSO-d_6) δ 21.33 ($CH(CH_2)_5$), 24.61 (C-2*), 25.68, 25.81 ($CH(CH_2)_5$), 26.02, 28.47, 28.61 ($CH(CH_2)_5$), 28.90 (C-3*), 46.97 (C-1*), 57.31 ($NHCH_{Chg}$), 59.10 (C-4*), 123.64 (C-2), 129.11 (C-4), 129.25 (C-3), 149.96 (C-1), 154.23 (OCONH), 169.64 (CON), 172.28 (COOH); LC/MS (ESI): H_2O/MeOH, 90:10 to 0:100; *m/z* 409.1 $[M + H]^+$; 99% purity; Q-TOF: HRMS (ESI): *m/z* $[M + H]^+$ calcd. for $C_{20}H_{25}ClN_2O_5$: 409.1525, found: 409.1520.

4-Chlorophenoxycarbonyl-cyclohexylalanyl-proline *tert*-butyl ester

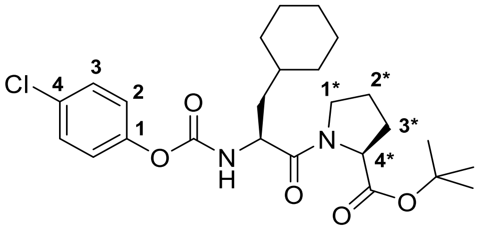

The synthesis was carried out according to GP7 using Cbz-Cha-Pro-O*t*Bu (0.92 g) for the hydrogenation. Subsequently, H-Cha-Pro-O*t*Bu (0.65 g, 2.00 mmol) was reacted with 4-chlorophenyl chloroformate (0.42 g, 2.20 mmol) to generate the desired carbamate. Column chromatography on silica gel using

petrolum ether / EtOAc (4:1) as eluent afforded the product as a colorless resin (0.88 g, 1.83 mmol, 92%). ^{1}H NMR (500 MHz, DMSO-d_6) δ 0.88–0.98 (m, 2H, CH(C$\underline{H}_2$)$_5$), 1.12–1.25 (m, 4H, C$\underline{H}$(C$\underline{H}_2$)$_5$), 1.37 (s, 9H, C(C$\underline{H}_3$)$_3$), 1.52–1.57 (m, 1H, CH(C$\underline{H}_2$)$_5$), 1.59–1.68 (m, 5H, CH(C$\underline{H}_2$)$_5$, NHCHC$\underline{H}_{2,Cha}$), 1.74–1.81 (m, 2H, NHCHC$\underline{H}_{2,Cha}$, 2*-$\underline{H}$), 1.90–1.94 (m, 2H, 2*-$\underline{H}$, 3*-$\underline{H}$), 2.10–2.18 (m, 1H, 3*-$\underline{H}$), 3.46 (dt, 1H, ^{2}J = 9.6 Hz, ^{3}J = 6.6 Hz, 1*-$\underline{H}$), 3.63 (dt, ^{2}J = 9.7 Hz, ^{3}J = 6.8 Hz, 1H, 1*-$\underline{H}$), 4.19 (dd, ^{3}J = 4.8 Hz, ^{3}J = 8.6 Hz, 1H, 4*-$\underline{H}$), 4.29–4.35 (m, 1H, NHC$\underline{H}_{Cha}$), 7.08–7.11 (m, 2H, 2-$\underline{H}$), 7.38–7.43 (m, 2H, 3-$\underline{H}$), 8.08 (d, ^{3}J = 7.9 Hz, 1H, OCON$\underline{H}$); ^{13}C NMR (125 MHz, DMSO-d_6) δ 24.73 ($\underline{C}$-2*), 25.70, 25.92, 26.15 (CH($\underline{C}$H$_2$)$_5$), 27.21 ($\underline{C}$-3*), 28.62 (C($\underline{C}$H$_3$)$_3$), 31.72, 33.56, 33.59 ($\underline{C}$H($\underline{C}$H$_2$)$_5$), 38.12 (NHCH$\underline{C}$H$_{2,Cha}$), 46.41 ($\underline{C}$-1*), 50.54 (NH$\underline{C}$H$_{Cha}$), 59.59 ($\underline{C}$-4*), 80.40 ($\underline{C}$(CH$_3$)$_3$), 123.55 ($\underline{C}$-2), 129.23 ($\underline{C}$-4), 129.28 ($\underline{C}$-3), 149.96 ($\underline{C}$-1), 154.18 (O$\underline{C}$ONH), 171.01, 171.55 (CH$\underline{C}$O); LC/MS (ESI): H_2O/MeCN, 90:10 to 0:100; *m/z* 479.1 [M + H]$^+$; 100% purity; Q-TOF: HRMS (ESI): *m/z* [M + H]$^+$ calcd. for $C_{25}H_{35}ClN_2O_5$: 479.2307, found: 479.2303.

4-Chlorophenoxycarbonyl-cyclohexylalanyl-proline (12)

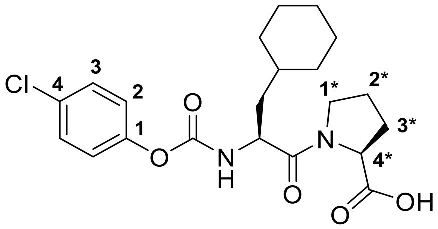

The synthesis was carried out according to GP8 using the corresponding *tert*-butyl ester (144 mg, 0.3 mmol). Column chromatography on silica gel using EtOAc as eluent afforded the product as a colorless resin (118 mg, 0.28 mmol, 93%). ^{1}H NMR (500 MHz, DMSO-d_6) δ 0.87–0.97 (m, 2H, CH(C$\underline{H}_2$)$_5$), 1.10–1.24 (m, 4H, C$\underline{H}$(C$\underline{H}_2$)$_5$), 1.41–1.53 (m, 2H, CH(C$\underline{H}_2$)$_5$), 1.58–1.68 (m, 5H, CH(C$\underline{H}_2$)$_5$, NHCHC$\underline{H}_{2,Cha}$), 1.78–1.86 (m, 2H, NHCHC$\underline{H}_{2,Cha}$, 2*-$\underline{H}$), 1.91–1.95 (m, 1H, 3*-$\underline{H}$), 2.08–2.16 (m, 1H, 3*-$\underline{H}$), 3.47 (dt, 1H, ^{2}J = 9.5 Hz , ^{3}J = 6.6 Hz, 1*-$\underline{H}$), 3.66 (dt, 1H, ^{2}J = 9.6 Hz , ^{3}J = 7.1 Hz, 1H, 1*-$\underline{H}$), 4.26 (dd, ^{3}J = 4.5 Hz, ^{3}J = 8.6 Hz, 1H, 4*-$\underline{H}$), 4.30–4.36 (m, 1H, NHC$\underline{H}_{Cha}$), 7.08–7.12 (m, 2H, 2-$\underline{H}$), 7.39–7.42 (m, 2H, 3-$\underline{H}$), 8.06 (d, ^{3}J = 7.9 Hz, 1H, OCON$\underline{H}$), one signal (COO$\underline{H}$) is not visible; ^{13}C NMR (125 MHz, DMSO-d_6) δ 24.73 ($\underline{C}$-2*), 25.81, 25.98, 26.19 (CH($\underline{C}$H$_2$)$_5$), 28.69 (C-3*), 31.98, 33.52, 33.57 ($\underline{C}$H($\underline{C}$H$_2$)$_5$), 38.26 (NHCH$\underline{C}$H$_{2,Cha}$), 46.53 ($\underline{C}$-1*), 50.46 (NH$\underline{C}$H$_{Cha}$), 58.80 ($\underline{C}$-4*), 123.59 ($\underline{C}$-2), 129.14 ($\underline{C}$-4), 129.30 ($\underline{C}$-3), 149.98 ($\underline{C}$-1), 154.14 (O$\underline{C}$ONH), 172.15, 173.61 (CH$\underline{C}$O); LC/MS (ESI): H_2O/MeCN, 90:10 to 0:100; *m/z* 423.1 [M + H]$^+$; 99% purity; Q-TOF: HRMS (ESI): *m/z* [M + H]$^+$ calcd. for $C_{21}H_{27}ClN_2O_5$: 423.1681, found: 423.1686.

4-Chlorophenoxycarbonyl-glycyl-phenylalanine

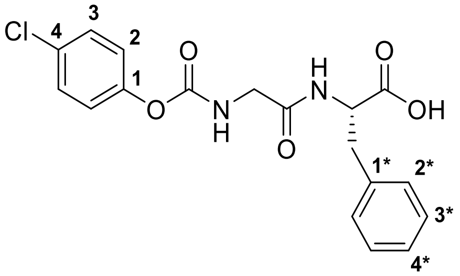

The compound was synthesized on a 2-chlorotrityl resin according to GP9 using Fmoc-Gly-OH (0.30 g). The product was obtained after recrystallization from EtOAc as a white solid (101 mg, 54%). mp 152–154 °C; ^{1}H NMR (500 MHz, DMSO-d_6) δ 2.90 (dd, ^2J = 13.8 Hz, ^3J = 8.9 Hz, 1H, CHC$H_{2,Phe}$), 3.05 (dd, ^2J = 13.8 Hz, ^3J = 5.2 Hz, 1H, CHC$H_{2,Phe}$), 3.58–3.73 (m, 2H, C$H_{2,Gly}$), 4.45 (dt, ^3J = 5.1 Hz, ^3J = 8.5 Hz, 1H, NHCH_{Phe}), 7.09–7.14 (m, 2H, 2-H), 7.17–7.23 (m, 3H, 2*-H, 4*-H), 7.23–7.29 (m, 2H, 3*-H), 7.39–7.44 (m, 2H, 3-H), 7.92 (t, ^3J = 6.2 Hz, 1H, OCONH), 8.20 (d, ^3J = 8.0 Hz, 1H, NHCH_{Phe}), 12.74 (br s, 1H, COOH); ^{13}C NMR (125 MHz, DMSO-d_6) δ 36.95 (CHC$H_{2,Phe}$), 43.40 (C$H_{2,Gly}$), 53.62 (NHCH_{Phe}), 123.61 (C-2), 126.60 (C-4*), 128.34 (C-2*), 129.17 (C-4), 129.26, 129.29 (C-3, C-3*), 137.57 (C-1*), 149.99 (C-1), 154.51 (OCONH), 168.65 (CHCO), 172.88 (COOH); LC/MS (ESI): H_2O/MeOH, 90:10 to 0:100; *m/z* 377.0 [M + H]$^+$, 394.2 [M + NH_4]$^+$; 100% purity; Q-TOF: HRMS (ESI): *m/z* [M + H]$^+$ calcd. for $C_{18}H_{17}ClN_2O_5$: 377.0899, found: 377.0876.

4-Chlorophenoxycarbonyl-valyl-phenylalanine

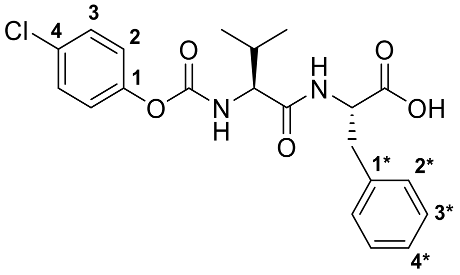

The compound was synthesized on a Wang resin according to GP9 using Fmoc-Val-OH (0.34 g). The product was obtained after recrystallization from EtOAc as a white solid (48 mg, 23%). mp 124–125 °C; ^{1}H NMR (600 MHz, DMSO-d_6) δ 0.84–0.86 (m, 6H, CH(CH_3)$_2$), 1.94–1.99 (m, 1H, CH(CH_3)$_2$), 2.89 (dd, ^2J = 13.9 Hz, ^3J = 9.2 Hz, 1H, CHC$H_{2,Phe}$), 3.05 (dd, ^2J = 13.9 Hz, ^3J = 5.1 Hz, 1H, CHC$H_{2,Phe}$), 3.88 (dd, ^3J = 7.1 Hz, ^3J = 8.9 Hz, 1H, NHCH_{Val}), 4.44–4.48 (m, 1H, NHCH_{Phe}), 7.09 (d, ^3J = 8.8 Hz, 2H, 2-H), 7.19–7.23 (m, 5H, 2*-H, 3*-H, 4*-H), 7.42 (d, ^3J = 8.9 Hz, 2H, 3-H), 7.77 (d, ^3J = 9.2 Hz, 1H, OCONH), 8.21 (d, ^3J = 7.9 Hz, 1H, NHCH_{Phe}), 12.69 (br s, 1H, COOH); ^{13}C NMR (150 MHz, DMSO-d_6) δ 18.59, 19.56 (CH(CH_3)$_2$), 30.88 (CH(CH_3)$_2$), 37.19 (CHC$H_{2,Phe}$), 53.81 (NHCH_{Phe}), 60.73 (NHCH_{Val}), 123.95 (C-2), 126.86 (C-4*), 128.61 (C-2*), 129.47 (C-4), 129.58, 129.63 (C-3, C-3*), 137.95 (C-1*), 150.34 (C-1), 153.42 (OCONH), 171.19 (CHCO), 173.25 (COOH); LC/MS (ESI):

H_2O/MeOH, 90:10 to 0:100; *m/z* 419.36 [M + H]$^+$; 97% purity; Q-TOF: HRMS (ESI): *m/z* [M + H]$^+$ calcd. for $C_{21}H_{23}ClN_2O_5$: 419.1368, found: 419.1383.

4-Chlorophenoxycarbonyl-isoleucyl-phenylalanine

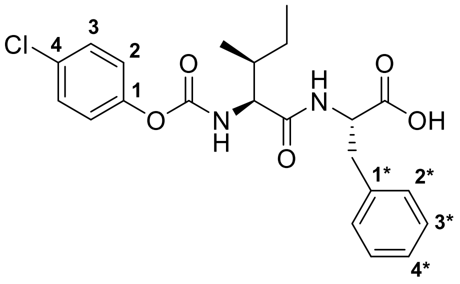

The compound was synthesized on a Wang resin according to GP9 using Fmoc-Ile-OH (0.35 g). Column chromatography on silica gel using EtOAc afforded the product as a white solid (149 mg, 69%). mp 174–176 °C; ^{1}H NMR (500 MHz, DMSO-d_6) δ 0.79–0.82 (m, 6H, 2 × CH_3), 1.08–1.14 (m, 1H, CHCH_2CH_3), 1.40–1.45 (m, 1H, CHCH_2CH$_3$), 1.70–1.74 (m, 1H, CHCH$_2$CH$_3$), 2.89 (dd, ^2J = 13.9 Hz, ^3J = 9.2 Hz, 1H, CHC$H_{2,Phe}$), 3.06 (dd, ^2J = 13.9 Hz, ^3J = 5.1 Hz, 1H, CHC$H_{2,Phe}$), 3.91 (t, ^3J = 8.5 Hz, 1H, NHCH_{Ile}), 4.44–4.49 (m, 1H, NHCH_{Phe}), 7.09 (d, ^3J = 8.9 Hz, 2H, 2-H), 7.19–7.23 (m, 5H, 2*-H, 3*-H, 4*-H), 7.42 (d, ^3J = 8.9 Hz, 2H, 3-H), 7.76 (d, ^3J = 9.2 Hz, 1H, OCONH), 8.21 (d, ^3J = 7.9 Hz, 1H, NHCH_{Phe}), 12.68 (br s, 1H, COOH); ^{13}C NMR (125 MHz, DMSO-d_6) δ 10.94 (CH_2CH_3), 15.26 ($CHCH_3$), 24.32 (CH_2CH_3), 36.54 ($CHCH_3$), 36.83 ($CHCH_{2,Phe}$), 53.40 ($NHCH_{Phe}$), 59.39 ($NHCH_{Ile}$), 123.53 (C-2), 126.46 (C-4*), 128.21 (C-2*), 129.07 (C-4), 129.21, 129.26 (C-3, C-3*), 137.60 (C-1*), 149.98 (C-1), 153.92 (OCONH), 170.87 (CHCO), 172.85 (COOH); LC/MS (ESI): H_2O/MeOH, 90:10 to 0:100; *m/z* 433.25 [M + H]$^+$; 95% purity; Q-TOF: HRMS (ESI): *m/z* [M + H]$^+$ calcd. for $C_{22}H_{25}ClN_2O_5$: 433.1525, found: 433.1540.

4-Chlorophenoxycarbonyl-phenylalanyl-phenylalanine

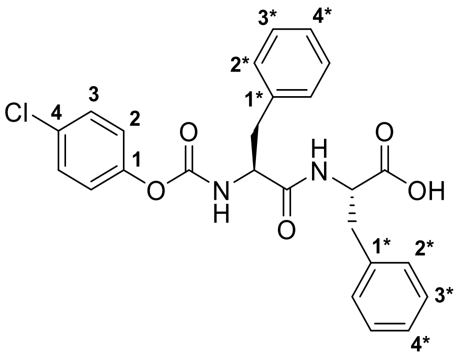

The compound was synthesized on a 2-chlorotrityl resin according to GP9 using Fmoc-Phe-OH (0.39 g). Column chromatography on silica gel using EtOAc afforded the product as a white solid (89 mg, 38%). mp 182–183 °C; ^{1}H NMR (500 MHz, DMSO-d_6) δ 2.75 (dd, ^2J = 13.7 Hz, ^3J = 10.8 Hz, 1H, CHCH_2), 2.95 (dd, ^2J = 13.9 Hz, ^3J = 8.5 Hz, 1H, CHCH_2), 3.02 (dd, ^2J = 13.9 Hz, ^3J = 3.8 Hz, 1H, CHCH_2), 3.09 (dd, ^2J = 14.0 Hz, ^3J = 5.4 Hz, 1H, CHCH_2), 4.27–4.32 (m, 1H, NHCH), 4.46–4.51 (m,

1H, NHC<u>H</u>), 6.93 (d, ^3J = 9.2 Hz, 2H, 2-<u>H</u>), 7.20–7.31 (m, 10H, 2 × (2*-<u>H</u>, 3*-<u>H</u>, 4*-<u>H</u>)), 7.35 (d, ^3J = 8.8 Hz, 2H, 3-<u>H</u>), 7.96 (d, ^3J = 8.9 Hz, 1H, OCON<u>H</u>), 8.32 (d, ^3J = 7.9 Hz, 1H, N<u>H</u>CH), 12.74 (br s, 1H, COO<u>H</u>); ^{13}C NMR (125 MHz, DMSO-d_6) δ 36.81, 37.57 (2 × CH<u>C</u>H$_2$), 53.63, 56.28 (2 × NH<u>C</u>H), 123.36 (<u>C</u>-2), 126.41, 126.52 (2 × <u>C</u>-4*), 128.15, 128.27 (2 × <u>C</u>-2*), 129.07 (<u>C</u>-4), 129.17, 129.26, 129.35 (<u>C</u>-3, 2 × <u>C</u>-3*), 137.53, 137.96 (2 × <u>C</u>-1*), 149.83 (<u>C</u>-1), 153.76 (O<u>C</u>ONH), 171.15 (CH<u>C</u>O), 172.80 (<u>C</u>OOH); LC/MS (ESI): H_2O/MeOH, 90:10 to 0:100; *m/z* 467.3 [M + H]$^+$; 99% purity; Q-TOF: HRMS (ESI): *m/z* [M + H]$^+$ calcd. for $C_{25}H_{23}ClN_2O_5$: 467.1368, found: 467.1371.

4-Chlorophenoxycarbonyl-propargylglycyl-phenylalanine

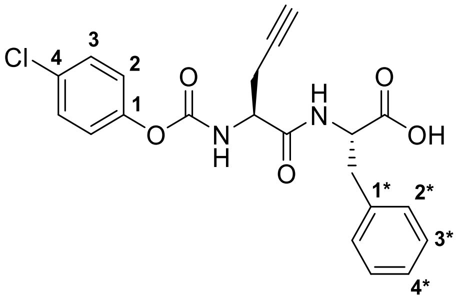

The compound was synthesized on a 2-chlorotrityl resin according to GP9 using Fmoc-Pra-OH (0.34 g). The product was purified by column chromatography on silica gel using EtOAc / MeOH (9:1) to obtain a white solid (155 mg, 75%). mp 170–172 °C; ^{1}H NMR (600 MHz, $CDCl_3$) δ 2.02 (t, ^3J = 2.6 Hz, 1H, C≡C<u>H</u>), 2.58–2.64 (m, 1H, NHCHC<u>H</u>$_{2,Pra}$), 2.73–2.80 (m, 1H, NHCHC<u>H</u>$_{2,Pra}$), 3.11 (dd, ^2J = 14.1 Hz, ^3J = 6.2 Hz, 1H, CHC<u>H</u>$_{2,Phe}$), 3.22 (dd, ^2J = 14.1 Hz, ^3J = 5.6 Hz, 1H, CHC<u>H</u>$_{2,Phe}$), 4.37 (dt, ^3J = 5.3 Hz, ^3J = 7.5 Hz, 1H, NHC<u>H</u>$_{Phe}$), 4.84–4.89 (m, 1H, NHC<u>H</u>$_{Pra}$), 5.92–5.97 (d, ^3J = 7.3 Hz, 1H, OCON<u>H</u>), 6.78 (d, ^3J = 7.6 Hz, 1H, N<u>H</u>CH$_{Phe}$), 7.03 (d, ^3J = 8.3 Hz, 2H, 2-<u>H</u>), 7.13–7.17 (m, 2H, 3-<u>H</u>), 7.21–7.32 (m, 5H, 2*-<u>H</u>, 3*-<u>H</u>, 4*-<u>H</u>), one signal (COO<u>H</u>) signal is not visible; ^{13}C NMR (150 MHz, $CDCl_3$) δ 22.43 (CH<u>C</u>H$_{2,Pra}$), 37.28 (CH<u>C</u>H$_{2,Phe}$), 53.20, 53.40 (NH<u>C</u>H$_{Pra}$, NH<u>C</u>H$_{Phe}$), 72.36 (C≡<u>C</u>H), 78.73 (<u>C</u>≡CH), 122.81 (<u>C</u>-2), 127.36 (<u>C</u>-4*), 128.74 (<u>C</u>-2*), 129.39, 129.42 (<u>C</u>-3, <u>C</u>-3*), 131.06 (<u>C</u>-4), 135.36 (<u>C</u>-1*), 149.12 (<u>C</u>-1), 153.93 (O<u>C</u>ONH), 169.47 (<u>C</u>ONH), 174.08 (<u>C</u>OOH); LC/MS (ESI): H_2O/MeOH, 90:10 to 0:100; *m/z* 415.0 [M + H]$^+$, 432.1 [M + NH_4]$^+$; 100% purity; Q-TOF: HRMS (ESI): *m/z* [M + H]$^+$ calcd. for $C_{21}H_{19}ClN_2O_5$: 415.1055, found: 415.1040.

4-Chlorophenoxycarbonyl-(β-propargyl)asparaginyl-phenylalanine

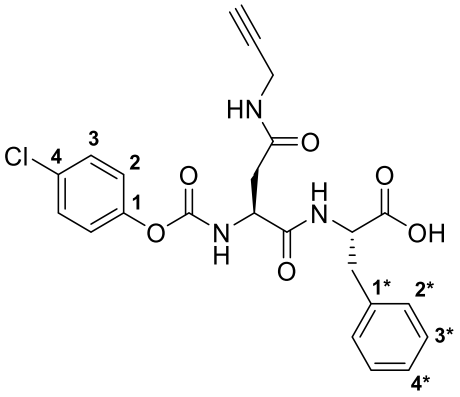

The compound was synthesized on a 2-chlorotrityl resin according to GP9 using Fmoc-(β-propargyl)Asn-OH (0.39 g). The crude product was dissolved in MeCN (105 mg/mL) and purified by semi-preparative HPLC using an isocratic elution with MeCN / H_2O (50:50) plus 0.05% TFA for 25 min (250 µL; 16.0 mL/min; 215 nm). Subsequent volatile evaporation and lyophilization afforded the product as a white solid (108 mg, 46%). mp 182–183 °C; ^{1}H NMR (500 MHz, DMSO-d_6) δ 2.42–2.45 (m, 1H, CH_2CONH), 2.55 (dd, ^2J = 16.1 Hz, ^3J = 4.1 Hz, 1H, CH_2CONH), 2.93 (dd, ^2J = 13.6 Hz, ^3J = 8.5 Hz, 1H, CHC$H_{2,Phe}$), 3.04 (dd, ^2J = 13.6 Hz, ^3J = 4.4 Hz, 1H, CHC$H_{2,Phe}$), 3.09 (s, 1H, C≡CH), 3.86 (d, ^3J = 2.9 Hz, 2H, CH_2C≡CH), 4.41–4.47 (m, 2H, 2 × NHCH), 7.08 (d, ^3J = 8.5 Hz, 2H, 2-H), 7.20–7.24 (m, 5H, 2*-H, 3*-H, 4*-H), 7.41 (d, ^3J = 8.5 Hz, 2H, 3-H), 7.93 (d, ^3J = 8.5 Hz, 1H, OCONH), 8.09 (d, ^3J = 7.0 Hz, 1H, NHCH$_{Phe}$), 8.28 (t, ^3J = 4.7 Hz, 1H, NHCH_2), 12.72 (s, 1H, COOH); ^{13}C NMR (125 MHz, DMSO-d_6) δ 28.11 (NHCH_2), 36.64, 37.17 (CH_2CONH, CHC$H_{2,Phe}$), 51.64, 53.72 (2 × NHCH), 73.17 (C≡CH), 81.17 (C≡CH), 123.48 (C-2), 126.51 (C-4*), 128.27 (C-2*), 129.11 (C-4), 129.26, 129.26 (C-3, C-3*), 137.55 (C-1*), 149.91 (C-1), 153.65 (OCONH), 168.90, 170.79 (CHCO, CH_2CO), 172.73 (COOH); LC/MS (ESI): H_2O/MeOH, 90:10 to 0:100; *m/z* 472.3 [M + H]$^+$; 100% purity; Q-TOF: HRMS (ESI): *m/z* [M + H]$^+$ calcd. for $C_{23}H_{22}ClN_3O_6$: 472.1270, found: 472.1252.

4-Chlorophenoxycarbonyl-(ε-Boc)lysyl-phenylalanine

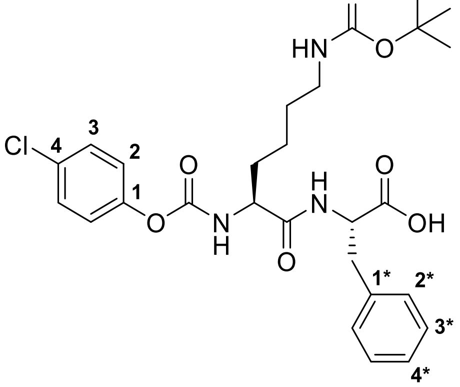

The compound was synthesized on a 2-chlorotrityl resin according to GP9 using Fmoc-(ε-Boc)Lys-OH (0.47 g). Column chromatography on silica gel using EtOAc afforded the product as a white solid (117 mg, 43%). The material was dissolved in water (20 mL) and subjected to lyophilization. mp 151–153 °C; ^{1}H NMR (500 MHz, DMSO-d_6) δ 1.23–1.24 (m, 2H, $C\underline{H}_{2,Lys}$), 1.30–1.39 (m, 11H, $C(C\underline{H}_3)_3$, $C\underline{H}_{2,Lys}$), 1.49–1.62 (m, 2H, $C\underline{H}_{2,Lys}$), 2.85–2.93 (m, 3H, $CHC\underline{H}_{2,Phe}$, $NHC\underline{H}_{2,Lys}$), 3.05 (dd, ^{2}J = 14.2 Hz, ^{3}J = 5.4 Hz, 1H, $CHC\underline{H}_{2,Phe}$), 3.95–4.00 (m, 1H, $NHC\underline{H}_{Lys}$), 4.41–4.45 (m, 1H, $NHC\underline{H}_{Phe}$), 6.69–6.75 (m, 1H, $N\underline{H}CO_2t$Bu), 7.10 (d, ^{3}J = 8.8 Hz, 2H, 2-$\underline{H}$), 7.18–7.25 (m, 5H, 2*-$\underline{H}$, 3*-$\underline{H}$, 4*-$\underline{H}$), 7.41 (d, ^{3}J = 8.8 Hz, 2H, 3-$\underline{H}$), 7.85 (d, ^{3}J = 8.2 Hz, 1H, OCON$\underline{H}$), 8.11 (d, ^{3}J = 7.9 Hz, 1H, $N\underline{H}CH_{Phe}$); one signal (COO$\underline{H}$) is not visible; ^{13}C NMR (125 MHz, DMSO-d_6) δ 22.87 ($\underline{C}H_{2,Lys}$), 28.41 ($C(\underline{C}H_3)_3$), 29.34 ($\underline{C}H_{2,Lys}$), 31.66 ($\underline{C}H_{2,Lys}$), 36.81 ($CH\underline{C}H_{2,Phe}$), 53.49, 54.96 ($NH\underline{C}H_{Lys}$, $NH\underline{C}H_{Phe}$), 77.48 ($\underline{C}(CH_3)_3$), 123.56 ($\underline{C}$-2), 126.50 ($\underline{C}$-4*), 128.24 ($\underline{C}$-2*), 129.10 ($\underline{C}$-4), 129.25, 129.28 ($\underline{C}$-3, $\underline{C}$-3*), 137.58 ($\underline{C}$-1*), 149.97 ($\underline{C}$-1), 153.85 (O$\underline{C}$ONH), 155.70 ($NH\underline{C}O_2t$Bu), 171.56 (CH$\underline{C}$O), 172.86 ($\underline{C}$OOH); one signal ($\underline{C}H_2NH$) is obscured by the solvent peak; LC/MS (ESI): H_2O/MeOH, 90:10 to 0:100; *m/z* 548.4 $[M + H]^+$; 97% purity; Q-TOF: HRMS (ESI): *m/z* $[M + H]^+$ calcd. for $C_{27}H_{34}ClN_3O_7$: 548.2158, found: 548.2135.

4-Chlorophenoxycarbonyl-β-alanyl-phenylalanine

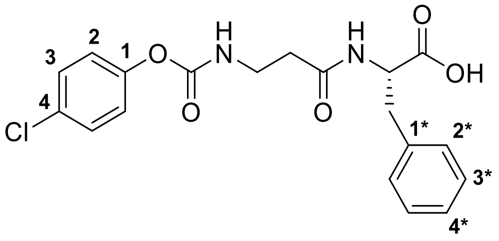

The compound was synthesized on a 2-chlorotrityl resin according to GP9 using Fmoc-β-Ala-OH (0.31 g). The product was obtained after recrystallization from EtOAc as a white solid (50 mg, 26%). mp 160–162 °C; ^{1}H NMR (500 MHz, DMSO-d_6) δ 2.28–2.38 (m, 2H, $NHCH_2C\underline{H}_{2,ß\text{-}Ala}$), 2.86 (dd, ^{2}J = 13.8 Hz, ^{3}J = 9.4 Hz, 1H, $CHC\underline{H}_{2,Phe}$), 3.05 (dd, ^{2}J = 13.9 Hz, ^{3}J = 5.0 Hz, 1H, $CHC\underline{H}_{2,Phe}$), 3.13–3.22 (m, 2H, $NHC\underline{H}_2CH_{2,ß\text{-}Ala}$), 4.41–4.48 (m, 1H, $NHC\underline{H}_{Phe}$), 7.09–7.13 (m, 2H, 2-$\underline{H}$), 7.16–7.28 (m, 5H, 2*-$\underline{H}$, 3*-$\underline{H}$, 4*-$\underline{H}$), 7.37–7.45 (m, 2H, 3-$\underline{H}$), 7.67 (t, ^{3}J = 5.7 Hz, 1H, OCON$\underline{H}$), 8.24 (d, ^{3}J = 8.1 Hz, 1H, $CON\underline{H}_{Phe}$), 12.66 (br s, 1H, COO$\underline{H}$); ^{13}C NMR (125 MHz, DMSO-d_6) δ 35.20 ($NHCH_2\underline{C}H_{2,ß\text{-}Ala}$), 36.92 ($CH\underline{C}H_{2,Phe}$), 37.28 ($NH\underline{C}H_2CH_{2,ß\text{-}Ala}$), 53.52 ($NH\underline{C}H_{Phe}$), 123.69 ($\underline{C}$-2), 126.52 ($\underline{C}$-4*), 128.28 ($\underline{C}$-2*), 129.08 ($\underline{C}$-4), 129.20, 129.23 ($\underline{C}$-3, $\underline{C}$-3*), 137.79 ($\underline{C}$-1*), 150.01 ($\underline{C}$-1), 153.97 (O$\underline{C}$ONH), 170.20 ($CH_2\underline{C}O$), 173.14 ($\underline{C}$OOH); LC/MS (ESI): H_2O/MeOH, 90:10 to 0:100; *m/z* 391.0 $[M + H]^+$, 408.1 $[M + NH_4]^+$; 98% purity; Q-TOF: HRMS (ESI): *m/z* $[M + H]^+$ calcd. for $C_{19}H_{19}ClN_2O_5$: 391.1055, found: 391.1029.

4-Chlorophenoxycarbonyl-valyl-leucine

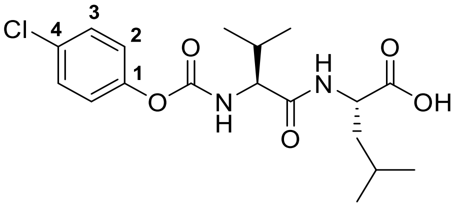

The compound was synthesized on a 2-chlorotrityl resin according to GP9 using Fmoc-Val-OH (0.34 g). The product was obtained after recrystallization from EtOAc as a white solid (46 mg, 24%). mp 172–174 °C; ^{1}H NMR (500 MHz, DMSO-d_6) δ 0.84 (d, ^3J = 6.6 Hz, 3H, C$\underline{H}_3$), 0.88–0.93 (m, 9H, 3 × C$\underline{H}_3$), 1.47–1.57 (m, 2H, CHC$\underline{H}_{2,Leu}$), 1.61–1.68 (m, 1H, C$\underline{H}$(CH$_3$)$_{2,Leu}$), 1.98–2.06 (m, 1H, C$\underline{H}$(CH$_3$)$_{2,Val}$), 3.89–3.92 (m, 1H, NHC$\underline{H}_{Val}$), 4.21–4.26 (m, 1H, NHC$\underline{H}_{Leu}$), 7.11 (d, ^3J = 8.9 Hz, 2H, 2-$\underline{H}$), 7.41 (d, ^3J = 8.8 Hz, 2H, 3-$\underline{H}$), 7.80 (d, ^3J = 8.8 Hz, 1H, OCON$\underline{H}$), 8.12 (d, ^3J = 7.9 Hz, 1H, N$\underline{H}$CH$_{Leu}$), 12.47 (s, 1H, COO$\underline{H}$); ^{13}C NMR (125 MHz, DMSO-d_6) δ 18.28, 19.25, 21.44, 22.96 (2 × CH($\underline{C}$H$_3$)$_2$), 24.37 ($\underline{C}$H(CH$_3$)$_{2,Leu}$), 30.52 ($\underline{C}$H(CH$_3$)$_{2,Val}$), 50.35 (NH$\underline{C}$H$_{Leu}$), 60.33 (NH$\underline{C}$H$_{Val}$), 123.58 ($\underline{C}$-2), 129.10 ($\underline{C}$-4), 129.29 ($\underline{C}$-3), 150.04 ($\underline{C}$-1), 154.16 (O$\underline{C}$ONH), 170.91 (CH$\underline{C}$O), 174.00 ($\underline{C}$OOH); one signal (CH$\underline{C}$H$_{2,Leu}$) is obscured by the solvent peak; LC/MS (ESI): H_2O/MeOH, 90:10 to 0:100; *m/z* 385.1 [M + H]$^+$; 100% purity; Q-TOF: HRMS (ESI): *m/z* [M + H]$^+$ calcd. for $C_{18}H_{25}ClN_2O_5$: 385.1525, found: 385.1533.

4-Chlorophenoxycarbonyl-leucyl-leucine

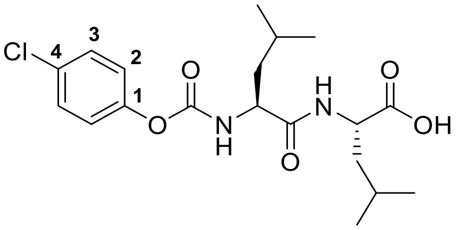

The compound was synthesized on a 2-chlorotrityl resin according to GP9 using Fmoc-Leu-OH (0.35 g). The product was obtained after recrystallization from EtOAc as a white solid (101 mg, 51%). mp 175–176 °C; ^{1}H NMR (500 MHz, DMSO-d_6) δ 0.83 (d, ^3J = 6.7 Hz, 3H, C$\underline{H}_3$), 0.88–0.91 (m, 9H, 3 × C$\underline{H}_3$), 1.47–1.55 (m, 4H, 2 × NHCHC$\underline{H}_2$), 1.60–1.74 (m, 2H, 2 × C$\underline{H}$(CH$_3$)$_2$), 4.07–4.11 (m, 1H, NHC$\underline{H}$), 4.21–4.26 (m, 1H, NHC$\underline{H}$), 7.11 (d, ^3J = 8.9 Hz, 2H, 2-$\underline{H}$), 7.41 (d, ^3J = 8.8 Hz, 2H, 3-$\underline{H}$), 7.92 (d, ^3J = 8.6 Hz, 1H, OCON$\underline{H}$), 8.10 (d, ^3J = 7.9 Hz, 1H, N$\underline{H}$CH), 12.46 (br s, 1H, COO$\underline{H}$); ^{13}C NMR (125 MHz, DMSO-d_6) δ 21.44, 21.64, 22.97, 23.17, 24.30, 24.36 (2 × $\underline{C}$H($\underline{C}$H$_3$)$_2$), 40.66 (NHCH$\underline{C}$H$_2$), 50.31, 53.27 (2 × NH$\underline{C}$H), 123.52 ($\underline{C}$-2), 129.07 ($\underline{C}$-4), 129.27 ($\underline{C}$-3), 150.01 ($\underline{C}$-1), 153.89 (O$\underline{C}$ONH), 172.01 (CH$\underline{C}$O), 174.02 ($\underline{C}$OOH), one signal (NHCH$\underline{C}$H$_2$) is obscured by the solvent peak; LC/MS (ESI): H_2O/MeOH, 90:10 to 0:100; *m/z* 399.2 [M + H]$^+$; 100% purity; Q-TOF: HRMS (ESI): *m/z* [M + H]$^+$ calcd. for $C_{19}H_{27}ClN_2O_5$: 399.1681, found: 399.1677.

4-Chlorophenoxycarbonyl-phenylalanyl-leucine

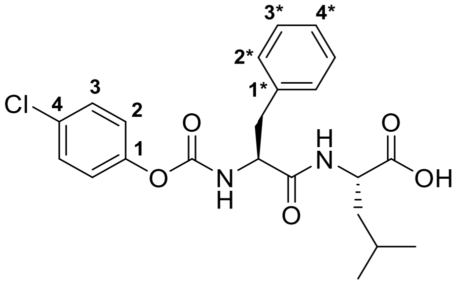

The compound was synthesized on a 2-chlorotrityl resin according to GP9 using Fmoc-Phe-OH (0.39 g). Column chromatography on silica gel using EtOAc afforded the product as a white solid (127 mg, 59%). mp 157–158 °C; ^{1}H NMR (600 MHz, DMSO-d_6) δ 0.88 (2 × d, ^3J = 6.6 Hz, 6H, $CH(CH_3)_{2,Leu}$), 1.54–1.58 (m, 2H, $CHCH_{2,Leu}$), 1.64–1.68 (m, 1H, $CH(CH_3)_{2,Leu}$), 2.78 (dd, ^2J = 13.8 Hz, ^3J = 11.0 Hz, 1H, $CHCH_{2,Phe}$), 3.07 (dd, ^2J = 14.0 Hz, ^3J = 3.6 Hz, 1H, $CHCH_{2,Phe}$), 4.25–4.33 (m, 2H, $NHCH_{Phe}$, $NHCH_{Leu}$), 6.95 (d, ^3J = 8.8 Hz, 2H, 2-H), 7.20–7.35 (m, 5H, 2*-H, 3*-H, 4*-H), 7.37 (d, ^3J = 8.8 Hz, 2H, 3-H), 8.01 (d, ^3J = 8.8 Hz, 1H, OCONH), 8.30 (d, ^3J = 7.7 Hz, 1H, $NHCH_{Leu}$), 12.58 (s, 1H, COOH); ^{13}C NMR (150 MHz, DMSO-d_6) δ 21.50, 23.01, 24.44 ($CH(CH_3)_2$), 37.55 ($CHCH_{2,Phe}$), 50.52 ($NHCH_{Leu}$), 56.29 ($NHCH_{Phe}$), 123.45 (C-2), 126.49 (C-4*), 128.23 (C-2*), 129.10 (C-4), 129.26, 129.43 (C-3, C-3*), 138.09 (C-1*), 149.90 (C-1), 153.92 (OCONH), 171.31 (CHCO), 174.08 (COOH); one signal ($CHCH_{2,Leu}$) is obscured by the solvent peak; LC/MS (ESI): H_2O/MeOH, 90:10 to 0:100; *m/z* 433.1 $[M + H]^+$; 100% purity; Q-TOF: HRMS (ESI): *m/z* $[M + H]^+$ calcd. for $C_{22}H_{25}ClN_2O_5$: 433.1525, found: 433.1528.

4-Chlorophenoxycarbonyl-cyclohexylalanyl-homoproline (13)

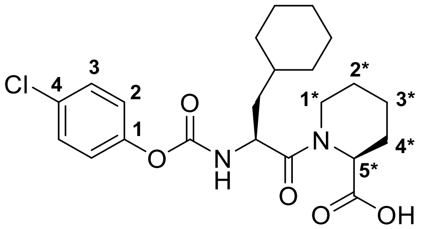

The compound was synthesized on a 2-chlorotrityl resin according to GP9 using Fmoc-Cha-OH (0.39 g). The crude product was dissolved in MeCN (568 mg/mL) and purified by semi-preparative HPLC using an isocratic elution with MeCN / H_2O (60:40) plus 0.1% TFA for 20 min (100 µL; 26.0 mL/min; 220 nm). Subsequent volatile evaporation and lyophilization afforded the product as a white solid (55 mg, 25%). mp 170–172 °C; ^{1}H NMR (600 MHz, DMSO-d_6) δ 0.83–0.91 (m, 1H, $CH(CH_2)_5$), 0.91–0.99 (m, 1H, $CH(CH_2)_5$), 1.08–1.17 (m, 2H, $CH(CH_2)_5$), 1.19–1.26 (m, 2H, $CH(CH_2)_5$), 1.30–1.56 (m, 4H, $NHCHCH_{2,Cha}$, 2*-H, 3*-H), 1.57–1.69 (m, 7H, $NHCHCH_{2,Cha}$, 3*-H, 4*-H, $CH(CH_2)_5$), 1.76–1.83 (m, 1H, $CH(CH_2)_5$), 2.09–2.14 (m, 1H, 4*-H), 3.14 (dt, ^2J = 13.1 Hz, ^3J = 2.9 Hz, 1H, 1*-H), 3.77–3.84 (m, 1H, 1*-H), 4.55–4.63 (m, 1H, $NHCH_{Leu}$), 5.10 (dd, ^3J = 2.3 Hz, ^3J = 6.0 Hz, 1H, 5*-H), 7.06–7.14 (m, 2H, 2-H), 7.39–7.45 (m, 2H, 3-H), 8.07 (d, ^3J = 8.1 Hz, 1H, OCONH), 12.82 (br s, 1H, COOH); ^{13}C NMR (150 MHz, DMSO-d_6) δ 20.82 (C-3*), 25.09 (C-2*), 25.81, 25.96,

26.19 (CH(C̲H$_2$)$_5$), 26.42 (C̲-4*), 31.96, 33.48, 33.66 (C̲H(C̲H$_2$)$_5$), 38.09 (NHCHC̲H$_{2,Cha}$), 42.97 (C̲-1*), 48.83 (NHC̲H$_{Cha}$), 51.74 (C̲-5*), 123.62 (C̲-2), 129.18 (C̲-4), 129.35 (C̲-3), 149.98 (C̲-1), 154.07 (OC̲ONH), 171.55 (C̲ON), 172.49 (C̲OOH); LC/MS (ESI): H_2O/MeOH, 90:10 to 0:100; *m/z* 437.2 [M + H]$^+$; 100% purity; Q-TOF: HRMS (ESI): *m/z* [M + H]$^+$ calcd. for $C_{22}H_{29}ClN_2O_5$: 437.1838, found: 437.1825.

7.3. Carbamates and ureas of type B

4-Chlorophenoxycarbonyl-(*N*-methyl)leucyl-phenylalanine *tert*-butyl ester

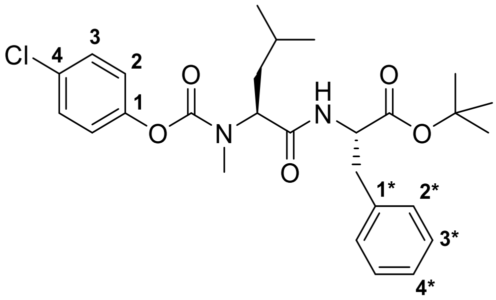

Cbz-(*N*Me)Leu-Pro-O*t*Bu (0.48 g, 1.00 mmol) was treated with Pd/C (10 wt%) and hydrogenated for 1h at 30 psi and rt. The reaction mixture was filtrated over celite and MeOH was evaporated. Subsequently, H-(*N*Me)Leu-Phe-O*t*Bu (0.23 g, 0.65 mmol), 4-chlorophenyl chloroformate (0.12 g, 0.65 mmol) and TEA (66 mg, 0.65 mmol) were dissolved in dry THF (20 ml) and stirred at rt for 2.5 h. Upon evaporation of the solvent, the residue was dissolved in EtOAc (60 mL) and washed with 10% $KHSO_4$ (3 × 50 mL), sat. aq. $NaHCO_3$ (3 × 50 mL) and brine (50 mL) and dried over Na_2SO_4. The solvent was evaporated and the residue was purified by column chromatography on silica gel using a gradient of petrolum ether / EtOAc (7:1 to 3:1) as eluent to obtain a colorless oil and a mixture of isomers (100 mg, 0.20 mmol, 31 %). ^{1}H NMR (500 MHz, DMSO-*d*$_6$; mixture of rotamers) δ 0.87–0.97 (m, 6H, CH(CH̲$_3$)$_2$), 1.36 (s, 9H, C(CH̲$_3$)$_3$), 1.43–1.65 (m, 3H, CHCH̲$_{2,Leu}$, CH̲(CH$_3$)$_{2,Leu}$), 2.72, 2.73 (2 × s, 3H, N(CH̲$_3$)), 2.91–2.98 (m, 1H, CHCH̲$_{2,Phe}$), 3.01–3.10 (m, 1H, CHCH̲$_{2,Phe}$), 4.35–4.46 (m, 1H, NHCH̲$_{Phe}$), 4.64–4.72 (m, 1H, NHCH̲$_{Leu}$), 7.02–7.07 (m, 1H, 4*-H̲), 7.14–7.28 (m, 6H, 2-H̲, 2*-H̲, 3*-H̲), 7.44–7.49 (m, 2H, 3-H̲), 8.37, 8.44 (2 × d, ^3J = 7.9 Hz, 1H, NH̲CH$_{Phe}$); ^{13}C NMR (125 MHz, DMSO-*d*$_6$, mixture of rotamers) δ 21.52, 21.70, 23.13, 24.56 (C̲H(C̲H$_3$)$_{2,Leu}$), 27.68 (C(C̲H$_3$)$_3$), 30.13, 30.47, 36.40, 36.43 (N(C̲H$_3$)), 37.23, 37.63 (CHC̲H$_{2,Leu}$), 40.24 (CHC̲H$_{2,Phe}$), 54.03, 54.26, 56.40, 56.76 (N(CH$_3$)C̲H$_{Leu}$, NHC̲H$_{Phe}$), 80.82 (C̲(CH$_3$)$_3$), 117.06, 123.68, 123.78, 126.53, 128.25, 129.18, 129.25, 129.39, 137.60, 137.68 (C̲-2, C̲-3, C̲-4, C̲-1*, C̲-2*, C̲-3*, C̲-4*), 150.18, 153.75, 154.30, 156.46 (C̲-1, OC̲ON(CH$_3$)), 170.46, 170.56 (CHC̲O); LC/MS (ESI): H_2O/MeCN, 90:10 to 0:100; *m/z* 503.1 [M + H]$^+$; 88% purity; $C_{27}H_{35}ClN_2O_5$.

4-Chlorophenoxycarbonyl-(*N*-methyl)leucyl-phenylalanine (14)

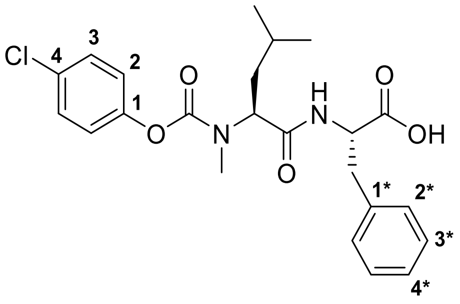

The synthesis was carried out according to GP8 using the corresponding *tert*-butyl ester (83 mg, 0.16 mmol). Column chromatography on silica gel using EtOAc as eluent afforded the product as a colorless oil and as a mixture of isomers (45 mg, 0.10 mmol, 61%). ^{1}H NMR (600 MHz, DMSO-d_6) δ 0.86–0.92 (m, 6H, $CH(CH_3)_2$), 1.41–1.62 (m, 3H, $CHCH_{2,Leu}$, $CH(CH_3)_{2,Leu}$), 2.63 (2 × s, 3H, $N(CH_3)$), 2.91–2.96 (m, 1H, $CHCH_{2,Phe}$), 3.09–3.13 (m, 1H, $CHCH_{2,Phe}$), 4.41–4.49 (m, 1H, $NHCH_{Phe}$), 4.60–4.67 (m, 1H, $NHCH_{Leu}$), 7.02–7.03 (m, 1H, 4*-H), 7.15–7.24 (m, 6H, 2-H, 2*-H, 3*-H), 7.43 – 7.46 (m, 2H, 3-H), 8.24, 8.34 (2 × d, ^3J = 8.0 Hz, 1H, $NHCH_{Phe}$), 12.31 (s, 1H, COOH); ^{13}C NMR (125 MHz, DMSO-d_6, mixture of rotamers) δ 21.19, 21.48, 21.66, 23.19, 24.50, 24.57 ($CH(CH_3)_{2,Leu}$), 29.98, 30.34 ($N(CH_3)$), 36.29, 36.40, 36.97, 37.42 ($CHCH_{2,Leu}$, $CHCH_{2,Phe}$), 53.65, 56.54 ($N(CH_3)CH_{Leu}$, $NHCH_{Phe}$), 123.69, 123.79, 126.45, 128.24, 129.15, 129.23, 129.27, 129.38, 137.91 (C-1, C-2, C-3, C-4, C-1*, C-2*, C-3*, C-4*), 154.30 ($OCON(CH_3)$), 170.10, 172.11, 172.98 (CHCO, COOH); LC/MS (ESI): H_2O/MeOH, 90:10 to 0:100; *m/z* 447.2 $[M + H]^+$; 99% purity; Q-TOF: HRMS (ESI): *m/z* $[M - H]^-$ calcd. for $C_{23}H_{27}ClN_2O_5$: 445.1536, found: 445.1534.

4-Chlorophenoxycarbonyl-(*N*-methyl)leucyl-proline *tert*-butyl ester

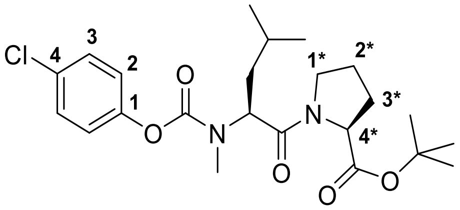

The synthesis was carried out according to GP7 using Cbz-(*N*Me)Leu-Pro-O*t*Bu (0.87 g) for the hydrogenation. Subsequently, H-(*N*Me)Leu-Pro-O*t*Bu (0.60 g, 2.00 mmol) was reacted with 4-chlorophenyl chloroformate (0.42 g, 2.20 mmol) to generate the desired carbamate. Column chromatography on silica gel using petrolum ether / EtOAc (3:1) as eluent afforded the product as a white solid (0.72 g, 1.59 mmol, 80%). mp 86–89 °C; ^{1}H NMR (500 MHz, DMSO-d_6, mixture of rotamers) δ 0.88–0.97 (m, 6H, $CH(CH_3)_{2,Leu}$), 1.30–1.38 (s, 9H, $C(CH_3)_3$), 1.53–1.66 (m, 3H, $CHCH_{2,Leu}$, $CH(CH_3)_{2,Leu}$), 1.76–1.82 (m, 1H, 2*-H), 1.88–2.01 (m, 2H, 2*-H, 3*-H), 2.11–2.21 (m, 1H, 3*-H), 2.85–2.98 (2 × s, 3H, $N(CH_3)$), 3.49–3.66 (m, 2H, 1*-H), 4.18–4.24 (m, 1H, 4*-H), 4.88–4.94 (m, 1H, NCH_{Leu}), 7.13–7.18 (m, 2H, 2-H), 7.42–7.48 (m, 2H, 3-H); ^{13}C NMR (125 MHz, DMSO-d_6, signals of the main rotamer are listed) δ 21.96, 22.88, 24.39 ($CH(CH_3)_{2,Leu}$, C-2*), 27.70 ($C(CH_3)_3$), 28.67 (C-3*),

30.05 (N($\underline{C}H_3$)), 37.16 ($CH\underline{C}H_{2,Leu}$), 46.61 ($\underline{C}$-1*), 54.19 (N(CH_3)$\underline{C}H_{Leu}$), 59.64 ($\underline{C}$-4*), 80.44 ($\underline{C}(CH_3)_3$), 123.83 ($\underline{C}$-2), 129.27 ($\underline{C}$-3, $\underline{C}$-4), 150.04 ($\underline{C}$-1), 154.40 (O$\underline{C}$ON(CH_3)), 168.77 (CH$\underline{C}$O), 170.88 ($\underline{C}$OO); LC/MS (ESI): H_2O/MeOH, 90:10 to 0:100; *m/z* 453.8 $[M + H]^+$; 99% purity; Q-TOF: HRMS (ESI): *m/z* $[M + H]^+$ calcd. for $C_{23}H_{33}ClN_2O_5$: 453.2151, found: 453.2153.

4-Chlorophenoxycarbonyl-(*N*-methyl)leucyl-proline (15)

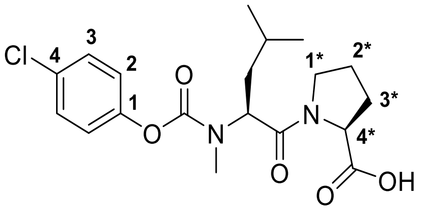

The synthesis was carried out according to GP8 using the corresponding *tert*-butyl ester (136 mg, 0.3 mmol). Column chromatography on silica gel using EtOAc as eluent afforded the product as a slightly yellow resin (111 mg, 0.28 mmol, 93%). ^1H NMR (500 MHz, DMSO-d_6) δ 0.87–0.96 (m, 6H, $CH(C\underline{H}_3)_{2,Leu}$), 1.51–1.59 (m, 2H, $CHC\underline{H}_{2,Leu}$), 1.60–1.70 (m, 1H, $C\underline{H}(CH_3)_{2,Leu}$), 1.82–1.87 (m, 1H, 2*-$\underline{H}$), 1.89–1.94 (m, 2H, 2*-$\underline{H}$, 3*-$\underline{H}$), 2.12–2.24 (m, 1H, 3*-$\underline{H}$), 2.75–2.97 (4 × s, 3H, N($C\underline{H}_3$)), 3.54–3.55 (m, 2H, 1*-$\underline{H}$), 4.24–4.30 (m, 1H, 4*-$\underline{H}$), 4.88–4.94 (m, 1H, $NHC\underline{H}_{Leu}$), 7.13–7.19 (m, 2H, 2-$\underline{H}$), 7.42–7.46 (m, 2H, 3-$\underline{H}$), one signal (COO$\underline{H}$) is not visible; ^{13}C NMR (125 MHz, DMSO-d_6) δ 21.95, 22.97, 24.34 ($\underline{C}H(\underline{C}H_3)_{2,Leu}$, $\underline{C}$-2*), 28.68 ($\underline{C}$-3*), 30.03 (N($\underline{C}H_3$)), 37.08 ($CH\underline{C}H_{2,Leu}$), 46.58 ($\underline{C}$-1*), 54.29 (N(CH_3)$\underline{C}H_{Leu}$), 58.81 ($\underline{C}$-4*), 123.84 ($\underline{C}$-2), 129.28 ($\underline{C}$-3, $\underline{C}$-4), 150.06 ($\underline{C}$-1), 154.40 (O$\underline{C}$ON(CH_3)), 168.79 (CH$\underline{C}$O), 173.20 ($\underline{C}$OOH); LC/MS (ESI): H_2O/MeOH, 90:10 to 0:100; *m/z* 397.1 $[M + H]^+$; 99% purity; Q-TOF: HRMS (ESI): *m/z* $[M + H]^+$ calcd. for $C_{19}H_{25}ClN_2O_5$: 397.1525, found: 397.1526.

(*S*)-2-(4-Chloroanilinocarbonyloxy)-4-methylpentanoyl-phenylalanine *tert*-butyl ester

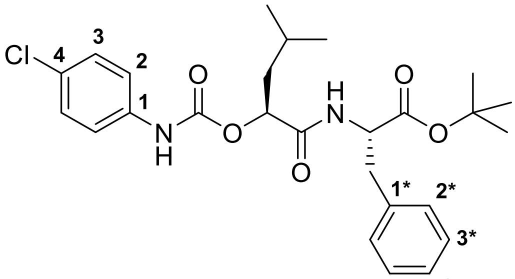

4-Chlorophenyl isocyanate (0.09 g, 0.60 mmol) and compound **34** (0.20 g, 0.60 mmol) were suspended in toluene (10 ml) and stirred under reflux for 2 h. Upon evaporation of the solvent, the residue was purified by column chromatography on silica gel using petrolum ether / EtOAc (1:1) as eluent to obtain the desired *N*-aryl carbamate as a white solid (0.24 g, 0.50 mmol, 83 %). mp 141–143 °C; ^1H NMR (500 MHz, DMSO-d_6) δ 0.89 (2 × d, ^3J = 6.6 Hz, 6H, $CH(C\underline{H}_3)_2$), 1.30 (s, 9H, $C(C\underline{H}_3)_3$), 1.47–1.53 (m, 1H, $OCHC\underline{H}_2$, 1.54–1.60 (m, 1H, $OCHC\underline{H}_2$), 1.66–1.74 (m, 1H, $C\underline{H}(CH_3)_2$), 2.93–3.02 (m, 2H, $CHC\underline{H}_{2,Phe}$), 4.38 (dt, ^3J = 7.8 Hz, ^3J = 6.5 Hz, 1H, $NHC\underline{H}_{Phe}$), 4.98 (dd, ^3J = 4.3 Hz, ^3J = 9.6 Hz, 1H, $OC\underline{H}CH_2$),

7.17–7.26 (m, 5H, 2*-$\underline{H}$, 3*-$\underline{H}$, 4*-$\underline{H}$), 7.31–7.34 (m, 2H, 3-$\underline{H}$), 7.48 (d, ^3J = 8.9 Hz, 2H, 2-$\underline{H}$), 8.34 (d, ^3J = 7.6 Hz, 1H, $N\underline{H}CH_{Phe}$), 9.89 (s, 1H, $OCON\underline{H}$); ^{13}C NMR (125 MHz, DMSO-d_6) δ 21.66, 23.14, 24.10 ($\underline{C}H(\underline{C}H_3)_2$), 27.62 ($C(\underline{C}H_3)_3$), 36.69 ($CH\underline{C}H_{2,Phe}$), 40.52 ($OCH\underline{C}H_2$), 54.00 ($NH\underline{C}H_{Phe}$), 71.86 ($O\underline{C}HCH_2$), 80.79 ($\underline{C}(CH_3)_3$), 119.73 ($\underline{C}$-2), 126.21, 126.55 ($\underline{C}$-4, $\underline{C}$-4*), 128.23, 128.76, 129.32 ($\underline{C}$-3, $\underline{C}$-2*, $\underline{C}$-3*), 137.73, 138.15 ($\underline{C}$-1, $\underline{C}$-1*), 152.80 ($O\underline{C}ONH$), 170.13, 170.28 ($CH\underline{C}O$); LC/MS (ESI): H_2O/MeOH, 90:10 to 0:100; *m/z* 489.2 $[M + H]^+$; 98% purity; Q-TOF: HRMS (ESI): *m/z* $[M - H]^-$ calcd. for $C_{26}H_{33}ClN_2O_5$: 487.2005, found: 487.2009.

(*S*)-2-(4-Chloroanilinocarbonyloxy)-4-methylpentanoyl-phenylalanine (16)

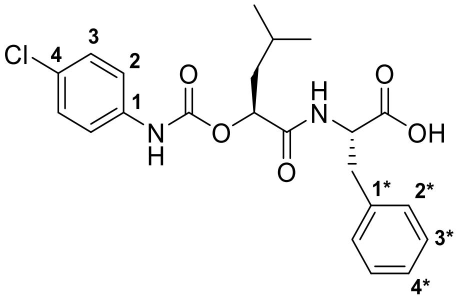

The synthesis was carried out according to GP8 using the corresponding *tert*-butyl ester (97 mg, 0.20 mmol). Column chromatography on silica gel was not required to obtain the product as a slightly brown solid (78 mg, 0.18 mmol, 90%). mp 161–163 °C; ^{1}H NMR (500 MHz, DMSO-d_6) δ 0.87 (2 × d, ^3J = 6.6 Hz, 6H, $CH(C\underline{H}_3)_2$), 1.44–1.49 (m, 1H, $OCHC\underline{H}_2$), 1.51–1.57 (m, 1H, $OCHC\underline{H}_2$), 1.64–1.72 (m, 1H, $C\underline{H}(CH_3)_2$), 2.95 (dd, ^2J = 13.9 Hz, ^3J = 8.8 Hz, 1H, $CHC\underline{H}_{2,Phe}$), 3.07 (dd, ^2J = 13.9 Hz, ^3J = 5.1 Hz, 1H, $CHC\underline{H}_{2,Phe}$), 4.47 (dt, ^3J = 5.2 Hz, ^3J = 8.4 Hz, 1H, $NHC\underline{H}_{Phe}$), 4.98 (dd, ^3J = 4.2 Hz, ^3J = 9.8 Hz, 1H, $OC\underline{H}CH_2$), 7.15–7.19 (m, 1H, 4*-$\underline{H}$), 7.21–7.25 (m, 4H, 2*-$\underline{H}$, 3*-$\underline{H}$), 7.31–7.34 (m, 2H, 3-$\underline{H}$), 7.47 (d, ^3J = 8.9 Hz, 2H, 2-$\underline{H}$), 8.25 (d, ^3J = 7.9 Hz, 1H, $N\underline{H}CH_{Phe}$), 9.89 (s, 1H, $OCON\underline{H}$), one signal ($COO\underline{H}$) is not visible; ^{13}C NMR (125 MHz, DMSO-d_6) δ 21.67, 23.17, 24.09 ($\underline{C}H(\underline{C}H_3)_2$), 36.63 ($CH\underline{C}H_{2,Phe}$), 40.50 ($OCH\underline{C}H_2$), 53.23 ($NH\underline{C}H_{Phe}$), 71.98 ($O\underline{C}HCH_2$), 119.81 ($\underline{C}$-2), 126.27, 126.53 ($\underline{C}$-4, $\underline{C}$-4*), 128.27, 128.80, 129.29 ($\underline{C}$-3, $\underline{C}$-2*, $\underline{C}$-3*), 137.59, 138.17 ($\underline{C}$-1, $\underline{C}$-1*), 152.81 ($O\underline{C}ONH$), 170.18 ($CH\underline{C}O$), 172.72 ($\underline{C}OOH$); LC/MS (ESI): H_2O/MeOH, 90:10 to 0:100; *m/z* 433.1 $[M + H]^+$; 100% purity; Q-TOF: HRMS (ESI): *m/z* $[M - H]^-$ calcd. for $C_{22}H_{25}ClN_2O_5$: 431.1379, found: 431.1387.

(*S*)-2-(4-Chloroanilinocarbonyloxy)-4-methylpentanoyl-proline *tert*-butyl ester

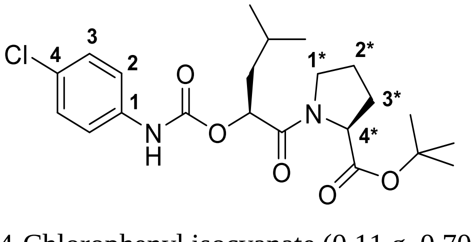

4-Chlorophenyl isocyanate (0.11 g, 0.70 mmol) and compound **35** (0.20 g, 0.70 mmol) were suspended in toluene (15 ml) and stirred under reflux for 2 h. Upon evaporation of the solvent, the residue was

purified by column chromatography on silica gel using petrolum ether / EtOAc (3:2) as eluent to obtain the desired *N*-aryl carbamate as a white solid (0.28 g, 0.63 mmol, 90 %). mp 155–157 °C; ^{1}H NMR (600 MHz, DMSO-d_6) δ 0.95 (2 × d, ^{3}J = 6.7 Hz, 6H, $CH(CH_3)_2$), 1.36 (s, 9H, $C(CH_3)_3$), 1.49–1.53 (m, 1H, $OCHCH_2$), 1.64–1.69 (m, 1H, $OCHCH_2$), 1.76–1.86 (m, 2H, $CH(CH_3)_2$, 2*-H), 1.92–1.97 (m, 2H, 2*-H, 3*-H), 2.13–2.19 (m, 1H, 3*-H), 3.47 (dt, ^{2}J = 9.6 Hz, ^{3}J = 6.8 Hz, 1H, 1*-H), 3.72 (dt, ^{2}J = 9.6 Hz, ^{3}J = 6.8 Hz, 1H, 1*-H), 4.20 (dd, ^{3}J = 5.1 Hz, ^{3}J = 8.5 Hz, 1H, 4*-H), 5.11 (dd, ^{3}J = 3.1 Hz, ^{3}J = 10.3 Hz, 1H, $OCHCH_2$), 7.30–7.32 (m, 2H, 3-H), 7.44–7.48 (m, 2H, 2-H), 10.00 (s, 1H, OCONH); ^{13}C NMR (125 MHz, DMSO-d_6) δ 21.48, 23.25, 24.23 ($CH(CH_3)_2$), 24.80 (C-2*), 27.70 ($C(CH_3)_3$), 28.53 (C-3*), 46.31 (C-1*), 59.60 (C-4*), 70.18 ($OCHCH_2$), 80.52 ($C(CH_3)_3$), 119.76 (C-2), 126.26 (C-4), 128.78 (C-3), 138.09 (C-1), 153.06 (OCONH), 168.53 (CHCO), 170.85 (COO), one signal ($OCHCH_2$) is obscured by the DMSO solvent signal; LC/MS (ESI): H_2O/MeOH, 90:10 to 0:100; *m/z* 439.1 $[M + H]^+$; 92% purity; Q-TOF: HRMS (ESI): *m/z* $[M + H]^+$ calcd. for $C_{22}H_{31}ClN_2O_5$: 439.1994, found: 439.1978.

(*S*)-2-(4-Chloroanilinocarbonyloxy)-4-methylpentanoyl-proline (17)

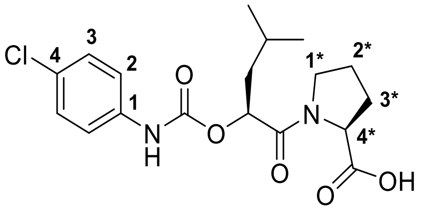

The synthesis was carried out according to GP8 using the corresponding *tert*-butyl ester (132 mg, 0.30 mmol). Column chromatography on silica gel using EtOAc as eluent afforded the product as a white solid (84 mg, 0.22 mmol, 73%). mp 188–190 °C; ^{1}H NMR (500 MHz, DMSO-d_6) δ 0.95 (d, ^{3}J = 6.6 Hz, 6H, $CH(CH_3)_2$), 1.49–1.54 (m, 1H, $OCHCH_2$), 1.63–1.69 (m, 1H, $OCHCH_2$), 1.79–1.87 (m, 2H, $CH(CH_3)_2$, 2*-H), 1.92–1.98 (m, 2H, 2*-H, 3*-H), 2.12–2.19 (m, 1H, 3*-H), 3.50 (dt, ^{2}J = 9.3 Hz, ^{3}J = 6.6 Hz, 1H, 1*-H), 3.73 (dt, ^{2}J = 9.4 Hz, ^{3}J = 6.9 Hz, 1H, 1*-H), 4.28 (dd, ^{3}J = 4.6 Hz, ^{3}J = 8.7 Hz, 1H, 4*-H), 5.13 (dd, ^{3}J = 3.4 Hz, ^{3}J = 10.1 Hz, 1H, $OCHCH_2$), 7.31 (d, ^{3}J = 8.6 Hz, 2H, 3-H), 7.46 (d, ^{3}J = 8.8 Hz, 2H, 2-H), 10.00 (s, 1H, OCONH), 12.43 (s, 1H, COOH); ^{13}C NMR (125 MHz, DMSO-d_6) δ 21.55, 23.26, 24.13 ($CH(CH_3)_2$), 24.78 (C-2*), 28.53 (C-3*), 46.31 (C-1*), 58.65 (C-4*), 70.17 ($OCHCH_2$), 119.78 (C-2), 126.27 (C-4), 128.77 (C-3), 138.77 (C-1), 153.08 (OCONH), 168.61 (CHCO), 173.14 (COOH), one signal ($OCHCH_2$) is obscured by the DMSO solvent signal; LC/MS (ESI): H_2O/MeOH, 90:10 to 0:100; *m/z* 383.2 $[M + H]^+$; 99% purity; Q-TOF: HRMS (ESI): *m/z* $[M + H]^+$ calcd. for $C_{18}H_{23}ClN_2O_5$: 383.1368, found: 383.1371.

4-Chloroanilinocarbonyl-leucyl-phenylalanine *tert*-butyl ester

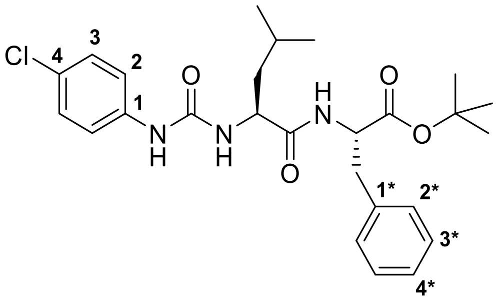

Cbz-Leu-Phe-O*t*Bu (0.47 g, 1.00 mmol) was treated with Pd/C (10 wt%) and hydrogenated for 1h at 30 psi and rt. The reaction mixture was filtrated over celite and MeOH was evaporated. The obtained amine (0.27 g, 0.80 mmol) and 4-chlorophenyl isocyanate (0.12 g, 0.80 mmol) were dissolved in dry CH_2Cl_2 (15 ml) and stirred at rt for 16 h. The solvent was evaporated and the residue was purified by recrystallization from petrolum ether / EtOAc (1:1, 8 ml) to obtain the urea as a white solid (0.15 g, 0.30 mmol, 37%). mp 168– 170 °C; ^{1}H NMR (500 MHz, DMSO-d_6) δ 0.88 (2 × d, ^3J = 6.6 Hz, 6H, CH(C$\underline{H}_3$)$_{2,\mathrm{Leu}}$), 1.30 (s, 9H, C(C$\underline{H}_3$)$_3$), 1.33–1.37 (m, 1H, CHC$\underline{H}_{2,\mathrm{Leu}}$), 1.42–1.47 (m, 1H, CHC$\underline{H}_{2,\mathrm{Leu}}$), 1.57–1.65 (m, 1H, C$\underline{H}$(CH$_3$)$_{2,\mathrm{Leu}}$), 2.89–3.00 (m, 2H, CHC$\underline{H}_{2,\mathrm{Phe}}$), 4.27 (dt, ^3J = 5.3 Hz, ^3J = 9.0 Hz, 1H, NHC$\underline{H}_{\mathrm{Phe}}$), 4.33–4.38 (m, 1H, NHC$\underline{H}_{\mathrm{Leu}}$), 6.26 (d, ^3J = 8.6 Hz, 1H, N$\underline{H}$CH$_{\mathrm{Leu}}$), 7.14–7.18 (m, 1H, 4*-$\underline{H}$), 7.20–7.26 (m, 6H, 3-$\underline{H}$, 2*-$\underline{H}$, 3*-$\underline{H}$), 7.36–7.39 (m, 2H, 2-$\underline{H}$), 8.39 (d, ^3J = 7.6 Hz, 1H, N$\underline{H}$CH$_{\mathrm{Phe}}$), 8.68 (s, 1H, PhN$\underline{H}$); ^{13}C NMR (125 MHz, DMSO-d_6) δ 22.12, 23.15, 24.30 ($\underline{C}$H($\underline{C}$H$_3$)$_2$), 27.62 (C($\underline{C}$H$_3$)$_3$), 36.82 (CH$\underline{C}$H$_{2,\mathrm{Phe}}$), 42.38 (CH$\underline{C}$H$_{2,\mathrm{Leu}}$), 51.06, 54.22 (NH$\underline{C}$H$_{\mathrm{Phe}}$, NH$\underline{C}$H$_{\mathrm{Leu}}$), 80.68 ($\underline{C}$(CH$_3$)$_3$), 119.08 ($\underline{C}$-2), 124.67 ($\underline{C}$-4), 126.52 ($\underline{C}$-4*), 128.21, 128.62, 129.26 ($\underline{C}$-3, $\underline{C}$-2*, $\underline{C}$-3*), 137.40 ($\underline{C}$-1*), 139.45 ($\underline{C}$-1), 154.52 (NH$\underline{C}$ONH), 170.42, 172.59 (CH$\underline{C}$O); LC/MS (ESI): H_2O/MeOH, 90:10 to 0:100; *m/z* 488.4 $[M + H]^+$; 98% purity; Q-TOF: HRMS (ESI): *m/z* $[M + H]^+$ calcd. for $C_{26}H_{34}ClN_3O_4$: 488.2311, found: 488.2299.

4-Chloroanilinocarbonyl-leucyl-phenylalanine (18)

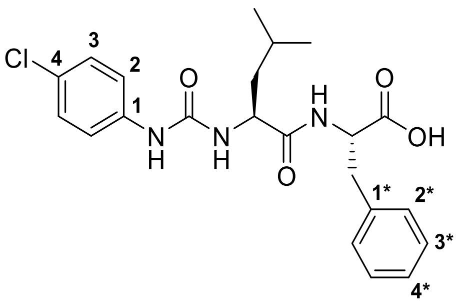

The synthesis was carried out according to GP8 using the corresponding *tert*-butyl ester (98 mg, 0.20 mmol). Column chromatography on silica gel using EtOAc as eluent afforded compound **18** as a white solid (37 mg, 0.09 mmol, 43 %). mp 208–210 °C; ^{1}H NMR (600 MHz, DMSO-d_6) δ 0.87 (2 × d, ^3J = 6.6 Hz, 6H, CH(C$\underline{H}_3$)$_{2,\mathrm{Leu}}$), 1.29–1.34 (m, 1H, CHC$\underline{H}_{2,\mathrm{Leu}}$), 1.39–1.44 (m, 1H, CHC$\underline{H}_{2,\mathrm{Leu}}$), 1.56–1.63 (m, 1H, C$\underline{H}$(CH$_3$)$_{2,\mathrm{Leu}}$), 2.91 (dd, ^2J = 13.9 Hz, ^3J = 9.2 Hz, 1H, CHC$\underline{H}_{2,\mathrm{Phe}}$), 3.05 (dd, ^2J = 13.9 Hz, ^3J = 5.1 Hz, 1H, CHC$\underline{H}_{2,\mathrm{Phe}}$), 4.25 (dt, 1H, ^3J = 5.1 Hz, ^3J = 9.0 Hz, NHC$\underline{H}_{\mathrm{Phe}}$), 4.40–4.44 (m, 1H,

NHC$\underline{H}_{Leu}$), 6.24 (d, ^3J = 8.5 Hz, 1H, N$\underline{H}$CH$_{Leu}$), 7.12–7.17 (m, 1H, 4*-$\underline{H}$), 7.20–7.23 (m, 4H, 2*-$\underline{H}$, 3*-$\underline{H}$), 7.24–7.26 (m, 2H, 3-$\underline{H}$), 7.36–7.39 (m, 2H, 2-$\underline{H}$), 8.32 (d, ^3J = 7.9 Hz, 1H, N$\underline{H}$CH$_{Phe}$), 8.68 (s, 1H, PhN$\underline{H}$), 12.63 (s, 1H, COO$\underline{H}$); ^{13}C NMR (125 MHz, DMSO-*d*6) δ 22.10, 23.23, 24.27 ($\underline{C}$H($\underline{C}$H$_3$)$_2$), 36.70 (CH$\underline{C}$H$_{2,Phe}$), 42.28 (CH$\underline{C}$H$_{2,Leu}$), 51.13, 53.47 (NH$\underline{C}$H$_{Phe}$, NH$\underline{C}$H$_{Leu}$), 119.09 ($\underline{C}$-2), 124.68 ($\underline{C}$-4), 126.48 ($\underline{C}$-4*), 128.24, 128.64, 129.21 ($\underline{C}$-3, $\underline{C}$-2*, $\underline{C}$-3*), 137.71 ($\underline{C}$-1*), 139.47 ($\underline{C}$-1), 154.50 (NH$\underline{C}$ONH), 172.63, 172.87 (CH$\underline{C}$O). LC/MS (ESI): H_2O/MeOH, 90:10 to 0:100; *m/z* 432.1 [M + H]$^+$; 95% purity; Q-TOF: HRMS (ESI): *m/z* [M - H]$^-$ calcd. for $C_{22}H_{26}ClN_3O_4$: 430.1539, found: 430.1557.

4-Chloroanilinocarbonyl-leucyl-proline *tert*-butyl ester

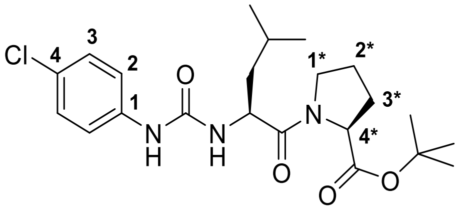

Cbz-Leu-Pro-O*t*Bu (0.63 g, 1.50 mmol) was treated with Pd/C (10 wt%) and hydrogenated for 1h at 30 psi and rt. The reaction mixture was filtrated over celite and MeOH was evaporated. The obtained amine (0.28 g, 1.00 mmol) and 4-chlorophenyl isocyanate (0.15 g, 1.00 mmol) were dissolved in dry CH_2Cl_2 (15 ml) and stirred at rt for 16 h. The solvent was evaporated and the residue was purified by column chromatography on silica gel using petrolum ether / EtOAc (2:1) as eluent to obtain the urea as a white solid (0.42 g, 0.97 mmol, 97 %). mp 63–65 °C; ^{1}H NMR (500 MHz, DMSO-d_6) δ 0.93 (2 × d, ^3J = 6.6 Hz, 6H, CH(C$\underline{H}_3$)$_{2,Leu}$), 1.37 (s, 9H, C(C$\underline{H}_3$)$_3$), 1.38–1.47 (m, 2H, CHC$\underline{H}_{2,Leu}$), 1.67–1.73 (m, 1H, C$\underline{H}$(CH$_3$)$_{2,Leu}$), 1.75–1.81 (m, 1H, 2*-$\underline{H}$), 1.91–1.96 (m, 2H, 2*-$\underline{H}$, 3*-$\underline{H}$), 2.12–2.19 (m, 1H, 3*-$\underline{H}$), 3.49 (dt, ^2J = 9.7 Hz, ^3J = 6.7 Hz, 1H, 1*-$\underline{H}$), 3.71 (dt, ^2J = 9.6 Hz, ^3J = 6.8 Hz, 1H, 1*-$\underline{H}$), 4.19 (dd, ^3J = 5.0 Hz, ^3J = 8.5 Hz, 1H, 4*-$\underline{H}$), 4.48–4.53 (m, 1H, NHC$\underline{H}_{Leu}$), 6.41 (d, ^3J = 8.5 Hz, 1H, N$\underline{H}$CH$_{Leu}$), 7.22–7.25 (m, 2H, 3-$\underline{H}$), 7.36–7.39 (m, 2H, 2-$\underline{H}$), 8.71 (s, 1H, PhN$\underline{H}$); ^{13}C NMR (125 MHz, DMSO-d_6) δ 21.17, 23.39, 24.34 ($\underline{C}$H($\underline{C}$H$_3$)$_2$), 24.69 ($\underline{C}$-2*), 27.73 (C($\underline{C}$H$_3$)$_3$), 28.75 ($\underline{C}$-3*), 41.52 (CH$\underline{C}$H$_{2,Leu}$), 46.59 ($\underline{C}$-1*), 48.99 (NH$\underline{C}$H$_{Leu}$), 59.55 ($\underline{C}$-4*), 80.47 ($\underline{C}$(CH$_3$)$_3$), 119.21 ($\underline{C}$-2), 124.84 ($\underline{C}$-4), 128.67 ($\underline{C}$-3), 139.35 ($\underline{C}$-1), 154.83 (NH$\underline{C}$ONH), 171.01, 171.16 (CH$\underline{C}$O); LC/MS (ESI): H_2O/MeOH, 90:10 to 0:100; *m/z* 438.4 [M + H]$^+$; 97% purity; Q-TOF: HRMS (ESI): *m/z* [M - H]$^-$ calcd. for $C_{22}H_{32}ClN_3O_4$: 436.2009, found: 436.2015.

4-Chloroanilinocarbonyl-leucyl-proline (19)

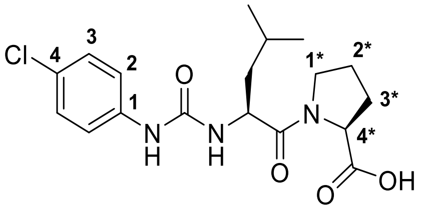

The compound was synthesized *via* solid-phase synthesis as follows. 2-Chlorotrityl resin-bound Fmoc-proline (0.50 mmol) was suspended in DMF (5 mL) and DIPEA (0.26 g, 2.00 mmol). Shaking was carried out continuously at 300 rpm. Fmoc-Leu-OH (1.00 mmol), HBTU (0.38 g, 1.00 mmol) and HOBt (0.15 g, 1.00 mmol) were suspended in DMF (5 mL), treated for 2 min in an ultrasonic bath and added to the resin. After shaking for 2 h, the resin was washed in a standard procedure with DMF (3 × 10 mL), CH_2Cl_2 (3 × 10 mL), MeOH (3 × 10 mL), again CH_2Cl_2 (3 × 10 mL) and finally dried. The Fmoc-protected resin-bound dipeptide was then suspended in DMF (8 mL) and piperidine (2 mL) and shaking was continued for 30 min. The solvent was filtered off and Fmoc-deprotection conditions were repeated once. The resin was washed and dried. The *N*-deprotected resin-bound dipeptide was suspended in CH_2Cl_2 (8 mL) and DIPEA (0.26 g, 2.00 mmol). After 5 min, 4-chlorophenyl isocyanate (0.15 g, 1.00 mmol) was added and the reaction mixture was shaken for 2 h. The solvent was filtered off and the resin was washed and dried. The resin-bound dipeptidic urea was shaken for 1 h in a mixture of TFA, TIPS and CH_2Cl_2 (10 mL, [0.2:0.2:9.6]). The product-containing solution was filtered off and collected and the cleavage conditions were applied to the resin once again. After evaporation of the solvent, the residue was purified by column chromatography on silica gel using EtOAc as eluent plus 1% AcOH as a modifier. The product was obtained as a white solid (175 mg, 92%): mp 95–97 °C.

The synthesis was carried out as well according to GP8 using the corresponding *tert*-butyl ester (131 mg, 0.30 mmol). Column chromatography on silica gel using EtOAc as eluent afforded the product as a white solid (75 mg, 0.20 mmol, 66 %). mp 71–73 °C; ^{1}H NMR (500 MHz, DMSO-d_6) δ 0.92 (2 × d, ^3J = 6.5 Hz, 6H, CH(CH_3)$_{2,Leu}$), 1.34–1.40 (m, 1H, CH$H_{2,Leu}$), 1.41–1.47 (m, 1H, CH$H_{2,Leu}$), 1.66–1.74 (m, 1H, CH(CH_3)$_{2,Leu}$), 1.80–1.86 (m, 1H, 2*-H), 1.90–1.98 (m, 2H, 2*-H, 3*-H), 2.12–2.19 (m, 1H, 3*-H), 3.52 (dt, ^2J = 9.3 Hz, ^3J = 6.6 Hz, 1H, 1*-H), 3.72 (dt, ^2J = 9.7 Hz, ^3J = 7.0 Hz, 1H, 1*-H), 4.27 (dd, ^3J = 4.6 Hz, ^3J = 8.6 Hz, 1H, 4*-H), 4.52 (dt, ^3J = 4.4 Hz, ^3J = 9.1 Hz, 1H, NHCH_{Leu}), 6.39 (d, ^3J = 8.5 Hz, 1H, NHCH_{Leu}), 7.22–7.25 (m, 2H, 3-H), 7.35–7.39 (m, 2H, 2-H), 8.72 (s, 1H, PhNH), one signal (COOH) is not visible; ^{13}C-NMR (125 MHz, DMSO-d_6) δ 21.79, 23.35, 24.20 (CH(CH_3)$_2$), 24.64 (C-2*), 28.70 (C-3*), 41.40 (CH$CH_{2,Leu}$), 46.55 (C-1*), 48.87 (NHCH_{Leu}), 58.52 (C-4*), 119.15 (C-2), 124.77 (C-4), 128.62 (C-3), 139.33 (C-1), 154.74 (NHCONH), 171.18 (CHCO), 173.25 (COOH). LC/MS (ESI): H_2O/MeOH, 90:10 to 0:100; *m/z* 382.2 $[M + H]^+$; 100% purity; Q-TOF: HRMS (ESI): *m/z* $[M - H]^-$ calcd. for $C_{18}H_{24}ClN_3O_4$: 380.1383, found: 380.1401.

7.4. Phenoles and anisol precursors

4-Acetamido-3-chlorophenol (40)

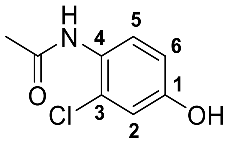

The synthesis was carried out according to GP10 using acetic acid (0.26 g, 4.40 mmol), *N*-methylmorpholine (0.85 g, 8.40 mmol) and isobutyl chloroformate (0.60 g, 4.40 mmol). Column chromatography on silica gel afforded the product as a white solid (0.59 g, 3.17 mmol, 79%). mp 106–108 °C; ^{1}H NMR (500 MHz, DMSO-d_6) δ 2.01 (s, 3H, CH_3), 6.70 (dd, ^3J = 8.7 Hz, ^4J = 2.7 Hz, 1H, 6-H), 6.83 (d, ^4J = 2.8 Hz, 1H, 2-H), 7.29 (d, ^3J = 8.6 Hz, 1H, 5-H), 9.26 (s, 1H, NH), 9.74 (s, 1H, OH); ^{13}C NMR (125 MHz, DMSO-d_6) δ 23.09 (CH_3), 114.47 (C-6), 115.68 (C-2), 126.57 (C-5), 128.60, 128.64 (C-3, C-4), 155.75 (C-1), 168.59 (CH_3CO); LC/MS (ESI): H_2O/MeOH, 90:10 to 0:100; *m/z* 185.9 $[M + H]^+$; 100% purity; $C_8H_8ClNO_2$.

3-Acetamido-4-chlorophenol (41)

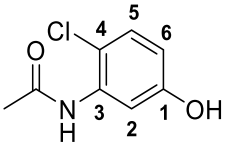

The synthesis was carried out according to GP10 using acetic acid (0.13 g, 2.20 mmol), *N*-methylmorpholine (0.22 g, 2.20 mmol), isobutyl chloroformate (0.30 g, 2.20 mmol) and 3-amino-4-chlorophenol (0.29 g, 2.00 mmol). Column chromatography on silica gel afforded the product as a white solid (0.28 g, 1.51 mmol, 75%). mp 166–168 °C; ^{1}H NMR (500 MHz, DMSO-d_6) δ 2.07 (s, 3H, CH_3), 6.55 (dd, ^3J = 8.7 Hz, ^4J = 2.9 Hz, 1H, 6-H), 7.20 (d, ^3J = 8.8 Hz, 1H, 5-H), 7.26 (s, 1H, 2-H), 9.23 (s, 1H, NH), 9.65 (s, 1H, OH); ^{13}C NMR (125 MHz, DMSO-d_6) δ 23.64 (CH_3), 112.43 (C-2), 113.15 (C-6), 115.21 (C-4), 129.70 (C-5), 135.67 (C-3), 156.50 (C-1), 168.66 (CH_3CO); LC/MS (ESI): H_2O/MeOH, 90:10 to 0:100; *m/z* 186.0 $[M + H]^+$; 100% purity; $C_8H_8ClNO_2$.

3-Chloro-4-phenylacetamidophenol (42)

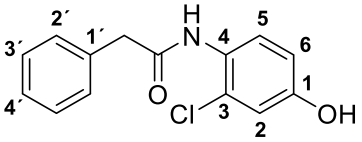

The synthesis was carried out according to GP10 using phenylacetic acid (0.60 g, 4.40 mmol), *N*-methylmorpholine (0.85 g, 8.40 mmol) and isobutyl chloroformate (0.60 g, 4.40 mmol). Column chromatography on silica gel afforded the product as a slightly brown solid (0.48 g, 1.83 mmol, 46%). mp 142–144 °C; ^{1}H NMR (600 MHz, DMSO-d_6) δ 3.63 (s, 2H, CH_2), 6.69 (dd, ^3J = 8.7 Hz, ^4J = 2.7 Hz, 1H, 6-H), 6.83 (d, ^4J = 2.6 Hz, 1H, 2-H), 7.20–7.25 (m, 1H, 5-H), 7.28–7.35 (m, 5H, 2´-H, 3´-H, 4´-H), 9.46 (s, 1H, NH), 9.77 (s, 1H, OH); ^{13}C NMR (150 MHz, DMSO-d_6) δ 42.56 (CH_2), 114.51 (C-6), 115.70 (C-2), 126.32, 126.60 (C-5, C-4´), 128.40 (C-2´), 128.44, 128.56 (C-3, C-4), 129.24 (C-3´), 136.22 (C-1´), 155.83 (C-1), 169.44 (CH_2CO); LC/MS (ESI): H_2O/MeOH, 90:10 to 0:100; *m/z* 262.0 $[M + H]^+$; 100% purity; $C_{14}H_{12}ClNO_2$.

4-Chloro-3-phenylacetamidophenol (43)

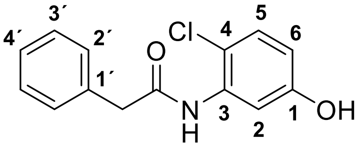

The synthesis was carried out according to GP10 using phenylacetic acid (0.60 g, 4.40 mmol), *N*-methylmorpholine (0.44 g, 4.40 mmol), isobutyl chloroformate (0.60 g, 4.40 mmol) and 3-amino-4-chlorophenol (0.57 g, 4.00 mmol). Recrystallization from EtOAc (5 mL) afforded the product as slightly red crystals (0.62 g, 2.37 mmol, 59%). mp 172–176 °C; ^{1}H NMR (500 MHz, DMSO-d_6) δ 3.72 (s, 2H, C<u>H</u>$_2$), 6.55 (dd, ^3J = 8.8 Hz, ^4J = 2.9 Hz, 1H, 6-<u>H</u>), 7.21 (d, ^3J = 8.7 Hz, 1H, 5-<u>H</u>), 7.23–7.27, 7.30–7.37 (2 × m, 6H, 2-<u>H</u>, 2´-<u>H</u>, 3´-<u>H</u>, 4´-<u>H</u>), 9.36 (s, 1H, N<u>H</u>), 9.65 (s, 1H, O<u>H</u>); ^{13}C NMR (125 MHz, DMSO-d_6) δ 42.90 (<u>C</u>H$_2$), 112.24 (<u>C</u>-2), 113.32 (<u>C</u>-6), 115.22 (<u>C</u>-4), 126.73 (<u>C</u>-4´), 128.48 (<u>C</u>-2´), 129.29 (<u>C</u>-3´), 129.75 (<u>C</u>-5), 135.42, 135.97 (<u>C</u>-3, <u>C</u>-1´), 156.52 (<u>C</u>-1), 169.47 (CH$_2$<u>C</u>O); LC/MS (ESI): H_2O/MeOH, 90:10 to 0:100; *m/z* 262.0 [M + H]$^+$; 100% purity; $C_{14}H_{12}ClNO_2$.

3-Chloro-4-(4-phenylbenzamido)phenol (44)

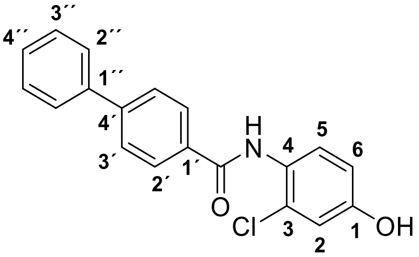

The synthesis was carried out according to GP11 using 4-phenyl benzoic acid (0.79 g, 4.00 mmol), HBTU (1.52 g, 4.00 mmol), DIPEA (1.29 g, 10.0 mmol) and 2-chloro-4-hydroxyanilinium chloride (0.72 g, 4.00 mmol). Recrystallization from EtOAc (10 mL) afforded the product as slightly orange crystals (0.24 g, 0.74 mmol, 19%). mp 220–223 °C (decomposition); ^{1}H NMR (500 MHz, DMSO-d_6) δ 6.78 (dd, ^3J = 8.6 Hz, ^4J = 2.7 Hz, 1H, 6-<u>H</u>), 6.91 (d, ^4J = 2.7 Hz, 1H, 2-<u>H</u>), 7.30 (d, ^3J = 8.6 Hz, 1H, 5-<u>H</u>), 7.39–7.44 (m, 1H, 4´´-<u>H</u>), 7.48–7.52 (m, 2H, 3´´-<u>H</u>), 7.73–7.76 (m, 2H, 2´´-<u>H</u>), 7.80–7.83 (m, 2H, 3´-<u>H</u>), 8.05 (d, ^3J = 8.4 Hz, 2H, 2´-<u>H</u>), 9.87 (s, 2H, N<u>H</u>, O<u>H</u>); ^{13}C NMR (125 MHz, DMSO-d_6) δ 114.60 (<u>C</u>-6), 115.86 (<u>C</u>-2), 126.39 (<u>C</u>-5), 126.75 (<u>C</u>-2´´), 127.04 (<u>C</u>-3´), 128.25 (<u>C</u>-4´´), 128.41 (<u>C</u>-3´´), 129.18 (<u>C</u>-2´), 130.04, 130.82 (C-3, <u>C</u>-4), 133.08 (<u>C</u>-1´), 139.28 (<u>C</u>-1´´), 143.28 (<u>C</u>-4´), 156.59 (<u>C</u>-1), 165.32 (<u>C</u>ONH); LC/MS (ESI): H_2O/MeOH, 90:10 to 0:100; *m/z* 324.1 [M + H]$^+$; 92% purity; $C_{19}H_{14}ClNO_2$.

4-Chloro-3-(4-phenylbenzamido)phenol (45)

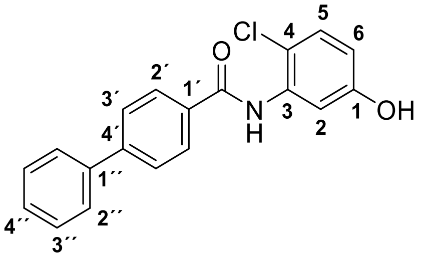

The synthesis was carried out according to GP11 using 4-phenyl benzoic acid (0.79 g, 4.00 mmol), HBTU (1.52 g, 4.00 mmol), DIPEA (0.78 g, 6.00 mmol) and 3-amino-4-chlorophenol (0.57 g, 4.00 mmol). Recrystallization from EtOAc (10 mL) afforded the product as slightly brown crystals (0.36 g, 1.11 mmol, 28%). mp 244–247 °C (decomposition); ^{1}H NMR (500 MHz, DMSO-d_6) δ 6.69 (dd, ^3J = 8.7 Hz, ^4J = 2.9 Hz, 1H, 6-H), 7.13 (d, ^4J = 2.9 Hz, 1H, 2-H), 7.31 (d, ^3J = 8.8 Hz, 1H, 5-H), 7.40–7.44 (m, 1H, 4´´-H), 7.48–7.53 (m, 2H, 3´´-H), 7.73–7.77 (m, 2H, 2´´-H), 7.81–7.85 (m, 2H, 3´-H), 8.04–8.08 (m, 2H, 2´-H), 9.76, 9.84 (2 x s, 2 x 1H, NH, OH); ^{13}C NMR (125 MHz, DMSO-d_6) δ 114.45 (C-2), 114.61 (C-6), 118.37 (C-4), 126.83 (C-2´´), 127.05 (C-3´), 128.30 (C-4´´), 128.47 (C-3´´), 129.19 (C-2´), 129.87 (C-5), 133.00 (C-1´), 135.62 (C-3), 139.22 (C-1´´), 143.49 (C-4´), 156.61 (C-1), 164.98 (CONH); LC/MS (ESI): H_2O/MeOH, 90:10 to 0:100; *m/z* 324.2 [M + H]$^+$; 96% purity; $C_{19}H_{14}ClNO_2$.

3-Chloro-4-(2-diphenylacetamido)phenol (46)

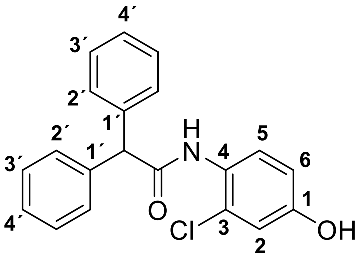

The synthesis was carried out according to GP10 using diphenylacetic acid (0.93 g, 4.40 mmol), *N*-methylmorpholine (0.85 g, 8.40 mmol) and isobutyl chloroformate (0.60 g, 4.40 mmol). Column chromatography on silica gel afforded the product as a slightly brown solid (0.60 g, 1.78 mmol, 44%). mp 172–174 °C; ^{1}H NMR (600 MHz, DMSO-d_6) δ 5.26 (s, 1H, Ph_2CH), 6.70 (dd, ^3J = 8.7 Hz, ^4J = 2.7 Hz, 1H, 6-H), 6.83 (d, ^4J = 2.7 Hz, 1H, 2-H), 7.22–7.26 (m, 2H, 4´-H), 7.28 (d, ^3J = 8.7 Hz, 1H, 5-H), 7.27–7.37 (m, 8H, 2´-H, 3´-H), 9.70 (s, 1H, NH), 9.79 (s, 1H, OH); ^{13}C NMR (150 MHz, DMSO-d_6) δ 56.51 (Ph_2CH), 114.53 (C-6), 115.73 (C-2), 126.09, 126.87 (C-5, C-4´), 128.42 (C-2´), 128.53, 128.78 (C-3, C-4), 128.69 (C-3´), 140.18 (C-1´), 155.97 (C-1), 170.36 (CHCO); LC/MS (ESI): H_2O/MeOH, 90:10 to 0:100; *m/z* 338.0 [M + H]$^+$; 100% purity; $C_{20}H_{16}ClNO_2$.

4-Chloro-3-(2-diphenylacetamido)phenol (47)

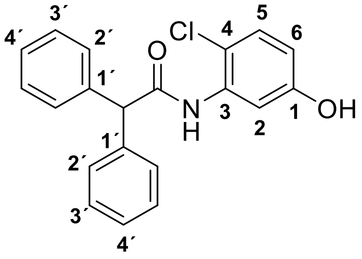

The synthesis was carried out according to GP10 using diphenylacetic acid (0.47 g, 2.20 mmol), *N*-methylmorpholine (0.22 g, 2.20 mmol), isobutyl chloroformate (0.30 g, 2.20 mmol) and 3-amino-4-chlorophenol (0.29 g, 2.00 mmol). Column chromatography on silica gel afforded the product as a slightly brown solid (0.39 g, 1.16 mmol, 58%). mp 180–182 °C; ^{1}H NMR (500 MHz, DMSO-d_6) δ 5.40 (s, 1H, Ph_2CH), 6.57 (dd, ^{3}J = 8.8 Hz, ^{4}J = 2.9 Hz, 1H, 6-H), 7.22 (d, ^{3}J = 8.8 Hz, 1H, 5-H), 7.23–7.28, 7.31–7.38 (2 x m, 11H, 2-H, 2´-H, 3´-H, 4´-H), 9.61 (s, 1H, NH), 9.66 (s, 1H, OH); ^{13}C NMR (125 MHz, DMSO-d_6) δ 56.71 (Ph_2CH), 112.46 (C-2), 113.63 (C-6), 115.58 (C-4), 127.00 (C-4´), 128.53 (C-2´), 128.70 (C-3´), 129.84 (C-5), 135.24 (C-3), 140.01 (C-1´), 156.56 (C-1), 170.39 (CHCO); LC/MS (ESI): H_2O/MeOH, 90:10 to 0:100; *m/z* 338.0 $[M + H]^+$; 100% purity; $C_{20}H_{16}ClNO_2$.

4-Bromo-3-phenylacetamidophenol (48)

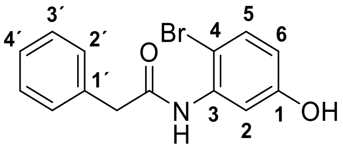

The synthesis was carried out according to GP10 using phenylacetic acid (1.20 g, 8.80 mmol), *N*-methylmorpholine (0.89 g, 8.80 mmol), isobutyl chloroformate (1.20 g, 8.80 mmol) and 3-amino-4-bromophenol (1.50 g, 8.00 mmol). Recrystallization from EtOAc (5 mL) afforded the product as slightly brown crystals (1.46 g, 4.77 mmol, 60%). mp 170–172 °C; ^{1}H NMR (500 MHz, DMSO-d_6) δ 3.70 (s, 2H, CH_2), 6.52 (dd, ^{3}J = 8.7 Hz, ^{4}J = 2.9 Hz, 1H, 6-H), 7.18 (d, ^{4}J = 2.9 Hz, 1H, 2-H), 7.22–7.27 (m, 1H, 5-H), 7.30–7.37 (m, 5H, 2´-H, 3´-H, 4´-H), 9.26 (s, 1H, NH), 9.69 (s, 1H, OH); ^{13}C NMR (125 MHz, DMSO-d_6) δ 42.92 (CH_2), 105.27 (C-2), 113.12 (C-6), 114.25 (C-4), 126.76 (C-4´), 128.50 (C-2´), 129.36 (C-3´), 132.85 (C-5), 135.90 (C-1´), 136.73 (C-3), 157.15 (C-1), 169.36 (CH_2CO); LC/MS (ESI): H_2O/MeOH, 90:10 to 0:100; *m/z* 306.0 $[M + H]^+$; 100% purity; $C_{14}H_{12}BrNO_2$.

4-Phenyl-3-phenylacetamidophenol (49)

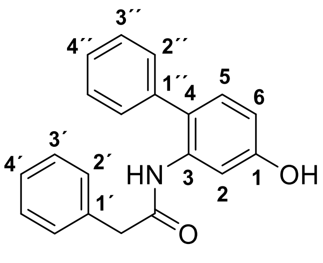

Compound **48** (0.61 g, 2.00 mmol) was suspended in 1,4-dioxane (20 mL) and H_2O (2 mL). Upon addition of $Na_3PO_4 \times 12\ H_2O$ (1.52 g, 4.00 mmol), $Pd[P(C_6H_5)_3]_4$ (0.23 g, 0.20 mmol) and phenylboronic acid (0.49 g, 4.00 mmol), the reaction mixture was stirred under reflux for 8 h. After evaporation of the solvent, the residue was dispensed in EtOAc (50 mL) and H_2O (50 mL). The organic layer was washed with brine (50 mL), dried over Na_2SO_4 and evaporated. The crude product was purified by column chromatography on silica gel using petrolum ether / EtOAc (2:1) as eluent to obtain a slightly yellow solid (0.22 g, 0.72 mmol, 36%). mp 208–211 °C; ^{1}H NMR (500 MHz, DMSO-d_6) δ 3.50 (s, 2H, C<u>H</u>$_2$), 6.65 (dd, ^3J = 8.5 Hz, ^4J = 2.6 Hz, 1H, 6-<u>H</u>), 7.04 (d, ^4J = 2.6 Hz, 1H, 2-<u>H</u>), 7.08 (d, ^3J = 8.3 Hz, 1H, 5-<u>H</u>), 7.17–7.33 (m, 10H, 2´-<u>H</u>, 3´-<u>H</u>, 4´-<u>H</u>, 2´´-<u>H</u>, 3´´-<u>H</u>, 4´´-<u>H</u>), 9.09 (s, 1H, N<u>H</u>), 9.51 (s, 1H, O<u>H</u>); ^{13}C NMR (125 MHz, DMSO-d_6) δ 42.97 (<u>C</u>H$_2$), 112.90 (<u>C</u>-2), 113.09 (<u>C</u>-6), 126.62 (<u>C</u>-4), 126.66 (<u>C</u>-4´´), 126.75 (<u>C</u>-4´), 128.41 (<u>C</u>-2´´), 128.45 (<u>C</u>-3´´), 128.92 (<u>C</u>-2´), 129.24 (<u>C</u>-3´), 131.10 (<u>C</u>-5), 135.57 (<u>C</u>-3), 135.90 (<u>C</u>-1´), 138.86 (<u>C</u>-1´´), 156.95 (<u>C</u>-1), 169.36 (CH$_2$<u>C</u>O); LC/MS (ESI): H_2O/MeOH, 90:10 to 0:100; *m/z* 303.9 $[M + H]^+$; 98% purity; $C_{20}H_{17}NO_2$.

3-Bromo-4-phenylacetamidoanisole

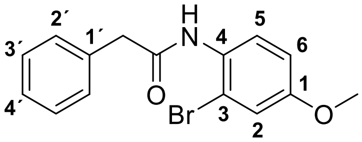

The synthesis was carried out according to GP11 using phenylacetic acid (0.68 g, 5.00 mmol), HBTU (1.90 g, 5.00 mmol), DIPEA (0.97 g, 7.50 mmol) and 3-bromo-4-aminoanisidine (1.01 g, 5.00 mmol). Column chromatography on silica gel using petrolum ether / EtOAc (2.5:1) as eluent and subsequent recrystallization from EtOAc (8 mL) afforded the product as slightly orange crystals (1.10 g, 3.44 mmol, 69%). mp 124–126 °C; ^{1}H NMR (500 MHz, DMSO-d_6) δ 3.65 (s, 2H, C<u>H</u>$_2$), 3.75 (s, 3H, C<u>H</u>$_3$), 6.93 (dd, ^3J = 8.9 Hz, ^4J = 2.9 Hz, 1H, 6-<u>H</u>), 7.20 (d, ^4J = 2.9 Hz, 1H, 2-<u>H</u>), 7.21–7.26 (m, 1H, 4´-<u>H</u>), 7.30–7.37 (m, 4H, 2´-<u>H</u>, 3´-<u>H</u>), 7.38 (d, ^3J = 8.9 Hz, 1H, 5-<u>H</u>), 9.50 (s, 1H, N<u>H</u>); ^{13}C NMR (125 MHz, DMSO-d_6) δ 42.58 (<u>C</u>H$_2$), 55.83 (<u>C</u>H$_3$), 114.07 (<u>C</u>-6), 117.42 (<u>C</u>-2), 119.51 (<u>C</u>-5), 126.63 (<u>C</u>-4´), 128.41 (<u>C</u>-2´), 128.58 (<u>C</u>-3), 129.29 (<u>C</u>-4), 129.32 (<u>C</u>-3´), 136.09 (<u>C</u>-1´), 157.60 (<u>C</u>-1), 169.46 (CH$_2$<u>C</u>O); LC/MS (ESI): H_2O/MeOH, 90:10 to 0:100; *m/z* 319.9 $[M + H]^+$; 96% purity; $C_{15}H_{14}BrNO_2$.

3-Bromo-4-phenylacetamidophenol (50)

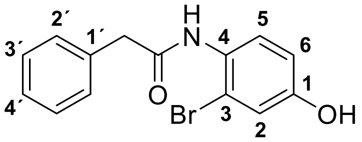

The synthesis was carried out according to GP12 using the corresponding anisole (0.64 g, 2.00 mmol) and BBr_3 (1.50 g, 6.00 mmol). Column chromatography on silica gel using petrolum ether / EtOAc (2:1) as eluent afforded the product as a slightly brown solid (0.44 g, 1.44 mmol, 72%). mp 140–142 °C; ^{1}H NMR (500 MHz, DMSO-d_6) δ 3.62 (s, 2H, C<u>H</u>$_2$), 6.73 (dd, ^3J = 8.7 Hz, ^4J = 2.7, 1H, 6-<u>H</u>), 6.99 (d, ^4J =

2.7 Hz, 1H, 2-H), 7.21–7.25, 7.29–7.36 (2 × m, 6H, 5-H, 2´-H, 3´-H, 4´-H), 9.41 (s, 1H, NH), 9.76 (s, 1H, OH); ^{13}C NMR (125 MHz, DMSO-*d*6) δ 42.56 (CH_2), 115.06 (C-6), 118.72 (C-2), 119.54 (C-5), 126.60 (C-4´), 127.72 (C-3), 128.39 (C-2´), 128.89 (C-4), 129.30 (C-3´), 136.16 (C-1´), 156.09 (C-1), 169.42 (CHCO); LC/MS (ESI): H_2O/MeOH, 90:10 to 0:100; *m/z* 306.0 $[M + H]^+$; 98% purity; $C_{14}H_{12}BrNO_2$.

3-Bromo-4-(2-diphenylacetamido)anisole

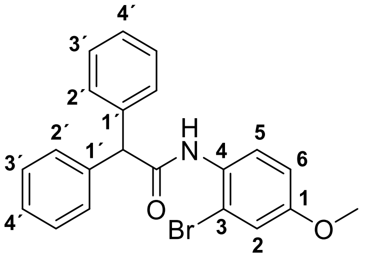

The synthesis was carried out according to GP11 using diphenylacetic acid (0.70 g, 3.30 mmol), HBTU (1.25 g, 3.30 mmol), DIPEA (0.43 g, 3.30 mmol) and 3-bromo-4-aminoanisidine (0.61 g, 3.00 mmol). Column chromatography on silica gel using petrolum ether / CH_2Cl_2 (1:1) as eluent afforded the product as a slightly brown solid (0.74 g, 1.88 mmol, 58%). mp 139–141 °C; ^{1}H NMR (500 MHz, DMSO-*d*6) δ 3.75 (s, 3H, CH_3), 5.27 (s, 1H, Ph_2CH), 6.93 (dd, ^3J = 8.9 Hz, ^4J = 2.9 Hz, 1H, 6-H), 7.20 (d, ^4J = 2.8 Hz, 1H, 2-H), 7.23–7.27 (m, 2H, 4´-H), 7.31–7.40 (m, 9H, 5-H, 2´-H, 3´-H), 9.73 (s, 1H, NH); ^{13}C NMR (125 MHz, DMSO-*d*6) δ 55.84 (CH_3), 56.58 (Ph_2CH), 114.10 (C-6), 117.45 (C-2), 119.67 (C-5), 126.90 (C-4´), 128.44 (C-2´), 128.67 (C-3), 128.74 (C-3´), 129.07 (C-4), 140.10 (C-1´), 157.73 (C-1), 170.39 (CHCO); LC/MS (ESI): H_2O/MeOH, 90:10 to 0:100; *m/z* 391.1 $[M + H]^+$; 100% purity; $C_{21}H_{18}BrNO_2$.

3-Bromo-4-(2-diphenylacetamido)phenol (51)

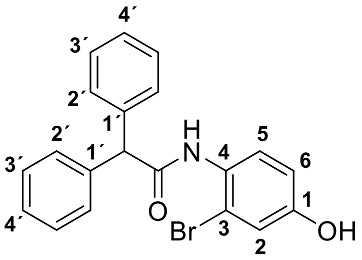

The synthesis was carried out according to GP12 using the corresponding anisole (0.20 g, 0.50 mmol) and BBr_3 (0.38 g, 1.50 mmol). Column chromatography on silica gel using petrolum ether / EtOAc (2:1) as eluent afforded the product as a slightly brown solid (0.13 g, 0.34 mmol, 69%). mp 165–169 °C; ^{1}H NMR (500 MHz, DMSO-*d*6) δ 5.24 (s, 1H, Ph_2CH), 6.74 (dd, ^3J = 8.7 Hz, ^4J = 2.7 Hz, 1H, 6-H), 6.99 (d, ^4J = 2.7 Hz, 1H, 2-H), 7.21 (d, ^3J = 8.7 Hz, 1H, 5-H), 7.23–7.26 (m, 2H, 4´-H), 7.30–7.37 (m, 8H, -2´-H, 3´-H), 9.64 (s, 1H, NH), 9.79 (s, 1H, OH); ^{13}C NMR (125 MHz, DMSO-*d*6) δ 56.57 (Ph_2CH), 115.10 (C-6), 118.76 (C-2), 119.67 (C-5), 126.88 (C-4´), 127.51 (C-3), 128.43 (C-2´), 128.74 (C-3´),

128.97 (C-4), 140.17 (C-1´), 156.23 (C-1), 170.37 (CHCO); LC/MS (ESI): H_2O/MeOH, 90:10 to 0:100; *m/z* 381.9 $[M + H]^+$; 94% purity; $C_{20}H_{16}BrNO_2$.

3-Bromo-4-(3,3-dibenzylureido)anisole

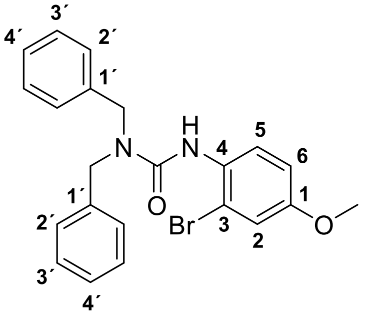

3-Bromo-4-aminoanisole (0.81 g, 4.00 mmol) was dissolved in CH_2Cl_2 (50 mL) and cooled to 0 °C. Upon addition of DIPEA (0.87 g, 4.40 mmol) and 4-nitrophenyl chloroformate (0.89 g, 4.40 mmol), the solution was stirred for 30 min. After addition of dibenzylamine (0.87 g, 4.40 mmol), the reaction mixture was stirred for 4 h whilst warming to rt. The reaction mixture was washed with 10% $KHSO_4$ (3 × 50 mL) and the organic layer was dried over Na_2SO_4 and evaporated. The crude product was purified by column chromatography on silica gel using petrolum ether / EtOAc (6:1) as eluent to obtain a brown solid (1.18 g, 2.77 mmol, 69%). mp 98–102 °C; ^{1}H NMR (600 MHz, $CDCl_3$) δ 3.72 (s, 3H, CH_3), 4.63 (s, 4H, $N(CH_2)_2$), 6.65 (s, 1H, NH), 6.83 (dd, ^3J = 9.0 Hz, ^4J = 2.8 Hz, 1H, 6-H), 6.95 (d, ^4J = 2.7 Hz, 1H, 2-H), 7.28 (t, ^3J = 7.1 Hz, 2H, 4´-H), 7.32 (d, ^3J = 7.2 Hz, 4H, 2´-H), 7.35 (t, ^3J = 7.5 Hz, 4H, 3´-H), 7.99 (d, ^3J = 9.0 Hz, 1H, 5-H); ^{13}C NMR (150 MHz, $CDCl_3$) δ 50.76 ($N(CH_2)_2$), 55.71 (CH_3), 113.88 (C-6), 114.24 (C-2), 117.15 (C-5), 123.14 (C-3), 127.35 (C-2´), 127.73 (C-4´), 128.91 (C-3´), 130.46 (C-4), 136.95 (C-1´), 155.54 (CONH), 155.78 (C-1); LC/MS (ESI): H_2O/MeOH, 90:10 to 0:100; *m/z* 425.1 $[M + H]^+$; 99% purity; $C_{22}H_{21}BrN_2O_2$.

3-Bromo-4-(3,3-dibenzylureido)phenol (52)

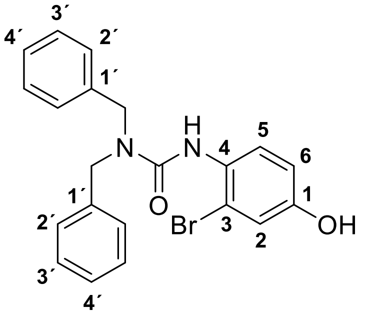

The synthesis was carried out according to GP12 using the corresponding anisole (0.64 g, 1.50 mmol) and BBr_3 (1.13 g, 4.50 mmol). Column chromatography on silica gel using petrolum ether / EtOAc (4:1) as eluent afforded the product as a brown solid (0.27 g, 0.66 mmol, 44%). mp 198–205 °C (decomposition); ^{1}H NMR (500 MHz, DMSO-d_6) δ 4.50 (s, 4H, $N(CH_2)_2$), 6.74 (dd, ^3J = 8.7 Hz, ^4J = 2.7 Hz, 1H, 6-H), 6.97 (d, ^4J = 2.7 Hz, 1H, 2-H), 7.23 (d, ^3J = 8.7 Hz, 1H, 5-H), 7.24–7.31 (m, 6H, 2´-H,

4´-H), 7.32–7.37 (m, 4H, 3´-H), 7.97 (s, 1H, NH), 9.65 (s, 1H, OH); ^{13}C NMR (125 MHz, DMSO-d_6) δ 49.16 ($\underline{C}H_2$), 114.93 (C-6), 118.53 (C-2), 120.99 (C-5), 127.15 (C-4´), 127.47 (C-2´), 128.56 (C-3´), 129.37 (C-3), 129.42 (C-4), 138.21 (C-1´), 155.58 (CONH), 156.17 (C-1); LC/MS (ESI): H_2O/MeOH, 90:10 to 0:100; *m/z* 411.3 $[M + H]^+$; 91% purity; $C_{21}H_{19}BrN_2O_2$.

3-Chloro-4-(2-dibenzylacetamido)anisole

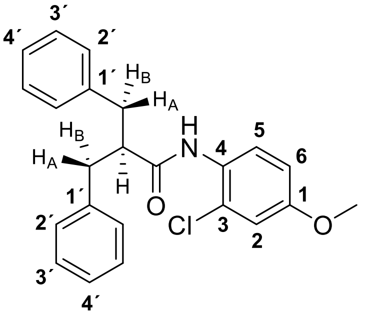

2-Benzyl-3-phenylpropionic acid (0.43 g, 1.80 mmol), EDC × HCl (0.38 g, 2.00 mmol), DMAP (24 mg, 0.20 mmol) and DIPEA (0.52 g, 4.00 mmol) were dissolved in dry CH_2Cl_2 (40 ml) and stirred at rt for 1 h. Upon addition of 2-chloro-4-anisidine (0.28 g, 1.80 mmol), the reaction mixture was stirred at rt for 16 h. After addition of CH_2Cl_2 (40 mL), the organic layer was washed with 10% $KHSO_4$ (3 × 40 ml) and dried over Na_2SO_4. After evaporation of the solvent, the crude product was purified by Column chromatography on silica gel using CH_2Cl_2 as eluent to obtain a brown solid (0.22 g, 0.58 mmol, 32%). mp 122–124 °C; ^{1}H NMR (600 MHz, DMSO-d_6) δ 2.71 (dd, ^2J = 13.6 Hz, ^3J = 5.7 Hz, 2H, $CH(C\underline{H}_AH_B)_2$), 2.94 (dd, ^2J = 13.5 Hz, ^3J = 9.0 Hz, 2H, $CH(CH_A\underline{H}_B)_2$), 3.14–3.18 (m, 1H, $C\underline{H}(CH_2)_2$), 3.71 (s, 3H, $OC\underline{H}_3$), 6.81 (dd, ^3J = 8.9 Hz, ^4J = 2.8 Hz, 1H, 6-H), 6.95 (d, ^4J = 2.8 Hz, 1H, 2-H), 7.02 (d, ^3J = 8.9 Hz, 1H, 5-H), 7.15–7.19 (m, 2H, 4´-H), 7.20–7.24 (m, 4H, 2´-H), 7.24–7.29 (m, 4H, 3´-H), 9.18 (s, 1H, NH); ^{13}C NMR (125 MHz, DMSO-d_6) δ 38.31 ($CH(\underline{C}H_AH_B)_2$), 49.08 ($\underline{C}H(CH_AH_B)_2$), 55.76 ($O\underline{C}H_3$), 113.27 (C-6), 114.27 (C-2), 126.16 (C-4´), 127.69 (C-5), 128.28 (C-2´), 128.40 (C-4), 128.99 (C-3), 129.03 (C-3´), 139.67 (C-1´), 157.37 (C-1), 172.95 (CHCO); LC/MS (ESI): H_2O/MeOH, 90:10 to 0:100; *m/z* 379.9 $[M + H]^+$; 100% purity; $C_{23}H_{22}ClNO_2$.

3-Chloro-4-(2-dibenzylacetamido)phenol (53)

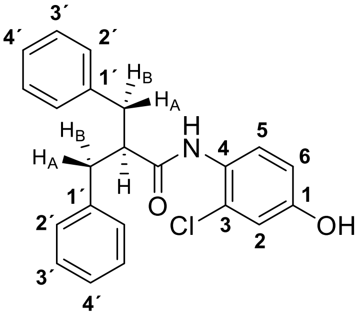

The synthesis was carried out according to GP12 using the corresponding anisole (0.19 g, 0.50 mmol) and BBr_3 (0.38 g, 1.50 mmol). Column chromatography on silica gel using petrolum ether / EtOAc (2:1)

as eluent afforded the product as a slightly brown resin (0.14 g, 0.38 mmol, 77%). ^{1}H NMR (600 MHz, $CDCl_3$) δ 2.73–2.82 (m, 1H, CH(CH_2)$_2$), 2.90 (dd, ^{2}J = 13.5 Hz, ^{3}J = 5.2 Hz, , 2H, CH(CH_AH_B)$_2$), 3.06 (dd, ^{2}J = 13.5 Hz, ^{3}J = 9.7 Hz, 2H, CH(CH_AH_B)$_2$), 6.44 (dd, ^{3}J = 8.8 Hz, ^{4}J = 2.5 Hz, 1H, 6-H), 6.57 (d, ^{4}J = 2.5 Hz, 1H, 2-H), 6.72 (s, 1H, NH), 7.11 (d, ^{3}J = 8.7 Hz, 1H, 5-H), 7.17–7.22 (m, 6H, 2´-H, 4´-H), 7.27 (t, ^{3}J = 7.5 Hz, 4H, 3´-H); ^{13}C NMR (150 MHz, $CDCl_3$) δ 39.09 (CH(CH_AH_B)$_2$), 53.31 (CH(CH_AH_B)$_2$), 114.84 (C-6), 116.27 (C-2), 125.20 (C-5), 126.08 (C-3), 126.59 (C-4´), 127.50 (C-4), 128.64 (C-2´), 128.90 (C-3´), 139.04 (C-1´), 154.77 (C-1), 173.84 (CHCO); LC/MS (ESI): H_2O/MeOH, 90:10 to 0:100; *m/z* 365.8 $[M + H]^+$; 98% purity; $C_{22}H_{20}ClNO_2$.

7.5. Dipeptide carbamates of type C

4-Acetamidophenoxycarbonyl-leucyl-proline *tert*-butyl ester

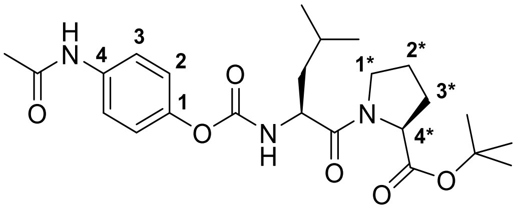

The synthesis was carried out according to GP13 using Cbz-Leu-Pro-O*t*Bu (0.31 g) for the hydrogenation and commercially available 4-acetamidophenol (76 mg) for the subsequent carbamate formation. Column chromatography on silica gel using CH_2Cl_2 / acetone (2:1) as eluent afforded the product as a white solid (110 mg, 0.24 mmol, 48%). mp 158–163 °C; ^{1}H NMR (500 MHz, DMSO-d_6) δ 0.92 (d, ^{3}J = 6.6 Hz, 6H, CH(CH_3)$_{2,Leu}$), 1.37 (s, 9H, C(CH_3)$_3$), 1.39–1.44 (m, 1H, CH$CH_{2,Leu}$), 1.53–1.59 (m, 1H, CH$CH_{2,Leu}$), 1.74–1.81 (m, 2H, CH(CH_3)$_{2,Leu}$, 2*-H), 1.90–1.94 (m, 2H, 2*-H, 3*-H), 2.02 (s, 3H, CH_3CO), 2.13–2.17 (m, 1H, 3*-H), 3.48 (dt, ^{2}J = 9.7 Hz, ^{3}J = 7.0 Hz, 1H, 1*-H), 3.66 (dt, ^{2}J = 9.6 Hz, ^{3}J = 6.9 Hz, 1H, 1*-H), 4.19 (dd, ^{3}J = 8.6 Hz, ^{3}J = 4.9 Hz, 1H, 4*-H), 4.27–4.32 (m, 1H, NHCH_{Leu}), 6.96–6.98 (m, 2H, 2-H), 7.51–7.53 (m, 2H, 3-H), 7.93 (d, ^{3}J = 8.0 Hz, 1H, OCONH), 9.90 (s, 1H, NHPh); ^{13}C NMR (150 MHz, DMSO-d_6) δ 21.45, 23.24, 24.00, 24.32, (CH(CH_3)$_{2,Leu}$, CH_3CO), 24.73 (C-2*), 27.70 (C(CH_3)$_3$), 28.65 (C-3*), 40.22 (CH$CH_{2,Leu}$), 46.48 (C-1*), 51.10 (NHCH_{Leu}), 59.59 (C-4*), 80.40 (C(CH_3)$_3$), 119.86 (C-2), 121.87 (C-3), 136.40 (C-4), 146.34 (C-1), 154.68 (OCONH), 168.23 (CH_3CO), 170.47, 171.02 (CHCO); LC/MS (ESI): H_2O/MeCN, 90:10 to 0:100; *m/z* 461.9 $[M + H]^+$; 99% purity; Q-TOF: HRMS (ESI): *m/z* $[M + H]^+$ calcd. for $C_{24}H_{35}N_3O_6$: 462.2599, found: 462.2567.

4-Acetamidophenoxycarbonyl-leucyl-proline

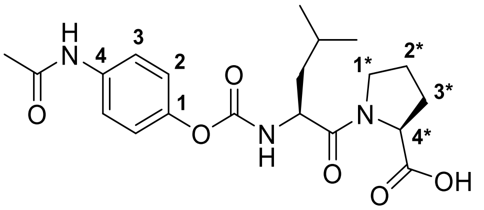

The synthesis was carried out according to GP8 using the corresponding *tert*-butyl ester (92 mg, 0.20 mmol). Column chromatography on silica gel was not required to obtain the desired compound as a colorless resin (83 mg, 0.20 mmol, 100%). ^{1}H NMR (500 MHz, DMSO-d_6) δ 0.92 (2 × d, ^3J = 6.7 Hz, 6H, CH(C**H**$_3$)$_{2,\mathrm{Leu}}$), 1.40–1.45 (m, 1H, CHC**H**$_{2,\mathrm{Leu}}$), 1.50–1.54 (m, 1H, CHC**H**$_{2,\mathrm{Leu}}$), 1.71–1.76 (m, 1H, C**H**(CH$_3$)$_{2\mathrm{Leu}}$), 1.80–1.85 (m, 1H, 2*-**H**), 1.90–1.94 (m, 2H, 2*-**H**, 3*-**H**), 2.01 (s, 3H, C**H**$_3$CO), 2.12–2.16 (m, 1H, 3*-**H**), 3.50 (dt, ^2J = 9.7 Hz, ^3J = 6.6 Hz, 1H, 1*-**H**), 3.68 (dt, ^2J = 9.8 Hz, ^3J = 6.9 Hz, 1H, 1*-**H**), 4.26 (dd, ^3J = 4.8 Hz, ^3J = 8.7 Hz, 1H, 4*-**H**), 4.29–4.32 (m, 1H, NHC**H**$_{\mathrm{Leu}}$), 6.96 – 6.99 (m, 2H, 2-**H**), 7.51 – 7.53 (m, 2H, 3-**H**), 7.93 (d, ^3J = 7.9 Hz, 1H, OCON**H**), 9.90 (s, 1H, N**H**Ph), one signal (COO**H**) is not visible; ^{13}C NMR (125 MHz, DMSO-d_6) δ 21.56, 23.23, 24.00, 24.24 (**C**H(**C**H$_3$)$_{2,\mathrm{Leu}}$, **C**H$_3$CO), 24.73 (**C**-2*), 28.65 (**C**-3*), 40.28 (CH**C**H$_{2,\mathrm{Leu}}$), 46.49 (**C**-1*), 51.03 (NH**C**H$_{\mathrm{Leu}}$), 58.66 (**C**-4*), 119.86 (**C**-2), 121.87 (**C**-3), 136.40 (**C**-4), 146.33 (**C**-1), 154.64 (O**C**ONH), 168.22 (CH$_3$**C**O), 170.48 (CH**C**O), 173.29 (**C**OOH); LC/MS (ESI): H_2O/MeCN, 90:10 to 0:100; *m/z* 406.2 [M + H]$^+$; 100% purity; Q-TOF: HRMS (ESI): *m/z* [M + H]$^+$ calcd. for $C_{20}H_{27}N_3O_6$: 406.1973, found: 406.1956.

3-Acetamidophenoxycarbonyl-leucyl-proline *tert*-butyl ester

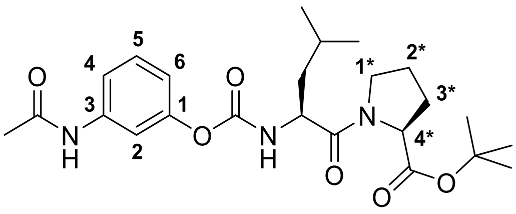

The synthesis was carried out according to GP13 using Cbz-Leu-Pro-O*t*Bu (0.31 g) for the hydrogenation and commercially available 3-acetamidophenol (76 mg) for the subsequent carbamate formation. Column chromatography on silica gel using CH_2Cl_2 / acetone (3:1) as eluent afforded the product as a colorless resin (127 mg, 0.28 mmol, 55%). ^{1}H NMR (600 MHz, DMSO-d_6) δ 0.93 (d, ^3J = 6.6 Hz, 6H, CH(C**H**$_3$)$_{2,\mathrm{Leu}}$), 1.37 (s, 9H, C(C**H**$_3$)$_3$), 1.40–1.44 (m, 1H, CHC**H**$_{2,\mathrm{Leu}}$), 1.54–1.59 (m, 1H, CHC**H**$_{2,\mathrm{Leu}}$), 1.75–1.80 (m, 2H, C**H**(CH$_3$)$_{2,\mathrm{Leu}}$, 2*-**H**), 1.89–1.94 (m, 2H, 2*-**H**, 3*-**H**), 2.02 (s, 3H, C**H**$_3$CO), 2.12–2.17 (m, 1H, 3*-**H**), 3.48 (dt, ^2J = 9.6 Hz, ^3J = 6.7 Hz, 1H, 1*-**H**), 3.66 (dt, ^2J = 9.8 Hz, ^3J = 6.9 Hz, 1H, 1*-**H**), 4.20 (dd, ^3J = 5.0 Hz, ^3J = 8.6 Hz, 1H, 4*-**H**), 4.28–4.31 (m, 1H, NHC**H**$_{\mathrm{Leu}}$), 6.71–6.73 (m, 1H, 6-**H**), 7.24 (t, ^3J = 8.1 Hz, 1H, 5-**H**), 7.32 (d, ^3J = 8.3 Hz, 1H, 4-**H**), 7.40 (t, ^3J = 2.1 Hz, 1H, 2-**H**), 8.00 (d, ^3J = 8.0 Hz, 1H, OCON**H**), 9.99 (s, 1H, N**H**Ph); ^{13}C NMR (150 MHz, $CDCl_3$) δ 21.60, 23.46, 24.52, 24.61 (**C**H(**C**H$_3$)$_{2,\mathrm{Leu}}$, **C**H$_3$CO), 24.81 (**C**-2*), 27.95 (C(**C**H$_3$)$_3$), 29.04 (**C**-3*), 42.01 (CH**C**H$_{2,\mathrm{Leu}}$), 46.84 (**C**-1*), 51.08 (NH**C**H$_{\mathrm{Leu}}$), 59.70 (**C**-4*), 81.46 (**C**(CH$_3$)$_3$), 113.26 (**C**-2), 116.67 (**C**-4), 117.16 (**C**-6), 129.47 (**C**-5), 138.96 (**C**-3), 151.16 (**C**-1), 154.72 (O**C**ONH), 168.31 (CH$_3$**C**O), 170.92, 170.94 (CH**C**O); LC/MS (ESI): H_2O/MeCN, 90:10 to 0:100; *m/z* 462.1 [M + H]$^+$; 100% purity; Q-TOF: HRMS (ESI): *m/z* [M + H]$^+$ calcd. for $C_{24}H_{35}N_3O_6$: 462.2599, found: 462.2583.

3-Acetamidophenoxycarbonyl-leucyl-proline

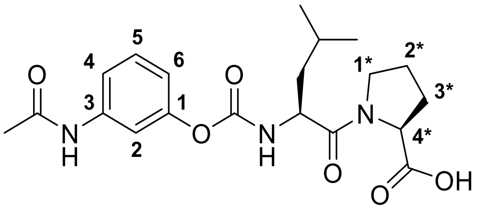

The synthesis was carried out according to GP8 using the corresponding *tert*-butyl ester (138 mg, 0.30 mmol). Column chromatography on silica gel afforded the desired compound as a colorless resin (62 mg, 0.15 mmol, 50%). ^{1}H NMR (500 MHz, DMSO-d_6) δ 0.92 (2 × d, ^3J = 6.7 Hz, 6H, CH(CH_3)$_{2,Leu}$), 1.40–1.45 (m, 1H, CHC$H_{2,Leu}$), 1.51–1.57 (m, 1H, CHC$H_{2,Leu}$), 1.71–1.77 (m, 1H, CH(CH$_3$)$_{2,Leu}$), 1.80–1.85 (m, 1H, 2*-H), 1.90–1.94 (m, 2H, 2*-H, 3*-H), 2.02 (s, 3H, CH_3CO), 2.12–2.16 (m, 1H, 3*-H), 3.48–3.52 (m, 1H, 1*-H), 3.65–3.70 (m, 1H, 1*-H), 4.26–4.33 (m, 2H, NHCH_{Leu}, 4*-H), 6.73 (dd, ^3J = 8.0 Hz, ^4J = 2.3 Hz, 1H, 6-H), 7.23 (t, ^3J = 8.1 Hz, 1H, 5-H), 7.33 (d, ^3J = 8.0 Hz, 1H, 4-H), 7.40 (t, ^4J = 2.0 Hz, 1H, 2-H), 7.98 (d, ^3J = 7.9 Hz, 1H, OCONH), 9.99 (s, 1H, NHPh), one signal (COOH) is not visible; ^{13}C NMR (125 MHz, DMSO-d_6) δ 21.59, 23.29, 24.17, 24.33 (CH(CH$_3$)$_{2,Leu}$, CH$_3$CO), 24.80 (C-2*), 28.71 (C-3*), 46.56 (C-1*), 51.11 (NHCH$_{Leu}$), 58.73 (C-4*), 112.46 (C-2), 115.59 (C-4), 116.27 (C-6), 129.36 (C-5), 140.33 (C-3), 151.30 (C-1), 154.43 (OCONH), 168.65 (CH$_3$CO), 170.54 (CHCO), 173.36 (COOH), one signal (CHCH$_{2,Leu}$) is obscured by the DMSO solvent signal; LC/MS (ESI): H_2O/MeCN, 90:10 to 0:100; *m/z* 406.2 [M + H]$^+$; 100% purity; Q-TOF: HRMS (ESI): *m/z* [M - H]$^-$ calcd. for $C_{24}H_{27}N_3O_6$: 404.1827, found: 404.1833.

4-Acetamido-3-chlorophenoxycarbonyl-leucyl-proline *tert*-butyl ester

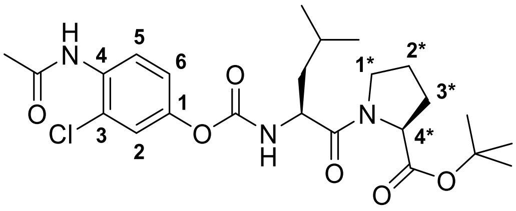

The synthesis was carried out according to GP13 using Cbz-Leu-Pro-O*t*Bu (0.31 g) for the hydrogenation and phenol **40** (93 mg) for the subsequent carbamate formation. Column chromatography on silica gel using CH_2Cl_2 / acetone (3:1) as eluent afforded the product as a colorless resin (106 mg, 0.21 mmol, 43%). ^{1}H NMR (600 MHz, DMSO-d_6) δ 0.88 (2 × d, ^3J = 6.7 Hz, 6H, CH(CH_3)$_{2Leu}$), 1.36 (s, 9H, C(CH_3)$_3$), 1.60–1.64 (m, 1H, CH(CH$_3$)$_{2,Leu}$), 1.74–1.79 (m, 2H, CHC$H_{2,Leu}$), 1.87–1.95 (m, 3H, 2*-H, 3*-H), 2.00 (s, 3H, CH_3CO), 2.12–2.15 (m, 1H, 3*-H), 3.44 (dt, ^2J = 9.8 Hz, ^3J = 6.9 Hz, 1H, 1*-H), 3.67 (dt, ^2J = 9.5 Hz, ^3J = 6.7 Hz, 1H, 1*-H), 4.15 (dd, ^3J = 5.0 Hz, ^3J = 8.5 Hz, 1H, 4*-H), 4.36–4.40 (m, 1H, NHCH_{Leu}), 6.19 (d, ^3J = 8.7 Hz, 1H, 5-H), 6.69 (dd, ^3J = 8.8 Hz, ^4J = 2.8 Hz, 1H, 6-H), 6.82 (d, ^4J = 2.8 Hz, 1H, 2-H), 7.29 (d, ^3J = 8.7 Hz, 1H, OCONH), 9.26 (s, 1H, NHPh); ^{13}C NMR (150 MHz, DMSO-d_6) δ 21.85, 23.07, 23.30, 24.18 (CH(CH$_3$)$_{2,Leu}$, CH$_3$CO), 24.66 (C-2*), 27.69 (C(CH$_3$)$_3$), 28.70 (C-3*), 41.53 (CHCH$_{2,Leu}$), 46.44 (C-1*), 49.05 (NHCH$_{Leu}$), 59.46 (C-4*), 80.32 (C(CH$_3$)$_3$),

114.44 (C-6), 115.64 (C-2), 126.52 (C-5), 128.57, 128.60 (C-3, C-4), 155.74 (OCONH), 157.25 (C-1), 168.53 (CH_3CO), 171.05, 171.44 (CHCO); LC/MS (ESI): H_2O/MeCN, 90:10 to 0:100; *m/z* 496.2 $[M + H]^+$; 99% purity; Q-TOF: HRMS (ESI): *m/z* $[M + H]^+$ calcd. for $C_{24}H_{34}ClN_3O_6$: 496.2209, found: 496.2140.

4-Acetamido-3-chlorophenoxycarbonyl-leucyl-proline

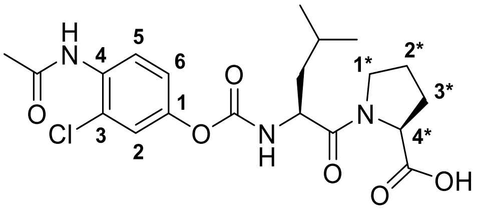

The synthesis was carried out according to GP8 using the corresponding *tert*-butyl ester (74 mg, 0.15 mmol). Column chromatography on silica gel afforded the desired compound as a colorless resin (43 mg, 0.10 mmol, 65%). ^{1}H NMR (500 MHz, DMSO-d_6) δ 0.92 (2 × d, ^3J = 6.6 Hz, 6H, $CH(CH_3)_{2,Leu}$), 1.40–1.46 (m, 1H, $CHCH_{2,Leu}$), 1.51–1.57 (m, 1H, $CHCH_{2,Leu}$), 1.71–1.77 (m, 1H, $CH(CH_3)_{2,Leu}$), 1.79–1.85 (m, 1H, 2*-H), 1.91–1.95 (m, 2H, 2*-H, 3*-H), 2.07 (s, 3H, CH_3CO), 2.12–2.16 (m, 1H, 3*-H), 3.48–3.52 (m, 1H, 1*-H), 3.68 (dt, ^2J = 9.6 Hz, ^3J = 6.9 Hz, 1H, 1*-H), 4.26 (dd, ^3J = 4.8 Hz, ^3J = 8.7 Hz, 1H, 4*-H), 4.29–4.33 (m, 1H, $NHCH_{Leu}$), 7.04 (dd, ^3J = 8.8 Hz, ^4J = 2.7 Hz, 1H, 6-H), 7.25 (d, ^4J = 2.6 Hz, 1H, 2-H), 7.61 (d, ^3J = 8.7 Hz, 1H, 5-H), 8.11 (d, ^3J = 7.9 Hz, 1H, OCONH), 9.50 (s, 1H, NHPh), one signal (COOH) is not visible; ^{13}C NMR (125 MHz, DMSO-d_6) δ 21.56, 23.24, 23.32, 24.25 ($CH(CH_3)_{2,Leu}$, CH_3CO), 24.75 (C-2*), 28.67 (C-3*), 40.29 ($CHCH_{2,Leu}$), 46.52 (C-1*), 51.16 ($NHCH_{Leu}$), 58.70 (C-4*), 120.87 (C-6), 122.68 (C-2), 127.05 (C-3), 127.10 (C-5), 132.28 (C-4), 148.18 (C-1), 154.12 (OCONH), 168.75 (CH_3CO), 172.09 (CHCO), 173.29 (COOH); LC/MS (ESI): H_2O/MeCN, 90:10 to 0:100; *m/z* 440.3 $[M + H]^+$; 100% purity; Q-TOF: HRMS (ESI): *m/z* $[M - H]^-$ calcd. for $C_{20}H_{26}ClN_3O_6$: 438.1437, found: 438.1443.

3-Acetamido-4-chlorophenoxycarbonyl-leucyl-proline *tert*-butyl ester

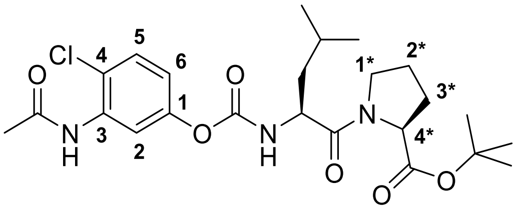

The synthesis was carried out according to GP13 using Cbz-Leu-Pro-O*t*Bu (0.31 g) for the hydrogenation and phenol **41** (93 mg) for the subsequent carbamate formation. Column chromatography on silica gel using petrolum ether / EtOAc (1:1) as eluent afforded the product as a colorless resin (126 mg, 0.25 mmol, 51%). ^{1}H NMR (600 MHz, DMSO-d_6) δ 0.92 (d, ^3J = 6.6 Hz, 6H, $CH(CH_3)_{2,Leu}$), 1.37 (s, 9H, $C(CH_3)_3$), 1.41–1.45 (m, 1H, $CHCH_{2,Leu}$), 1.53–1.60 (m, 1H, $CHCH_{2,Leu}$), 1.72–1.81 (m, 2H, $CH(CH_3)_{2,Leu}$, 2*-H), 1.89–1.93 (m, 2H, 2*-H, 3*-H), 2.09 (s, 3H, CH_3CO), 2.12–2.17 (m, 1H, 3*-H),

3.48 (dt, ^2J = 9.6 Hz, ^3J = 6.9 Hz, 1H, 1*-H), 3.66 (dt, ^2J = 9.5 Hz, ^3J = 6.9 Hz, 1H, 1*-H), 4.20 (dd, ^3J = 5.0 Hz, ^3J = 8.6 Hz, 1H, 4*-H), 4.26–4.33 (m, 1H, $NHCH_{Leu}$), 6.90 (dd, ^3J = 8.8 Hz, ^4J = 2.9 Hz, 1H, 6-H), 7.44 (d, ^3J = 8.8 Hz, 1H, 5-H), 7.54 (d, ^4J = 1.8 Hz, 1H, 2-H), 8.10 (d, ^3J = 8.0 Hz, 1H, OCONH), 9.48 (s, 1H, NHPh); ^{13}C NMR (150 MHz, DMSO-d_6) δ 21.42, 23.24, 24.24, 24.34 ($CH(CH_3)_{2,Leu}$, CH_3CO), 24.74 (C-2*), 27.71 ($C(CH_3)_3$), 28.65 (C-3*), 40.90 ($CHCH_{2,Leu}$), 46.50 (C-1*), 51.17 ($NHCH_{Leu}$), 59.59 (C-4*), 80.42 ($C(CH_3)_3$), 118.52 (C-2), 119.24 (C-6), 129.71 (C-5), 135.76 (C-3), 149.72 (C-1), 154.07 (OCONH), 168.94 (CH_3CO), 170.34 (CHCO), 171.00 (COO), one signal (C-4) is not visible; LC/MS (ESI): H_2O/MeCN, 90:10 to 0:100; *m/z* 496.1 $[M + H]^+$; 99% purity; Q-TOF: HRMS (ESI): *m/z* $[M + H]^+$ calcd. for $C_{24}H_{34}ClN_3O_6$: 496.2209, found: 496.2173.

3-Acetamido-4-chlorophenoxycarbonyl-leucyl-proline

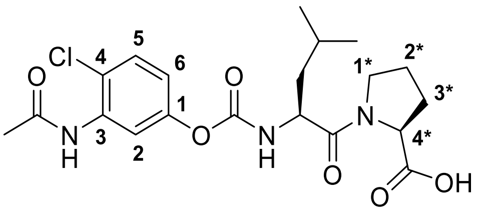

The synthesis was carried out according to GP8 using the corresponding *tert*-butyl ester (149 mg, 0.30 mmol). Column chromatography on silica gel afforded the desired compound as a colorless resin (109 mg, 0.25 mmol, 83%). ^{1}H NMR (500 MHz, DMSO-d_6) δ 0.92 (2 × d, ^3J = 6.7 Hz, 6H, $CH(CH_3)_{2,Leu}$), 1.40–1.46 (m, 1H, $CHCH_{2,Leu}$), 1.51–1.57 (m, 1H, $CHCH_{2,Leu}$), 1.71–1.76 (m, 1H, $CH(CH_3)_{2,Leu}$), 1.80–1.85 (m, 1H, 2*-H), 1.89–1.95 (m, 2H, 2*-H, 3*-H), 2.09 (s, 3H, CH_3CO), 2.13–2.18 (m, 1H, 3*-H), 3.50 (dt, ^2J = 9.7 Hz, ^3J = 6.6 Hz, 1H, 1*-H), 3.67 (dt, ^2J = 9.5 Hz, ^3J = 6.9 Hz, 1H, 1*-H), 4.27 (dd, ^3J = 4.6 Hz, ^3J = 8.6 Hz, 1H, 4*-H), 4.29–4.33 (m, 1H, $NHCH_{Leu}$), 6.90 (dd, ^3J = 8.8 Hz, ^4J = 2.8 Hz, 1H, 6-H), 7.44 (d, ^3J = 8.8 Hz, 1H, 5-H), 7.55 (d, ^4J = 2.8 Hz, 1H, 2-H), 8.08 (d, ^3J = 7.9 Hz, 1H, OCONH), 9.47 (s, 1H, NHPh), one signal (COOH) is not visible; ^{13}C NMR (125 MHz, DMSO-d_6) δ 21.52, 23.22, 23.60, 24.25 ($CH(CH_3)_{2,Leu}$, CH_3CO), 24.73 (C-2*), 28.64 (C-3*), 46.50 (C-1*), 51.11 ($NHCH_{Leu}$), 58.67 (C-4*), 118.53 (C-2), 119.24 (C-6), 121.67 (C-4), 129.70 (C-5), 135.75 (C-3), 149.72 (C-1), 154.03 (OCONH), 168.94 (CH_3CO), 170.34 (CHCO), 173.28 (COOH), one signal ($CHCH_{2,Leu}$) is obscured by the DMSO solvent signal; LC/MS (ESI): H_2O/MeCN, 90:10 to 0:100; *m/z* 440.2 $[M + H]^+$; 95% purity; Q-TOF: HRMS (ESI): *m/z* $[M - H]^-$ calcd. for $C_{20}H_{26}ClN_3O_6$: 438.1437, found: 438.1447.

4-Chloro-3-phenylacetamidophenoxycarbonyl-leucyl-proline *tert*-butyl ester

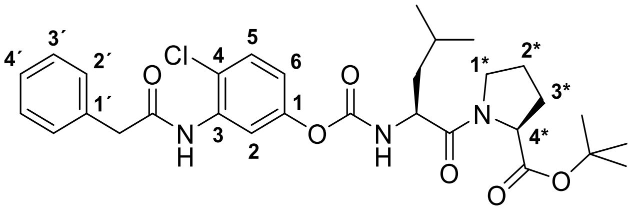

The synthesis was carried out according to GP13 using Cbz-Leu-Pro-O*t*Bu (0.31 g) for the hydrogenation and phenol **43** (131 mg) for the subsequent carbamate formation. Column chromatography on silica gel using petrolum ether / EtOAc (2:1) as eluent afforded the product as a colorless resin (155 mg, 0.27 mmol, 54%). ^{1}H NMR (600 MHz, $CDCl_3$) δ 0.98 (2 × d, ^3J = 6.6 Hz, 6H, CH(CH_3)$_{2,Leu}$), 1.44 (s, 9H, C(CH_3)$_3$), 1.49–1.55 (m, 1H, CH$CH_{2,Leu}$), 1.56–1.63 (m, 1H, CH$CH_{2,Leu}$), 1.78–1.84 (m, 1H, CH(CH_3)$_{2,Leu}$), 1.90–2.06 (m, 3H, 2*-H, 3*H), 2.16–2.22 (m, 1H, 3*-H), 3.53–3.59 (m, 1H, 1*-H), 3.68–3.73 (m, 1H, 1*-H), 3.76 (m, 2H, PhCH_2), 4.38–4.43 (m, 1H, NHCH_{Leu}), 4.51–4.56 (t, ^3J = 8.0 Hz, 1H, 4*-H), 5.72 (d, ^3J = 8.9 Hz, 1H, 1H, OCONH), 6.77 (dd, ^3J = 8.7 Hz, ^4J = 2.7 Hz, 1H, 6*-H), 7.20 (d, ^3J = 8.6 Hz, 1H, 5-H), 7.31–7.35 (m, 3H, 2´-H, 4´-H), 7.40 (t, ^3J = 7.5 Hz, 2H, 3´-H), 7.62 (s, 1H, NHPh), 8.23 (d, ^4J = 2.7 Hz, 1H, 2-H); ^{13}C NMR (150 MHz, $CDCl_3$) δ 21.63, 23.49, 24.58 (CH(CH_3)$_{2,Leu}$), 24.84 (C-2*), 27.99 (C(CH_3)$_3$), 29.07 (C-3*), 42.15 (CH$CH_{2,Leu}$), 45.16 (PhCH_2), 46.84 (C-1*), 51.07 (NHCH_{Leu}), 59.67 (C-4*), 81.44 (C(CH_3)$_3$), 114.60 (C-2), 118.03 (C-6), 118.86 (C-4), 127.95 (C-4´), 128.95 (C-5), 129.39, 129.72 (C-2´, C-3´), 133.76 (C-1´), 134.91 (C-3), 150.01 (C-1), 154.20 (OCONH), 169.00 (CH_2CO), 170.81, 171.01 (CHCO); LC/MS (ESI): H_2O/MeCN, 90:10 to 0:100; *m/z* 572.4 [M + H]$^+$; 100% purity; Q-TOF: HRMS (ESI): *m/z* [M + H]$^+$ calcd. for $C_{30}H_{38}ClN_3O_6$: 572.2522, found: 572.2460.

4-Chloro-3-phenylacetamidophenoxycarbonyl-leucyl-proline (20)

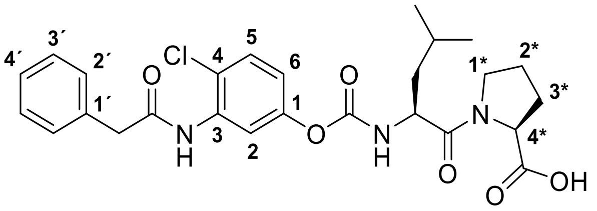

The synthesis was carried out according to GP8 using the corresponding *tert*-butyl ester (58 mg, 0.10 mmol). Column chromatography on silica gel afforded the desired compound as a colorless solid (48 mg, 0.09 mmol, 90%). mp 88–96 °C; ^{1}H NMR (600 MHz, $CDCl_3$) δ 0.98 (2 × d, ^3J = 6.6 Hz, 6H, CH(CH_3)$_{2,Leu}$), 1.48–1.55 (m, 1H, CH$CH_{2,Leu}$), 1.60–1.67 (m, 1H, CH$CH_{2,Leu}$), 1.76–1.83 (m, 1H, CH(CH_3)$_{2,Leu}$), 2.02–2.09 (m, 2H, 2*-H), 2.12–2.18 (m, 1H, 3*-H), 2.23–2.30 (m, 1H, 3*-H), 3.56–3.62 (m, 1H, 1*-H), 3.75–3.81 (m, 3H, 1*-H, PhCH_2), 4.56 (dt, ^3J = 4.1 Hz, ^3J = 9.5 Hz, 1H, NHCH_{Leu}), 4.63 (dd, ^3J = 3.8 Hz, ^3J = 8.3 Hz, 1H, 4*H), 5.88 (d, ^3J = 8.8 Hz, 1H, OCONH), 6.79 (dd, ^3J = 8.7 Hz, ^4J = 2.8 Hz, 1H, 6-H), 7.22 (d, ^3J = 8.7 Hz, 1H, 5-H), 7.32–7.36 (m, 3H, 2´-H, 4´-H), 7.41 (t, ^3J = 7.5 Hz, 2H, 3´-H), 7.66 (s, 1H, NHPh), 8.25 (d, ^4J = 2.7 Hz, 1H, 2-H), one signal (COOH) is not visible; ^{13}C NMR (150 MHz, $CDCl_3$) δ 21.72, 23.30, 24.52 (CH(CH_3)$_{2,Leu}$), 24.87 (C-2*), 27.89 (C-3*), 41.64 (CH$CH_{2,Leu}$), 45.10 (PhCH_2), 47.33 (C-1*), 51.04 (NHCH_{Leu}), 59.44 (C-4*), 114.55 (C-2), 117.98 (C-6), 119.00 (C-4), 127.96 (C-4´), 128.99 (C-5), 129.38, 129.69 (C-2´, C-3´), 133.66 (C-1´), 134.89 (C-3), 149.86 (C-1), 154.21 (OCONH), 169.16 (CH_2CO), 173.05 (CHCO), 173.55 (COOH); LC/MS (ESI): H_2O/MeCN, 90:10 to 0:100; *m/z* 516.3 [M + H]$^+$; 99% purity; Q-TOF: HRMS (ESI): *m/z* [M - H]$^-$ calcd. for $C_{26}H_{30}ClN_3O_6$: 514.1750, found: 514.1765.

4-Chloro-3-(4-phenylbenzamido)phenoxycarbonyl-leucyl-proline *tert*-butyl ester

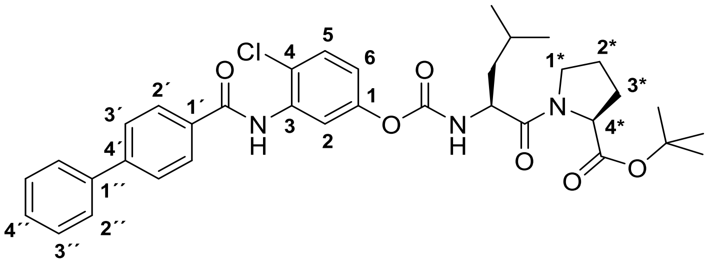

The synthesis was carried out according to GP13 using Cbz-Leu-Pro-O*t*Bu (0.31 g) for the hydrogenation and phenol **45** (162 mg) for the subsequent carbamate formation. Column chromatography on silica gel using petrolum ether / EtOAc (2:1) as eluent afforded the product as a colorless resin (127 mg, 0.20 mmol, 40%). ^{1}H NMR (600 MHz, CDCl$_3$) δ 1.00 (2 × d, ^{3}J = 6.6 Hz, 6H, CH(C$\underline{H}_3$)$_{2,\mathrm{Leu}}$), 1.45 (s, 9H, C(C$\underline{H}_3$)$_3$), 1.52–1.57 (m, 1H, CHC$\underline{H}_{2,\mathrm{Leu}}$), 1.60–1.65 (m, 1H, CHC$\underline{H}_{2,\mathrm{Leu}}$), 1.81–1.87 (m, 1H, C$\underline{H}$(CH$_3$)$_{2,\mathrm{Leu}}$), 1.92–2.06 (m, 3H, 2*-$\underline{H}$, 3*-$\underline{H}$), 2.17–2.22 (m, 1H, 3*-$\underline{H}$), 3.55–3.60 (m, 1H, 1*-$\underline{H}$), 3.73 (dt, ^{2}J = 9.5 Hz, ^{3}J = 6.9 Hz, 1H, 1*-$\underline{H}$), 4.43 (dd, ^{3}J = 4.6 Hz, ^{3}J = 8.5 Hz, 1H, 4*-$\underline{H}$), 4.57 (dt, ^{3}J = 3.6 Hz, ^{3}J = 10.1 Hz, 1H, NHC$\underline{H}_{\mathrm{Leu}}$), 5.81 (d, ^{3}J = 8.9 Hz, 1H, OCON$\underline{H}$), 6.87 (dd, ^{3}J = 8.7 Hz, ^{4}J = 2.8 Hz, 1H, 6-$\underline{H}$), 7.35 (d, ^{3}J = 8.7 Hz, 1H, 5-$\underline{H}$), 7.39 (t, ^{3}J = 7.4 Hz, 1H, 4´´-$\underline{H}$), 7.46 (t, ^{3}J = 7.7 Hz, 2H, 3´´-$\underline{H}$), 7.62 (d, ^{3}J = 7.3 Hz, 2H, 2´´-$\underline{H}$), 7.71 (d, ^{3}J = 8.2 Hz, 2H, 2´-$\underline{H}$), 7.95 (d, ^{3}J = 8.3 Hz, 2H, 3´-$\underline{H}$), 8.46 (m, 2H, 2-$\underline{H}$, N$\underline{H}$Ph); ^{13}C NMR (150 MHz, CDCl$_3$) δ 21.62, 23.46, 24.56 ($\underline{C}$H($\underline{C}$H$_3$)$_{2,\mathrm{Leu}}$), 24.81 ($\underline{C}$-2*), 27.95 ($\underline{C}$-3*), 29.05 (C($\underline{C}$H$_3$)$_3$), 42.11 (CH$\underline{C}$H$_{2,\mathrm{Leu}}$), 46.82 ($\underline{C}$-1*), 51.06 (NH$\underline{C}$H$_{\mathrm{Leu}}$), 59.66 ($\underline{C}$-4*), 81.41 ($\underline{C}$(CH$_3$)$_3$), 114.95 ($\underline{C}$-2), 118.10 ($\underline{C}$-6), 119.15 ($\underline{C}$-4), 127.23, 127.60, 127.62 ($\underline{C}$-3´, $\underline{C}$-2´´, $\underline{C}$-3´´), 128.19 ($\underline{C}$-4´´), 128.97 ($\underline{C}$-2´), 129.06 ($\underline{C}$-5), 133.00 ($\underline{C}$-1´), 135.22 ($\underline{C}$-3), 139.76 ($\underline{C}$-1´´), 145.13 ($\underline{C}$-4´), 150.18 ($\underline{C}$-1), 154.22 (O$\underline{C}$ONH), 164.83 (Ph$\underline{C}$O), 170.83, 170.97 (CH$\underline{C}$O); LC/MS (ESI): H_2O/MeCN, 90:10 to 0:100; *m/z* 634.4 [M + H]$^+$; 97% purity; Q-TOF: HRMS (ESI): *m/z* [M + H]$^+$ calcd. for $C_{35}H_{40}ClN_3O_6$: 634.2678, found: 634.2680.

4-Chloro-3-(4-phenylbenzamido)phenoxycarbonyl-leucyl-proline

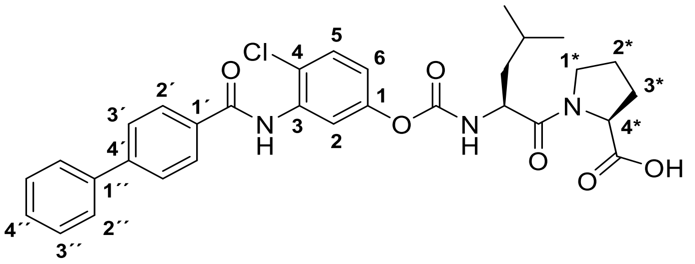

The synthesis was carried out according to GP8 using the corresponding *tert*-butyl ester (127 mg, 0.20 mmol). Column chromatography on silica gel afforded the desired compound as a colorless solid (106 mg, 0.18 mmol, 92%). mp 99–102 °C: ^{1}H NMR (600 MHz, CDCl$_3$) δ 0.99 (2 × d, ^{3}J = 6.5 Hz, 6H, CH(C$\underline{H}_3$)$_{2,\mathrm{Leu}}$), 1.51–1.56 (m, 1H, CHC$\underline{H}_{2,\mathrm{Leu}}$), 1.63–1.69 (m, 1H, CHC$\underline{H}_{2,\mathrm{Leu}}$), 1.78–1.86 (m, 1H, C$\underline{H}$(CH$_3$)$_{2,\mathrm{Leu}}$), 2.02–2.10 (m, 2H, 2*-$\underline{H}$), 2.12–2.19 (m, 1H, 3*-$\underline{H}$), 2.24–2.29 (m, 1H, 3*-$\underline{H}$), 3.58–3.63 (m, 1H, 1*-$\underline{H}$), 3.79 (dt, ^{2}J = 9.9 Hz, ^{3}J = 7.5 Hz, 1H, 1*-$\underline{H}$), 4.59 (dt, ^{3}J = 4.1 Hz, ^{3}J = 9.6 Hz, 1H,

NHCH$_{Leu}$), 4.64 (dd, ^3J = 3.9 Hz, ^3J = 8.4 Hz, 1H, 4*-H), 5.99 (d, ^3J = 8.7 Hz, 1H, OCONH), 6.88 (dd, ^3J = 8.8 Hz, ^4J = 2.7 Hz, 1H, 6-H), 7.37 (d, ^3J = 8.7 Hz, 1H, 5-H), 7.39 (t, ^3J = 7.4 Hz, 1H, 4´´-H), 7.47 (t, ^3J = 7.6 Hz, 2H, 3´´-H), 7.62 (d, ^3J = 7.3 Hz, 2H, 2´´-H), 7.72 (d, ^3J = 8.3 Hz, 2H, 2´-H), 7.95 (d, ^3J = 8.3 Hz, 2H, 3´-H), 8.45 (d, ^4J = 2.7 Hz, 1H, 2-H), 8.48 (s, 1H, NHPh), one signal (COOH) is not visible; ^{13}C NMR (150 MHz, CDCl$_3$) δ 21.71, 23.31, 24.54 (CH(CH$_3$)$_{2,Leu}$), 24.87 (C-2*), 27.94 (C-3*), 41.58 (CHCH$_{2,Leu}$), 47.33 (C-1*), 51.09 (NHCH$_{Leu}$), 59.45 (C-4*), 114.93 (C-2), 118.08 (C-6), 119.32 (C-4), 127.22, 127.61, 127.64 (C-3´, C-2´´, C-3´´), 128.21 (C-4´´), 128.98 (C-2´), 129.12 (C-5), 132.90 (C-1´), 135.23 (C-3), 139.72 (C-1´´), 145.18 (C-4´), 150.05 (C-1), 154.26 (OCONH), 164.92 (PhCO), 173.02, 173.25 (CHCO); LC/MS (ESI): H_2O/MeCN, 90:10 to 0:100; *m/z* 558.3 [M + H]$^+$; 97% purity; Q-TOF: HRMS (ESI): *m/z* [M + H]$^+$ calcd. for $C_{31}H_{32}ClN_3O_6$: 578.2052, found: 578.2054.

4-Bromo-3-phenylacetamidophenoxycarbonyl-leucyl-proline

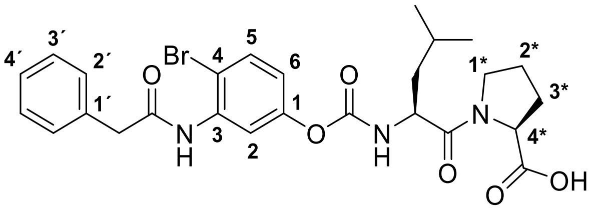

The synthesis was carried out according to GP13 using Cbz-Leu-Pro-O*t*Bu (0.31 g) for the hydrogenation and phenol **48** (169 mg) for the subsequent carbamate formation. Column chromatography on silica gel using petrolum ether / EtOAc (3:1) as eluent resulted in a partially purified product, which was subjected to a *tert*-butyl ester cleavage according to GP8. Column chromatography on silica gel afforded the desired compound as a colorless solid (44 mg, 0.08 mmol, 79%). mp 85–91 °C; ^{1}H NMR (600 MHz, CDCl$_3$) δ 0.97 (2 × d, ^3J = 6.5 Hz, 6H, CH(CH$_3$)$_{2,Leu}$), 1.48–1.53 (m, 1H, CHCH$_{2,Leu}$), 1.60–1.66 (m, 1H, CHCH$_{2,Leu}$), 1.75–1.81 (m, 1H, CH(CH$_3$)$_{2,Leu}$), 2.00–2.15 (m, 3H, 2*-H, 3*-H), 2.23–2.29 (m, 1H, 3*-H), 3.55–3.60 (m, 1H, 1*-H), 3.73–3.79 (m, 3H, 1*-H, PhCH$_2$), 4.55 (dt, ^3J = 4.0 Hz, ^3J = 9.5 Hz, 1H, NHCH$_{Leu}$), 4.60 (dd, ^3J = 4.0 Hz, ^3J = 8.1 Hz, 1H, 4*H), 5.91 (d, ^3J = 8.8 Hz, 1H, OCONH), 6.73 (dd, ^3J = 8.6 Hz, ^4J = 2.8 Hz, 1H, 6-H), 7.31–7.35 (m, 3H, 2´-H, 4´-H), 7.37 (d, ^3J = 8.8 Hz, 1H, 5-H), 7.40 (t, ^3J = 7.6 Hz, 2H, 3´-H), 7.64 (s, 1H, NHPh), 8.22 (d, ^4J = 2.8 Hz, 1H, 2-H), one signal (COOH) is not visible; ^{13}C NMR (150 MHz, CDCl$_3$) δ 21.70, 23.32, 24.52 (CH(CH$_3$)$_{2,Leu}$), 24.88 (C-2*), 27.86 (C-3*), 41.61 (CHCH$_{2,Leu}$), 45.14 (PhCH$_2$), 47.34 (C-1*), 51.06 (NHCH$_{Leu}$), 59.56 (C-4*), 108.90 (C-2), 114.74 (C-6), 118.53 (C-4), 127.98 (C-4´), 129.41, 129.83 (C-2´, C-3´), 132.16 (C-5), 133.64 (C-1´), 136.07 (C-3), 150.53 (C-1), 154.12 (OCONH), 169.20 (CH$_2$CO), 173.02 (CHCO), 173.16 (COOH); LC/MS (ESI): H_2O/MeCN, 90:10 to 0:100; *m/z* 560.1 [M + H]$^+$; 99% purity; Q-TOF: HRMS (ESI): *m/z* [M + H]$^+$ calcd. for $C_{26}H_{30}BrN_3O_6$: 560.1391, found: 560.1402.

4-Phenyl-3-phenylacetamidophenoxycarbonyl-leucyl-proline *tert*-butyl ester

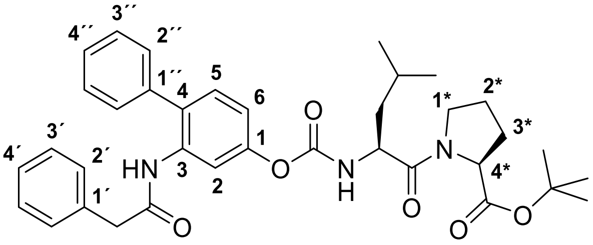

The synthesis was carried out according to GP13 using Cbz-Leu-Pro-O*t*Bu (0.31 g) for the hydrogenation and phenol **49** (151 mg) for the subsequent carbamate formation. Column chromatography on silica gel using petrolum ether / EtOAc (1:1) as eluent afforded the product as a colorless resin (102 mg, 0.17 mmol, 33%). ^{1}H NMR (600 MHz, $CDCl_3$) δ 0.99 (2 × d, ^3J = 6.6 Hz, 6H, CH(CH$_3$)$_{2,Leu}$), 1.45 (s, 9H, C(CH$_3$)$_3$), 1.53–1.57 (m, 1H, CHCH$_{2,Leu}$), 1.59–1.65 (m, 1H, CHCH$_{2,Leu}$), 1.81–1.87 (m, 1H, CH(CH$_3$)$_{2,Leu}$), 1.92–2.05 (m, 3H, 2*-H, 3*-H), 2.17–2.22 (m, 1H, 3*-H), 3.55–3.59 (m, 3H, 1*-H, PhCH$_2$), 3.74 (dt, ^2J = 9.7 Hz, ^3J = 6.9 Hz, 1H, 1*-H), 4.42 (dd, ^3J = 4.6 Hz, ^3J = 8.4 Hz, 1H, 4*-H), 4.57 (dt, ^3J = 3.6 Hz, ^3J = 10.2 Hz, 1H, NHCH$_{Leu}$), 5.77 (d, ^3J = 9.0 Hz, 1H, OCONH), 6.87 (dd, ^3J = 8.3 Hz, ^4J = 2.4 Hz, 1H, 6-H), 6.96–7.02 (m, 4H, 2´´-H, 3´´-H), 7.06 (d, ^3J = 8.3 Hz, 1H, 5-H), 7.15–7.19 (m, 4H, 2´-H, 4´-H, NHPh), 7.20–7.24 (m, 2H, 3´-H), 7.25–7.29 (m, 1H, 4´´-H), 8.24 (d, ^4J = 2.3 Hz, 1H, 2-H); ^{13}C NMR (150 MHz, $CDCl_3$) δ 21.64, 23.48, 24.54 (CH(CH$_3$)$_{2,Leu}$), 24.81 (C-2*), 27.96 (C(CH$_3$)$_3$), 29.06 (C-3*), 42.17 (CHCH$_{2,Leu}$), 45.14 (PhCH$_2$), 46.82 (C-1*), 51.00 (NHCH$_{Leu}$), 59.65 (C-4*), 81.39 (C(CH$_3$)$_3$), 113.83 (C-2), 117.33 (C-6), 127.53, 127.63 (C-4´, C-4´´), 128.78 (C-4), 128.84, 129.05 (C-2´´, C-3´´), 129.17, 129.30 (C-2´, C-3´), 130.34 (C-5), 133.58 (C-1´), 135.45 (C-1´´), 136.96 (C-3), 150.63 (C-1), 154.62 (OCONH), 168.80 (CH$_2$CO), 170.93, 171.02 (CHCO); LC/MS (ESI): H_2O/MeCN, 90:10 to 0:100; *m/z* 614.4 [M + H]$^+$; 91% purity; Q-TOF: HRMS (ESI): *m/z* [M + H]$^+$ calcd. for $C_{36}H_{43}N_3O_6$: 614.3225, found: 614.3253.

4-Phenyl-3-phenylacetamidophenoxycarbonyl-leucyl-proline (21)

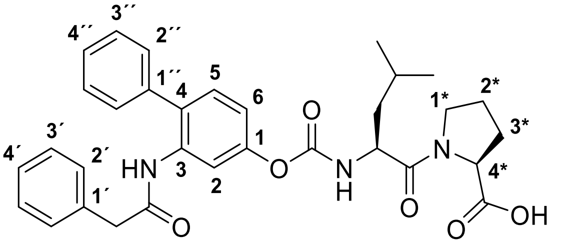

The synthesis was carried out according to GP8 using the corresponding *tert*-butyl ester (62 mg, 0.10 mmol). Column chromatography on silica gel afforded the desired compound as a colorless solid (43 mg, 0.08 mmol, 77%). mp 108–112 °C; ^{1}H NMR (600 MHz, $CDCl_3$) δ 0.99 (2 × d, ^3J = 6.5 Hz, 6H, CH(CH$_3$)$_{2,Leu}$), 1.51–1.55 (m, 1H, CHCH$_{2,Leu}$), 1.62–1.67 (m, 1H, CHCH$_{2,Leu}$), 1.78–1.85 (m, 1H, CH(CH$_3$)$_{2,Leu}$), 2.02–2.10 (m, 2H, 2*-H), 2.16–2.23 (m, 2H, 3*-H), 3.53–3.64 (m, 3H, 1*-H, PhCH$_2$), 3.81 (dt, ^2J = 9.8 Hz, ^3J = 7.4 Hz, 1H, 1*-H), 4.57–4.63 (m, 2H, 4*-H, NHCH$_{Leu}$), 6.02 (d, ^3J = 8.8 Hz, 1H, OCONH), 6.88 (dd, ^3J = 8.3 Hz, ^4J = 2.4 Hz, 1H, 6-H), 6.96–7.02 (m, 4H, 2´´-H, 3´´-H), 7.07 (d, ^3J

= 8.3 Hz, 1H, 5-H), 7.14–7.23 (m, 6H, 2´-H, 3´-H, 4´-H, NHPh), 7.28 (t, ^3J = 7.4 Hz, 1H, 4´´-H), 8.23 (d, ^4J = 2.4 Hz, 1H, 2-H), one signal (COOH) is not visible; ^{13}C NMR (150 MHz, $CDCl_3$) δ 21.75, 23.29, 24.50 ($CH(CH_3)_{2,Leu}$), 24.86 (C-2*), 28.18 (C-3*), 41.56 ($CHCH_{2,Leu}$), 45.05 ($PhCH_2$), 47.27 (C-1*), 51.04 ($NHCH_{Leu}$), 59.32 (C-4*), 113.86 (C-2), 117.37 (C-6), 127.57, 127.68 (C-4´, C-4´´), 128.81, 129.07 (C-2´´, C-3´´), 128.97 (C-4), 129.19, 129.29 (C-2´, C-3´), 130.39 (C-5), 133.45 (C-1´), 135.37 (C-1´´), 136.84 (C-3), 150.51 (C-1), 154.70 (OCONH), 169.10 (CH_2CO), 172.91 (CHCO), 174.64 (COOH); LC/MS (ESI): H_2O/MeCN, 90:10 to 0:100; *m/z* 558.1 $[M + H]^+$; 97% purity; Q-TOF: HRMS (ESI): *m/z* $[M - H]^-$ calcd. for $C_{32}H_{35}N_3O_6$: 556.2453, found: 556.2432.

4-Chloro-3-(2-diphenylacetamido)phenoxycarbonyl-leucyl-proline *tert*-butyl ester

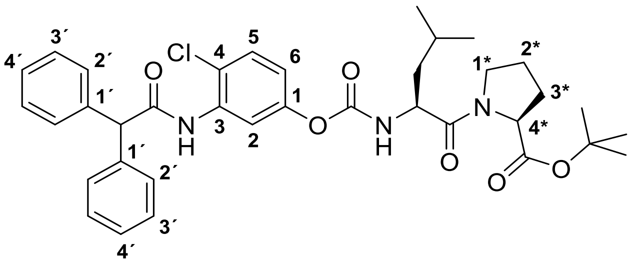

The synthesis was carried out according to GP13 using Cbz-Leu-Pro-O*t*Bu (0.31 g) for the hydrogenation and phenol **47** (169 mg) for the subsequent carbamate formation. Column chromatography on silica gel using petrolum ether / EtOAc (3:1) as eluent afforded the product as a colorless solid (210 mg, 0.32 mmol, 65%). mp 94–97 °C; ^{1}H NMR (600 MHz, $CDCl_3$) δ 0.97 (2 × d, ^3J = 6.6 Hz, 6H, $CH(CH_3)_{2,Leu}$), 1.43 (s, 9H, $C(CH_3)_3$), 1.50–1.55 (m, 1H $CHCH_{2,Leu}$), 1.56–1.62 (m, 1H, $CHCH_{2,Leu}$), 1.77–1.83 (m, 1H, $CH(CH_3)_{2,Leu}$), 1.91–2.04 (m, 3H, 2*-H, 3*-H), 2.15–2.21 (m, 1H, 3*-H), 3.53–3.58 (m, 1H, 1*-H), 3.68–3.73 (m, 1H, 1*-H), 4.40 (dd, ^3J = 4.5 Hz, ^3J = 8.5 Hz, 1H, 4*-H), 4.53 (dt, ^3J = 3.6 Hz, ^3J = 10.2 Hz, 1H, $NHCH_{Leu}$), 5.12 (s, 1H, Ph_2CH), 5.75 (d, ^3J = 8.9 Hz, 1H, OCONH), 6.79 (dd, ^3J = 8.8 Hz, ^4J = 2.7 Hz, 1H, 6-H), 7.22 (d, ^3J = 8.7 Hz, 1H, 5-H), 7.28– 7.32 (m, 6H, 2´-H, 4´-H), 7.33–7.37 (m, 4H, 3´-H), 7.85 (s, 1H, NHPh), 8.32 (d, ^4J = 2.7 Hz, 1H, 2-H); ^{13}C NMR (150 MHz, $CDCl_3$) δ 21.59, 23.43, 23.80 ($CH(CH_3)_{2,Leu}$), 24.78 (C-2*), 27.94 ($C(CH_3)_3$), 29.02 (C-3*), 42.07 ($CHCH_{2,Leu}$), 46.80 (C-1*), 51.02 ($NHCH_{Leu}$), 59.64 (PH_2CH), 60.49 (C-4*), 81.40 ($C(CH_3)_3$), 114.69 (C-2), 118.19 (C-6), 118.95 (C-4), 127.66 (C-4´), 128.93 (C-5), 129.01 (C-2´, C-3´), 134.86 (C-3), 138.54 (C-1´), 150.00 (C-1), 154.18 (OCONH), 170.10, 170.81, 170.5 (CHCO); LC/MS (ESI): H_2O/MeCN, 90:10 to 0:100; *m/z* 648.5 $[M + H]^+$; 94% purity; Q-TOF: HRMS (ESI): *m/z* $[M + H]^+$ calcd. for $C_{36}H_{42}ClN_3O_6$: 648.2835, found: 648.2847.

4-Chloro-3-(2-diphenylacetamido)phenoxycarbonyl-leucyl-proline (22)

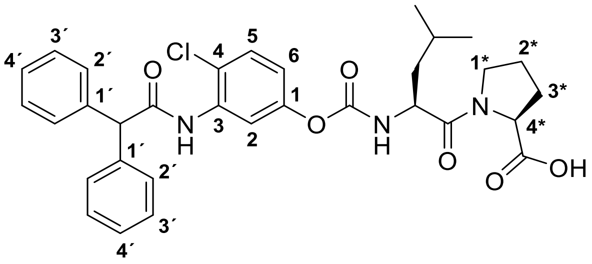

The synthesis was carried out according to GP8 using the corresponding *tert*-butyl ester (195 mg, 0.30 mmol). Column chromatography on silica gel afforded the desired compound as a colorless solid (135 mg, 0.23 mmol, 76%). mp 102–106 °C; ^{1}H NMR (600 MHz, $CDCl_3$) δ 0.96 (2 × d, ^{3}J = 6.5 Hz, 6H, CH(C<u>H</u>$_3$)$_{2,Leu}$), 1.47–1.53 (m, 1H, CHC<u>H</u>$_{2,Leu}$), 1.58–1.64 (m, 1H, CHC<u>H</u>$_{2,Leu}$), 1.74–1.82 (m, 1H, C<u>H</u>(CH$_3$)$_{2,Leu}$), 1.98–2.07 (m, 2H, 2*-<u>H</u>), 2.12–2.18 (m, 1H, 3*-<u>H</u>), 2.19–2.25 (m, 1H, 3*-<u>H</u>), 3.54–3.62 (m, 1H, 1*-<u>H</u>), 3.73–3.82 (m, 1H, 1*-<u>H</u>), 4.52–4.58 (m, 1H, NHC<u>H</u>$_{Leu}$), 4.59–4.63 (m, 1H, 4*-<u>H</u>), 5.13 (s, 1H, Ph$_2$C<u>H</u>), 5.92 (d, ^{3}J = 8.7 Hz, 1H, OCON<u>H</u>), 6.80 (dd, ^{4}J = 2.4 Hz, ^{3}J = 8.7 Hz, 1H, 6-<u>H</u>), 7.23 (d, ^{3}J = 8.7 Hz, 1H, 5-<u>H</u>), 7.28-7.32 (m, 6H, 2´-<u>H</u>, 4´-<u>H</u>), 7.35 (t, ^{3}J = 7.5 Hz, 4H, 3´-<u>H</u>), 7.87 (s, 1H, N<u>H</u>Ph), 8.33 (d, ^{4}J = 2.1 Hz, 1H, 2-<u>H</u>), one signal (COO<u>H</u>) is not visible; ^{13}C NMR (150 MHz, $CDCl_3$) δ 21.73, 23.31, 24.51 (<u>C</u>H(<u>C</u>H$_3$)$_{2,Leu}$), 24.88 (<u>C</u>-2*), 28.06 (<u>C</u>-3*), 41.63 (CH<u>C</u>H$_{2,Leu}$), 47.28 (<u>C</u>-1*), 51.06 (NH<u>C</u>H$_{Leu}$), 59.35 (PH$_2$<u>C</u>H), 60.50 (<u>C</u>-4*), 114.69 (<u>C</u>-2), 118.18 (<u>C</u>-6), 119.10 (<u>C</u>-4), 127.71 (<u>C</u>-4´), 129.03, 129.05 (<u>C</u>-2´, <u>C</u>-3´), 129.11 (<u>C</u>-5), 134.90 (<u>C</u>-3), 138.51 (<u>C</u>-1´), 149.92 (<u>C</u>-1), 154.24 (O<u>C</u>ONH), 170.25, 174.29, 176.15 (CH<u>C</u>O); LC/MS (ESI): H_2O/MeCN, 90:10 to 0:100; *m/z* 592.2 [M + H]$^+$; 99% purity; Q-TOF: HRMS (ESI): *m/z* [M + H]$^+$ calcd. for $C_{32}H_{34}ClN_3O_6$: 592.2209, found: 592.2175.

4-Fluorophenoxycarbonyl-leucyl-proline *tert*-butyl ester

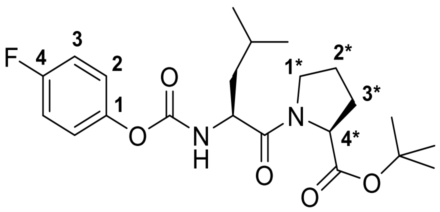

The synthesis was carried out according to GP13 using Cbz-Leu-Pro-O*t*Bu (0.31 g) for the hydrogenation and 4-fluorophenol (56 mg) for the subsequent carbamate formation. Column chromatography on silica gel using petrolum ether / EtOAc (3:1) as eluent afforded the product as a colorless resin (121 mg, 0.29 mmol, 57%). ^{1}H NMR (600 MHz, DMSO-d_6) δ 0.92 (d, ^{3}J = 6.6 Hz, 6H, CH(C<u>H</u>$_3$)$_{2,Leu}$), 1.37 (s, 9H, C(C<u>H</u>$_3$)$_3$), 1.40–1.44 (m, 1H, CHC<u>H</u>$_{2,Leu}$), 1.54–1.59 (m, 1H, CHC<u>H</u>$_{2,Leu}$), 1.72–1.80 (m, 2H, C<u>H</u>(CH$_3$)$_{2,Leu}$, 2*-<u>H</u>), 1.89–1.95 (m, 2H, 2*-<u>H</u>, 3*-<u>H</u>), 2.12–2.18 (m, 1H, 3*-<u>H</u>), 3.48 (dt, ^{2}J = 9.7 Hz, ^{3}J = 6.8 Hz, 1H, 1*-<u>H</u>), 3.66 (dt, ^{2}J = 9.6 Hz, ^{3}J = 6.9 Hz, 1H, 1*-<u>H</u>), 4.19 (dd, ^{3}J = 5.0 Hz, ^{3}J = 8.6 Hz, 1H, 4*-<u>H</u>), 4.28–4.23 (m, 1H, NHC<u>H</u>$_{Leu}$), 7.08–7.10 (m, 2H, 3-<u>H</u>), 7.16–7.19 (m, 2H, 2-<u>H</u>), 8.04 (d, ^{3}J = 8.0 Hz, 1H, OCON<u>H</u>); ^{13}C NMR (150 MHz, DMSO-d_6) δ 21.41, 23.21, 24.30

(CH(CH$_3$)$_{2,Leu}$), 24.71 (C-2*), 27.67 (C(CH$_3$)$_3$), 28.63 (C-3*), 46.46 (C-1*), 51.13 (NHCH$_{Leu}$), 59.57 (C-4*), 80.39 (C(CH$_3$)$_3$), 115.87 (d, ^2J = 23.2 Hz, C-3), 123.48 (d, ^3J = 8.3 Hz, C-2), 147.21 (d, ^4J = 2.1 Hz, C-1), 154.46 (OCONH), 159.19 (d, ^1J = 240.2 Hz, C-4), 170.37, 170.98 (CHCO), one signal (CHCH$_{2,Leu}$) is obscured by the DMSO solvent signal; LC/MS (ESI): H_2O/MeCN, 90:10 to 0:100; *m/z* 423.4 [M + H]$^+$; 98% purity; Q-TOF: HRMS (ESI): *m/z* [M + H]$^+$ calcd. for $C_{22}H_{31}FN_2O_5$: 423.2290, found: 423.2246.

4-Fluorophenoxycarbonyl-leucyl-proline

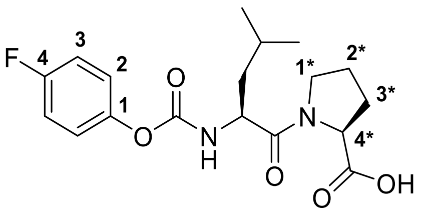

The synthesis was carried out according to GP8 using the corresponding *tert*-butyl ester (106 mg, 0.25 mmol). Column chromatography on silica gel was not required to obtain the product as a colorless resin (92 mg, 0.15 mmol, 100%). ^{1}H NMR (600 MHz, DMSO-d_6) δ 0.92 (2 × d, ^3J = 6.6 Hz, 6H, CH(CH_3)$_{2,Leu}$), 1.41–1.45 (m, 1H, CHC$H_{2,Leu}$), 1.52–1.57 (m, 1H, CHC$H_{2,Leu}$), 1.70–1.76 (m, 1H, CH(CH$_3$)$_{2,Leu}$), 1.80–1.85 (m, 1H, 2*-H), 1.89–1.94 (m, 2H, 2*-H, 3*-H), 2.11–2.17 (m, 1H, 3*-H), 3.50 (dt, ^2J = 9.6 Hz, ^3J = 6.6 Hz, 1H, 1*-H), 3.67 (dt, ^2J = 9.6 Hz, ^3J = 6.9 Hz, 1H, 1*-H), 4.26 (dd, ^3J = 4.7 Hz, ^3J = 8.7 Hz, 1H, 4*-H), 4.29–4.32 (m, 1H, NHCH_{Leu}), 7.09–7.11 (m, 2H, 3-H), 7.16–7.19 (m, 2H, 2-H), 8.03 (d, ^3J = 7.9 Hz, 1H, OCONH), 12.38 (s, 1H, COOH); ^{13}C NMR (150 MHz, DMSO-d_6) δ 21.56, 23.25, 24.26 (CH(CH$_3$)$_{2,Leu}$), 24.76 (C-2*), 28.67 (C-3*), 46.52 (C-1*), 51.10 (NHCH$_{Leu}$), 58.68 (C-4*), 115.91 (d, ^2J = 23.4 Hz, C-3), 123.53 (d, ^3J = 8.7 Hz, C-2), 147.25 (d, ^4J = 2.8 Hz, C-1), 154.46 (OCONH), 159.23 (d, ^1J = 240.7 Hz, C-4), 170.41 (CHCO), 173.30 (COOH), one signal (CHCH$_{2,Leu}$) is obscured by the DMSO solvent signal; LC/MS (ESI): H_2O/MeCN, 90:10 to 0:100; *m/z* 367.1 [M + H]$^+$; 100% purity; Q-TOF: HRMS (ESI): *m/z* [M + H]$^+$ calcd. for $C_{18}H_{23}FN_2O_5$: 367.1664, found: 367.1668.

3-Chlorophenoxycarbonyl-leucyl-proline *tert*-butyl ester

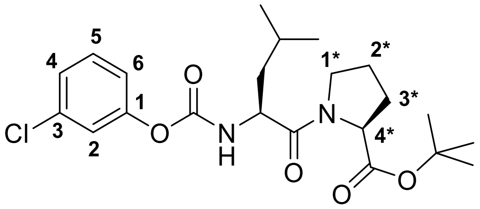

The synthesis was carried out according to GP13 using Cbz-Leu-Pro-O*t*Bu (0.31 g) for the hydrogenation and commercially available 3-chlorophenol (65 mg) for the subsequent carbamate formation. Column chromatography on silica gel using petrolum ether / EtOAc (3:1) as eluent afforded the product as a colorless resin (74 mg, 0.17 mmol, 34%). ^{1}H NMR (600 MHz, $CDCl_3$) δ 0.99 (2 × d, ^3J

= 6.6 Hz, 6H, $CH(C\underline{H}_3)_{2,Leu}$), 1.44 (s, 9H, $C(C\underline{H}_3)_3$), 1.52–1.57 (m, 1H, $CHC\underline{H}_{2,Leu}$), 1.58–1.64 (m, 1H, $CHC\underline{H}_{2,Leu}$), 1.78–1.84 (m, 1H, $C\underline{H}(CH_3)_{2,Leu}$), 1.92–2.06 (m, 3H, 2*-$\underline{H}$, 3*-$\underline{H}$), 2.16–2.21 (m, 1H, 3*-$\underline{H}$), 3.55–3.59 (m, 1H, 1*-$\underline{H}$), 3.71 (dt, ^2J = 9.7 Hz, ^3J = 6.9 Hz, 1H, 1*-$\underline{H}$), 4.40 (dd, ^3J = 4.5 Hz, ^3J = 8.4 Hz, 1H, 4*-$\underline{H}$), 4.52–4.57 (m, 1H, $NHC\underline{H}_{Leu}$), 5.78 (d, ^3J = 8.9 Hz, 1H, OCON$\underline{H}$), 6.98–7.01 (m, 1H, 6-$\underline{H}$), 7.11–7.16 (m, 2H, 2-$\underline{H}$, 4-$\underline{H}$), 7.24 (t, ^3J = 8.3 Hz, 1H, 5-$\underline{H}$); ^{13}C NMR (150 MHz, $CDCl_3$) δ 21.57, 23.44, 24.61 ($\underline{C}H(\underline{C}H_3)_2$), 24.81 ($\underline{C}$-2*), 27.95 ($C(\underline{C}H_3)_3$), 29.03 ($\underline{C}$-3*), 41.97 ($CH\underline{C}H_{2,Leu}$), 46.82 ($\underline{C}$-1*), 51.09 ($NH\underline{C}H_{Leu}$), 59.67 ($\underline{C}$-4*), 81.46 ($\underline{C}(CH_3)_3$), 119.97 ($\underline{C}$-6), 122.28 ($\underline{C}$-2), 125.60 ($\underline{C}$-4), 129.93 ($\underline{C}$-5), 134.44 ($\underline{C}$-3), 151.43 ($\underline{C}$-1), 154.13 (O$\underline{C}$ONH), 170.83, 170.90 (CH$\underline{C}$O); LC/MS (ESI): H_2O/MeCN, 90:10 to 0:100; *m/z* 439.1 $[M + H]^+$; 94% purity; Q-TOF: HRMS (ESI): *m/z* $[M + H]^+$ calcd. for $C_{22}H_{31}ClN_2O_5$: 439.1994, found: 439.2023.

3-Chlorophenoxycarbonyl-leucyl-proline (23)

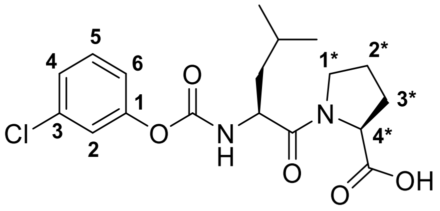

The synthesis was carried out according to GP8 using the corresponding *tert*-butyl ester (66 mg, 0.15 mmol). Column chromatography on silica gel afforded the desired compound as a colorless resin (48 mg, 0.13 mmol, 84%). ^{1}H NMR (600 MHz, $CDCl_3$) δ 0.98 (2 × d, ^3J = 6.6 Hz, 6H, $CH(C\underline{H}_3)_{2,Leu}$), 1.48–1.54 (m, 1H, $CHC\underline{H}_{2,Leu}$), 1.60–1.66 (m, 1H, $CHC\underline{H}_{2,Leu}$), 1.76–1.82 (m, 1H, $C\underline{H}(CH_3)_{2,Leu}$), 2.02–2.16 (m, 3H, 2*-$\underline{H}$, 3*-$\underline{H}$), 2.26–2.30 (m, 1H, 3*-$\underline{H}$), 3.56–3.60 (m, 1H, 1*-$\underline{H}$), 3.76 (dt, ^2J = 9.7 Hz, ^3J = 7.4 Hz, 1H, 1*-$\underline{H}$), 4.56 (dt, ^3J = 4.0 Hz, ^3J = 9.9 Hz, 1H, $NHC\underline{H}_{Leu}$), 4.61 (dd, ^3J = 3.9 Hz, ^3J = 8.1 Hz, 1H, 4*-$\underline{H}$), 5.78 (d, ^3J = 8.8 Hz, 1H, OCON$\underline{H}$), 6.99–7.02 (m, 1H, 6-$\underline{H}$), 7.14 (t, ^4J = 2.1 Hz, 1H, 2-$\underline{H}$), 7.15–7.17 (m, 1H, 4-$\underline{H}$), 7.24 (t, ^3J = 9.1 Hz, 1H, 5-$\underline{H}$), one signal (COO$\underline{H}$) is not visible; ^{13}C NMR (150 MHz, $CDCl_3$) δ 21.68, 23.32, 24.59 ($\underline{C}H(\underline{C}H_3)_2$), 24.91 ($\underline{C}$-2*), 27.82 ($\underline{C}$-3*), 41.62 ($CH\underline{C}H_{2,Leu}$), 47.35 ($\underline{C}$-1*), 51.11 ($NH\underline{C}H_{Leu}$), 59.47 ($\underline{C}$-4*), 119.93 ($\underline{C}$-6), 122.26 ($\underline{C}$-2), 125.80 ($\underline{C}$-4), 130.02 ($\underline{C}$-5), 134.53 ($\underline{C}$-3), 151.31 ($\underline{C}$-1), 154.13 (O$\underline{C}$ONH), 173.11, 173.16 (CH$\underline{C}$O); LC/MS (ESI): H_2O/MeCN, 90:10 to 0:100; *m/z* 383.2 $[M + H]^+$; 100% purity; Q-TOF: HRMS (ESI): *m/z* $[M + H]^+$ calcd. for $C_{18}H_{23}ClN_2O_5$: 383.1368, found: 383.1393.

3-Chloro-4-phenylacetamidophenoxycarbonyl-leucyl-proline *tert*-butyl ester

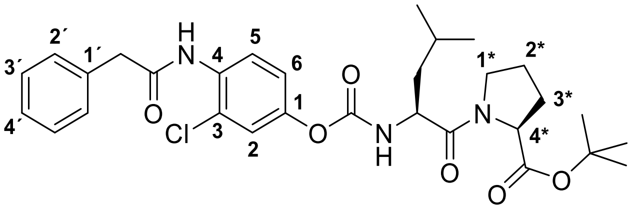

The synthesis was carried out according to GP13 using Cbz-Leu-Pro-O*t*Bu (0.31 g) for the hydrogenation and phenol **42** (131 mg) for the subsequent carbamate formation. Column chromatography on silica gel using petrolum ether / EtOAc (2:1) as eluent afforded the product as a colorless resin (214 mg, 0.37 mmol, 75%). ^{1}H NMR (600 MHz, $CDCl_3$) δ 0.98 (2 × d, ^{3}J = 6.6 Hz, 6H, $CH(CH_3)_{2,Leu}$), 1.43 (s, 9H, $C(CH_3)_3$), 1.52–1.59 (m, 2H, $CHCH_{2,Leu}$), 1.76–1.82 (m, 1H, $CH(CH_3)_{2,Leu}$), 1.90–2.06 (m, 3H, 2*-H, 3*-H), 2.14–2.22 (m, 1H, 3*-H), 3.53–3.58 (m, 1H, 1*-H), 3.68 (dt, ^{2}J = 7.2 Hz, ^{3}J = 6.8 Hz, 1H, 1*-H), 3.76 (s, 2H, $PhCH_2$), 4.39 (dd, ^{3}J = 4.5 Hz, ^{3}J = 8.6 Hz, 1H, 4*-H), 4.51 (dt, ^{3}J = 3.7 Hz, ^{3}J = 9.5 Hz, 1H, $NHCH_{Leu}$), 5.70 (d, ^{3}J = 8.9 Hz, 1H, OCONH), 6.99 (dd, ^{3}J = 9.0 Hz, ^{4}J = 2.6 Hz, 1H, 6-H), 7.10 (d, ^{4}J = 2.6 Hz, 1H, 2-H), 7.31–7.35 (m, 3H, 2´-H, 4´-H), 7.40 (t, ^{3}J = 7.5 Hz, 2H, 3´-H), 7.55 (s, 1H, NHPh), 8.33 (d, ^{3}J = 9.2 Hz, 1H, 5-H); ^{13}C NMR (150 MHz, $CDCl_3$) δ 21.58, 23.44, 24.60 ($CH(CH_3)_{2,Leu}$), 24.81 (C-2*), 27.95 ($C(CH_3)_3$), 29.03 (C-3*), 41.99 ($CHCH_{2,Leu}$), 45.06 ($PhCH_2$), 46.80 (C-1*), 51.09 ($NHCH_{Leu}$), 59.66 (C-4*), 81.44 ($C(CH_3)_3$), 120.88 (C-6), 121.34 (C-2), 122.29 (C-5), 122.64 (C-4´), 127.89 (C-3), 129.35, 129.68 (C-2´, C-3´), 131.85 (C-4), 133.87 (C-1´), 146.73 (C-1), 154.15 (OCONH), 168.97 (CH_2CO), 170.76, 170.91 (CHCO); LC/MS (ESI): H_2O/MeCN, 90:10 to 0:100; *m/z* 572.4 $[M + H]^+$; 98% purity; Q-TOF: HRMS (ESI): *m/z* $[M + H]^+$ calcd. for $C_{30}H_{38}ClN_3O_6$: 572.2522, found: 572.2478.

3-Chloro-4-phenylacetamidophenoxycarbonyl-leucyl-proline (24)

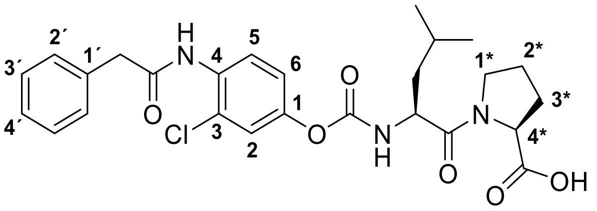

The synthesis was carried out according to GP8 using the corresponding *tert*-butyl ester (57 mg, 0.10 mmol). Column chromatography on silica gel afforded the desired compound as a colorless solid (46 mg, 0.09 mmol, 89%). mp 86–90 °C; ^{1}H NMR (500 MHz, DMSO-d_6) δ 0.92 (2 × d, ^{3}J = 6.6 Hz, 6H, $CH(CH_3)_{2,Leu}$), 1.40–1.46 (m, 1H, $CHCH_{2,Leu}$), 1.51–1.57 (m, 1H, $CHCH_{2,Leu}$), 1.69–1.77 (m, 1H, $CH(CH_3)_{2,Leu}$), 1.79–1.85 (m, 1H, 2*-H), 1.88–1.95 (m, 2H, 2*-H, 3*-H), 2.10–2.16 (m, 1H, 3*-H), 3.50 (dt, ^{2}J = 9.7 Hz, ^{3}J = 6.6 Hz, 1H, 1*-H), 3.67 (dt, ^{2}J = 9.6 Hz, ^{3}J = 7.1 Hz, 1H, 1*-H), 3.71 (s, 2H, $PhCH_2$), 4.26 (dd, ^{3}J = 4.7 Hz, ^{3}J = 8.7 Hz, 1H, 4*-H), 4.28–4.33 (m, 1H¸ $NHCH_{Leu}$), 7.04 (dd, ^{3}J = 8.8 Hz, ^{4}J = 2.6 Hz, 1H, 6-H), 7.22–7.27 (m, 2H, 3´-H), 7.30–7.36 (m, 4H, 2-H, 2´-H, 4´-H), 7.61 (d, ^{3}J = 8.8 Hz, 1H, 5-H), 8.10 (d, ^{3}J = 7.9 Hz, 1H, OCONH), 9.67 (s, 1H, NHPh), 12.13 (s, 1H, COOH); ^{13}C NMR (125 MHz, DMSO-d_6) δ 21.55, 23.23, 24.24 ($CH(CH_3)_{2,Leu}$), 24.73 (C-2*), 28.66 (C-3*), 42.61 ($CHCH_{2,Leu}$), 46.50 (C-1*), 51.15 ($NHCH_{Leu}$), 58.72 (C-4*), 120.91 (C-6), 122.71 (C-2), 126.69 (C-5), 126.99 (C-4´), 127.08 (C-3), 128.44 (C-3´), 129.28 (C-2´), 132.04 (C-4), 135.95 (C-1´), 148.31 (C-1), 154.07 (OCONH), 169.61 (CH_2CO), 170.28 (CHCO), 173.29 (COOH), oe signal ($PhCH_2$) is not visible; LC/MS (ESI): H_2O/MeCN, 90:10 to 0:100; *m/z* 516.4 $[M + H]^+$; 99% purity; Q-TOF: HRMS (ESI): *m/z* $[M + H]^+$ calcd. for $C_{26}H_{30}ClN_3O_6$: 516.1896, found: 516.1884.

3-Bromo-4-phenylacetamidophenoxycarbonyl-leucyl-proline *tert*-butyl ester

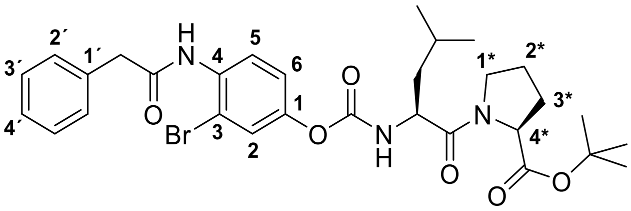

The synthesis was carried out according to GP13 using Cbz-Leu-Pro-O*t*Bu (0.31 g) for the hydrogenation and phenol **50** (153 mg) for the subsequent carbamate formation. Column chromatography on silica gel using petrolum ether / EtOAc (2:1) as eluent afforded the product as a colorless resin (145 mg, 0.24 mmol, 47%). ^{1}H NMR (600 MHz, $CDCl_3$) δ 0.93 (2 × d, ^{3}J = 6.5 Hz, 6H, CH(C<u>H</u>$_3$)$_{2,Leu}$), 1.39 (s, 9H, C(C<u>H</u>$_3$)$_3$), 1.45–1.50 (m, 1H, CHC<u>H</u>$_{2,Leu}$), 1.51–1.57 (m, 1H, CHC<u>H</u>$_{2,Leu}$), 1.72–1.77 (m, 1H, C<u>H</u>(CH$_3$)$_{2,Leu}$), 1.87–1.94 (m, 2H, 2*-<u>H</u>), 1.96–2.00 (m, 1H, 3*-<u>H</u>), 2.11 – 2.15 (m, 1H, 3*-<u>H</u>), 3.48–3.54 (m, 1H, 1*-<u>H</u>), 3.64 (dt, ^{2}J = 9.6 Hz, ^{3}J = 6.7 Hz, 1H, 1*-<u>H</u>), 3.71 (s, 2H, PhC<u>H</u>$_2$), 4.34 (dd, ^{3}J = 4.6 Hz, ^{3}J = 8.6 Hz, 1H, 4*-<u>H</u>), 4.47 (dt, ^{3}J = 3.7 Hz, ^{3}J = 9.6 Hz, 1H, NHC<u>H</u>$_{Leu}$), 5.72 (d, ^{3}J = 8.9 Hz, 1H, OCON<u>H</u>), 6.99 (dd, ^{3}J = 9.1 Hz, ^{4}J = 2.7 Hz, 1H, 6-<u>H</u>), 7.20 (d, ^{4}J = 2.3 Hz, 1H, 2-<u>H</u>), 7.29 (d, ^{3}J = 7.6 Hz, 3H, 2´-<u>H</u>, 4´-<u>H</u>), 7.35 (t, ^{3}J = 7.5 Hz, 2H, 3´-<u>H</u>), 7.50 (s, 1H, N<u>H</u>Ph), 8.27 (d, ^{3}J = 9.1 Hz, 1H, 5-<u>H</u>); ^{13}C NMR (150 MHz, $CDCl_3$) δ 21.57, 23.42, 24.59 (<u>C</u>H(<u>C</u>H$_3$)$_{2,Leu}$), 24.80 (<u>C</u>-2*), 27.94 (C(<u>C</u>H$_3$)$_3$), 29.02 (<u>C</u>-3*), 41.86 (CH<u>C</u>H$_{2,Leu}$), 45.07 (<u>C</u>-1*), 46.84 (Ph<u>C</u>H$_2$), 51.09 (NH<u>C</u>H$_{Leu}$), 59.70 (<u>C</u>-4*), 81.50 (<u>C</u>(CH$_3$)$_3$), 112.63 (<u>C</u>-6), 121.40 (<u>C</u>-2), 121.48 (<u>C</u>-5), 125.34 (<u>C</u>-3), 127.92 (<u>C</u>-4´), 129.38, 129.83 (<u>C</u>-2´, <u>C</u>-3´), 132.95 (<u>C</u>-4), 133.81 (<u>C</u>-1´), 146.89 (<u>C</u>-1), 154.19 (O<u>C</u>ONH), 169.10 (CH$_2$<u>C</u>O), 170.86, 170.94 (CH<u>C</u>O); LC/MS (ESI): H_2O/MeCN, 90:10 to 0:100; *m/z* 616.3 [M + H]$^{+}$; 92% purity; Q-TOF: HRMS (ESI): *m/z* [M + H]$^{+}$ calcd. for $C_{30}H_{38}BrN_3O_6$: 616.2017, found: 616.1995.

3-Bromo-4-phenylacetamidophenoxycarbonyl-leucyl-proline

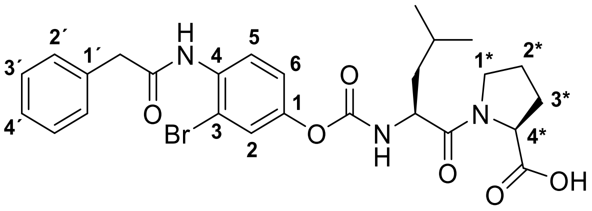

The synthesis was carried out according to GP8 using the corresponding *tert*-butyl ester (62 mg, 0.10 mmol). Column chromatography on silica gel afforded the desired compound as a colorless solid (52 mg, 0.09 mmol, 93%). mp 82–85 °C; ^{1}H NMR (600 MHz, $CDCl_3$-*d*) δ 0.96 (2 × d, ^{3}J = 6.6 Hz, 6H, CH(C<u>H</u>$_3$)$_{2,Leu}$), 1.46–1.52 (m, 1H, CHC<u>H</u>$_{2,Leu}$), 1.58–1.63 (m, 1H, CHC<u>H</u>$_{2,Leu}$), 1.73–1.80 (m, 1H, C<u>H</u>(CH$_3$)$_{2,Leu}$), 1.99–2.06 (m, 2H, 2*-<u>H</u>), 2.09–2.16 (m, 1H, 3*-<u>H</u>), 2.20–2.26 (m, 1H, 3*-<u>H</u>), 3.54–3.59 (m, 1H, 1*-<u>H</u>), 3.71–3.75 (m, 1H, 1*-<u>H</u>), 3.77 (s, 2H, PhC<u>H</u>$_2$), 4.53 (dt, ^{3}J = 4.1 Hz, ^{3}J = 9.7 Hz, 1H, NHC<u>H</u>$_{Leu}$), 4.58 (dd, ^{3}J = 4.0 Hz, ^{3}J = 8.3 Hz, 1H, 4*-<u>H</u>), 5.84 (d, ^{3}J = 8.8 Hz, 1H, OCON<u>H</u>), 7.04 (dd, ^{3}J = 9.1 Hz, ^{4}J = 2.7 Hz, 1H, 6-<u>H</u>), 7.25 (d, ^{4}J = 3.1 Hz, 1H, 2-<u>H</u>), 7.34 (d, ^{3}J = 7.2 Hz, 3H, 2´-<u>H</u>, 4´-<u>H</u>), 7.40 (t, ^{3}J = 7.6 Hz, 2H, 3´-<u>H</u>), 7.55 (s, 1H, N<u>H</u>Ph), 8.33 (d, ^{3}J = 9.1 Hz, 1H, 5-<u>H</u>), one signal (COO<u>H</u>)

is not visible; ^{13}C NMR (150 MHz, $CDCl_3$) δ 21.65, 23.31, 24.55 (CH(CH_3)$_{2,Leu}$), 24.88 (C-2*), 27.97 (C-3*), 41.49 (CH$CH_{2,Leu}$), 45.06 (C-1*), 47.27 (PhCH_2), 51.10 (NHCH_{Leu}), 59.41 (C-4*), 112.68 (C-6), 121.46 (C-2, C-5), 125.32 (C-3), 127.95 (C-4´), 129.40, 129.83 (C-2´, C-3´), 133.07 (C-4), 133.78 (C-1´), 146.76 (C-1), 154.18 (OCONH), 169.17 (CH_2CO), 172.88, 173.32 (COOH); LC/MS (ESI): H_2O/MeCN, 90:10 to 0:100; *m/z* 560.1 $[M + H]^+$; 95% purity; Q-TOF: HRMS (ESI): *m/z* $[M + H]^+$ calcd. for $C_{26}H_{30}BrN_3O_6$: 560.1391, found: 560.1370.

3-Chloro-4-(4-phenylbenzamido)phenoxycarbonyl-leucyl-proline *tert*-butyl ester

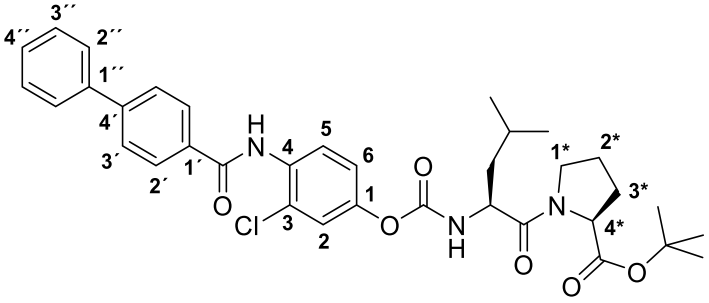

The synthesis was carried out according to GP13 using Cbz-Leu-Pro-O*t*Bu (0.31 g) for the hydrogenation and phenol **44** (162 mg) for the subsequent carbamate formation. Column chromatography on silica gel using petrolum ether / EtOAc (2:1) as eluent afforded the product as a colorless resin (86 mg, 0.14 mmol, 27%). ^{1}H NMR (600 MHz, $CDCl_3$) δ 1.00 (2 × d, ^3J = 6.6 Hz, 6H, CH(CH_3)$_{2,Leu}$), 1.45 (s, 9H, C(CH_3)$_3$), 1.49–1.53 (m, 1H, CH$CH_{2,Leu}$), 1.59–1.65 (m, 1H, CH$CH_{2,Leu}$), 1.80–1.87 (m, 1H, CH(CH_3)$_{2,Leu}$), 1.92–2.08 (m, 3H, 2*-H, 3*-H), 2.16–2.23 (m, 1H, 3*-H), 3.55–3.61 (m, 1H, 1*-H), 3.72 (dt, ^2J = 9.9 Hz, ^3J = 6.9 Hz, 1H, 1*-H), 4.41 (dd, ^3J = 4.5 Hz, ^3J = 8.5 Hz, 1H, 4*-H), 4.56 (dt, ^3J = 3.5 Hz, ^3J = 10.1 Hz, 1H, NHCH_{Leu}), 5.77 (d, ^3J = 8.9 Hz, 1H, OCONH), 7.10 (dd, ^3J = 9.0 Hz, ^4J = 2.6 Hz, 1H, 6-H), 7.26 (d, ^4J = 2.6 Hz, 1H, 2-H), 7.39 (t, ^3J = 7.4 Hz, 1H, 4´´-H), 7.47 (t, ^3J = 7.7 Hz, 2H, 3´´-H), 7.62 (d, ^3J = 7.2 Hz, 2H, 2´´-H), 7.72 (d, ^3J = 8.3 Hz, 2H, 2´-H), 7.96 (d, ^3J = 8.3 Hz, 2H, 3´-H), 8.39 (s, 1H, NHPh), 8.55 (d, ^3J = 9.1 Hz, 1H, 5-H); ^{13}C NMR (150 MHz, $CDCl_3$) δ 21.60, 23.46, 24.63 (CH(CH_3)$_{2,Leu}$), 24.83 (C-2*), 27.96 (C(CH_3)$_3$), 29.04 (C-3*), 42.00 (CH$CH_{2,Leu}$), 46.83 (C-1*), 51.13 (NHCH_{Leu}), 59.68 (C-4*), 81.46 (C(CH_3)$_3$), 121.13 (C-6), 121.70 (C-2), 122.46 (C-5), 122.94 (C-3), 127.24, 127.58, 127.62 (C-3´, C-2´´, C-3´´), 128.18 (C-4´´), 128.97 (C-2´), 132.18 (C-4), 133.08 (C-1´), 139.78 (C-1´´), 145.05 (C-4´), 146.82 (C-1), 154.22 (OCONH), 164.85 (PhCO), 170.80, 170.92 (CHCO); LC/MS (ESI): H_2O/MeCN, 90:10 to 0:100; *m/z* 634.4 $[M + H]^+$; 97% purity; Q-TOF: HRMS (ESI): *m/z* $[M + H]^+$ calcd. for $C_{35}H_{40}ClN_3O_6$: 634.2678, found: 634.2640.

3-Chloro-4-(4-phenylbenzamido)phenoxycarbonyl-leucyl-proline (25)

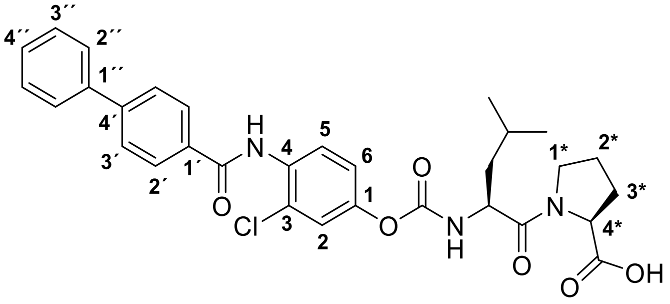

The synthesis was carried out according to GP8 using the corresponding *tert*-butyl ester (76 mg, 0.12 mmol). Column chromatography on silica gel afforded the desired compound as a colorless solid (65 mg, 0.11 mmol, 94%). mp 96–100 °C; ^{1}H NMR (600 MHz, $CDCl_3$) δ 0.99 (2 × d, ^3J = 6.6 Hz, 6H, $CH(CH_3)_{2,Leu}$), 1.51–1.56 (m, 1H, $CHCH_{2,Leu}$), 1.61–1.68 (m, 1H, $CHCH_{2,Leu}$), 1.79–1.84 (m, 1H, $CH(CH_3)_{2,Leu}$), 2.02–2.12 (m, 2H, 2*-H), 2.14–2.24 (m, 2H, 3*-H), 3.58–3.63 (m, 1H, 1*-H), 3.79 (dt, ^2J = 9.9 Hz, ^3J = 7.4 Hz, 1H, 1*-H), 4.52–4.63 (m, 2H, 4*-H, $NHCH_{Leu}$), 6.02 (d, ^3J = 8.7 Hz, 1H, OCONH), 7.10 (dd, ^3J = 9.0 Hz, ^4J = 2.6 Hz, 1H, 6-H), 7.25 (d, ^3J = 2.6 Hz, 1H, 2-H), 7.39 (t, ^3J = 7.4 Hz, 1H, 4´´-H), 7.46 (t, ^3J = 7.7 Hz, 2H, 3´´-H), 7.61 (d, ^3J = 7.2 Hz, 2H, 2´´-H), 7.71 (d, ^3J = 8.3 Hz, 2H, 2´-H), 7.96 (d, ^3J = 8.3 Hz, 2H, 3´-H), 8.40 (s, 1H, NHPh), 8.55 (d, ^3J = 9.0 Hz, 1H, 5-H), one signal (COOH) is not visible; ^{13}C NMR (151 MHz, $CDCl_3$) δ 21.65, 23.31, 24.58 ($CH(CH_3)_{2,Leu}$), 24.89 (C-2*), 28.11 (C-3*), 41.35 ($CHCH_{2,Leu}$), 47.33 (C-1*), 51.19 ($NHCH_{Leu}$), 59.42 (C-4*), 121.11 (C-6), 121.81 (C-2), 122.43 (C-5), 123.06 (C-3), 127.23, 127.59, 127.63 (C-3´, C-2´´, C-3´´), 128.21 (C-4´´), 128.98 (C-2´), 132.28 (C-4), 132.97 (C-1´), 139.74 (C-1´´), 145.11 (C-4´), 146.70 (C-1), 154.30 (OCONH), 164.96 (PhCO), 172.96 (CHCO), 173.46 (COOH); LC/MS (ESI): H_2O/MeCN, 90:10 to 0:100; *m/z* 578.3 $[M + H]^+$; 100% purity; Q-TOF: HRMS (ESI): *m/z* $[M - H]^-$ calcd. for $C_{31}H_{32}ClN_3O_6$: 576.1907, found: 576.1938.

3-Bromo-4-(3,3-dibenzylureido)phenoxycarbonyl-leucyl-proline *tert*-butyl ester

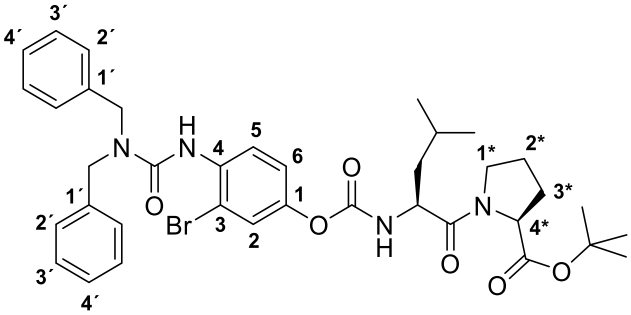

The synthesis was carried out according to GP13 using Cbz-Leu-Pro-O*t*Bu (0.31 g) for the hydrogenation and phenol **52** (206 mg) for the subsequent carbamate formation. Column chromatography on silica gel using petrolum ether / EtOAc (2:1) as eluent afforded the product as a colorless resin (260 mg, 0.36 mmol, 72%). ^{1}H NMR (600 MHz, $CDCl_3$) δ 0.97 (2 × d, ^3J = 6.5 Hz, 6H, $CH(CH_3)_{2,Leu}$), 1.43 (s, 9H, $C(CH_3)_3$), 1.50–1.55 (m, 1H, $CHCH_{2,Leu}$), 1.55–1.61 (m, 1H, $CHCH_{2,Leu}$),

1.76–1.83 (m, 1H, CH(CH$_3$)$_{2,Leu}$), 1.91–1.99 (m, 2H, 2*-H), 2.00–2.06 (m, 1H, 3*-H), 2.13–2.20 (m, 1H, 3*-H), 3.53–3.58 (m, 1H, 1*-H), 3.69 (dt, ^2J = 9.9 Hz, ^3J = 6.8 Hz, 1H, 1*-H), 4.39 (dd, ^3J = 4.4 Hz, ^3J = 8.5 Hz, 1H, 4*-H), 4.52 (dt, ^3J = 3.7, ^3J = 9.7 Hz, 1H, NHCH$_{Leu}$), 4.62 (s, 4H, N(CH$_2$)$_2$, 5.69 (d, ^3J = 8.9 Hz, 1H, OCONH), 6.86 (s, 1H, NHPh), 7.02 (dd, ^3J = 9.1 Hz, ^4J = 2.6 Hz, 1H, 6-H), 7.20 (d, ^4J = 2.7 Hz, 1H, 2-H), 7.26–7.32 (m, 6H, 2´-H, 4´-H), 7.34 (t, ^3J = 7.5 Hz, 4H, 3´-H), 8.18 (d, ^3J = 9.1 Hz, 1H, 5-H); ^{13}C NMR (150 MHz, CDCl$_3$) δ 21.60, 23.43, 24.58 (CH(CH$_3$)$_{2,Leu}$), 24.80 (C-2*), 27.94 (C(CH$_3$)$_3$), 29.02 (C-3*), 41.97 (CHCH$_{2,Leu}$), 46.80 (C-1*), 50.79 (N(CH$_2$)$_2$), 51.05 (NHCH$_{Leu}$), 59.65 (C-4*), 81.42 (C(CH$_3$)$_3$), 112.37 (C-6), 121.37 (C-2, C-5), 125.09 (C-3), 127.32 (C-2´), 127.80 (C-4´), 128.95 (C-3´), 134.60 (C-4), 136.75 (C-1´), 145.80 (C-1), 154.36 (OCONH), 155.36 (NCONH), 170.85, 170.94 (CHCO); LC/MS (ESI): H$_2$O/MeCN, 90:10 to 0:100; *m/z* 721.1 [M + H]$^+$; 95% purity; Q-TOF: HRMS (ESI): *m/z* [M + H]$^+$ calcd. for C$_{37}$H$_{45}$BrN$_4$O$_6$: 721.2595, found: 721.2555.

3-Bromo-4-(3,3-dibenzylureido)phenoxycarbonyl-leucyl-proline (26)

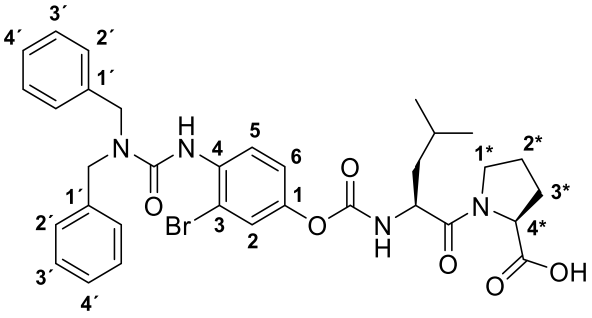

The synthesis was carried out according to GP8 using the corresponding *tert*-butyl ester (130 mg, 0.18 mmol). Column chromatography on silica gel afforded the desired compound as a slightly brown resin (81 mg, 0.12 mmol, 68%). ^{1}H NMR (600 MHz, CDCl$_3$) δ 0.96 (2 × d, ^3J = 6.6 Hz, 6H, CH(CH$_3$)$_{2Leu}$), 1.47–1.52 (m, 1H, CHCH$_{2,Leu}$), 1.57–1.63 (m, 1H, CHCH$_{2,Leu}$), 1.74–1.81 (m, 1H, CH(CH$_3$)$_{2,Leu}$), 1.99–2.06 (m, 2H, 2*-H), 2.10–2.15 (m, 1H, 3*-H), 2.20–2.25 (m, 1H, 3*-H), 3.55–3.59 (m, 1H, 1*-H), 3.75 (m, 1H, 1*-H), 4.53 (dt, ^3J = 4.0 Hz, ^3J = 9.6 Hz, 1H, NHCH$_{Leu}$), 4.59 (dd, ^3J = 8.3 Hz, ^3J = 3.8 Hz, 1H, 4*-H), 4.63 (s, 4H, N(CH$_2$)$_2$), 5.84 (d, ^3J = 8.8 Hz, 1H, OCONH), 6.86 (s, 1H, NHPh), 7.03 (dd, ^3J = 9.0 Hz, ^4J = 2.7 Hz, 1H, 6-H), 7.21 (d, ^4J = 2.7 Hz, 1H, 2-H), 7.32–7.28 (m, 6H, 2´-H, 4´-H), 7.35 (t, ^3J = 7.4 Hz, 4H, 3´-H), 8.18 (d, ^3J = 9.1 Hz, 1H, 5-H), one signal (COOH) is not visible; ^{13}C NMR (150 MHz, CDCl$_3$) δ 21.69, 23.29, 24.54 (CH(CH$_3$)$_{2,Leu}$), 24.86 (C-2*), 27.98 (C(CH$_3$)$_3$), 41.48 (CHCH$_{2,Leu}$), 47.27 (C-1*), 50.83 (N(CH$_2$)$_2$), 51.07 (NHCH$_{Leu}$), 59.36 (C-4*), 112.45 (C-6), 121.36, 121.49 (C-2, C-5), 125.08 (C-3), 127.32 (C-2´), 127.83 (C-4´), 128.97 (C-3´), 134.70 (C-4), 136.69 (C-1´), 145.69 (C-1), 154.40 (OCONH), 155.41 (NCONH), 172.99, 173.68 (CHCO); LC/MS (ESI): H$_2$O/MeCN, 90:10 to 0:100; *m/z* 665.3 [M + H]$^+$; 99% purity; Q-TOF: HRMS (ESI): *m/z* [M + H]$^+$ calcd. for C$_{32}$H$_{37}$BrN$_4$O$_6$: 665.1969, found: 665.1952.

3-Bromo-4-(2-diphenylacetamido)phenoxycarbonyl-leucyl-proline *tert*-butyl ester

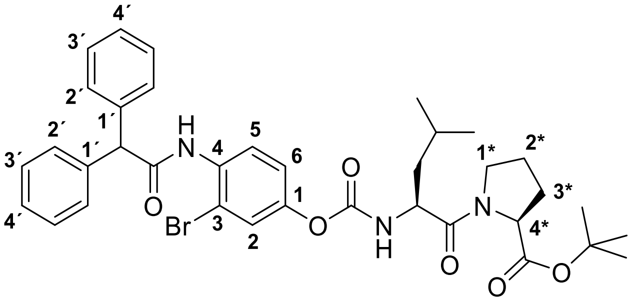

The synthesis was carried out according to GP13 using Cbz-Leu-Pro-O*t*Bu (0.31 g) for the hydrogenation and phenol **51** (191 mg) for the subsequent carbamate formation. Column chromatography on silica gel using CH_2Cl_2 / MeOH (39:1) as eluent afforded the product as a colorless resin (178 mg, 0.26 mmol, 51%). ^{1}H NMR (600 MHz, $CDCl_3$) δ 0.98 (2 × d, ^3J = 6.6 Hz, 6H, CH(C$\underline{H}_3$)$_{2,Leu}$), 1.43 (s, 9H, C(C$\underline{H}_3$)$_3$), 1.49–1.55 (m, 1H, CHC$\underline{H}_{2,Leu}$), 1.56–1.62 (m, 1H, CHC$\underline{H}_{2,Leu}$), 1.77–1.83 (m, 1H, C$\underline{H}$(CH$_3$)$_{2,Leu}$), 1.91–1.99 (m, 2H, 2*-$\underline{H}$), 2.00–2.05 (m, 1H, 3*-$\underline{H}$), 2.15–2.21 (m, 1H, 3*-$\underline{H}$), 3.56 (dt, ^2J = 9.4 Hz, ^3J = 7.9 Hz, 1H, 1*-$\underline{H}$), 3.71 (dt, ^2J = 9.6 Hz, ^3J = 7.1 Hz, 1H, 1*-$\underline{H}$), 4.39 (dd, ^3J = 4.4 Hz, ^3J = 8.4 Hz, 1H, 4*-$\underline{H}$), 4.52 (dt, ^3J = 3.2 Hz, ^3J = 10.3 Hz, 1H, NHC$\underline{H}_{Leu}$), 5.14 (s, 1H, Ph$_2$C$\underline{H}$), 5.93 (d, ^3J = 9.0 Hz, 1H, OCON$\underline{H}$), 7.05 (dd, ^3J = 9.0 Hz, ^4J = 2.2 Hz, 1H, 6-$\underline{H}$), 7.27 (d, ^4J = 2.3 Hz, 1H, 2-$\underline{H}$), 7.28–7.33 (m, 6H, 2´-$\underline{H}$, 4´-$\underline{H}$), 7.36 (t, ^3J = 7.6 Hz, 4H, 3´-$\underline{H}$), 7.78 (s, 1H, N$\underline{H}$Ph), 8.40 (d, ^3J = 9.0 Hz, 1H, 5-$\underline{H}$); ^{13}C NMR (150 MHz, $CDCl_3$) δ 21.57, 23.40, 24.57 ($\underline{C}$H($\underline{C}$H$_3$)$_{2,Leu}$), 24.79 ($\underline{C}$-2*), 27.92 (C($\underline{C}$H$_3$)$_3$), 29.00 ($\underline{C}$-3*), 41.77 (CH$\underline{C}$H$_{2,Leu}$), 46.85 ($\underline{C}$-1*), 51.09 (NH$\underline{C}$H$_{Leu}$), 59.70 (Ph$_2$$\underline{C}$H), 60.48 ($\underline{C}$-4*), 81.48 ($\underline{C}$(CH$_3$)$_3$), 112.69 ($\underline{C}$-6), 121.43 ($\underline{C}$-2), 121.56 ($\underline{C}$-5), 125.40 ($\underline{C}$-3), 127.64 ($\underline{C}$-4´), 129.04, 129.07 ($\underline{C}$-2´, $\underline{C}$-3´), 132.94 ($\underline{C}$-4), 138.63 ($\underline{C}$-1´), 147.01 ($\underline{C}$-1), 154.25 (O$\underline{C}$ONH), 170.20, 170.84, 171.04 (CH$\underline{C}$O); LC/MS (ESI): H_2O/MeCN, 90:10 to 0:100; *m/z* 692.5 $[M + H]^+$; 93% purity; Q-TOF: HRMS (ESI): *m/z* $[M + H]^+$ calcd. for $C_{36}H_{42}BrN_3O_6$: 692.2330, found: 692.2321.

3-Bromo-4-(2-diphenylacetamido)phenoxycarbonyl-leucyl-proline (27)

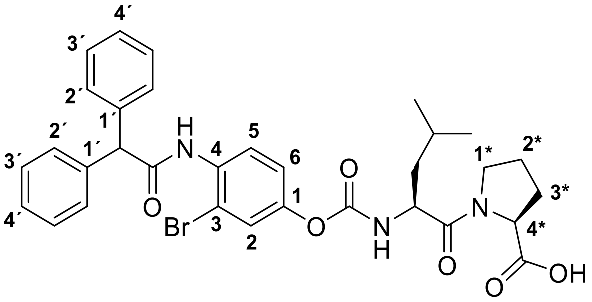

The synthesis was carried out according to GP8 using the corresponding *tert*-butyl ester (70 mg, 0.10 mmol). Column chromatography on silica gel afforded the desired compound as a colorless solid (50 mg, 0.08 mmol, 79%). mp 94–97 °C; ^{1}H NMR (600 MHz, $CDCl_3$) δ 0.96 (2 × d, ^3J = 6.6 Hz, 6H, CH(C$\underline{H}_3$)$_{2,Leu}$), 1.47–1.53 (m, 1H, CHC$\underline{H}_{2,Leu}$), 1.57–1.64 (m, 1H, CHC$\underline{H}_{2,Leu}$), 1.73–1.81 (m, 1H, C$\underline{H}$(CH$_3$)$_{2,Leu}$), 1.98–2.05 (m, 2H, 2*-$\underline{H}$), 2.10–2.23 (m, 2H, 3*-$\underline{H}$), 3.57 (dt, ^2J = 9.3 Hz, ^3J = 8.4 Hz,

1H, 1*-H), 3.75 (dt, ^2J = 9.0 Hz, ^3J = 8.1 Hz, 1H, 1*-H), 4.53 (dt, ^3J = 3.6 Hz, ^3J = 9.5 Hz, 1H, NHCH_{Leu}), 4.58 (dd, ^3J = 3.7 Hz, ^3J = 7.7 Hz, 1H, 4*-H), 5.14 (s, 1H, Ph_2CH), 5.90 (d, ^3J = 8.8 Hz, 1H, OCONH), 7.06 (dd, ^3J = 9.0 Hz, ^4J = 2.2 Hz, 1H, 6-H), 7.26-7.28 (m, 1H, 2-H), 7.28-7.33 (m, 6H, 2´-H, 4´-H), 7.36 (t, ^3J = 7.6 Hz, 4H, 3´-H), 7.78 (s, 1H, NHPh), 8.41 (d, ^3J = 9.1 Hz, 1H, 5-H), one signal (COOH) is not visible; ^{13}C NMR (150 MHz, $CDCl_3$) δ 21.66, 23.29, 24.53 ($CH(CH_3)_{2,Leu}$), 24.85 (C-2*), 28.11 (C-3*), 41.46 ($CHCH_{2,Leu}$), 47.21 (C-1*), 51.09 ($NHCH_{Leu}$), 59.26 (Ph_2CH), 60.46 (C-4*), 112.74 (C-6), 121.49 (C-2), 121.52 (C-5), 125.37 (C-3), 127.67 (C-4´), 129.05, 129.07 (C-2´, C-3´), 133.06 (C-4), 138.60 (C-1´), 146.87 (C-1), 154.20 (OCONH), 170.26, 172.72 (CHCO), 174.04 (COOH); LC/MS (ESI): H_2O/MeCN, 90:10 to 0:100; *m/z* 636.3 $[M + H]^+$; 100% purity; Q-TOF: HRMS (ESI): *m/z* $[M + H]^+$ calcd. for $C_{32}H_{35}BrN_3O_6$: 636.1704, found: 636.1695.

3-Chloro-4-(2-diphenylacetamido)phenoxycarbonyl-leucyl-proline *tert*-butyl ester

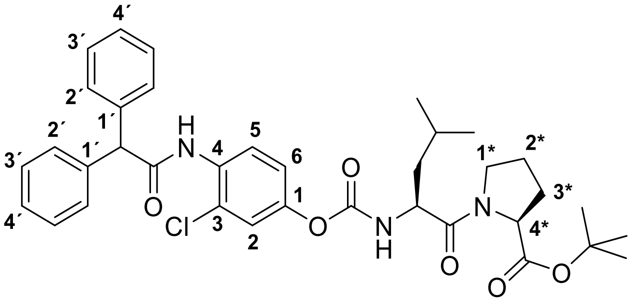

The synthesis was carried out according to GP13 using Cbz-Leu-Pro-O*t*Bu (0.31 g) for the hydrogenation and phenol **46** (169 mg) for the subsequent carbamate formation. Column chromatography on silica gel using petrolum ether / EtOAc (2:1) as eluent afforded the product as a colorless resin (190 mg, 0.29 mmol, 59%). ^{1}H NMR (600 MHz, DMSO-d_6) δ 0.88 (2 × d, ^3J = 6.6 Hz, 6H, $CH(CH_3)_{2,Leu}$), 1.24–1.30 (m, 2H, $CHCH_{2,Leu}$), 1.36 (s, 9H, $C(CH_3)_3$), 1.58–1.66 (m, 1H, $CH(CH_3)_{2,Leu}$), 1.73–1.79 (m, 1H, 2*-H), 1.86–1.94 (m, 2H, 2*-H, 3*H), 2.10–2.17 (m, 1H, 3*H), 3.44 (dt, ^2J = 9.8 Hz, ^3J = 6.9 Hz, 1H, 1*-H), 3.67 (dt, ^2J = 9.6 Hz, ^3J = 6.9 Hz, 1H, 1*-H), 4.15 (dd, ^3J = 5.0 Hz, ^3J = 8.6 Hz, 1H, 4*H), 4.38 (dt, ^3J = 4.4 Hz, ^3J = 9.3 Hz, 1H, $NHCH_{Leu}$), 5.25 (s, 1H, Ph_2CH), 6.19 (d, ^3J = 8.7 Hz, 1H, 5-H), 6.69 (dd, ^3J = 8.7 Hz, ^4J = 2.7 Hz, 1H, 6-H), 6.83 (d, ^4J = 2.7 Hz, 1H, 2-H), 7.22–7.26 (m, 2H, 4´-H), 7.28 (d, ^3J = 8.8 Hz, 1H, OCONH), 7.31–7.37 (m, 8H, 2´-H, 3´-H), 9.70 (s, 1H, NHPh); ^{13}C NMR (150 MHz, DMSO-d_6) δ 21.86, 23.34, 24.21 ($CH(CH_3)_{2,Leu}$), 24.69 (C-2*), 27.71 ($C(CH_3)_3$), 28.73 (C-3*), 41.54 ($CHCH_{2,Leu}$), 46.47 (C-1*), 49.07 ($NHCH_{Leu}$), 56.53 (Ph_2CH), 59.49 (C-4*), 80.37 ($C(CH_3)_3$), 114.56 (C-6), 115.75 (C-2), 126.09 (C-5), 126.90 (C-4´), 128.45 (C-2´), 128.56 (C-3), 128.71 (C-3´), 128.81 (C-4), 140.21 (C-1´), 156.04 (C-1), 157.28 (OCONH), 170.40, 171.08, 171.48 (CHCO); LC/MS (ESI): H_2O/MeCN, 90:10 to 0:100; *m/z* 648.4 $[M + H]^+$; 96% purity; Q-TOF: HRMS (ESI): *m/z* $[M - H]^-$ calcd. for $C_{36}H_{40}ClN_3O_6$: 646.2689, found: 646.2700.

3-Chloro-4-(2-diphenylacetamido)phenoxycarbonyl-leucyl-proline (28)

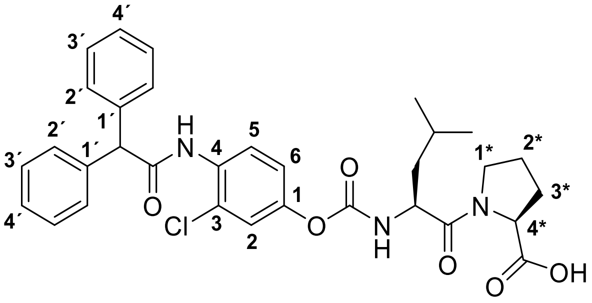

The synthesis was carried out according to GP8 using the corresponding *tert*-butyl ester (65 mg, 0.10 mmol). Column chromatography on silica gel afforded the desired compound as a colorless solid (45 mg, 0.08 mmol, 76%). mp 98–104 °C; ^{1}H NMR (600 MHz, DMSO-d_6) δ 0.91 (2 × d, ^{3}J = 6.6 Hz, 6H, CH(C$\underline{H}_3$)$_{2,Leu}$), 1.40–1.45 (m, 1H, CHC$\underline{H}_{2,Leu}$), 1.51–1.56 (m, 1H, CHC$\underline{H}_{2,Leu}$), 1.70–1.76 (m, 1H, C$\underline{H}$(CH$_3$)$_{2,Leu}$), 1.78–1.84 (m, 1H, 2*-$\underline{H}$), 1.88–1.94 (m, 2H, 2H, 2*-$\underline{H}$, 3*-$\underline{H}$), 2.10–2.17 (m, 1H, 3*-$\underline{H}$), 3.49 (dt, ^{2}J = 9.6 Hz, ^{3}J = 6.6 Hz, 1H, 1*-$\underline{H}$), 3.67 (dt, ^{2}J = 9.7 Hz, ^{3}J = 7.0 Hz, 1H, 1*-$\underline{H}$), 4.26 (dd, ^{3}J = 4.7 Hz, ^{3}J = 8.7 Hz, 1H, 4*-$\underline{H}$), 4.30 (dt, ^{3}J = 4.2 Hz, ^{3}J = 10.3 Hz, 1H, NHC$\underline{H}_{Leu}$), 5.35 (s, 1H, Ph$_2$C$\underline{H}$), 7.05 (dd, ^{3}J = 8.8 Hz, ^{4}J = 2.6 Hz, 1H, 6-$\underline{H}$), 7.23–7.28 (m, 3H, 2-$\underline{H}$, 4´-$\underline{H}$), 7.31–7.38 (m, 8H, 2´-$\underline{H}$, 3´-$\underline{H}$), 7.59 (d, ^{3}J = 8.8 Hz, 1H, 5-$\underline{H}$), 8.12 (d, ^{3}J = 7.9 Hz, 1H, OCON$\underline{H}$), 9.92 (s, 1H, N$\underline{H}$Ph), 12.34 (s, 1H, COO$\underline{H}$); ^{13}C NMR (150 MHz, DMSO-d_6) δ 21.54, 23.24, 24.24 ($\underline{C}$H($\underline{C}$H$_3$)$_{2,Leu}$), 24.74 ($\underline{C}$-2*), 28.66 ($\underline{C}$-3*), 40.22 (CH$\underline{C}$H$_{2,Leu}$), 46.51 ($\underline{C}$-1*), 51.15 (NH$\underline{C}$H$_{Leu}$), 56.56 (Ph$_2$$\underline{C}$HCO), 58.71 ($\underline{C}$-4*), 120.97 ($\underline{C}$-6), 122.77 ($\underline{C}$-2), 126.98 ($\underline{C}$-4´), 127.25 ($\underline{C}$-5), 127.46 ($\underline{C}$-3), 128.51 ($\underline{C}$-2´), 128.70 ($\underline{C}$-3´), 131.83 ($\underline{C}$-4), 139.99 ($\underline{C}$-1´), 148.52 ($\underline{C}$-1), 154.05 (O$\underline{C}$ONH), 170.28, 170.52 (CH$\underline{C}$O), 173.30 ($\underline{C}$OOH); LC/MS (ESI): H_2O/MeCN, 90:10 to 0:100; *m/z* 592.2 [M + H]$^+$; 100% purity; Q-TOF: HRMS (ESI): *m/z* [M + H]$^+$ calcd. for $C_{32}H_{34}ClN_3O_6$: 592.2209, found: 592.2184.

3-Chloro-4-(2-diphenylacetamido)phenoxycarbonyl-leucyl-phenylalanine *tert*-butyl ester

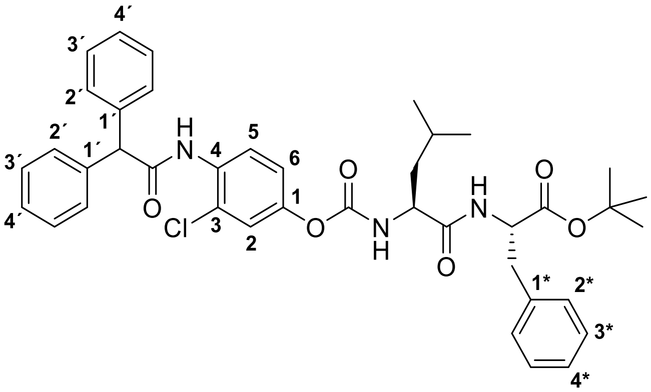

The synthesis was carried out according to GP13 using Cbz-Leu-Phe-O*t*Bu (0.35 g) for the hydrogenation and phenol **46** (169 mg) for the subsequent carbamate formation. Column chromatography on silica gel using CH_2Cl_2 / MeOH (29:1) as eluent afforded the product as a colorless solid (117 mg, 0.17 mmol, 34%). mp 164–167 °C; ^{1}H NMR (600 MHz, $CDCl_3$) δ 0.92 (2 × d, ^{3}J = 6.4 Hz, 6H, CH(C$\underline{H}_3$)$_{2,Leu}$), 1.39 (s, 9H, C(C$\underline{H}_3$)$_3$), 1.49–1.53 (m, 1H, CHC$\underline{H}_{2,Leu}$), 1.60–1.71 (m, 2H,

$CHC\underline{H}_{2,Leu}$, $C\underline{H}(CH_3)_{2,Leu}$), 3.04–3.13 (m, 2H, $CHC\underline{H}_{2,Phe}$), 4.15 (dt, ^3J = 5.6 Hz, ^3J = 8.7 Hz, 1H, $NHC\underline{H}_{Phe}$), 4.71 (q, ^3J = 6.1 Hz, 1H, $NHC\underline{H}_{Leu}$), 5.14 (s, 1H, $Ph_2C\underline{H}$), 5.40 (d, ^3J = 8.3 Hz, 1H, $OCON\underline{H}$), 6.27 (d, ^3J = 7.6 Hz, 1H, $N\underline{H}CH_{Phe}$), 7.01 (dd, ^3J = 9.0 Hz, ^4J = 2.4 Hz, 1H, 6-$\underline{H}$), 7.10–7.14 (m, 3H, 2*-$\underline{H}$, 4*-$\underline{H}$), 7.19–7.23 (m, 2H, 3*-$\underline{H}$), 7.27–7.32 (m, 6H, 2´-$\underline{H}$, 4´-$\underline{H}$), 7.34–7.38 (m, 4H, 3´-$\underline{H}$), 7.79 (s, 1H, $N\underline{H}Ph$), 8.44 (d, ^3J = 9.1 Hz, 1H, 5-$\underline{H}$), one signal (2-$\underline{H}$) is not visible; ^{13}C NMR (150 MHz, $CDCl_3$) δ 21.99, 22.89, 24.68 ($\underline{C}H(C\underline{H}_3)_{2,Leu}$), 27.95 ($C(\underline{C}H_3)_3$), 37.94 ($CH\underline{C}H_{2,Phe}$), 41.65 ($CH\underline{C}H_{2,Leu}$), 53.59, 53.68 ($NH\underline{C}H_{Phe}$, $NH\underline{C}H_{Leu}$), 60.48 ($Ph_2\underline{C}H$), 82.65 ($\underline{C}(CH_3)_3$), 120.84 ($\underline{C}$-6), 121.44 ($\underline{C}$-2), 122.30 ($\underline{C}$-5), 122.80 ($\underline{C}$-3), 127.07 ($\underline{C}$-4*), 127.68 ($\underline{C}$-4´), 128.43 ($\underline{C}$-2*), 129.02, 129.05 ($\underline{C}$-2´, $\underline{C}$-3´), 129.50 ($\underline{C}$-3*), 131.94 ($\underline{C}$-4), 135.83 ($\underline{C}$-1*), 138.66 ($\underline{C}$-1´), 146.72 ($\underline{C}$-1), 153.76 ($O\underline{C}ONH$), 170.15, 170.99 ($CH\underline{C}O$). Peak 170.15 contains two signals; LC/MS (ESI): H_2O/MeCN, 90:10 to 0:100; *m/z* 715.7 $[M + NH_4]^+$; 99% purity; Q-TOF: HRMS (ESI): *m/z* $[M + NH_4]^+$ calcd. for $C_{40}H_{44}ClN_3O_6$: 715.3257, found: 715.3247.

3-Chloro-4-(2-diphenylacetamido)phenoxycarbonyl-leucyl-phenylalanine (29)

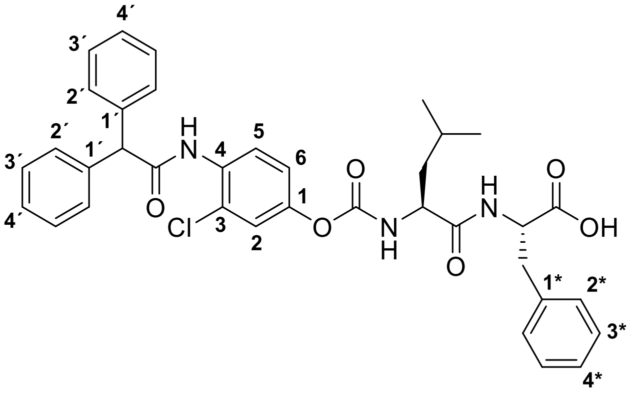

The synthesis was carried out according to GP8 using the corresponding *tert*-butyl ester (70 mg, 0.10 mmol). Column chromatography on silica gel afforded the desired compound as a colorless solid (53 mg, 0.08 mmol, 83%). mp 187–192 °C; ^1H NMR (600 MHz, DMSO-d_6) δ 0.86 (2 × d, ^3J = 6.7 Hz, 6H, $CH(C\underline{H}_3)_{2,Leu}$), 1.36–1.49 (m, 2H, $CHC\underline{H}_{2,Leu}$), 1.58–1.66 (m, 1H, $C\underline{H}(CH_3)_{2,Leu}$), 2.91 (dd, ^2J = 13.9 Hz, ^3J = 8.9 Hz, 1H, $CHC\underline{H}_{2,Phe}$), 3.05 (dd, ^2J = 14.0 Hz, ^3J = 5.3 Hz, 1H, $CHC\underline{H}_{2,Phe}$), 4.05 (dt, ^3J = 5.7 Hz, ^3J = 9.4 Hz, 1H, $NHC\underline{H}_{Phe}$), 4.42 (q, ^3J = 8.2 Hz, 1H, $NHC\underline{H}_{Leu}$), 5.35 (s, 1H, $Ph_2C\underline{H}$), 7.04 (dd, ^4J = 2.6 Hz, ^3J = 8.8 Hz, 1H, 6-$\underline{H}$), 7.14–7.28 (m, 8H), 7.31–7.39 (m, 8H, 2-$\underline{H}$, 2´-$\underline{H}$, 3´-$\underline{H}$, 4´-$\underline{H}$, 2*-$\underline{H}$, 3*-$\underline{H}$, 4*-$\underline{H}$), 7.60 (d, ^3J = 8.8 Hz, 1H, 5-$\underline{H}$), 7.95 (d, ^3J = 8.5 Hz, 1H, $OCON\underline{H}$), 8.15 (d, ^3J = 7.9 Hz, 1H, $N\underline{H}CH_{Phe}$), 9.93 (s, 1H, $N\underline{H}Ph$), one signal ($COO\underline{H}$) is not visible; ^{13}C NMR (150 MHz, DMSO-d_6) δ 21.68, 23.10, 24.29 ($\underline{C}H(\underline{C}H_3)_{2,Leu}$), 36.76 ($CH\underline{C}H_{2,Phe}$), 40.68 ($CH\underline{C}H_{2,Leu}$), 53.44, 53.48 ($NH\underline{C}H_{Phe}$, $NH\underline{C}H_{Leu}$), 56.58 ($Ph_2\underline{C}H$), 120.96 ($\underline{C}$-6), 122.75 ($\underline{C}$-2), 126.50 ($\underline{C}$-5), 127.01 ($\underline{C}$-4´), 127.28, 127.49 ($\underline{C}$-3, $\underline{C}$-4*), 128.26 ($\underline{C}$-2*), 128.53, 128.73 ($\underline{C}$-2´, $\underline{C}$-3´), 129.31 ($\underline{C}$-3*), 131.82 ($\underline{C}$-4), 137.67 ($\underline{C}$-1*), 140.01 ($\underline{C}$-1´), 148.58 ($\underline{C}$-1), 153.76 ($O\underline{C}ONH$), 170.56, 171.84 ($CH\underline{C}O$), 172.89 ($\underline{C}OOH$); LC/MS (ESI): H_2O/MeCN, 90:10 to 0:100; *m/z* 642.3 $[M + H]^+$; 99% purity; Q-TOF: HRMS (ESI): *m/z* $[M + H]^+$ calcd. for $C_{36}H_{36}ClN_3O_6$: 642.2365, found: 642.2322.

3-Chlorophenoxycarbonyl-cyclohexylalanyl-phenylalanine *tert*-butyl ester

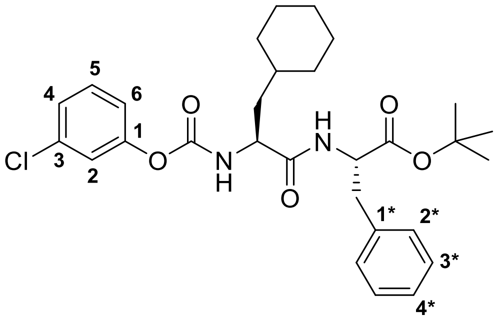

The synthesis was carried out according to GP13 using Cbz-Cha-Phe-O*t*Bu (0.38 g) for the hydrogenation and commercially available 3-chlorophenol (64 mg) for the subsequent carbamate formation. Column chromatography on silica gel using petrolum ether / EtOAc (4:1) as eluent afforded the product as a colorless solid (68 mg, 0.13 mmol, 26%). mp 128–131 °C; ^{1}H NMR (600 MHz, $CDCl_3$) δ 0.84–0.99 (m, 2H, CH(C$\underline{H}_2$)$_5$), 1.08–1.26 (m, 3H, C$\underline{H}$(C$\underline{H}_2$)$_5$), 1.39 (s, 9H, C(C$\underline{H}_3$)$_3$), 1.49–1.56 (m, 1H, CH(C$\underline{H}_2$)$_5$), 1.58–1.70 (m, 6H, CH(C$\underline{H}_2$)$_5$, NHCHC$\underline{H}_{2,Cha}$), 1.74–1.80 (m, 1H, NHCHC$\underline{H}_{2,Cha}$), 3.05–3.12 (m, 2H, CHC$\underline{H}_{2,Phe}$), 4.20 (dt, ^{3}J = 5.7 Hz, ^{3}J = 8.7 Hz, 1H, NHC$\underline{H}_{Phe}$), 4.72 (q, ^{3}J = 6.1 Hz, 1H, NHC$\underline{H}_{Cha}$), 5.49 (d, ^{3}J = 8.3 Hz, 1H, OCON$\underline{H}$), 6.34 (d, ^{3}J = 7.6 Hz, 1H, N$\underline{H}$CH$_{Phe}$), 7.01 (d, ^{3}J = 8.1 Hz, 1H, 6-$\underline{H}$), 7.12–7.28 (m, 8H, 2-$\underline{H}$, 4-$\underline{H}$, 5-$\underline{H}$, 2*-$\underline{H}$, 3*-$\underline{H}$, 4*-$\underline{H}$); ^{13}C NMR (150 MHz, $CDCl_3$) δ 25.98, 26.11, 26.30 (CH($\underline{C}$H$_2$)$_5$), 27.92 (C($\underline{C}$H$_3$)$_3$), 32.62, 33.56, 33.98 ($\underline{C}$H($\underline{C}$H$_2$)$_5$), 37.99 (CH$\underline{C}$H$_{2,Phe}$), 40.19 (NHCH$\underline{C}$H$_{2,Cha}$), 53.03, 53.60 (NH$\underline{C}$H$_{Phe}$, NH$\underline{C}$H$_{Cha}$), 82.61 ($\underline{C}$(CH$_3$)$_3$), 119.83 ($\underline{C}$-6), 122.15 ($\underline{C}$-2), 125.66 ($\underline{C}$-4), 127.03 ($\underline{C}$-4*), 128.41, 129.51 ($\underline{C}$-2*, $\underline{C}$-3*), 129.95 ($\underline{C}$-5), 134.47 ($\underline{C}$-3), 135.85 ($\underline{C}$-1*), 151.34 ($\underline{C}$-1), 153.68 (O$\underline{C}$ONH), 170.18, 171.18 (CH$\underline{C}$O); LC/MS (ESI): H_2O/MeCN, 90:10 to 0:100; *m/z* 529.2 [M + H]$^{+}$; 92% purity; Q-TOF: HRMS (ESI): *m/z* [M + H]$^{+}$ calcd. for $C_{29}H_{37}ClN_2O_5$: 529.2464, found: 529.2467.

3-Chlorophenoxycarbonyl-cyclohexylalanyl-phenylalanine

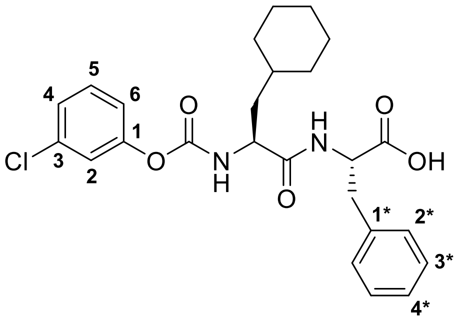

The synthesis was carried out according to GP8 using the corresponding *tert*-butyl ester (63 mg, 0.12 mmol). Column chromatography on silica gel afforded the desired compound as a colorless solid (39 mg, 0.08 mmol, 69%). mp 88–91 °C; ^{1}H NMR (600 MHz, $CDCl_3$) δ 0.80-0.97 (m, 3H, C$\underline{H}$(C$\underline{H}_2$)$_5$), 1.08–1.21 (m, 3H, CH(C$\underline{H}_2$)$_5$), 1.30–1.38 (m, 1H, CH(C$\underline{H}_2$)$_5$), 1.47–1.54 (m, 1H, CH(C$\underline{H}_2$)$_5$), 1.58–1.68 (m, 4H, CH(C$\underline{H}_2$)$_5$, NHCHC$\underline{H}_{2,Cha}$), 1.69–1.76 (m, 1H, NHCHC$\underline{H}_{2,Cha}$), 3.04 (dd, ^{2}J = 14.1 Hz, ^{3}J = 6.5 Hz, 1H, CHC$\underline{H}_{2,Phe}$), 3.21 (dd, ^{2}J = 14.1 Hz, ^{3}J = 5.5 Hz, 1H, CHC$\underline{H}_{2,Phe}$), 4.20–4.28 (m, 1H, NHC$\underline{H}_{Phe}$),

4.84 (q, ^3J = 6.5 Hz, 1H, NHCH$_{Cha}$), 5.69 (d, ^3J = 8.5 Hz, 1H, OCONH), 6.57 (d, ^3J = 7.7 Hz, 1H, NHCH$_{Phe}$), 6.97–7.01 (m, 1H, 6-H), 7.07–7.16 (m, 3H), 7.16–7.30 (m, 5H, 4-H, 5-H, 2*-H, 3*-H, 4*-H), one signal (COOH) is not visible; ^{13}C NMR (150 MHz, $CDCl_3$) δ 25.96, 26.08, 26.28 (CH(CH_2)$_5$), 32.64, 33.46, 33.91 (CH(CH_2)$_5$), 37.33 (CHCH$_{2,Phe}$), 39.80 (NHCHCH$_{2,Cha}$), 52.93, 53.15 (NHCH$_{Phe}$, NHCH$_{Cha}$), 119.83 (C-6), 122.13 (C-2), 125.82 (C-4), 127.29 (C-4*), 128.66, 129.36 (C-2*, C-3*), 130.03 (C-5), 134.53 (C-3), 135.43 (C-1*), 151.24 (C-1), 153.93 (OCONH), 172.06, 173.88 (CHCO); LC/MS (ESI): H_2O/MeCN, 90:10 to 0:100; *m/z* 473.0 [M + H]$^+$; 96% purity; Q-TOF: HRMS (ESI): *m/z* [M + H]$^+$ calcd. for $C_{25}H_{29}ClN_2O_5$: 473.1838, found: 473.1760.

3-Chloro-4-(2-diphenylacetamido)phenoxycarbonyl-cyclohexylalanyl-phenylalanine *tert*-butyl ester

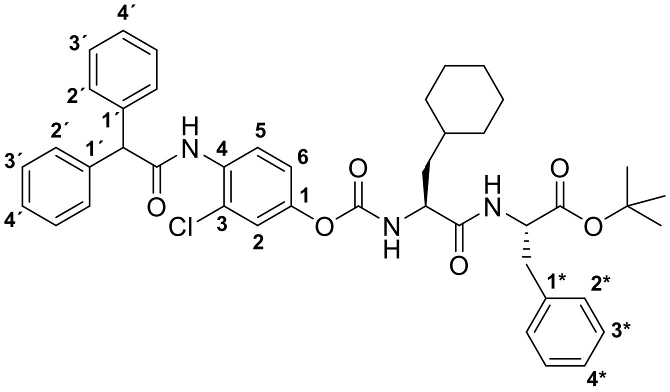

The synthesis was carried out according to GP13 using Cbz-Cha-Phe-O*t*Bu (0.38 g) for the hydrogenation and phenol **46** (169 mg) for the subsequent carbamate formation. Column chromatography on silica gel using petrolum ether / EtOAc (3:1) as eluent afforded the product as a colorless solid (185 mg, 0.25 mmol, 50%). mp 134–139 °C; ^{1}H NMR (600 MHz, $CDCl_3$) δ 0.84–0.98 (m, 3H, CH(CH_2)$_5$), 1.07–1.19 (m, 3H, CH(CH_2)$_5$), 1.39 (s, 9H, C(CH_3)$_3$), 1.48–1.54 (m, 1H, CH(CH_2)$_5$), 1.58–1.70 (m, 5H, CH(CH_2)$_5$, NHCHCH$_{2,Cha}$), 1.72–1.78 (m, 1H, NHCHCH$_{2,Cha}$), 3.04–3.12 (m, 2H, CHCH$_{2,Phe}$), 4.18 (q, ^3J = 8.5 Hz, 1H, NHCH$_{Phe}$), 4.71 (q, ^3J = 6.3 Hz, 1H, NHCH$_{Cha}$), 5.14 (s, 1H, Ph_2CH), 5.42 (d, ^3J = 8.2 Hz, 1H, OCONH), 6.31 (d, ^3J = 7.6 Hz, 1H, NHCH$_{Phe}$), 7.02 (dd, ^3J = 8.9 Hz, ^4J = 1.6 Hz, 1H, 6-H), 7.11–7.14 (m, 2H), 7.19–7.26 (m, 4H, 2-H, 2*-H, 3*-H, 4*-H), 7.27–7.33 (m, 6H, 2´-H, 4´-H), 7.36 (t, ^3J = 7.5 Hz, 4H, 3´-H), 7.80 (s, 1H, NHPh), 8.43 (d, ^3J = 9.1 Hz, 1H, 5-H); ^{13}C NMR (150 MHz, $CDCl_3$) δ 26.00, 26.14, 26.32 (CH(CH_2)$_5$), 27.97 (C(CH_3)$_3$), 32.65, 33.59, 34.01 (CH(CH_2)$_5$), 38.00 (CHCH$_{2,Phe}$), 40.19 (NHCHCH$_{2,Cha}$), 53.09 (NHCH$_{Phe}$), 53.63 (NHCH$_{Cha}$), 60.49 (Ph_2CH), 82.68 (C(CH_3)$_3$), 120.86 (C-6), 121.48 (C-2), 122.33 (C-5), 122.85 (C-3), 127.08 (C-4*), 127.72 (C-4´), 128.45 (C-2*), 129.04, 129.08 (C-2´, C-3´), 129.54 (C-3*), 131.94 (C-4), 135.85 (C-1*), 138.67 (C-1´), 146.77 (C-1), 153.79 (OCONH), 170.18, 170.22, 171.25 (CHCO); LC/MS (ESI): H_2O/MeCN, 90:10 to 0:100; *m/z* 738.1 [M + H]$^+$; 98% purity; Q-TOF: HRMS (ESI): *m/z* [M + H]$^+$ calcd. for $C_{43}H_{48}ClN_3O_6$: 738.3304, found: 738.3261.

3-Chloro-4-(2-diphenylacetamido)phenoxycarbonyl-cyclohexylalanyl-phenylalanine (30)

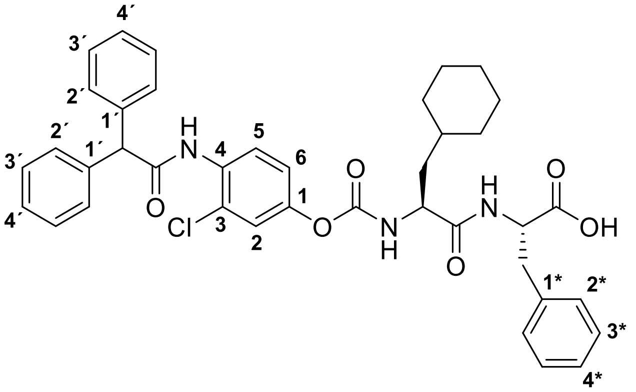

The synthesis was carried out according to GP8 using the corresponding *tert*-butyl ester (74 mg, 0.10 mmol). Column chromatography on silica gel afforded the desired compound as a colorless solid (52 mg, 0.08 mmol, 76%). mp 160–164 °C; ^{1}H NMR (600 MHz, DMSO-d_6) δ 0.79–0.87 (m, 3H, C<u>H</u>(C<u>H</u>$_2$)$_5$), 1.08–1.16 (m, 3H, CH(C<u>H</u>$_2$)$_5$), 1.40–1.46 (m, 2H, CH(C<u>H</u>$_2$)$_5$), 1.55–1.73 (m, 5H, CH(C<u>H</u>$_2$)$_5$, NHCHC<u>H</u>$_{2,Cha}$), 2.91 (dd, ^2J = 13.5 Hz, ^3J = 8.5 Hz, 1H, CHC<u>H</u>$_{2,Phe}$), 3.07 (dd, ^2J = 13.6 Hz, ^3J = 4.1 Hz, 1H, CHC<u>H</u>$_{2,Phe}$), 4.04 (q, ^3J = 7.0 Hz, 1H, NHC<u>H</u>$_{Phe}$), 4.33–4.43 (m, 1H, NHC<u>H</u>$_{Cha}$), 5.36 (s, 1H, Ph$_2$C<u>H</u>), 7.04 (dd, ^3J = 8.8 Hz, ^4J = 2.6 Hz, 1H, 6-<u>H</u>), 7.13–7.28 (m, 8H), 7.30–7.41 (m, 9H, 2-<u>H</u>, 2*-<u>H</u>, 3*-<u>H</u>, 4*-<u>H</u>, 2´-<u>H</u>, 3´-<u>H</u>, 4´-<u>H</u>, N<u>H</u>Ph or N<u>H</u>CH$_{Phe}$), 7.60 (d, ^3J = 8.8 Hz, 1H, 5-<u>H</u>), 7.98 (d, ^3J = 8.4 Hz, 1H, OCON<u>H</u>), 9.93 (s, 1H, COO<u>H</u>), one signal (N<u>H</u>Ph or N<u>H</u>CH$_{Phe}$) is not visible; ^{13}C NMR (150 MHz, CDCl$_3$) δ 25.85, 25.97, 26.21 (CH(<u>C</u>H$_2$)$_5$), 32.36, 33.29, 33.74 (<u>C</u>H(<u>C</u>H$_2$)$_5$), 38.68 (CH<u>C</u>H$_{2,Phe}$), 39.51 (NHCH<u>C</u>H$_{2,Cha}$), 53.27 (NH<u>C</u>H$_{Phe}$), 54.86 (NH<u>C</u>H$_{Cha}$), 60.37 (Ph$_2$<u>C</u>H), 120.87 (<u>C</u>-6), 121.84 (<u>C</u>-2), 122.01 (<u>C</u>-5), 123.20 (<u>C</u>-3), 127.06 (<u>C</u>-4*), 127.73, 127.75 (<u>C</u>-4´), 128.51 (<u>C</u>-2*), 128.95, 128.96, 129.06 (<u>C</u>-2´, <u>C</u>-3´), 129.25 (<u>C</u>-3*), 131.45 (<u>C</u>-4), 135.56 (<u>C</u>-1*), 138.44, 138.50 (<u>C</u>-1´), 146.93 (<u>C</u>-1), 153.88 (O<u>C</u>ONH), 170.57 (CH<u>C</u>O), 175.56 (<u>C</u>OOH), one signal (CHCO) is not visible; LC/MS (ESI): H_2O/MeCN, 90:10 to 0:100; *m/z* 682.5 [M + H]$^+$; 96% purity; Q-TOF: HRMS (ESI): *m/z* [M + H]$^+$ calcd. for $C_{39}H_{40}ClN_3O_6$: 682.2678, found: 682.2673.

3-Chloro-4-(2-dibenzylacetamido)phenoxycarbonyl-cyclohexylalanyl-phenylalanine *tert*-butyl ester

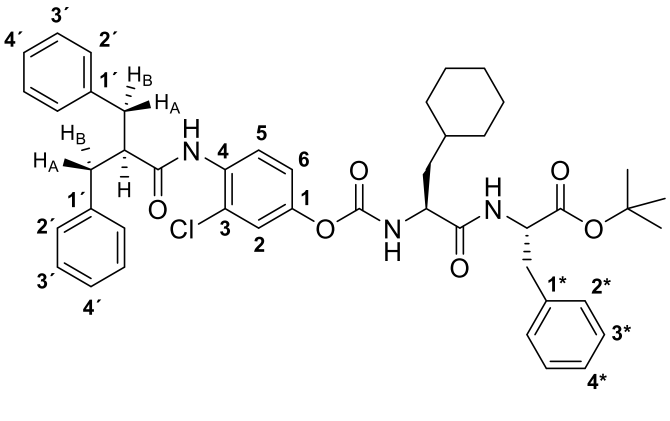

The synthesis was carried out according to GP13 using Cbz-Cha-Phe-O*t*Bu (0.38 g) for the hydrogenation and phenol **53** (183 mg) for the subsequent carbamate formation. Column chromatography on silica gel using petrolum ether / EtOAc (3:1) as eluent afforded the product as a colorless solid (163 mg, 0.21 mmol, 43%). mp 145–148 °C; ^{1}H NMR (600 MHz, $CDCl_3$) δ 0.91–0.98 (m, 2H, $CH(C\underline{H}_2)_5$), 1.08–1.18 (m, 3H, $C\underline{H}(C\underline{H}_2)_5$), 1.39 (s, 9H, $C(C\underline{H}_3)_3$), 1.48–1.54 (m, 1H, $CH(C\underline{H}_2)_5$), 1.58–1.70 (m, 6H), 1.72–1.78 (m, 1H, $CH(C\underline{H}_2)_5$, $NHCHC\underline{H}_{2,Cha}$), 2.71–2.77 (m, 1H, $C\underline{H}(CH_AH_B)_2$), 2.88 (dd, ^2J = 13.6 Hz, ^3J = 5.3 Hz, 2H, $CH(C\underline{H}_AH_B)_2$), 3.04–3.10 (m, 4H, $CH(CH_A\underline{H}_B)_2$, $CHC\underline{H}_{2Phe}$), 4.17 (dt, ^3J = 5.9 Hz, ^3J = 8.6 Hz, 1H, $NHC\underline{H}_{Phe}$), 4.70 (q, ^3J = 6.1 Hz, 1H, $NHC\underline{H}_{Cha}$), 5.45 (d, ^3J = 8.2 Hz, 1H, OCON$\underline{H}$), 6.31 (d, ^3J = 7.6 Hz, 1H, $N\underline{H}CH_{Phe}$), 6.89 (s, 1H, N$\underline{H}$Ph), 6.94 (dd, ^3J = 8.9 Hz, ^4J = 1.2 Hz, 1H, 6-$\underline{H}$), 7.03 (d, ^4J = 2.0 Hz, 1H, 2-$\underline{H}$), 7.12 (d, ^3J = 7.0 Hz, 2H, 2*-$\underline{H}$), 7.16–7.19 (m, 6H), 7.22–7.25 (m, 7H, 2´-$\underline{H}$, 3´-$\underline{H}$, 4´-$\underline{H}$, 3*-$\underline{H}$, 4*-$\underline{H}$), 8.14 (d, ^3J = 8.9 Hz, 1H, 5-$\underline{H}$); ^{13}C NMR (150 MHz, $CDCl_3$) δ 25.97, 26.10, 26.29 ($CH(\underline{C}H_2)_5$), 27.93 ($C(\underline{C}H_3)_3$), 32.61, 33.55, 33.96 ($\underline{C}H(\underline{C}H_2)_5$), 37.98 ($CH\underline{C}H_{2,Phe}$), 39.01 ($CH(\underline{C}H_AH_B)_2$), 40.18 ($NHCH\underline{C}H_{2,Cha}$), 53.02, 53.59, 53.75 ($NH\underline{C}H_{Phe}$, $NH\underline{C}H_{Cha}$, $\underline{C}H(CH_AH_B)_2$), 82.62 ($\underline{C}(CH_3)_3$), 120.57 ($\underline{C}$-6), 122.08 ($\underline{C}$-2), 122.20 ($\underline{C}$-5), 122.94 ($\underline{C}$-3), 126.61 ($\underline{C}$-4´), 127.04 ($\underline{C}$-4*), 128.41 ($\underline{C}$-2*), 128.65, 128.80 ($\underline{C}$-2´, $\underline{C}$-3´), 129.50 ($\underline{C}$-3*), 131.53 ($\underline{C}$-4), 135.83 ($\underline{C}$-1*), 139.10 ($\underline{C}$-1´), 146.59 ($\underline{C}$-1), 153.79 (O$\underline{C}$ONH), 170.19, 171.19, 172.47 (CH$\underline{C}$O); LC/MS (ESI): H_2O/MeCN, 90:10 to 0:100; *m/z* 783.4 $[M + NH_4]^+$; 99% purity; Q-TOF: HRMS (ESI): *m/z* $[M + NH_4]^+$ calcd. for $C_{45}H_{52}ClN_3O_6$: 783.3883, found: 783.3913.

3-Chloro-4-(2-dibenzylacetamido)phenoxycarbonyl-cyclohexylalanyl-phenylalanine (31)

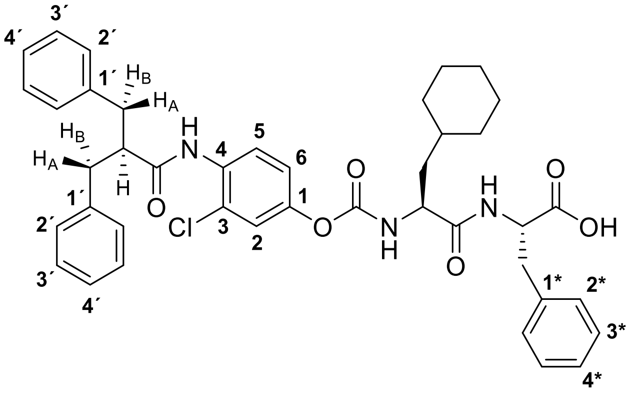

The synthesis was carried out according to GP8 using the corresponding *tert*-butyl ester (77 mg, 0.10 mmol). Column chromatography on silica gel afforded the desired compound as a colorless solid (68 mg, 0.10 mmol, 96%). mp 176–178 °C; ^{1}H NMR (600 MHz, $CDCl_3$) δ 0.90–0.95 (m, 2H, $CH(C\underline{H}_2)_5$), 1.08–1.16 (m, 3H, $C\underline{H}(C\underline{H}_2)_5$), 1.59–1.73 (m, 8H, $CH(C\underline{H}_2)_5$, $NHCHC\underline{H}_{2,Cha}$), 2.75–2.81 (m, 1H, $C\underline{H}(CH_AH_B)_2$), 2.87–2.93 (m, 2H, $CH(C\underline{H}_AH_B)_2$), 3.03–3.09 (m, 3H, $CH(CH_A\underline{H}_B)_2$, $CHC\underline{H}_{2Phe}$), 3.19 (d, ^2J = 14.0 Hz, ^3J = 5.4 Hz, $CHC\underline{H}_{2Phe}$), 4.24 (dt, ^3J = 6.0 Hz, ^3J = 8.8 Hz, 1H, $NHC\underline{H}_{Phe}$), 4.88 (q, ^3J = 6.4 Hz, 1H, $NHC\underline{H}_{Cha}$), 5.84 (d, ^3J = 8.3 Hz, 1H, OCON$\underline{H}$), 6.65 (d, ^3J = 7.9 Hz, 1H, $N\underline{H}CH_{Phe}$), 6.80 (dd, ^3J = 9.0 Hz, ^4J = 2.5 Hz, 1H, 6-$\underline{H}$), 6.90 (d, ^4J = 2.2 Hz, 1H, 2-$\underline{H}$), 6.93 (s, 1H, N$\underline{H}$Ph), 7.14 (d, ^3J = 7.0 Hz, 2H, 2*-$\underline{H}$), 7.18–7.20 (m, 6H), 7.22–7.27 (m, 7H, 2´-$\underline{H}$, 3´-$\underline{H}$, 4´-$\underline{H}$, 3*-$\underline{H}$, 4*-$\underline{H}$), 7.93

(d, ^3J = 9.0 Hz, 1H, 5-H), one signal (COOH) is not visible; ^{13}C NMR (150 MHz, $CDCl_3$) δ 25.96, 26.06, 26.30 (CH(CH_2)$_5$), 32.57, 33.47, 33.88 (CH(CH_2)$_5$), 37.49 (CH$CH_{2,Phe}$), 39.01 (CH(CH_AH_B)$_2$), 40.81 (NHCH$CH_{2,Cha}$), 53.01, 53.12, 53.63 (NHCH_{Phe}, NHCH_{Cha}, CH(CH_AH_B)$_2$), 120.58 (C-6), 121.90 (C-2), 122.82 (C-5), 123.60 (C-3), 126.69 (C-4´), 127.21 (C-4*), 128.60 (C-2*), 128.69, 128.70, 128.80, 128.82 (C-2´, C-3´), 129.41 (C-3*), 131.13 (C-4), 135.54 (C-1*), 138.97 (C-1´), 146.83 (C-1), 153.87 (OCONH), 172.04, 172.98 (CHCO), 174.54 (COOH); LC/MS (ESI): H_2O/MeCN, 90:10 to 0:100; *m/z* 710.6 $[M + H]^+$; 98% purity; Q-TOF: HRMS (ESI): *m/z* $[M + H]^+$ calcd. for $C_{41}H_{44}ClN_3O_6$: 710.2991, found: 710.2967.

8. NMR Spectra of Selected Compounds (1-31)

Compound **1**

^{1}H NMR

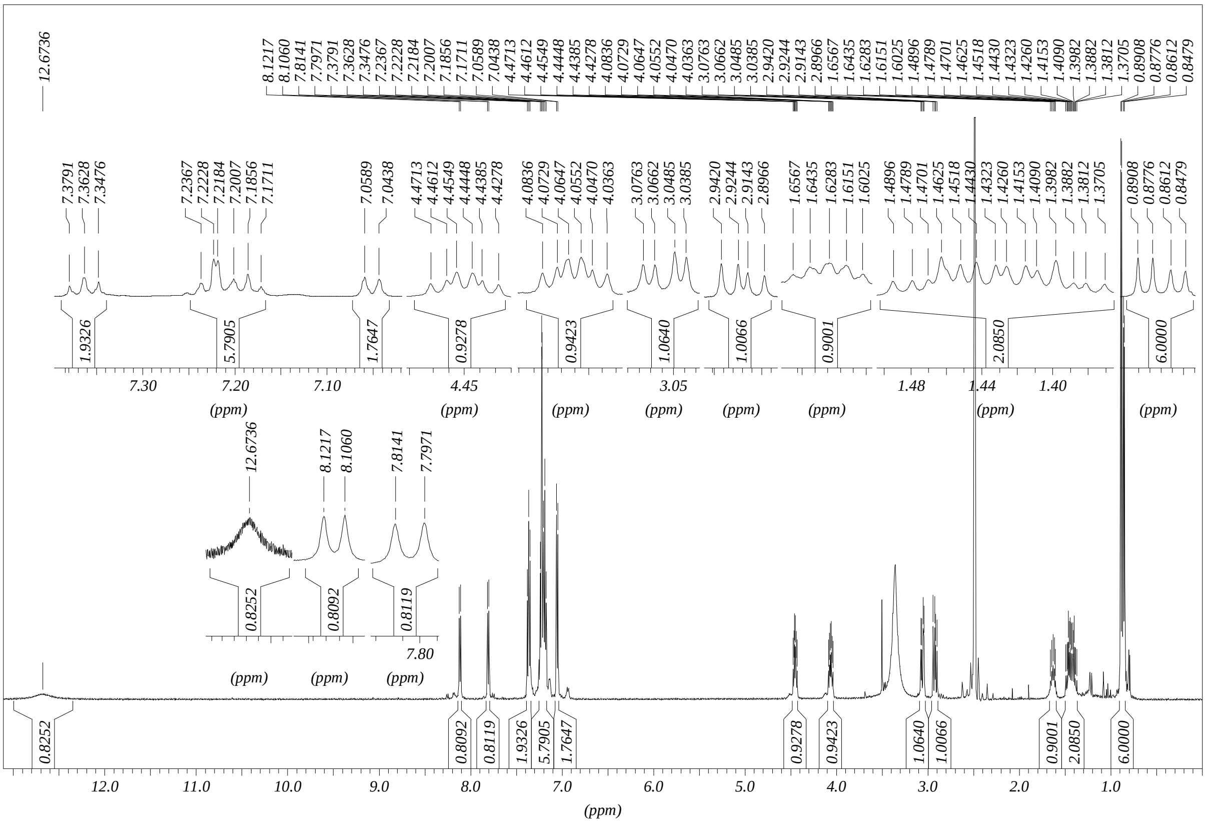

^{13}C NMR

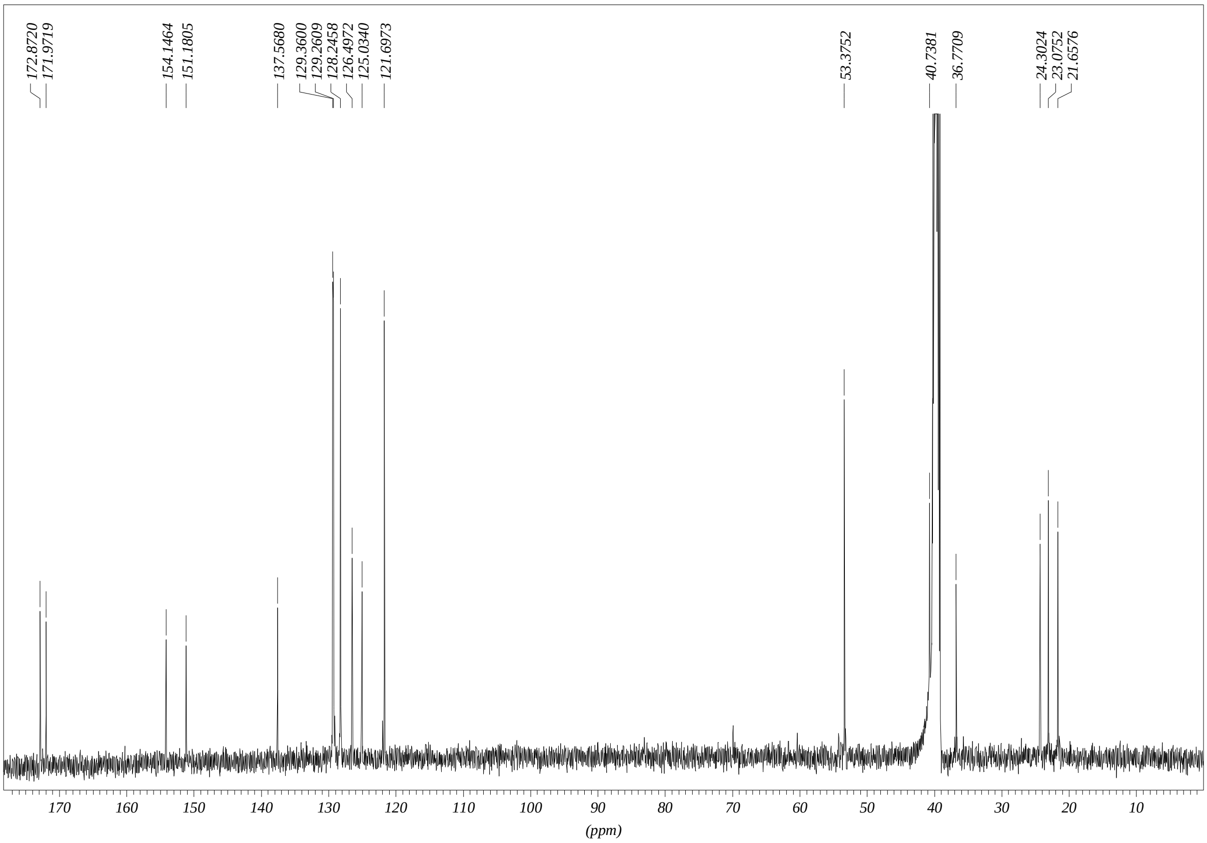

Compound **2**

^{1}H NMR

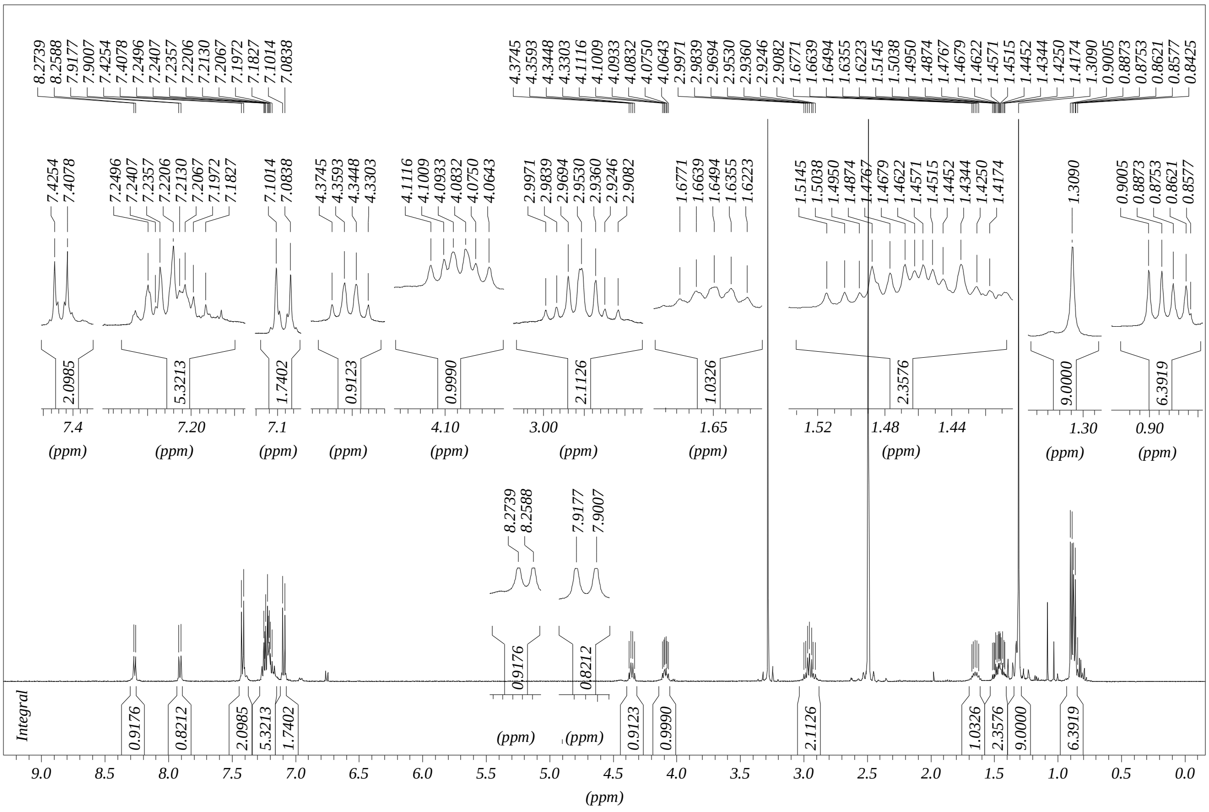

^{13}C NMR

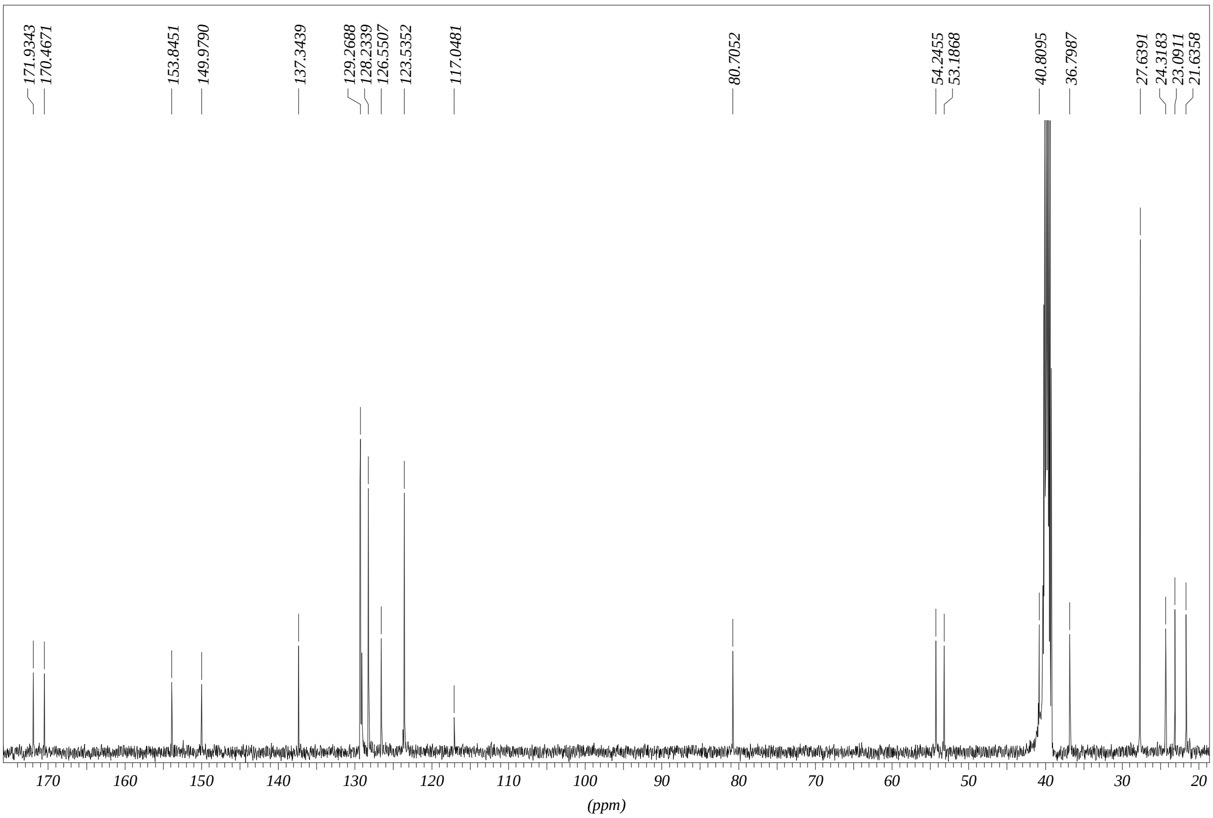

Compound **3**

^{1}H NMR

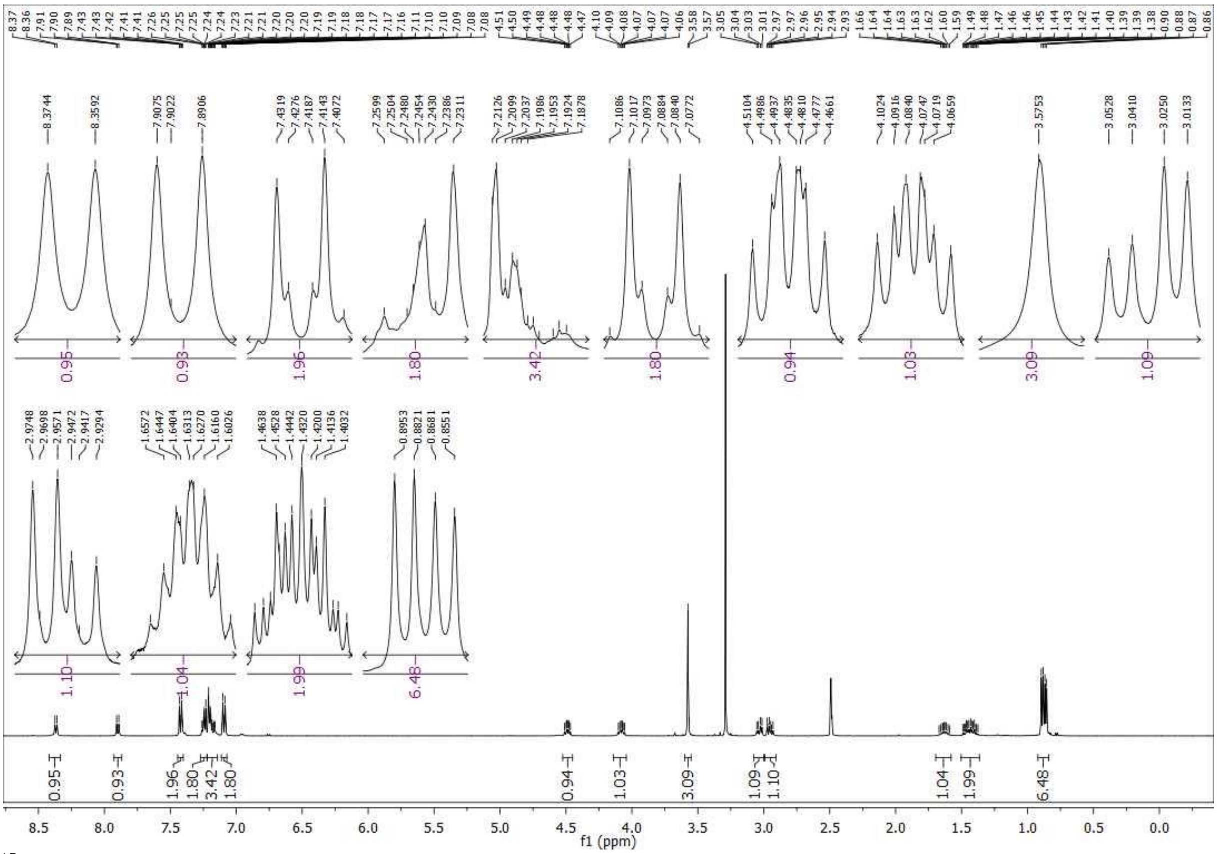

^{13}C NMR

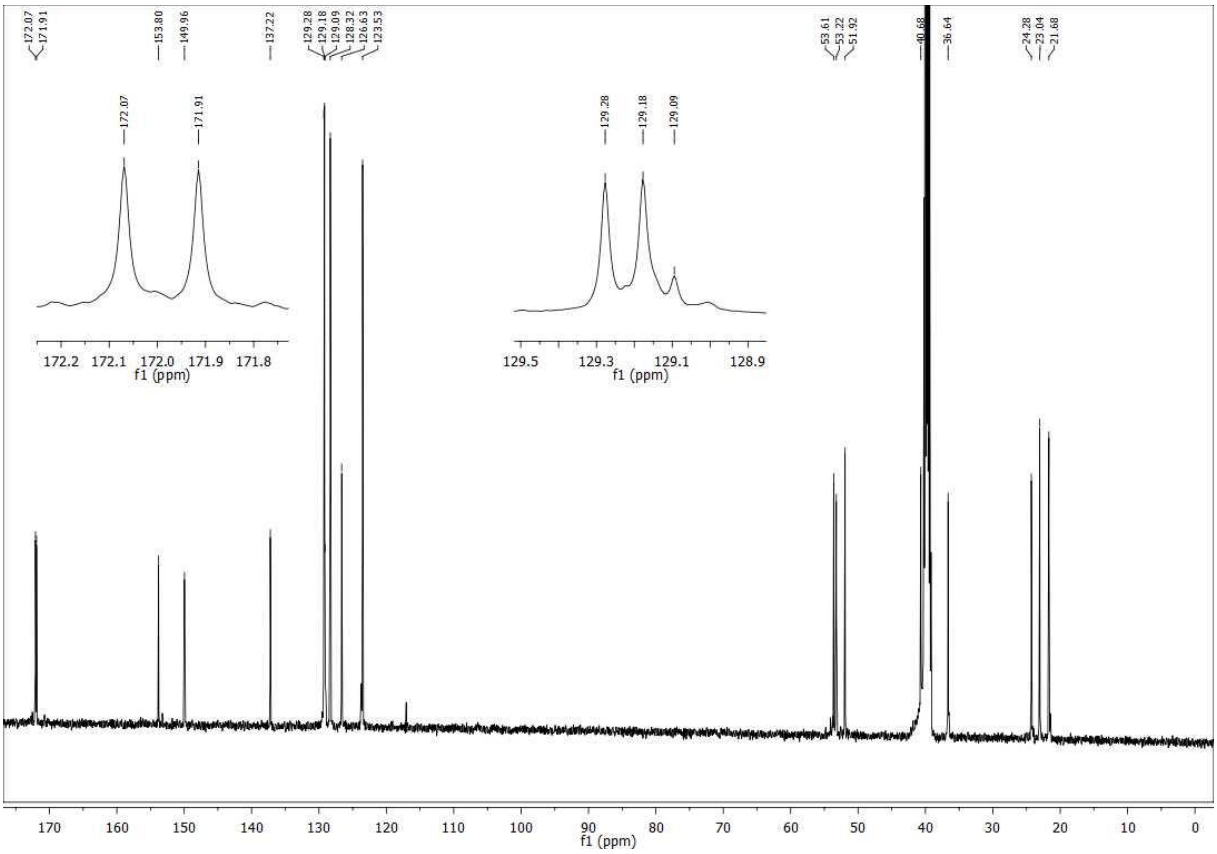

Compound **4**

^{1}H NMR

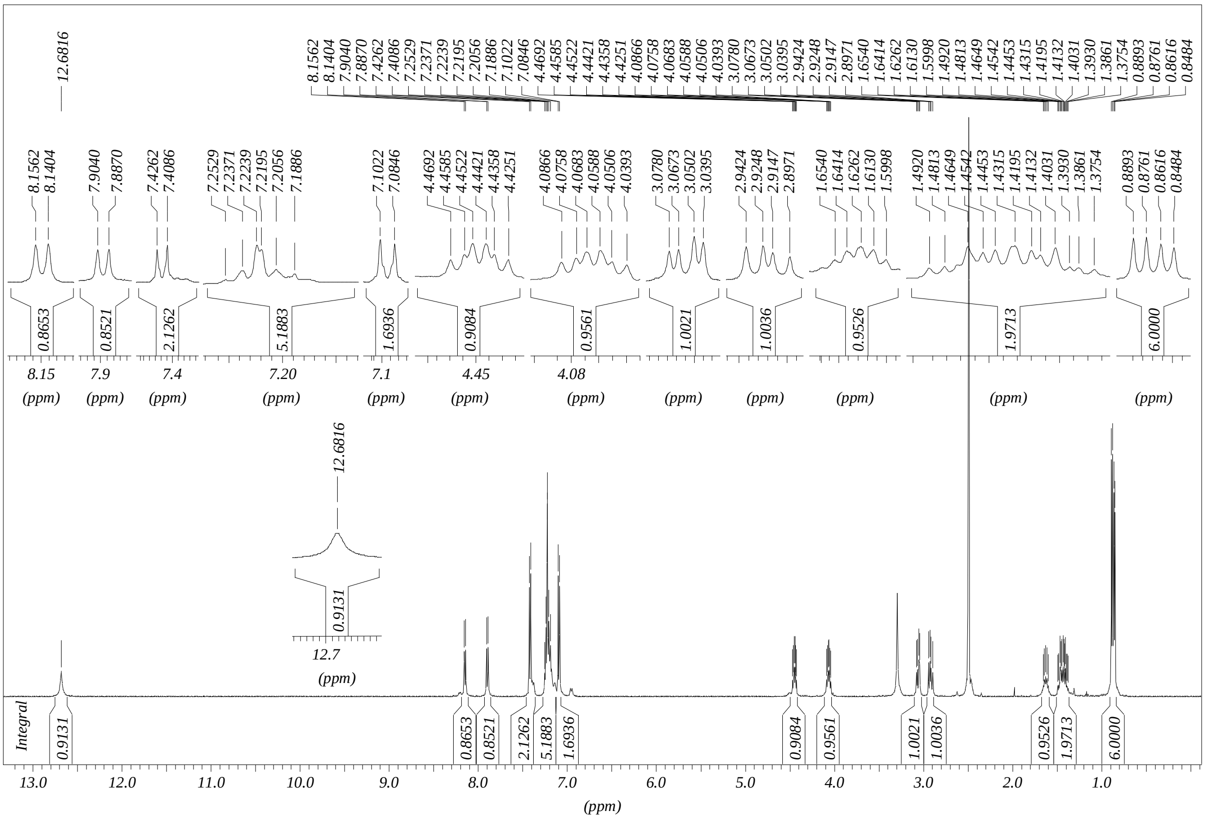

^{13}C NMR

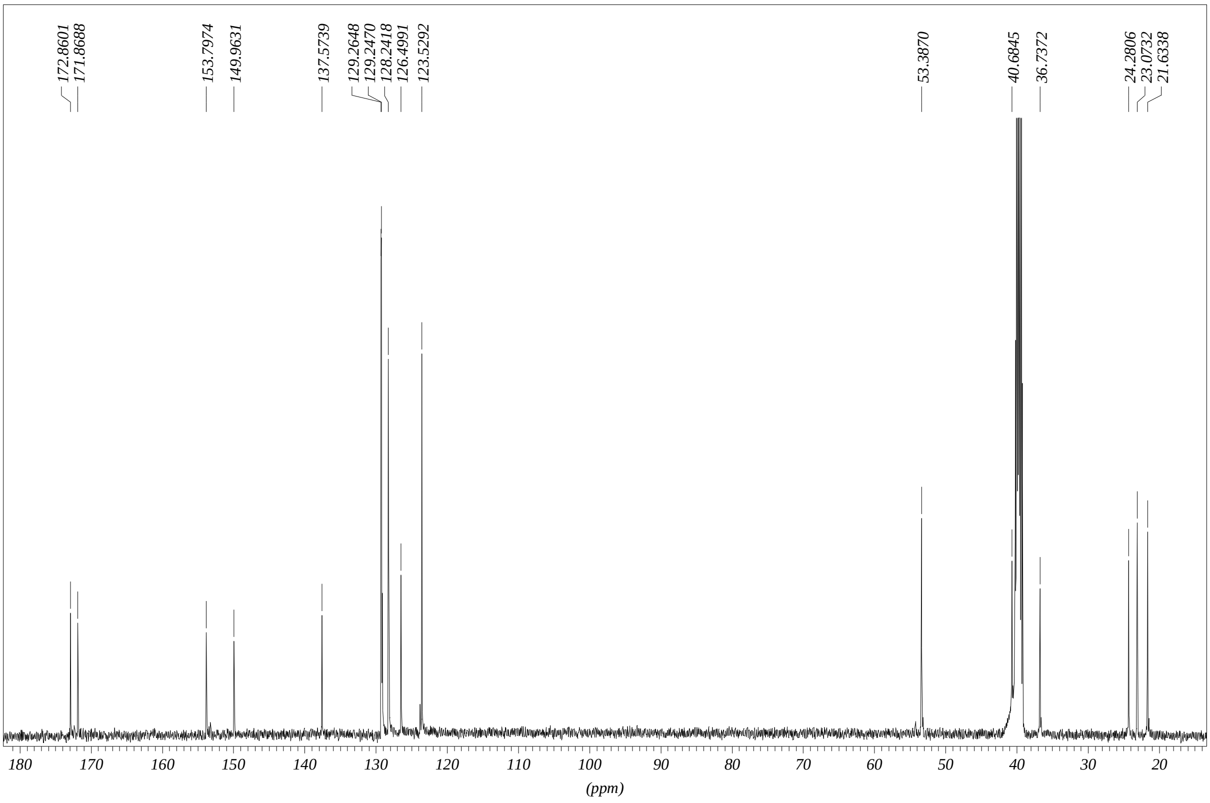

Compound **5**

^{1}H NMR

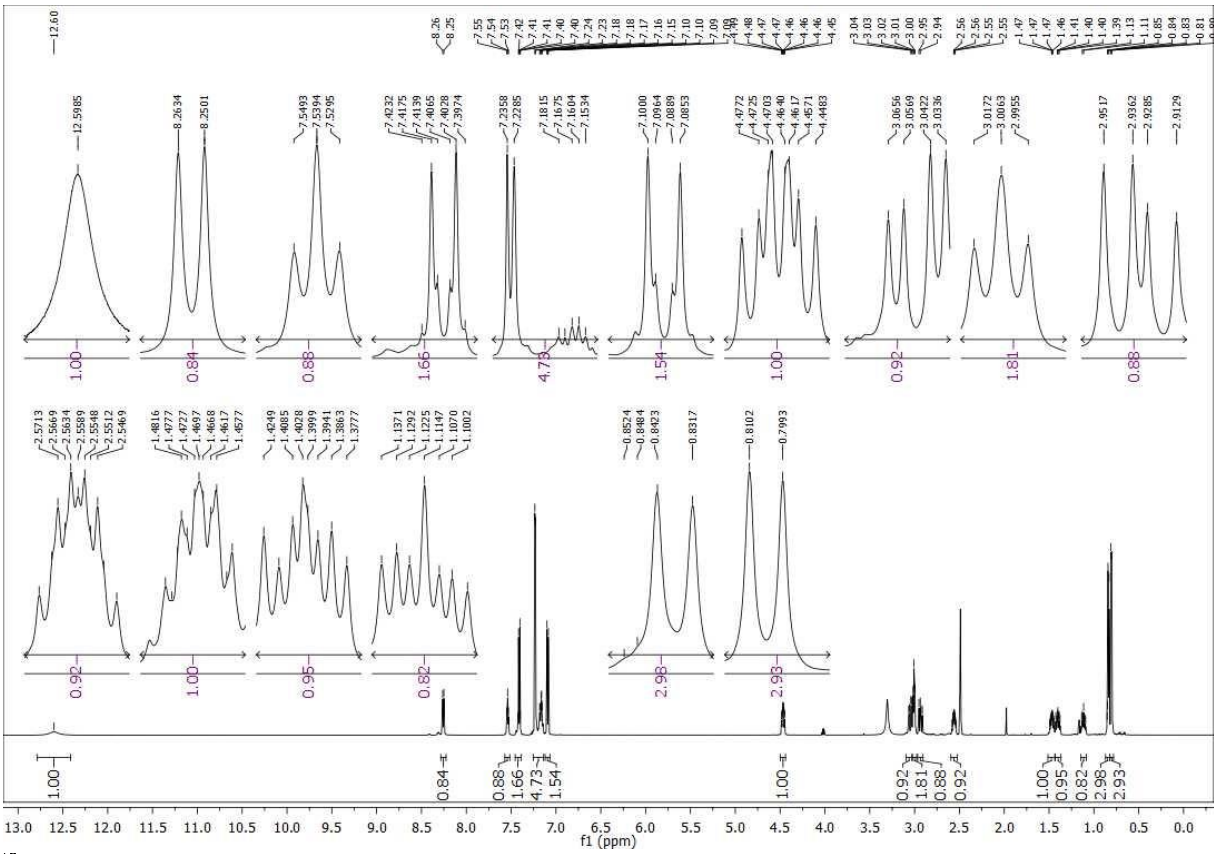

^{13}C NMR

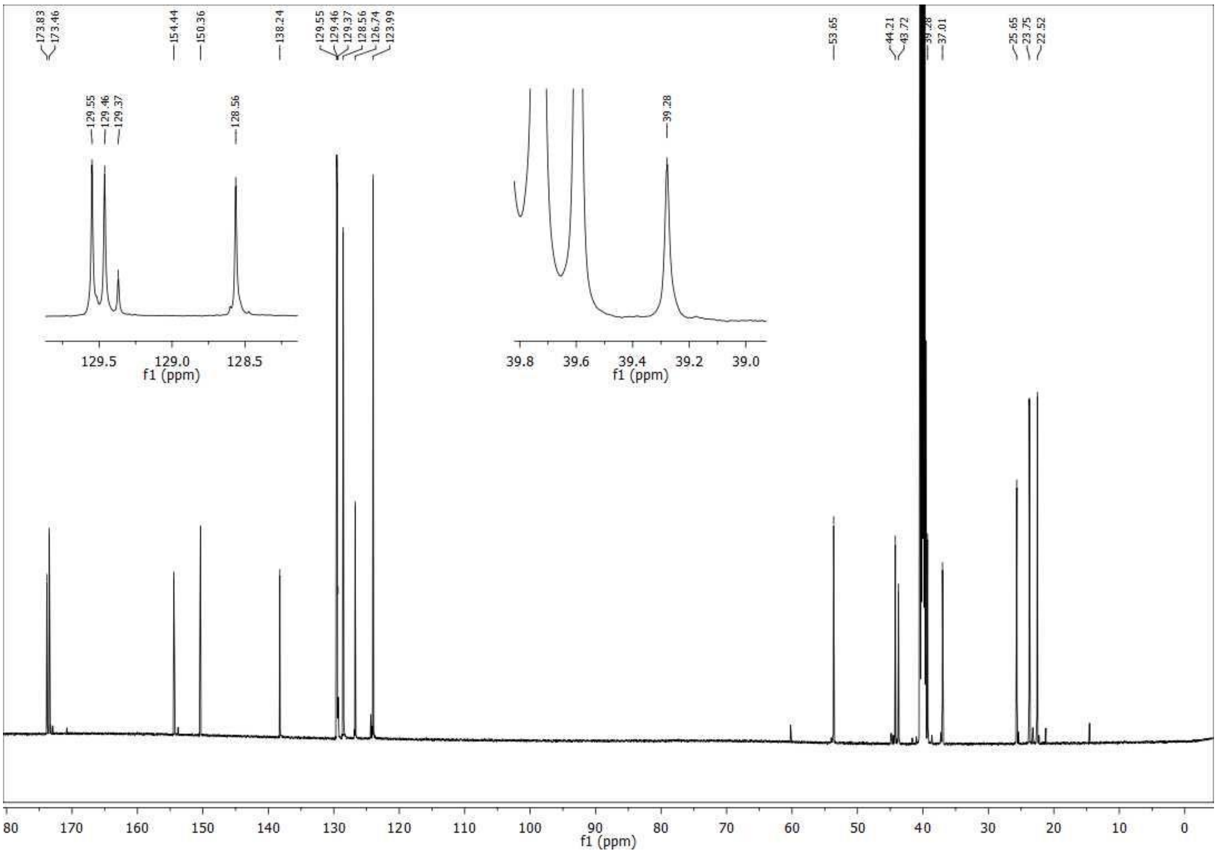

Compound **6**

^{1}H NMR

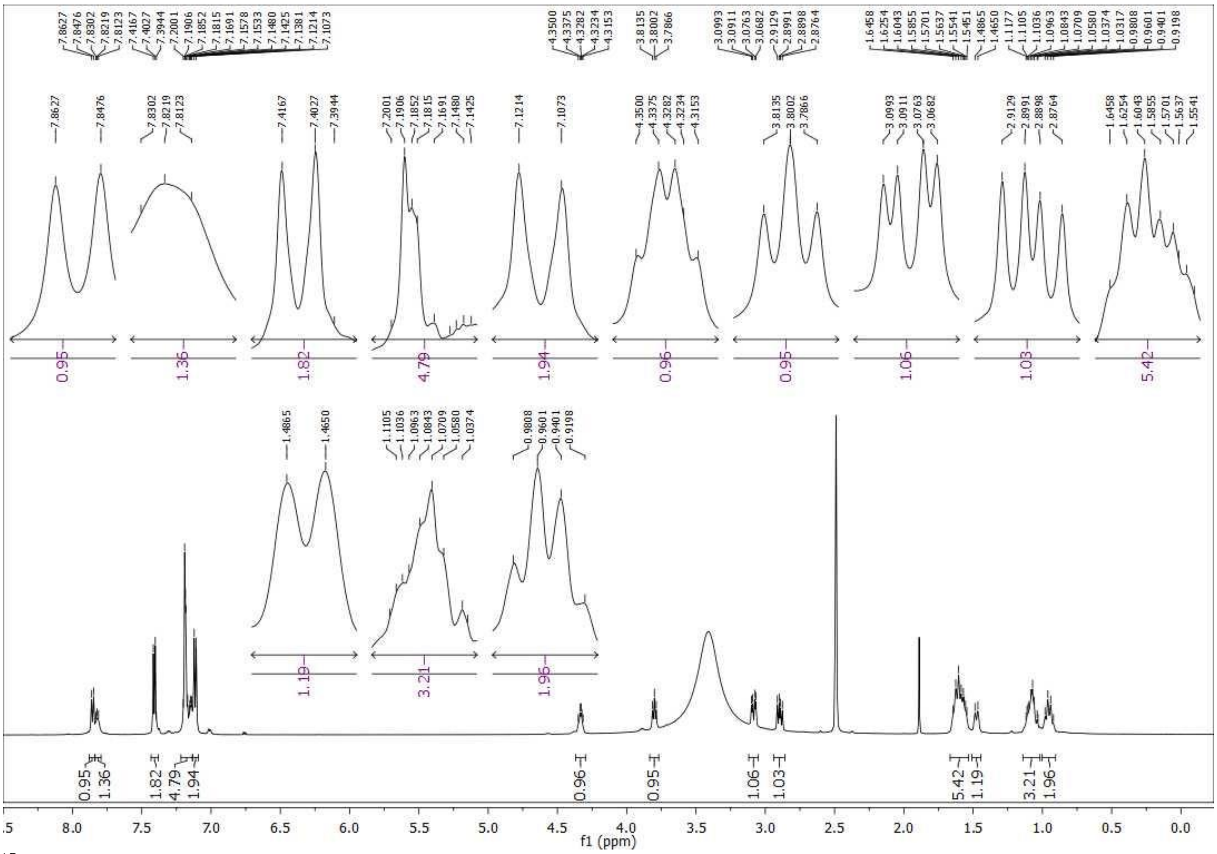

^{13}C NMR

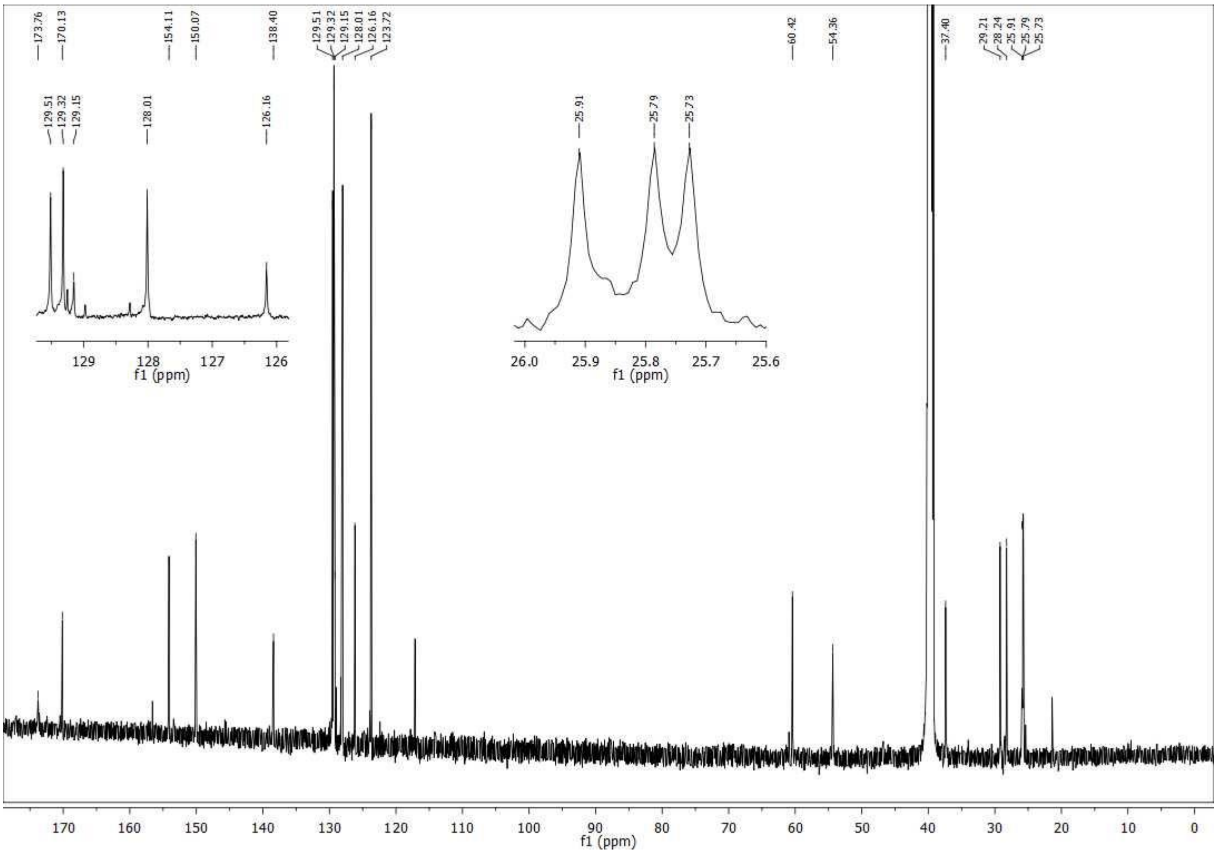

Compound **7**

^{1}H NMR

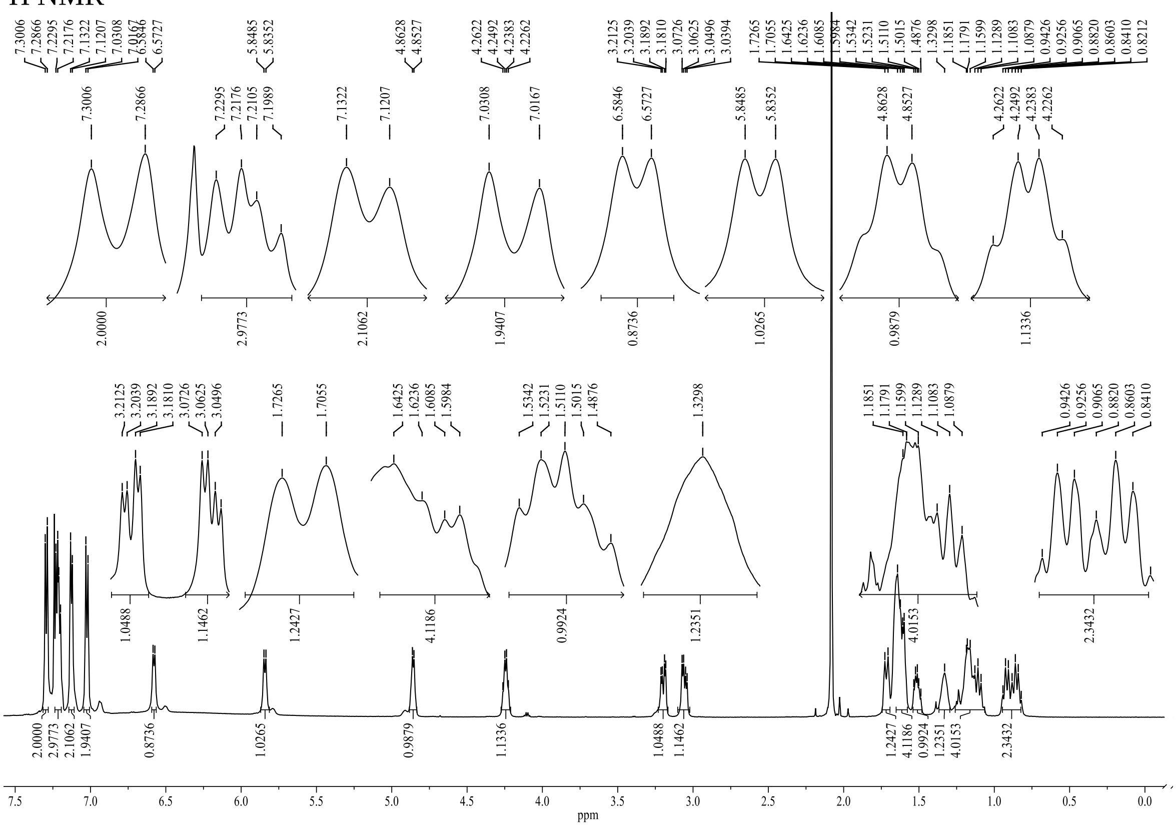

^{13}C NMR

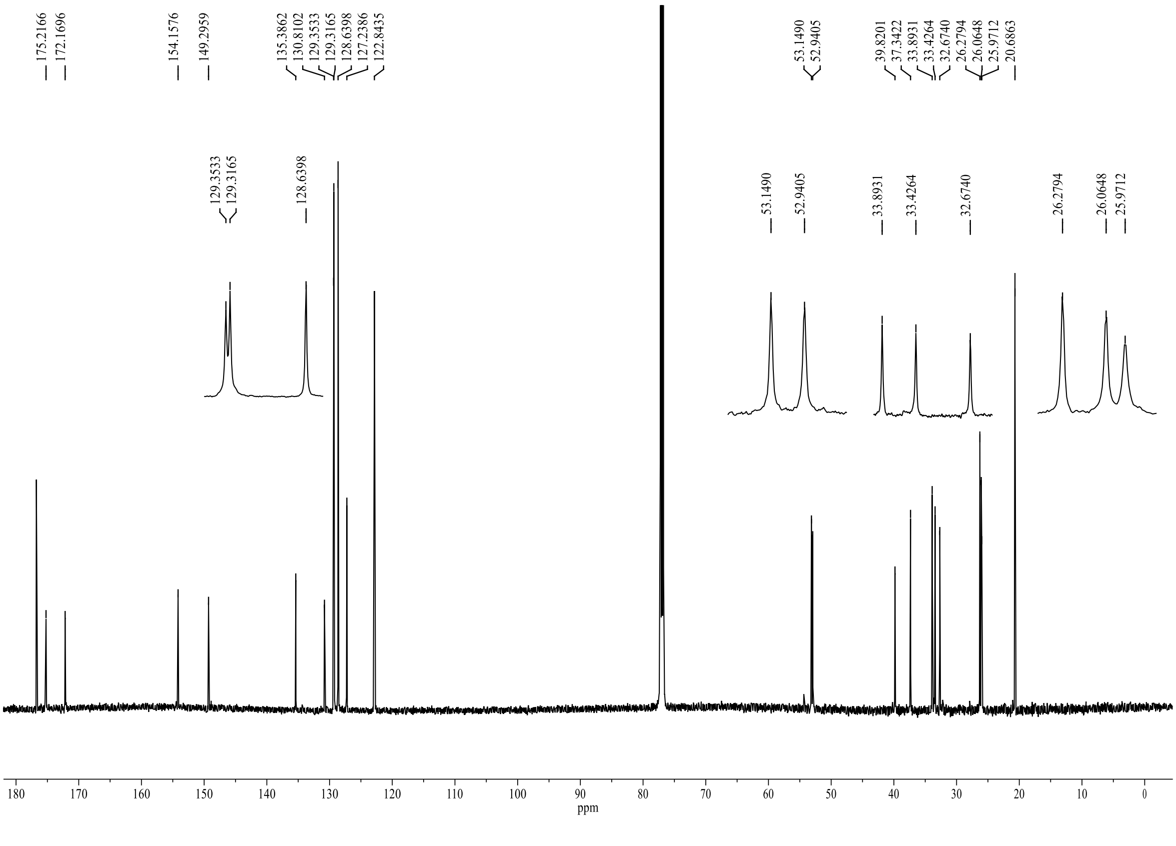

Compound **8**

^{1}H NMR

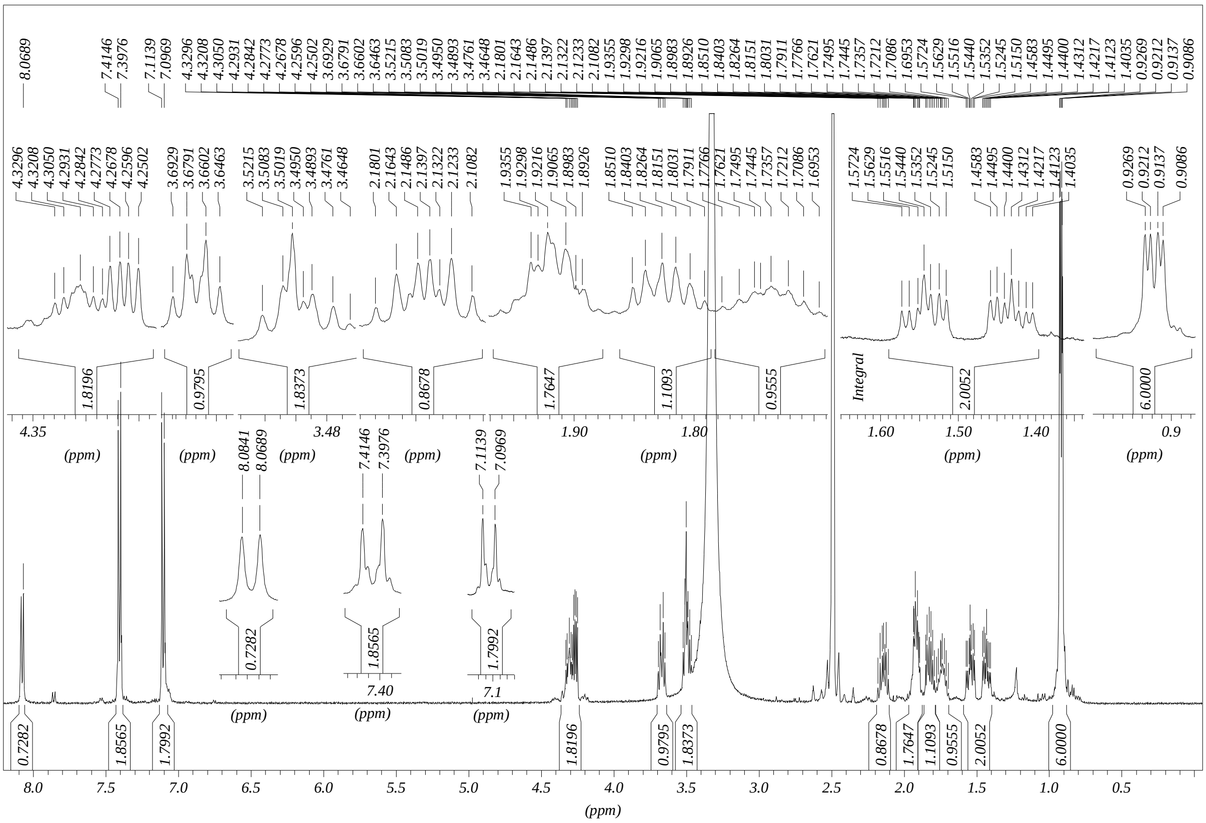

^{13}C NMR

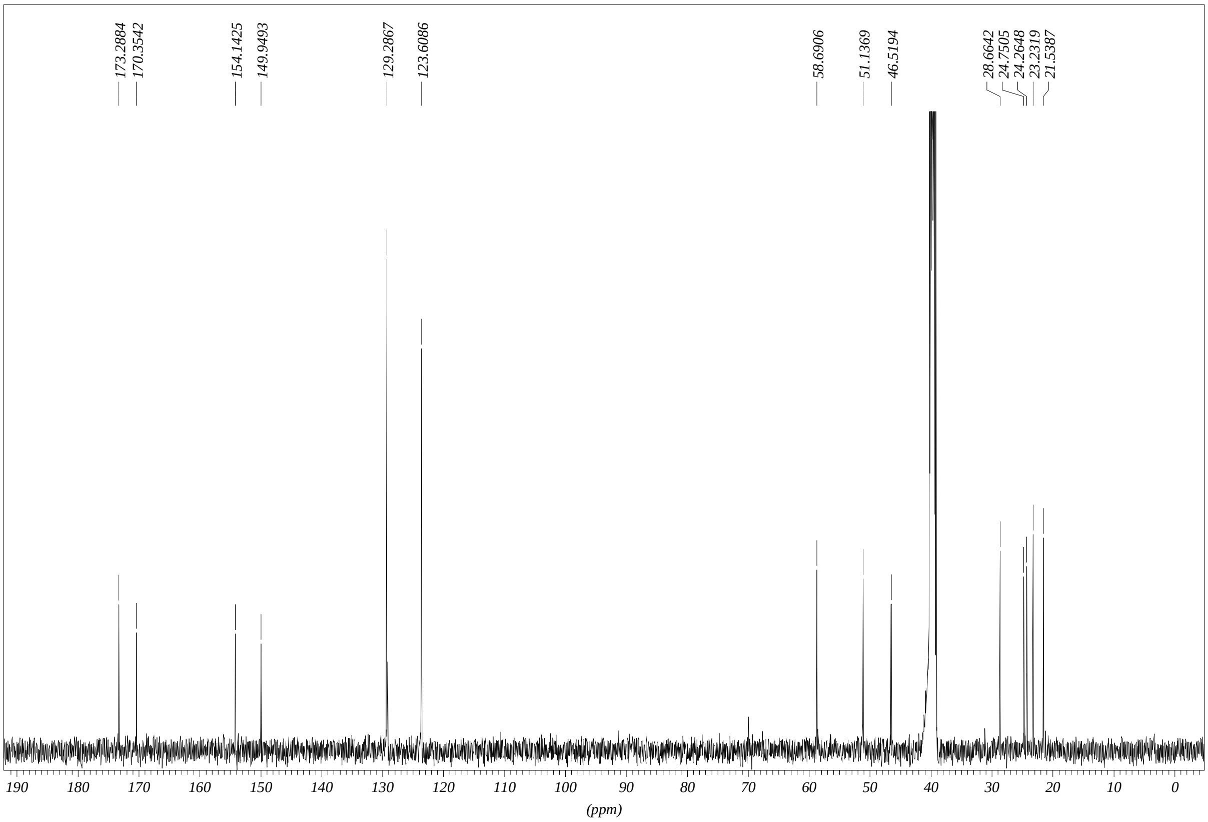

Compound **9**

^{1}H NMR

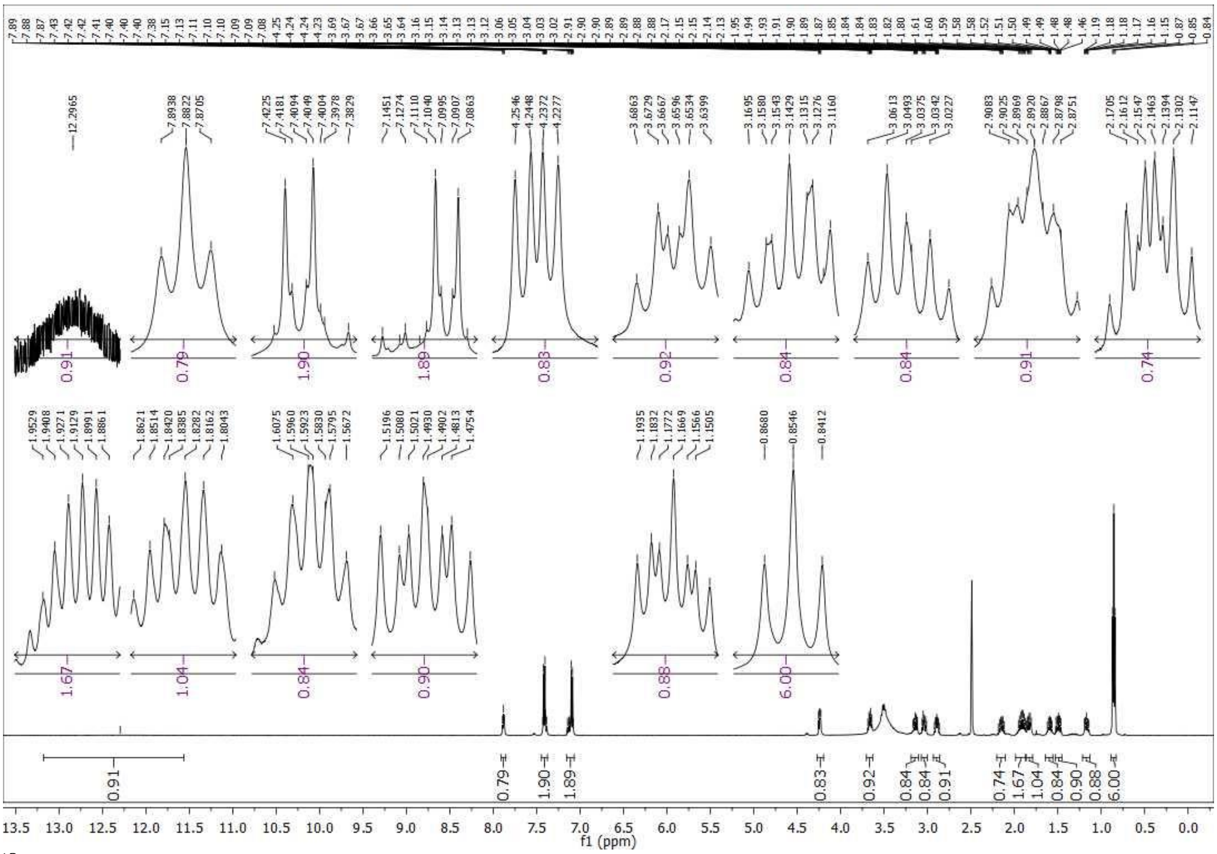

^{13}C NMR

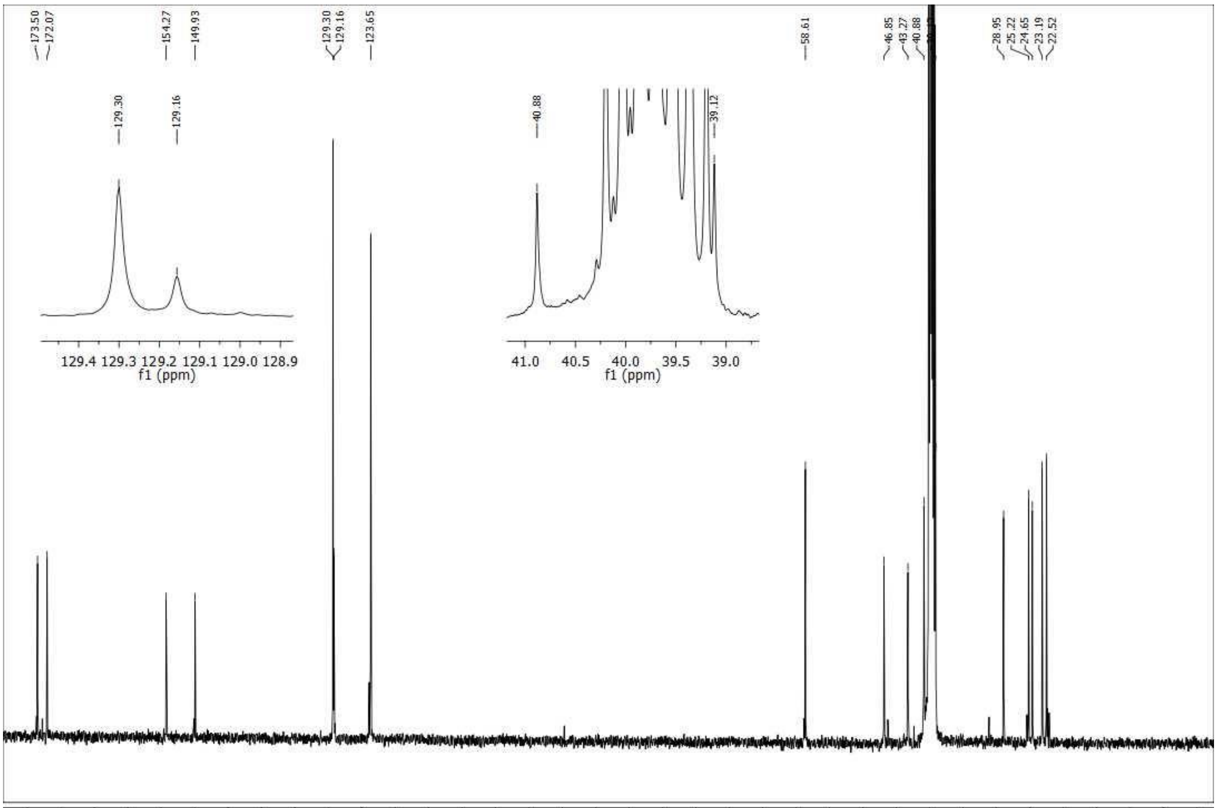

Compound **10**

^{1}H NMR

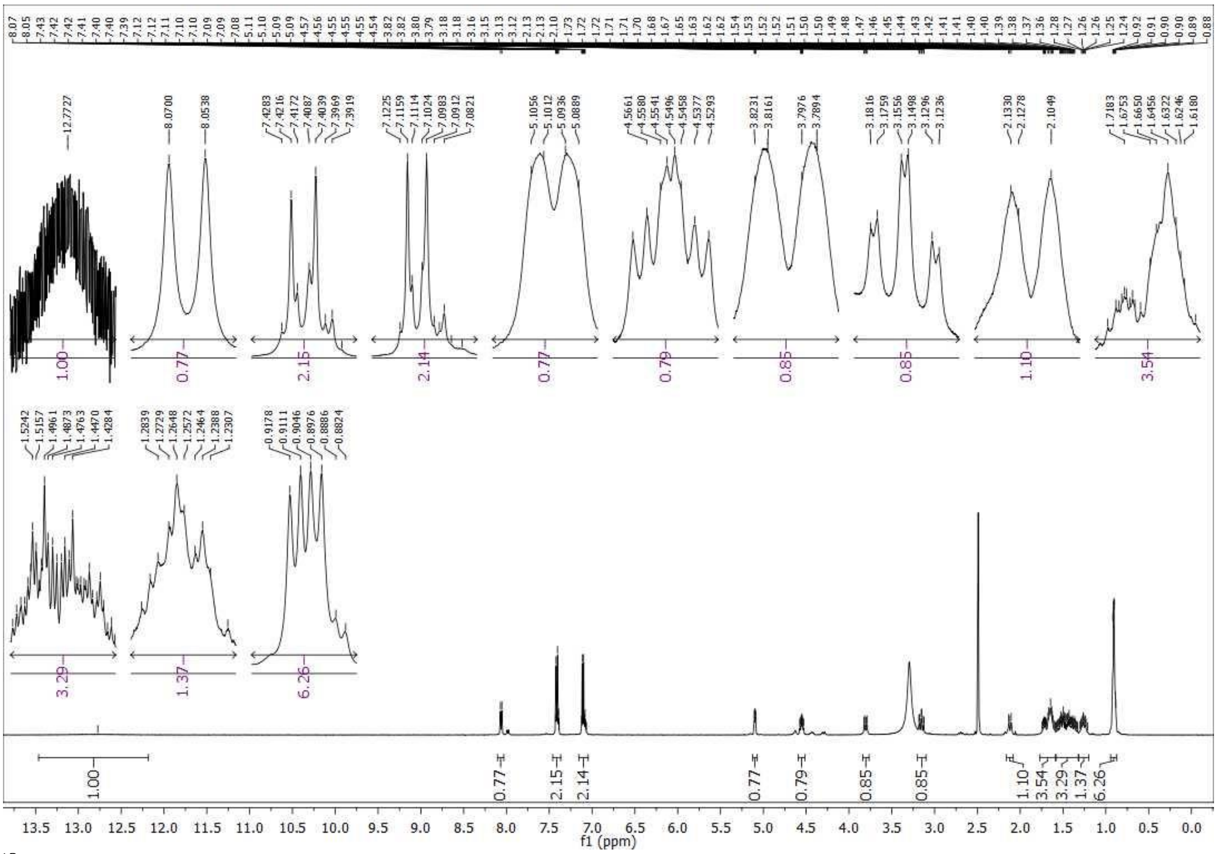

^{13}C NMR

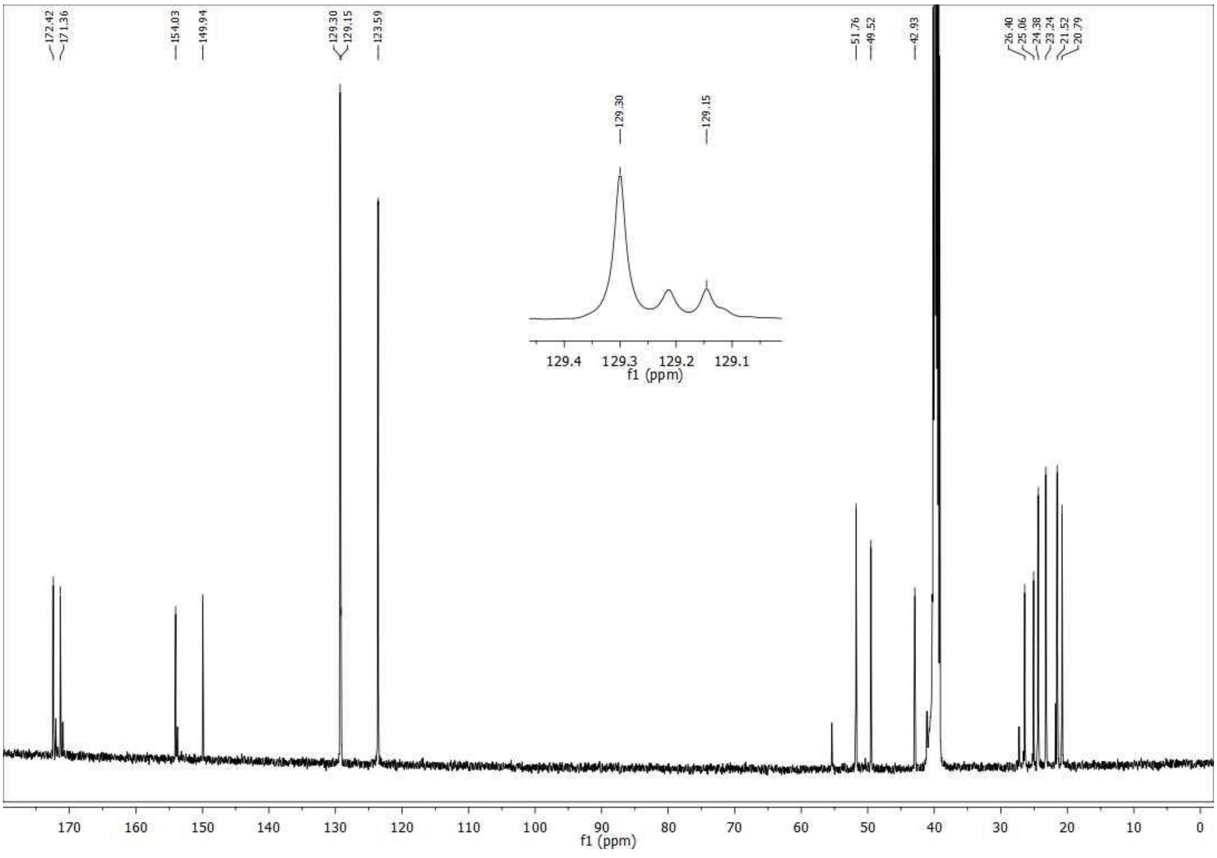

Compound **11**

^{1}H NMR

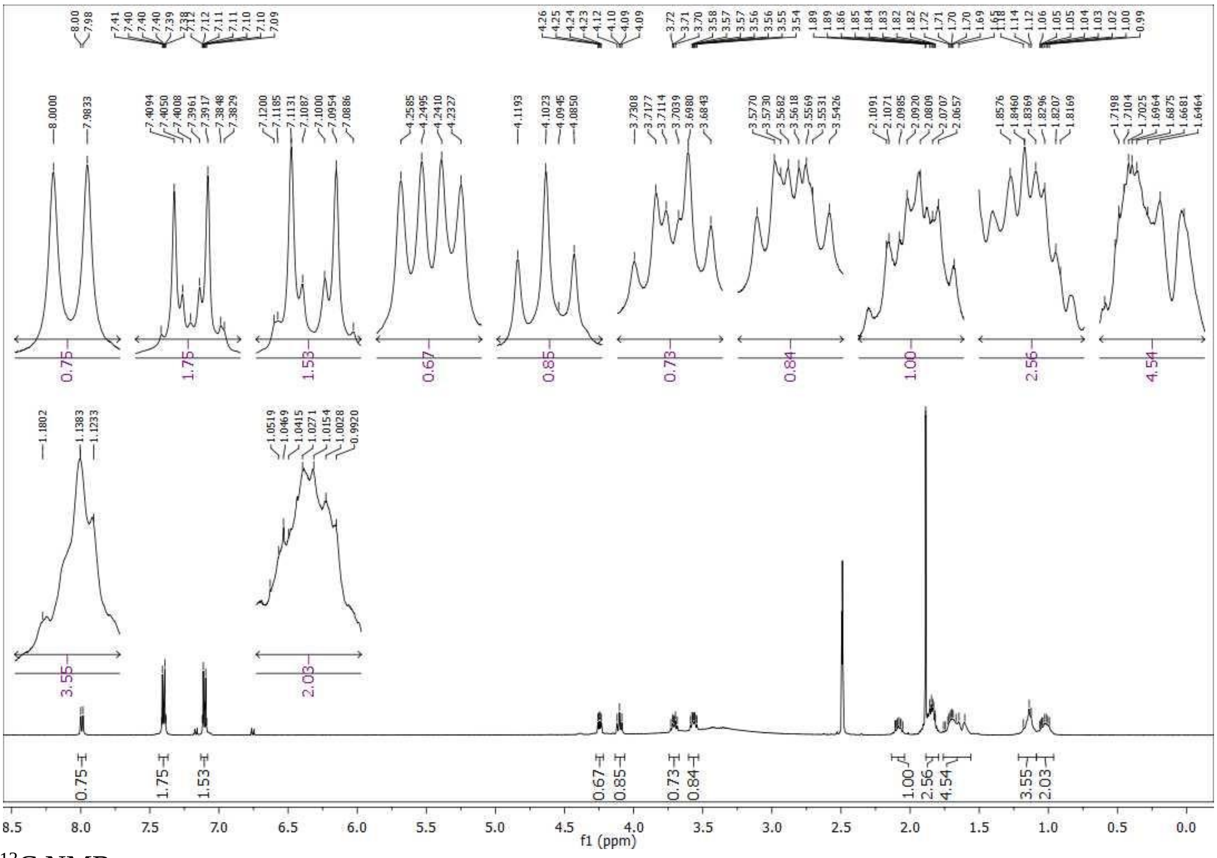

^{13}C NMR

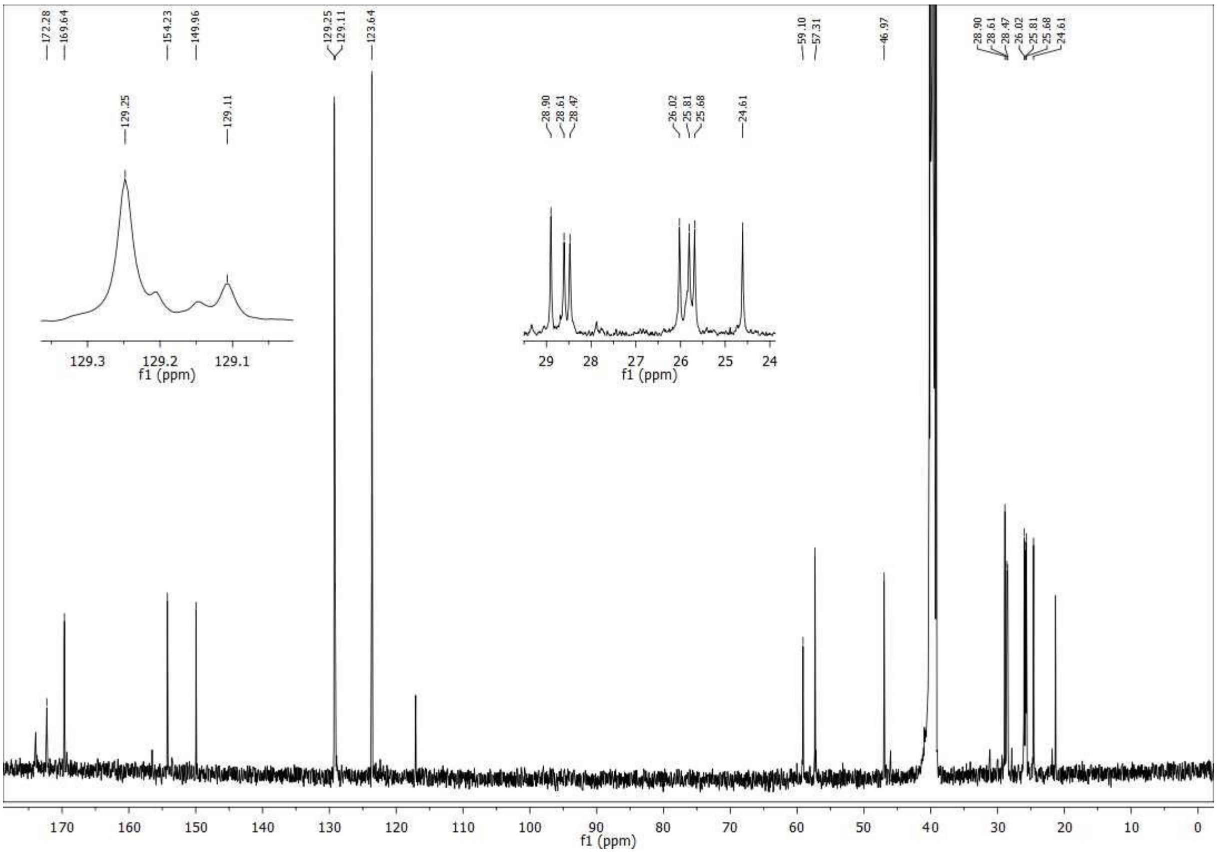

Compound **12**

^{1}H NMR

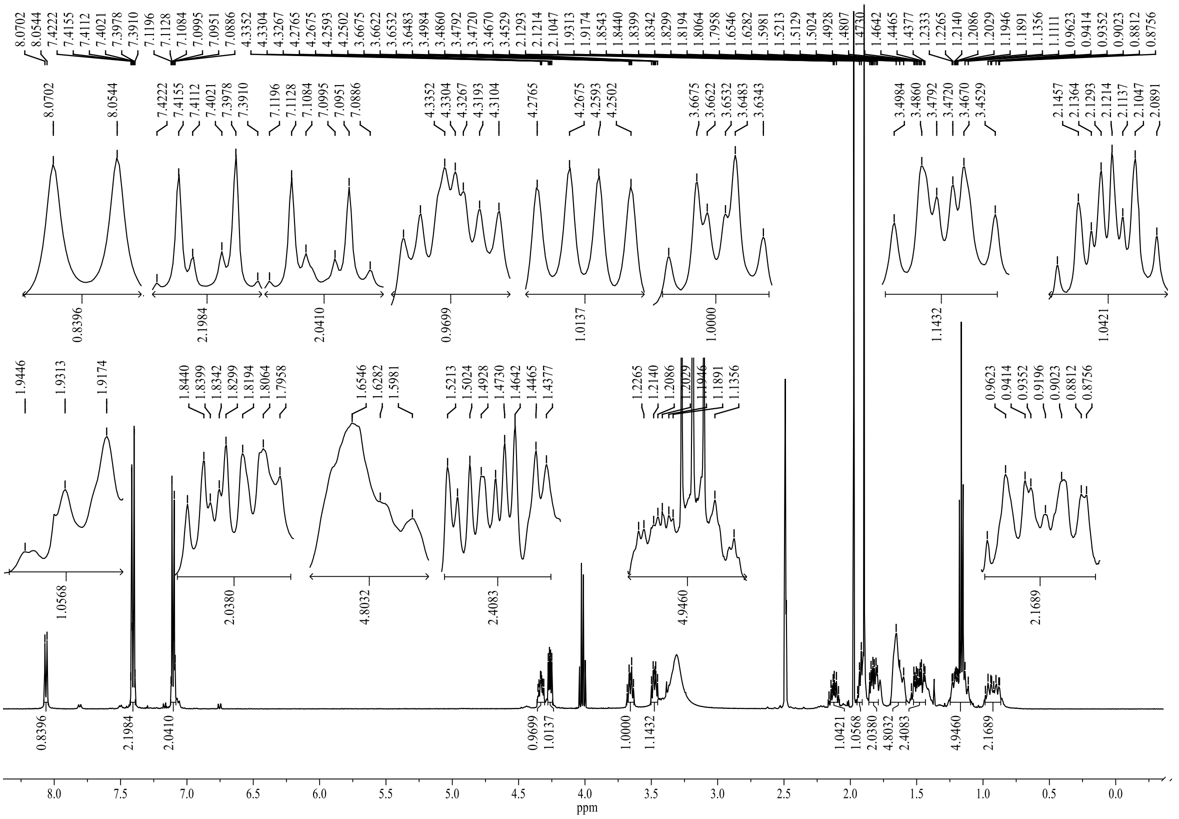

^{13}C NMR

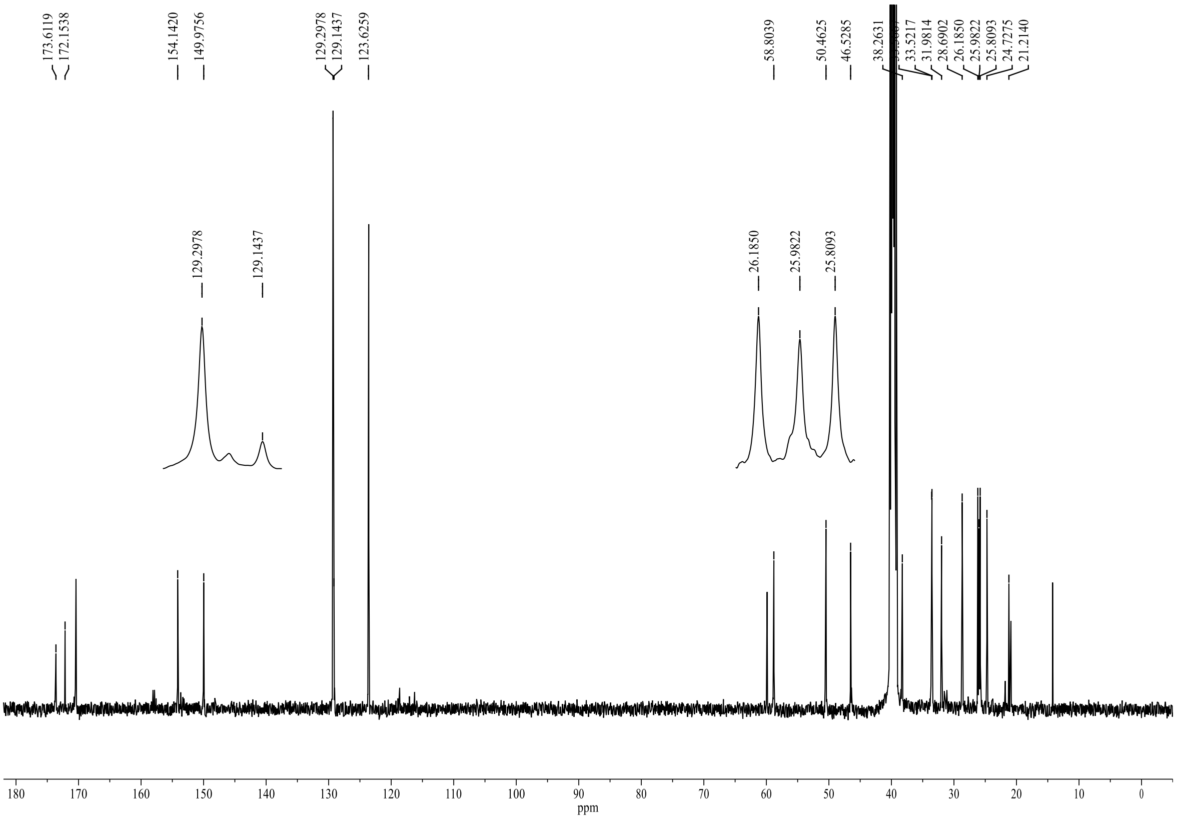

Compound **13**

^{1}H NMR

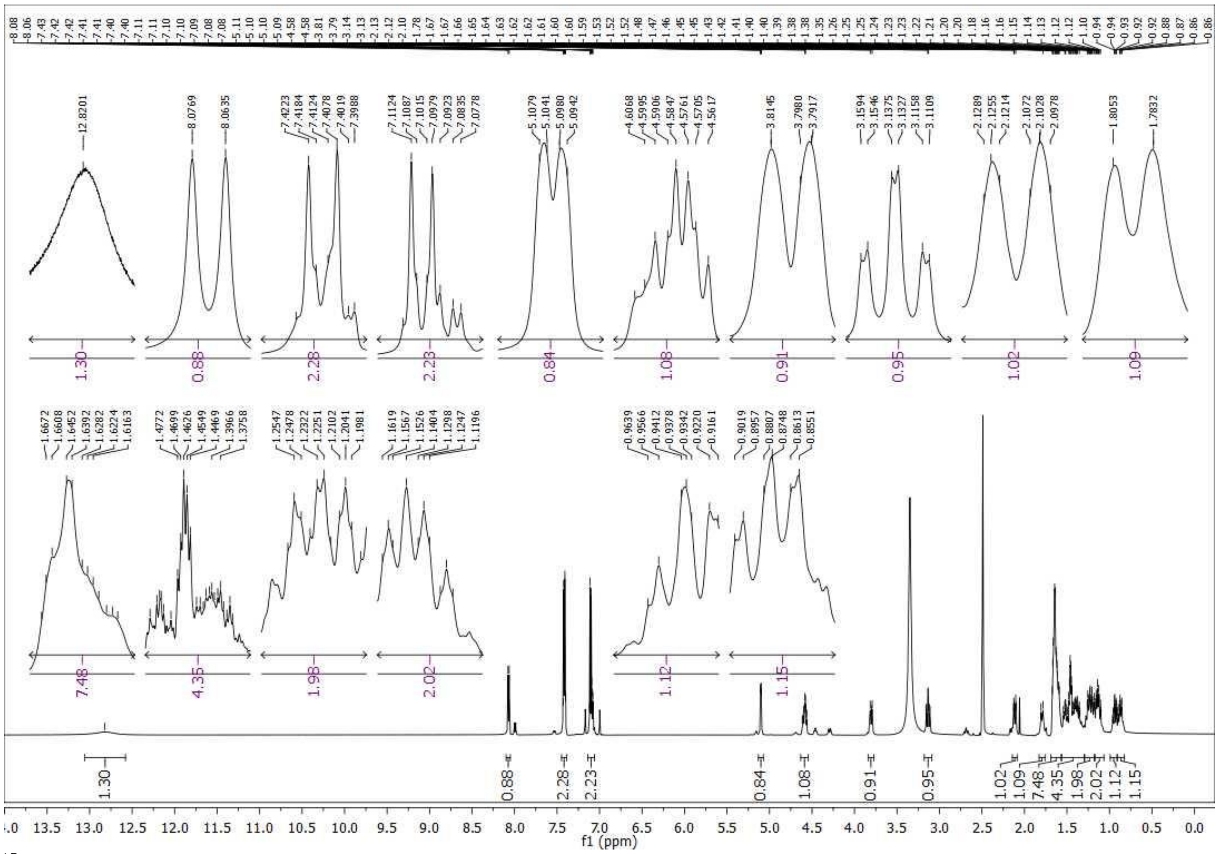

^{13}C NMR

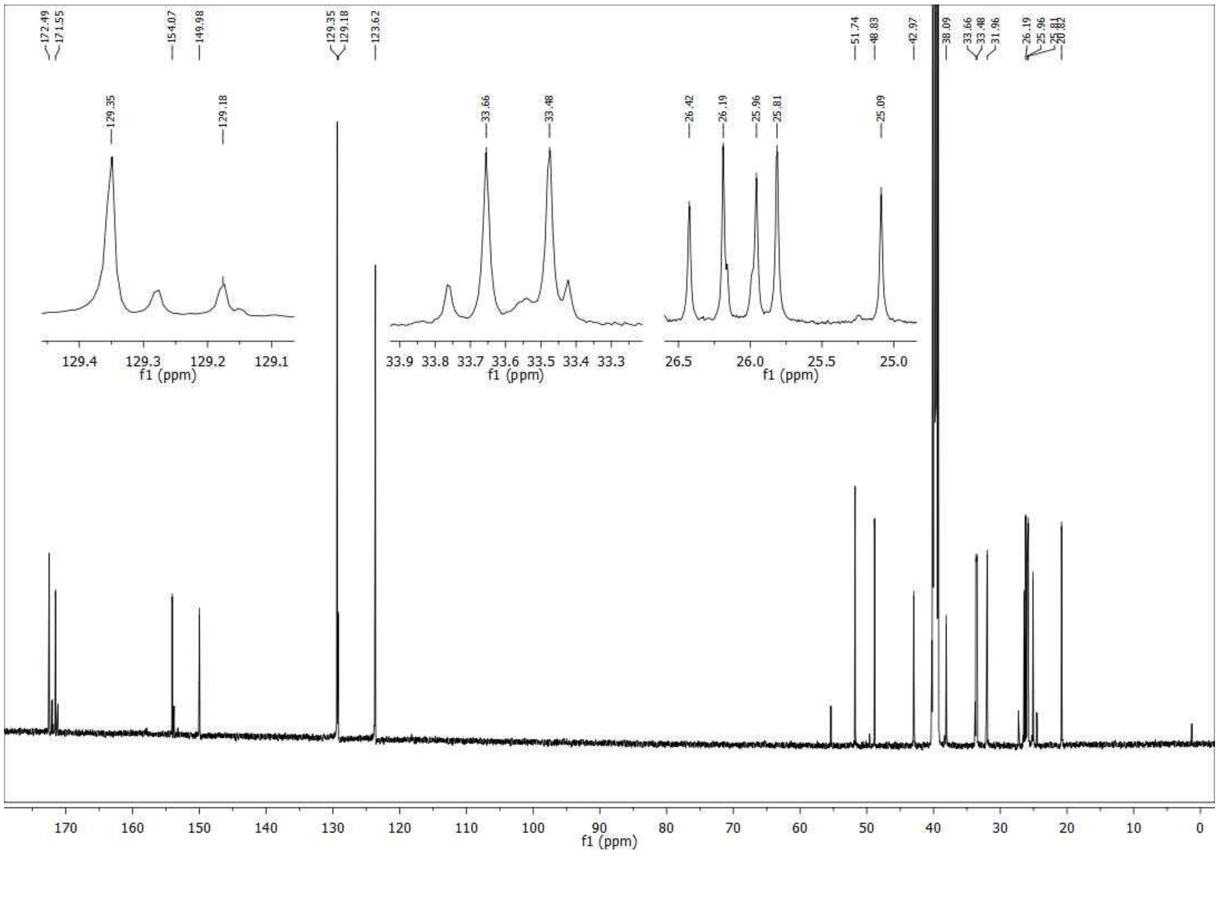

Compound **14**

^{1}H NMR

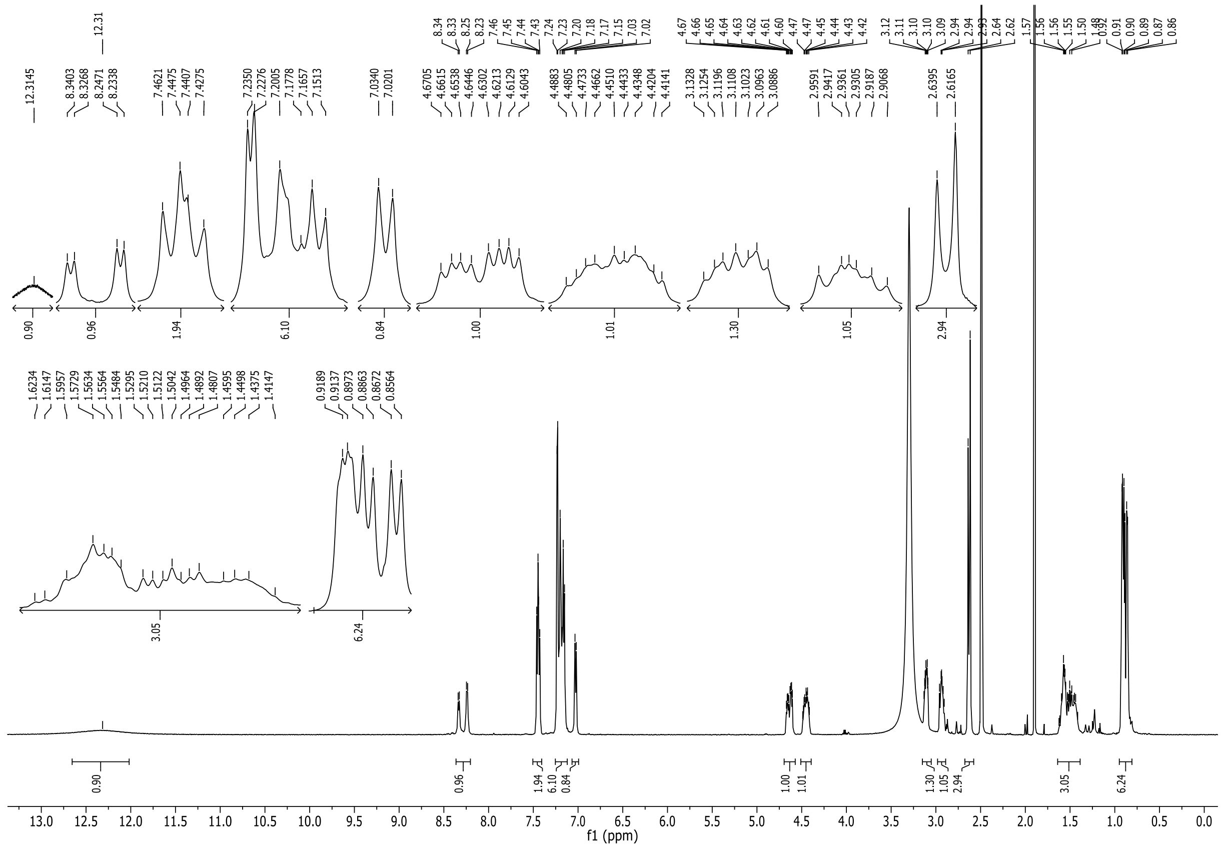

^{13}C NMR

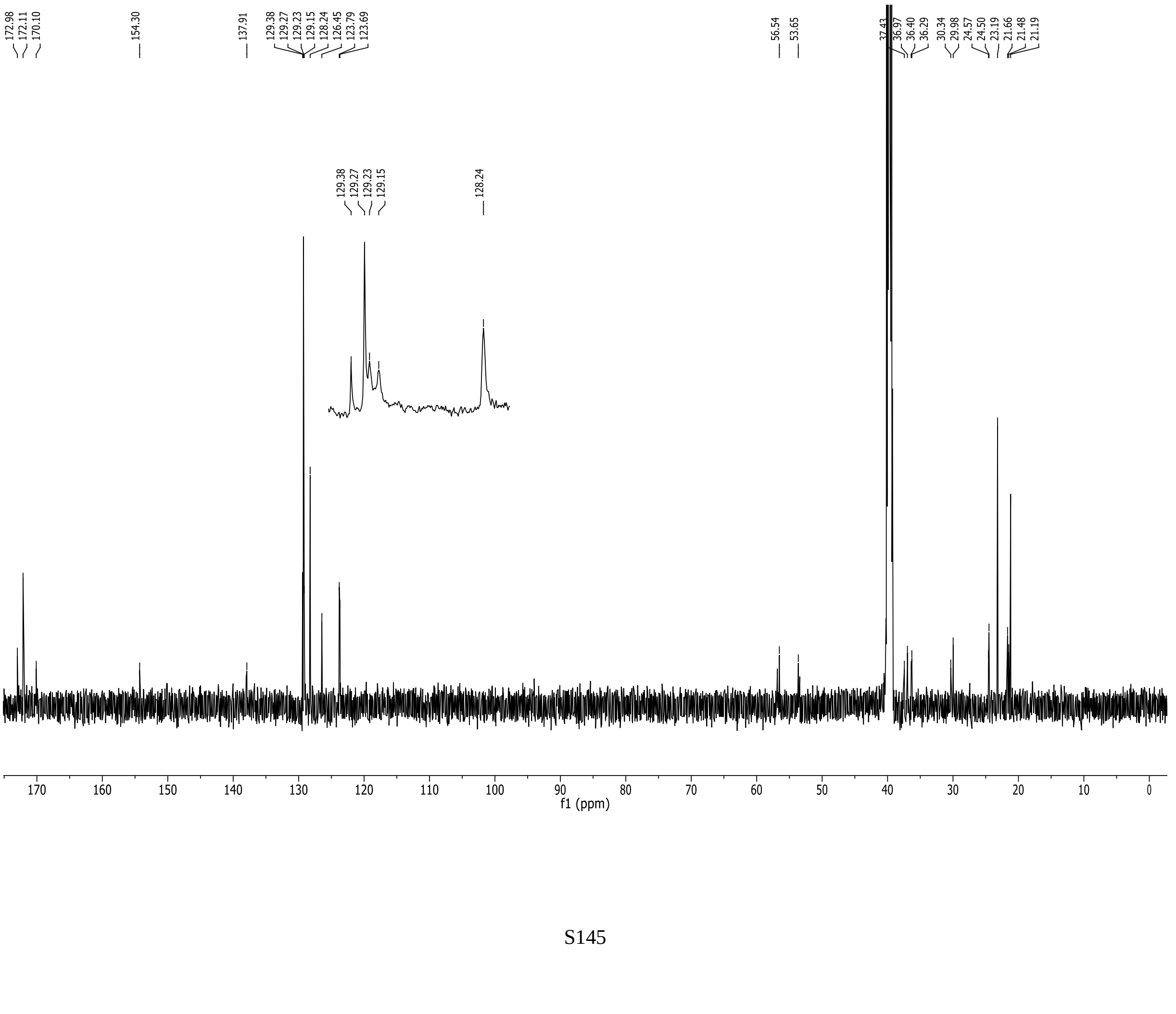

Compound **15**

^{1}H NMR

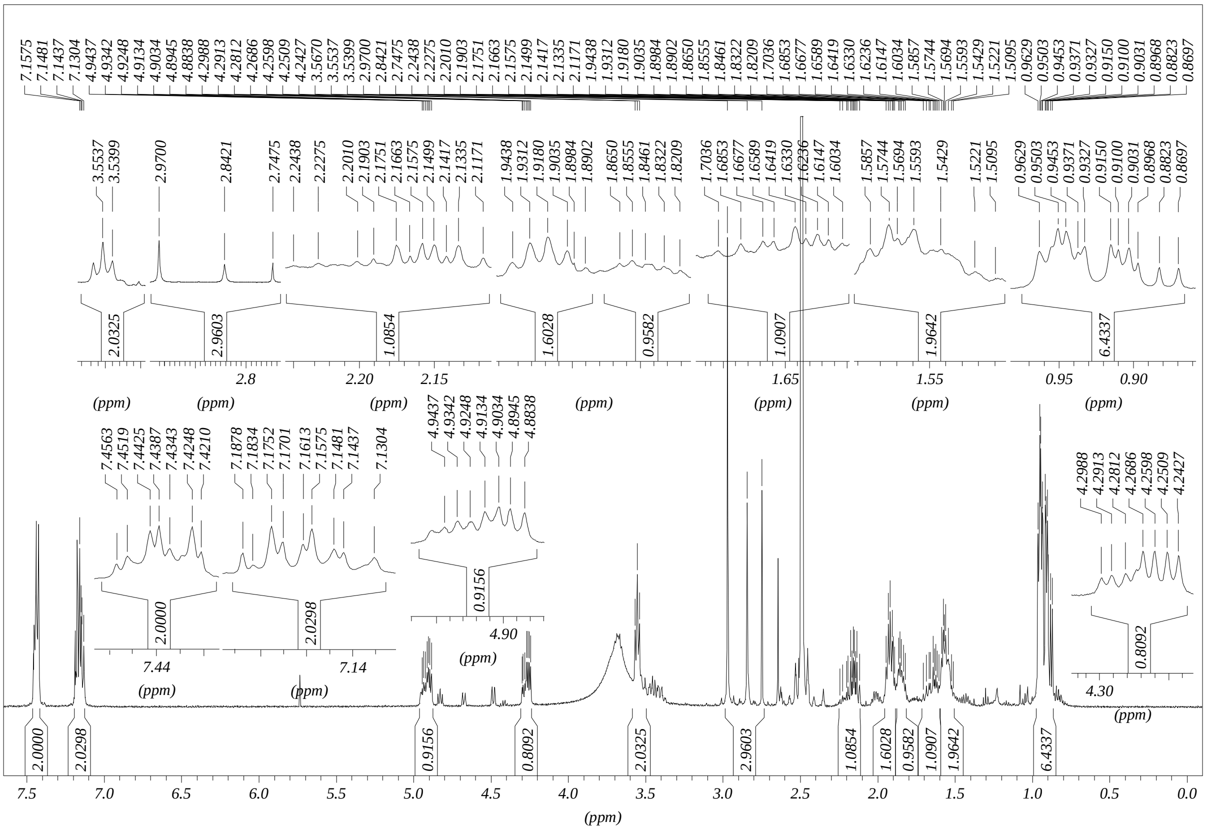

^{13}C NMR

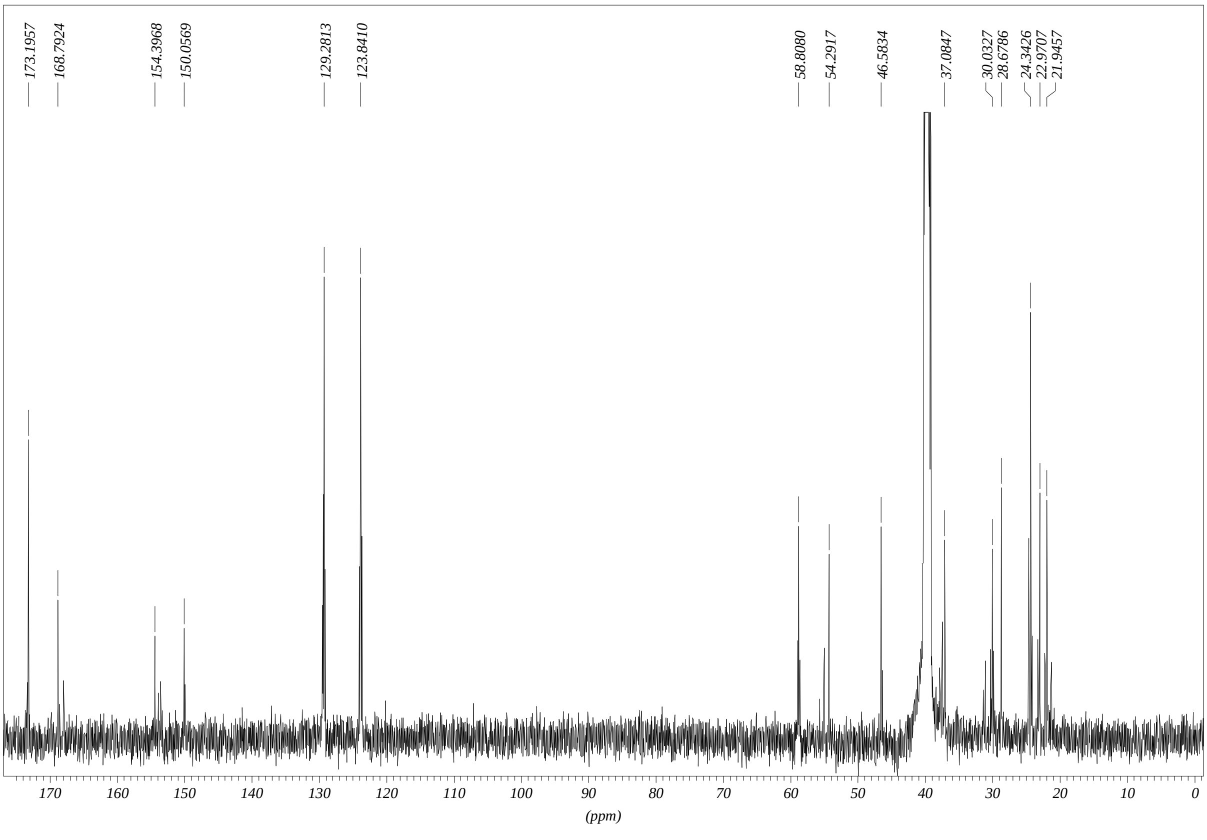

Compound **16**

^{1}H NMR

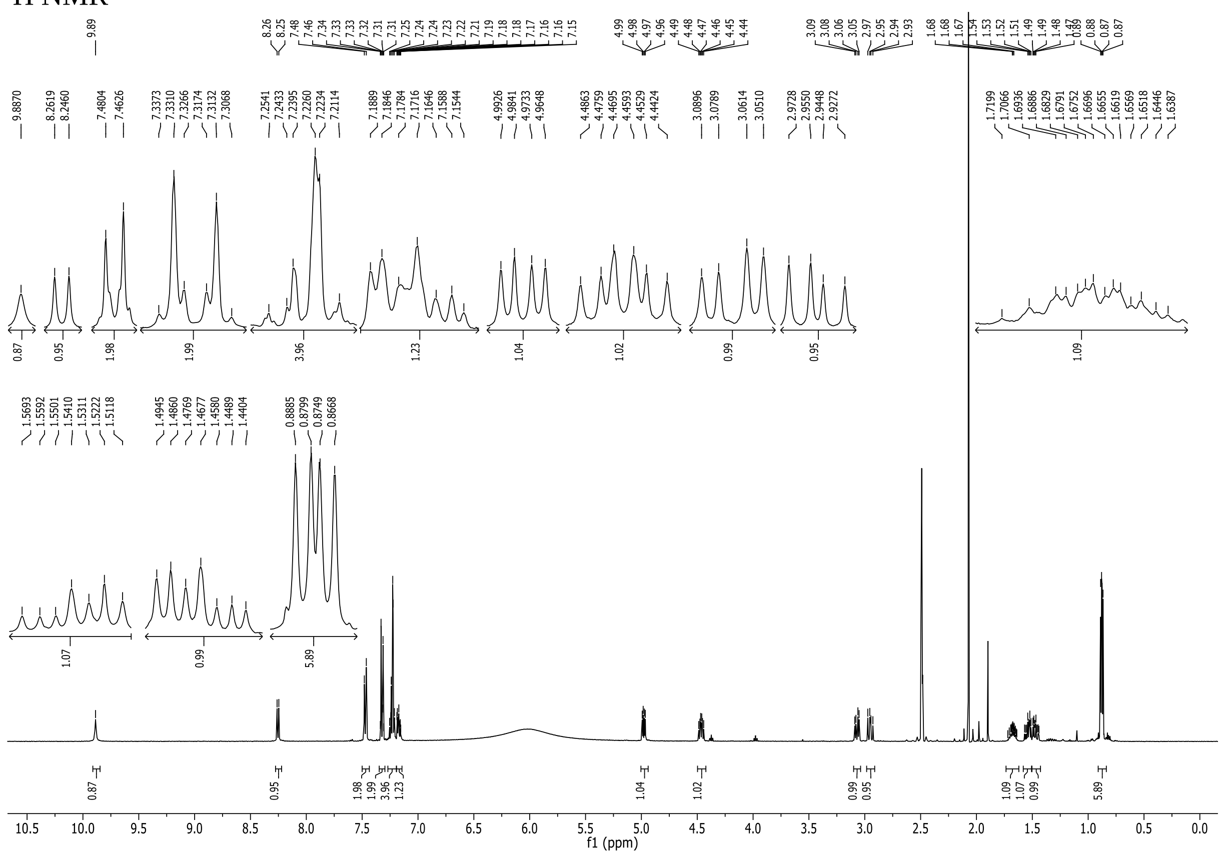

^{13}C NMR

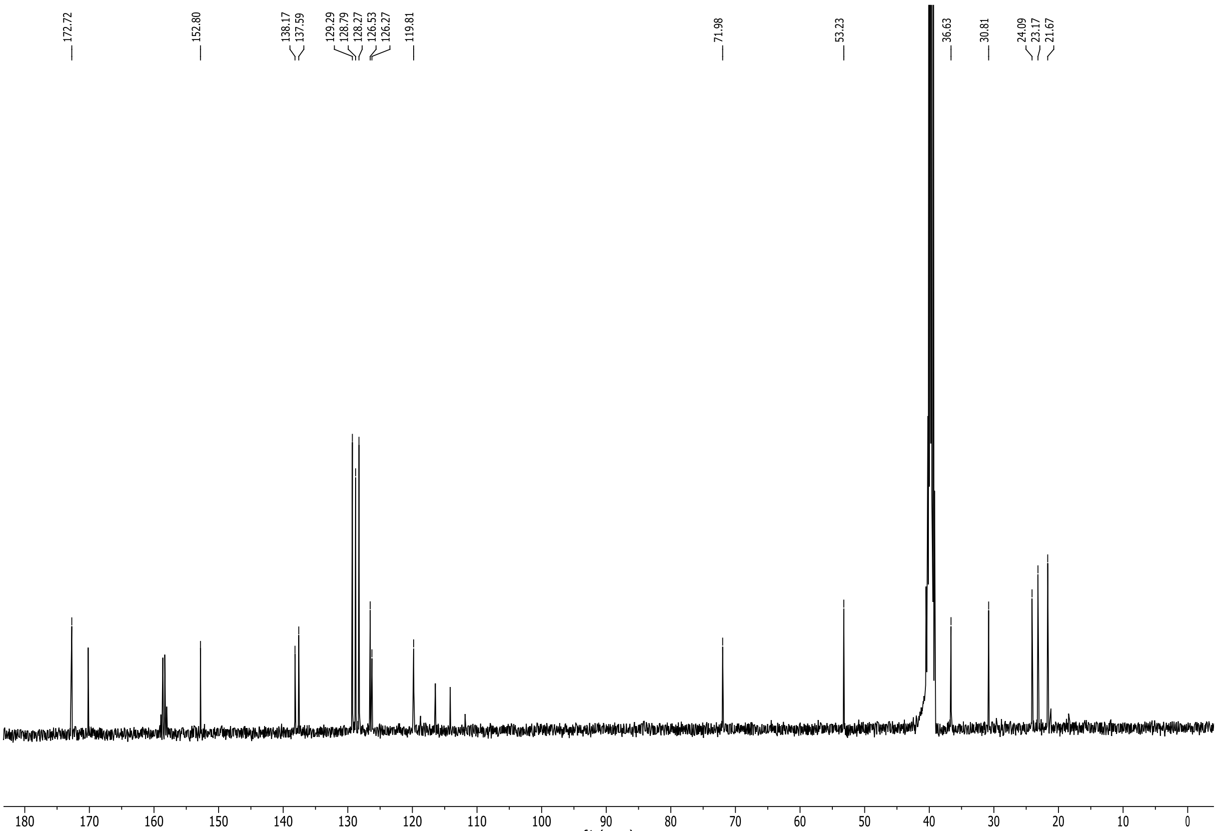

Compound **17**

^{1}H NMR

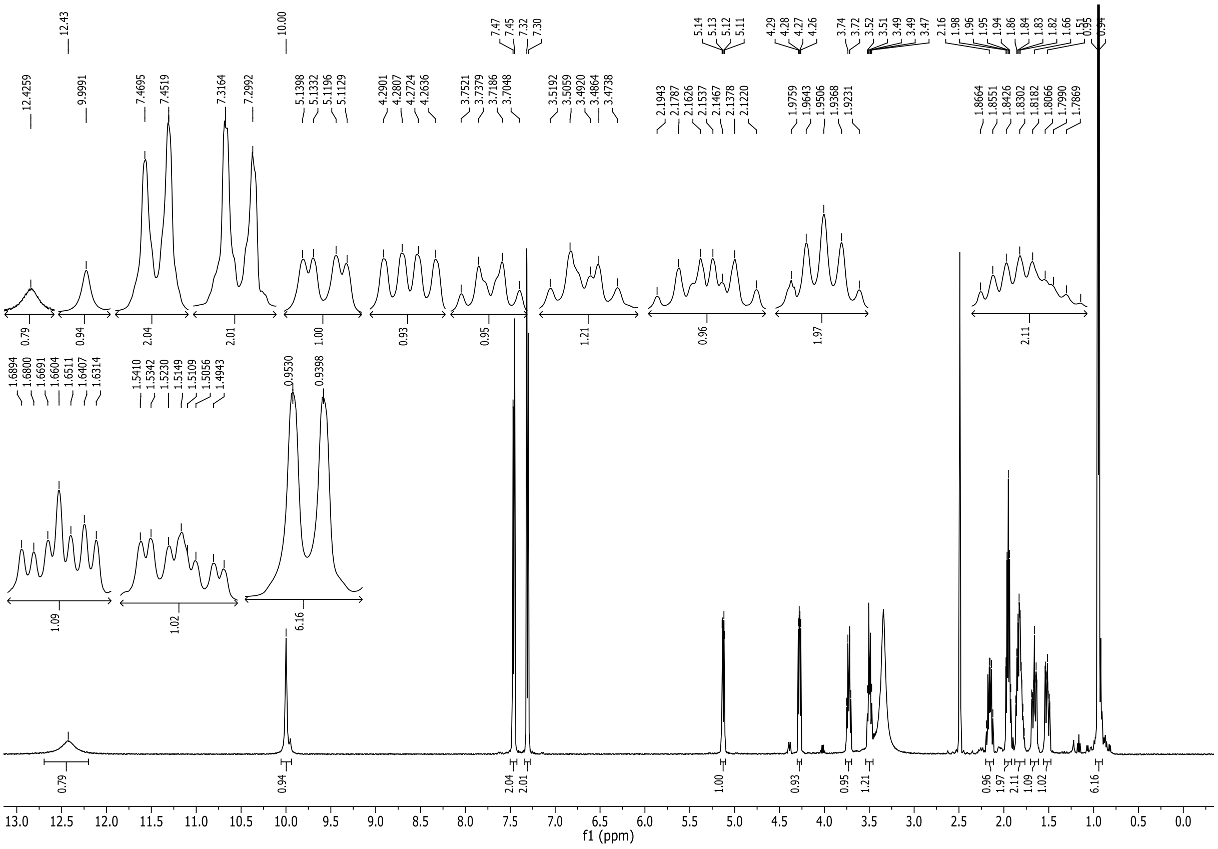

^{13}C NMR

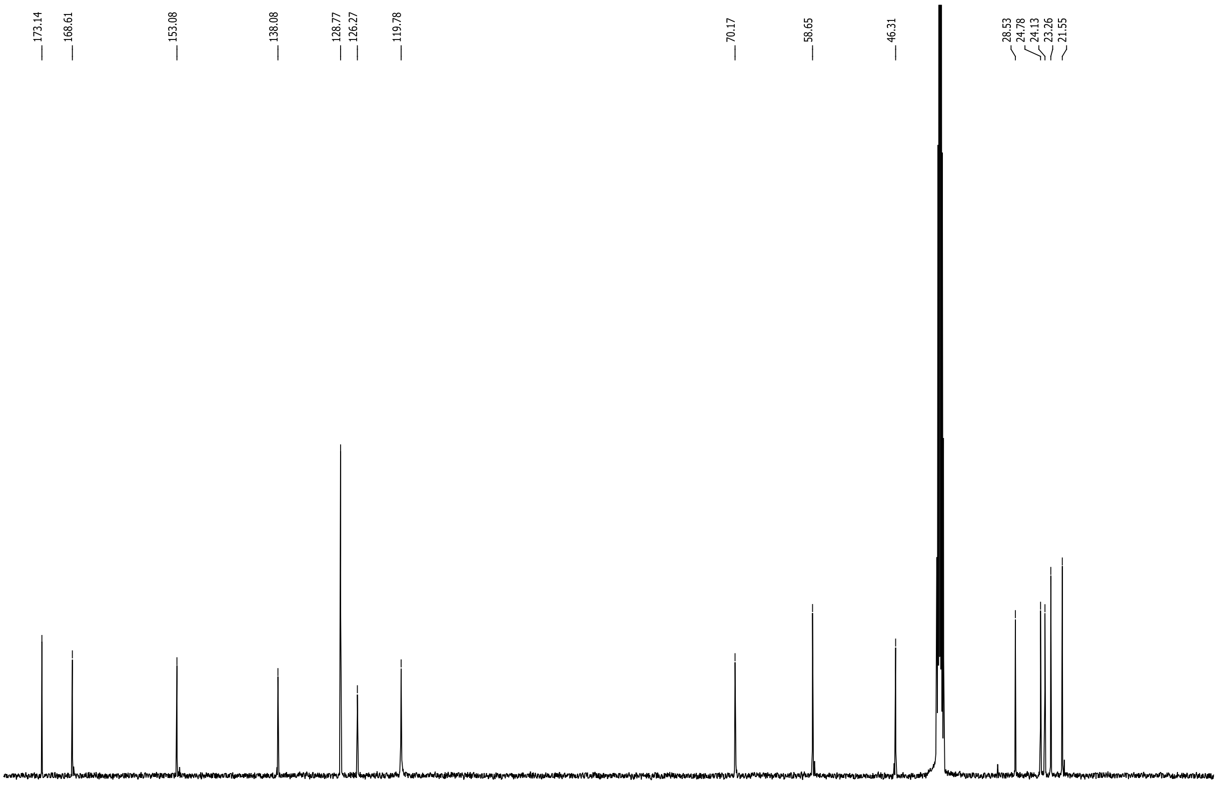

Compound **18**

^{1}H NMR

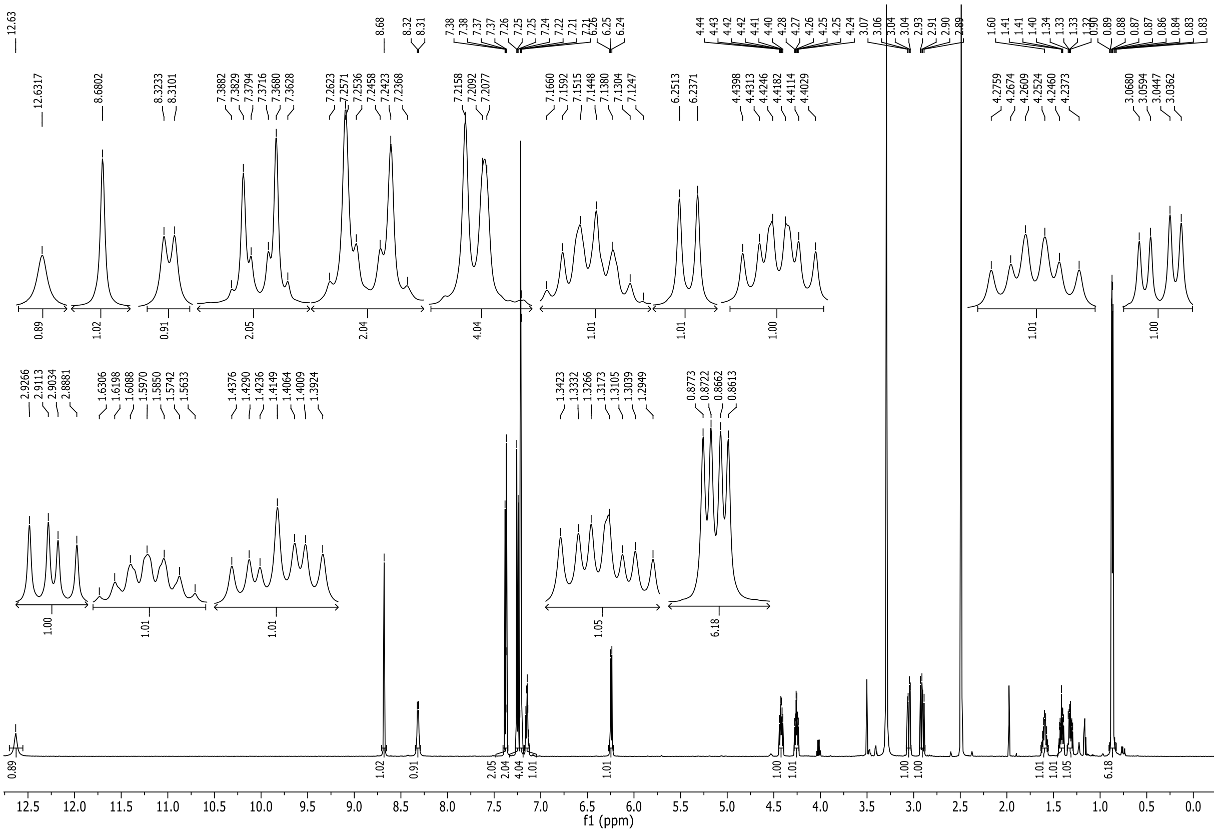

^{13}C NMR

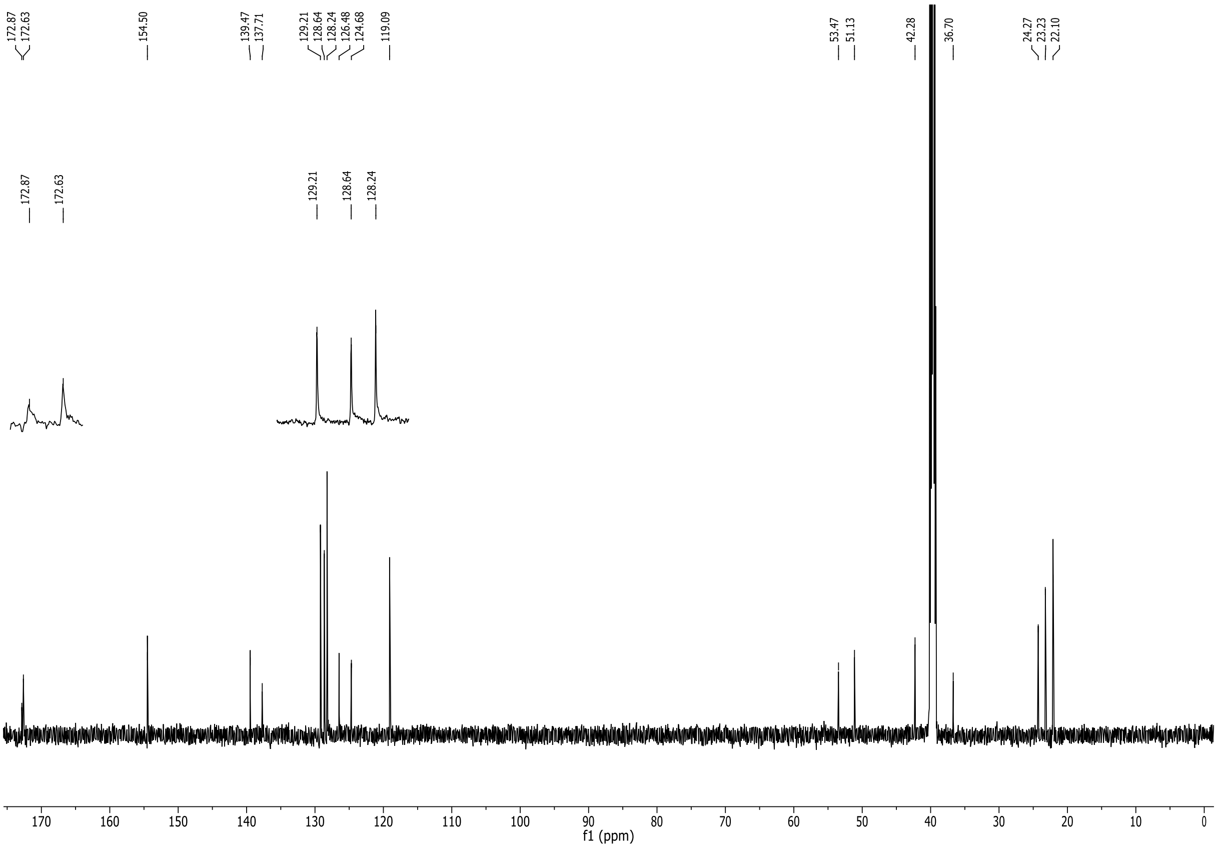

Compound **19**

^{1}H NMR

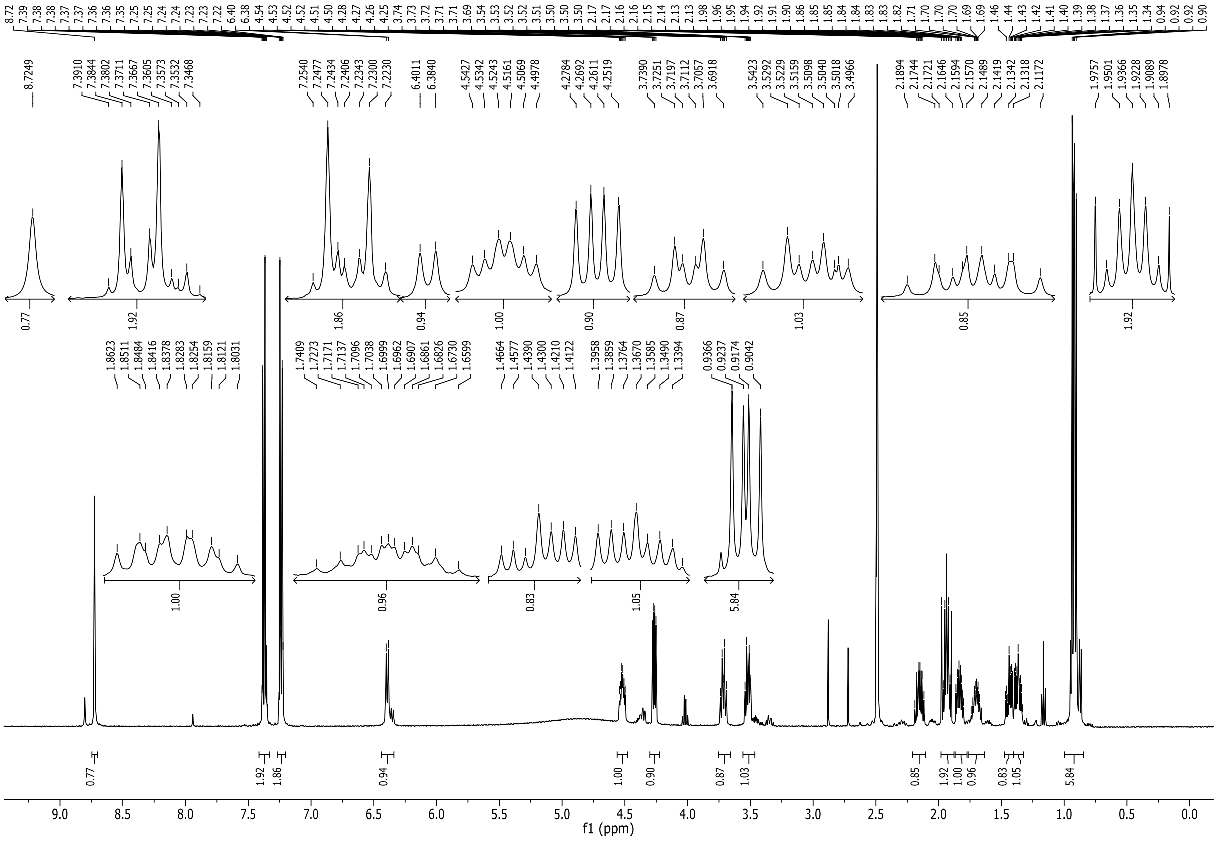

^{13}C NMR

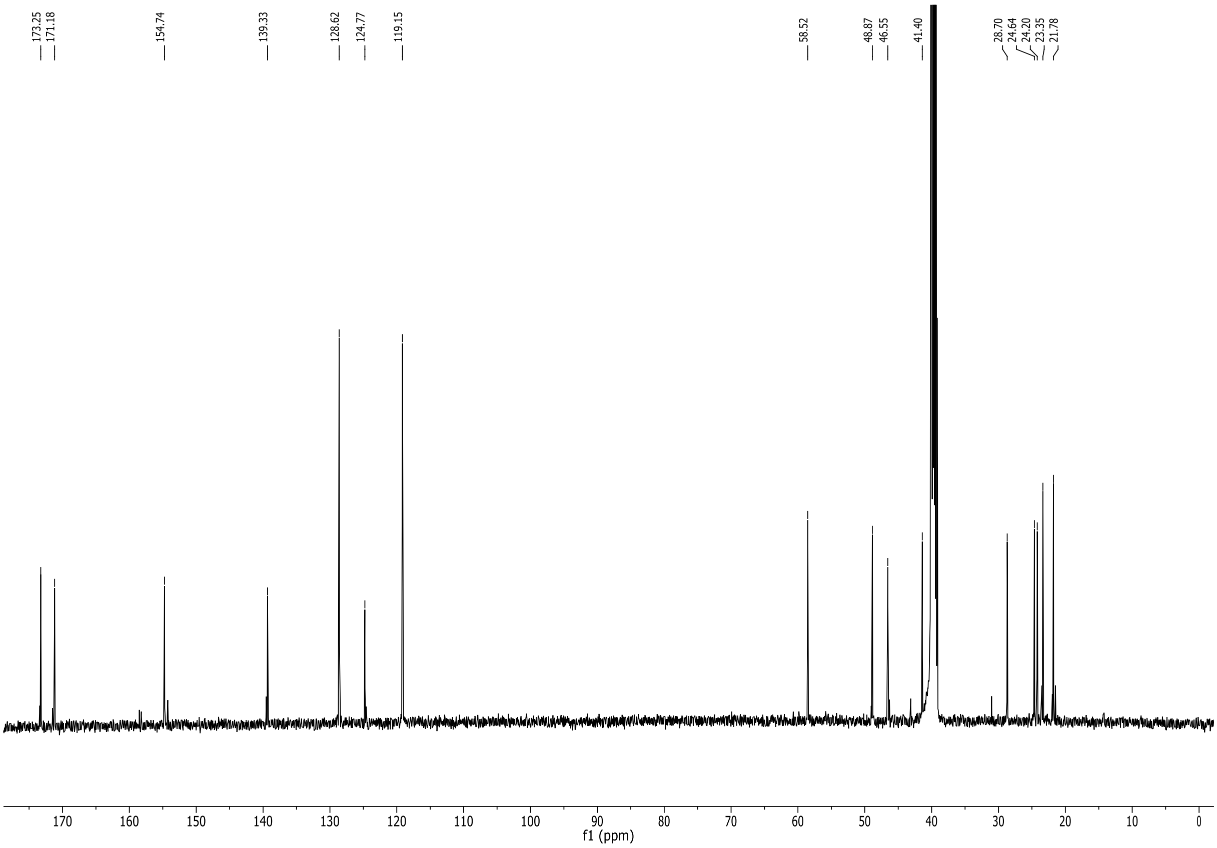

Compound **20**

^{1}H NMR

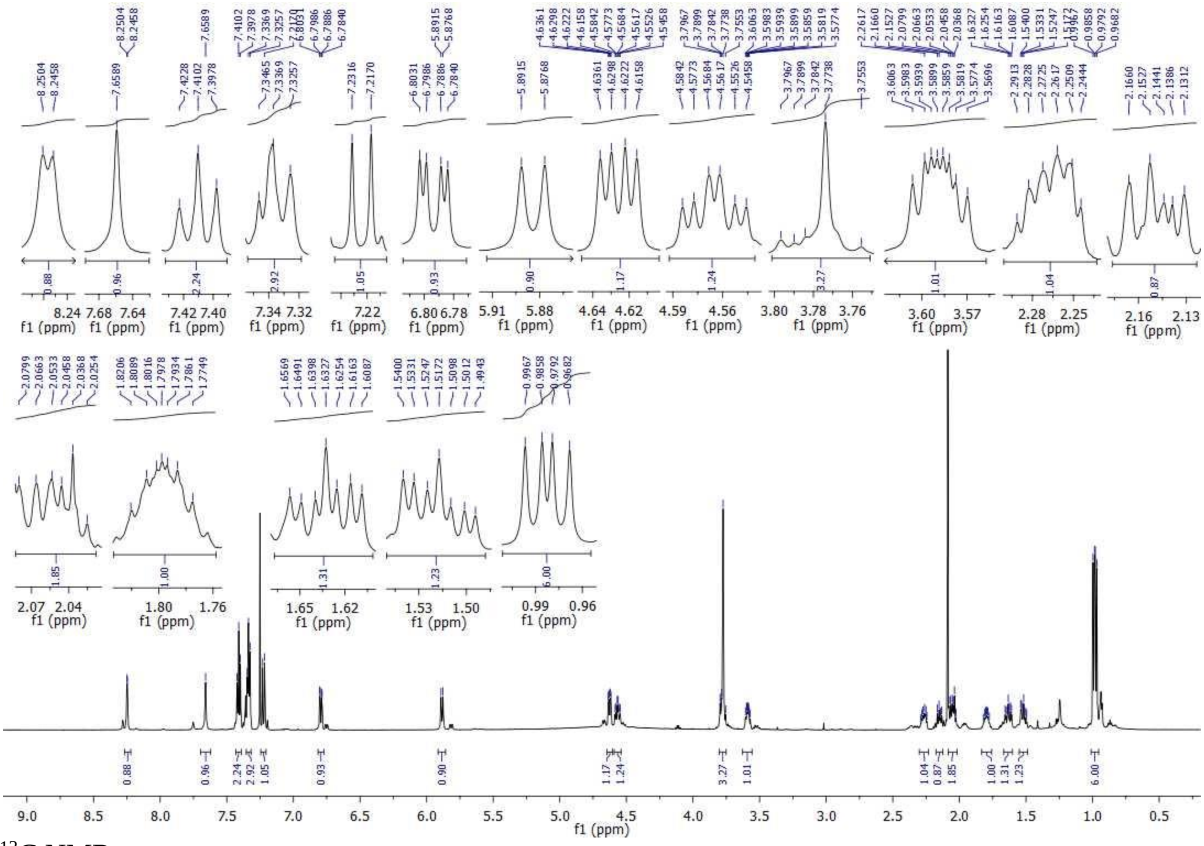

^{13}C NMR

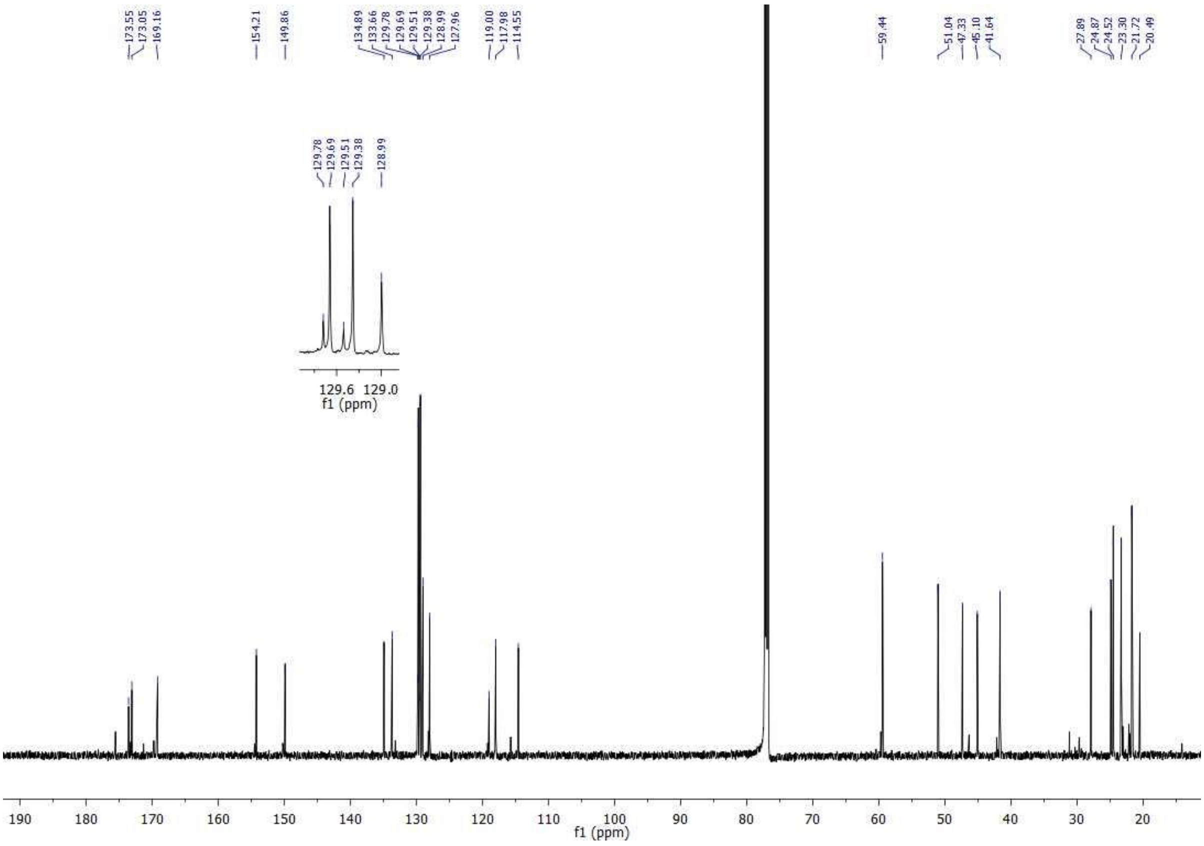

Compound **21**

^{1}H NMR

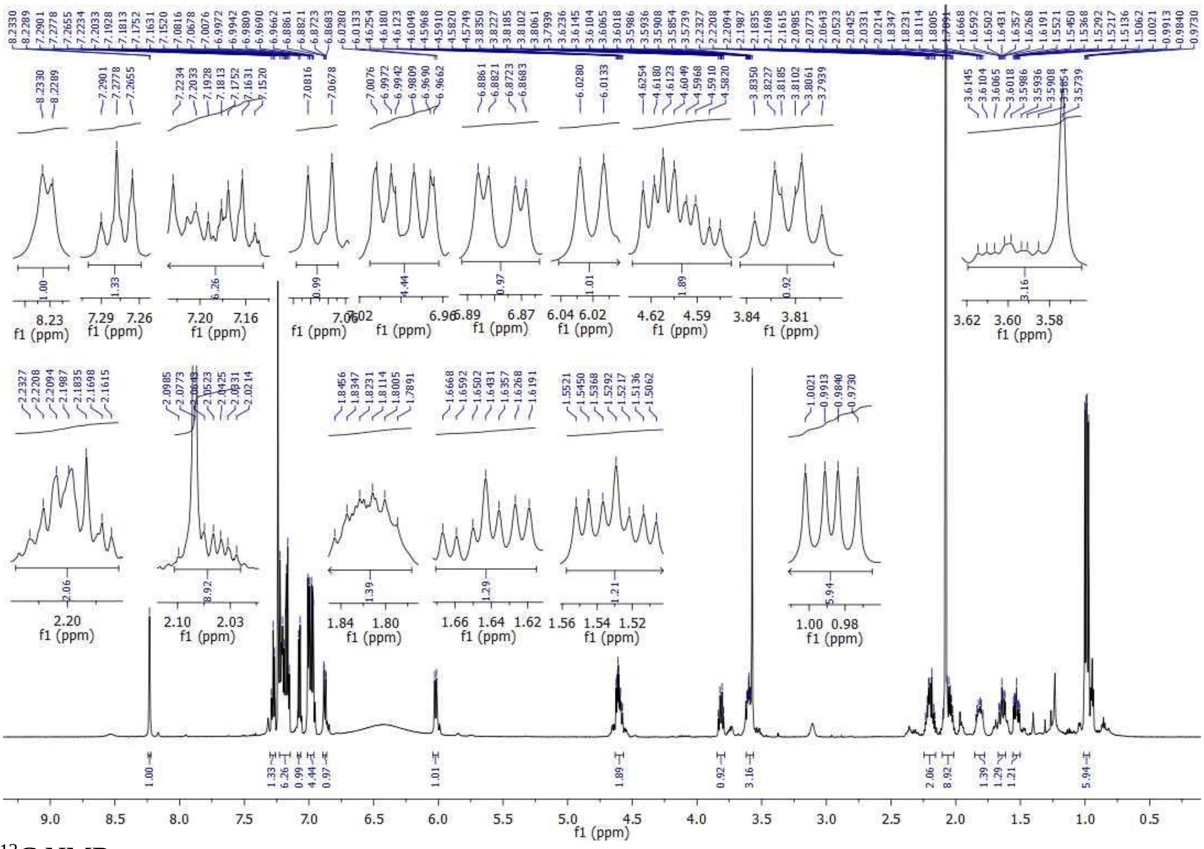

^{13}C NMR

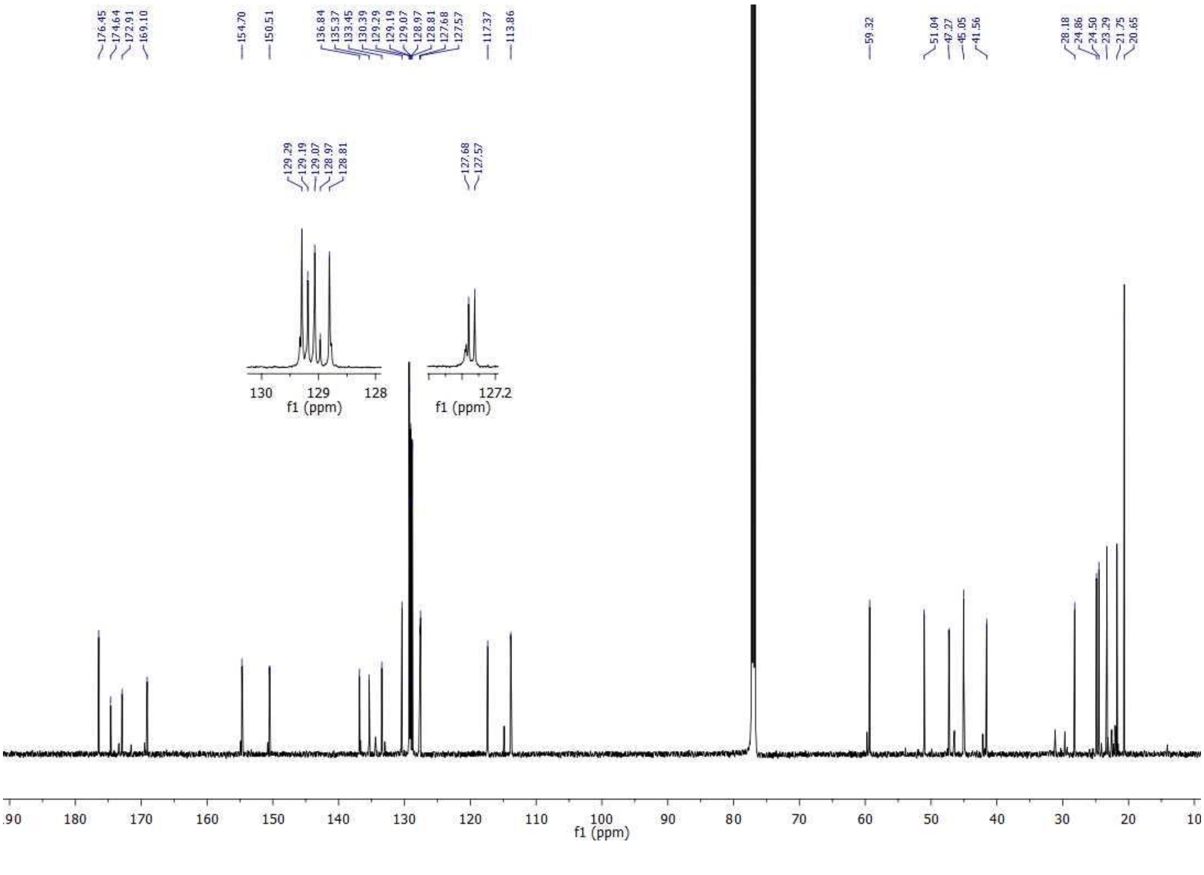

Compound **22**

^{1}H NMR

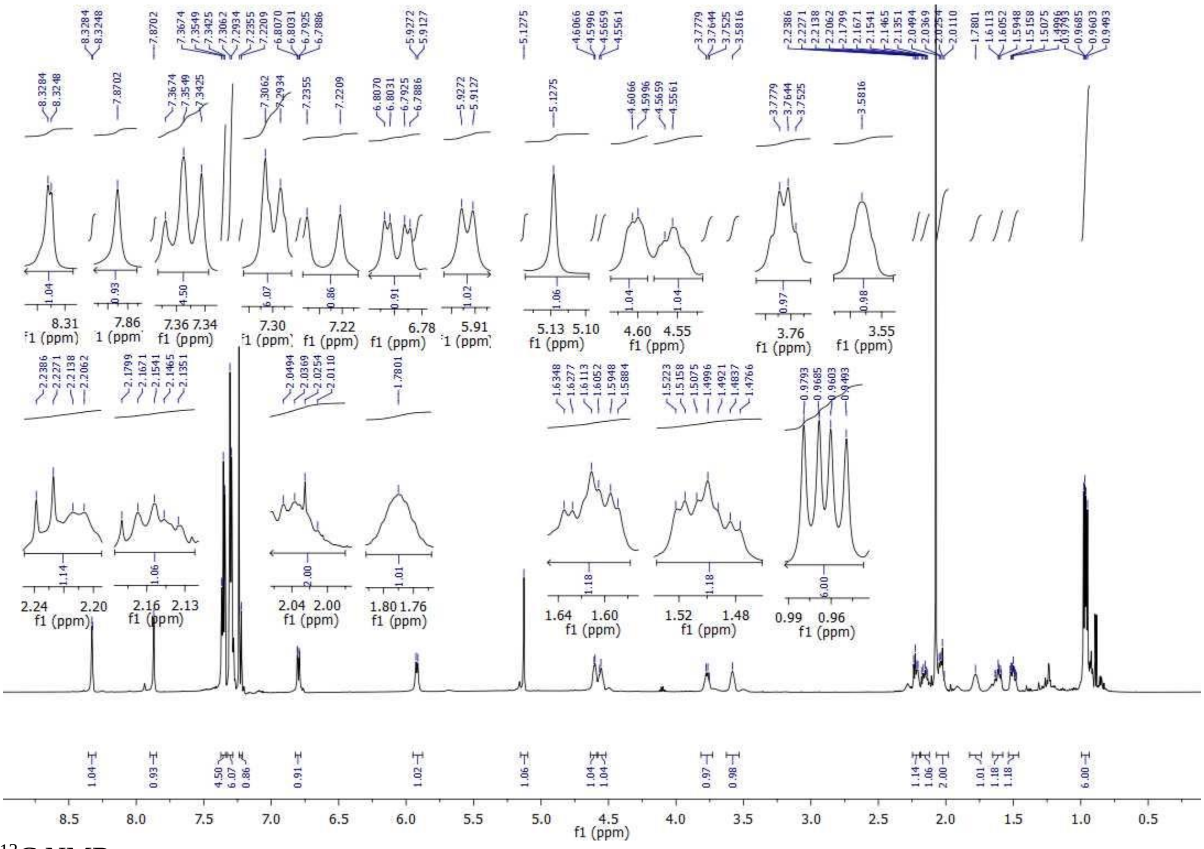

^{13}C NMR

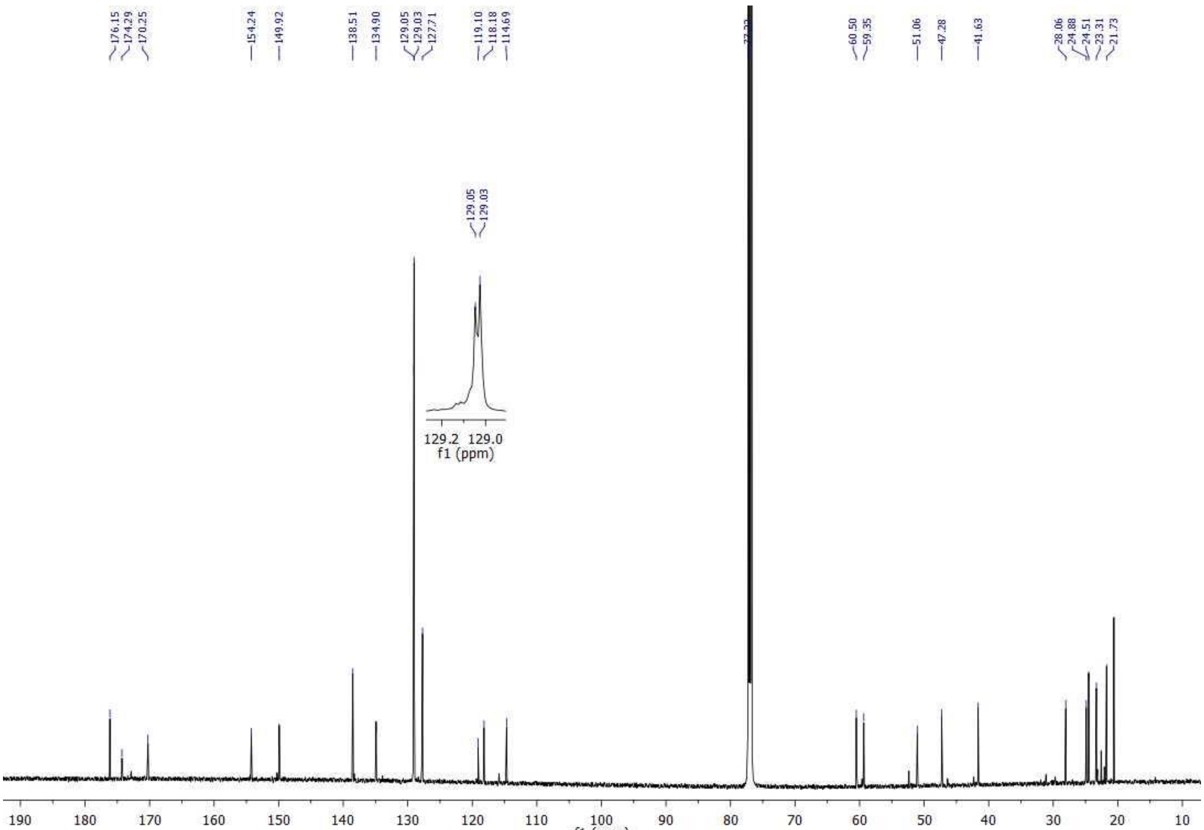

Compound **23**

^{1}H NMR

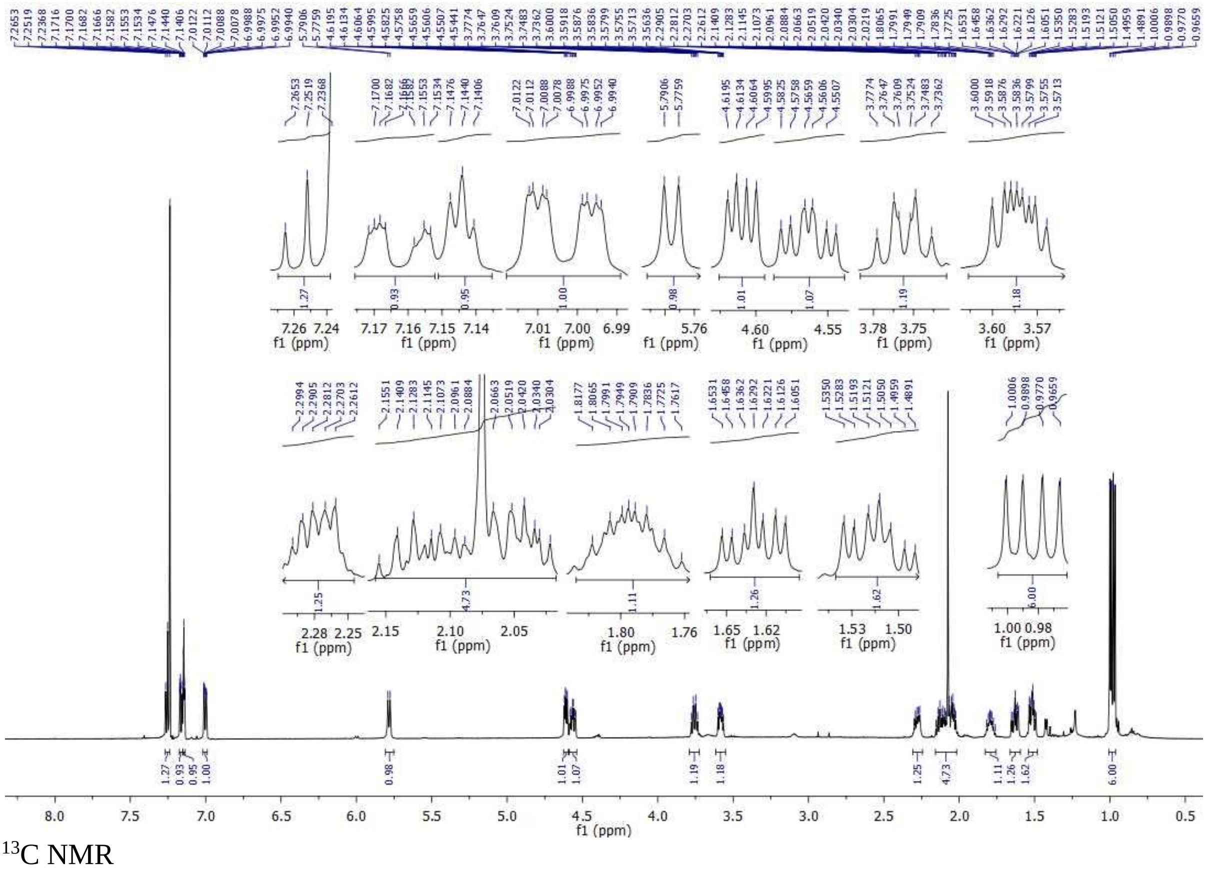

^{13}C NMR

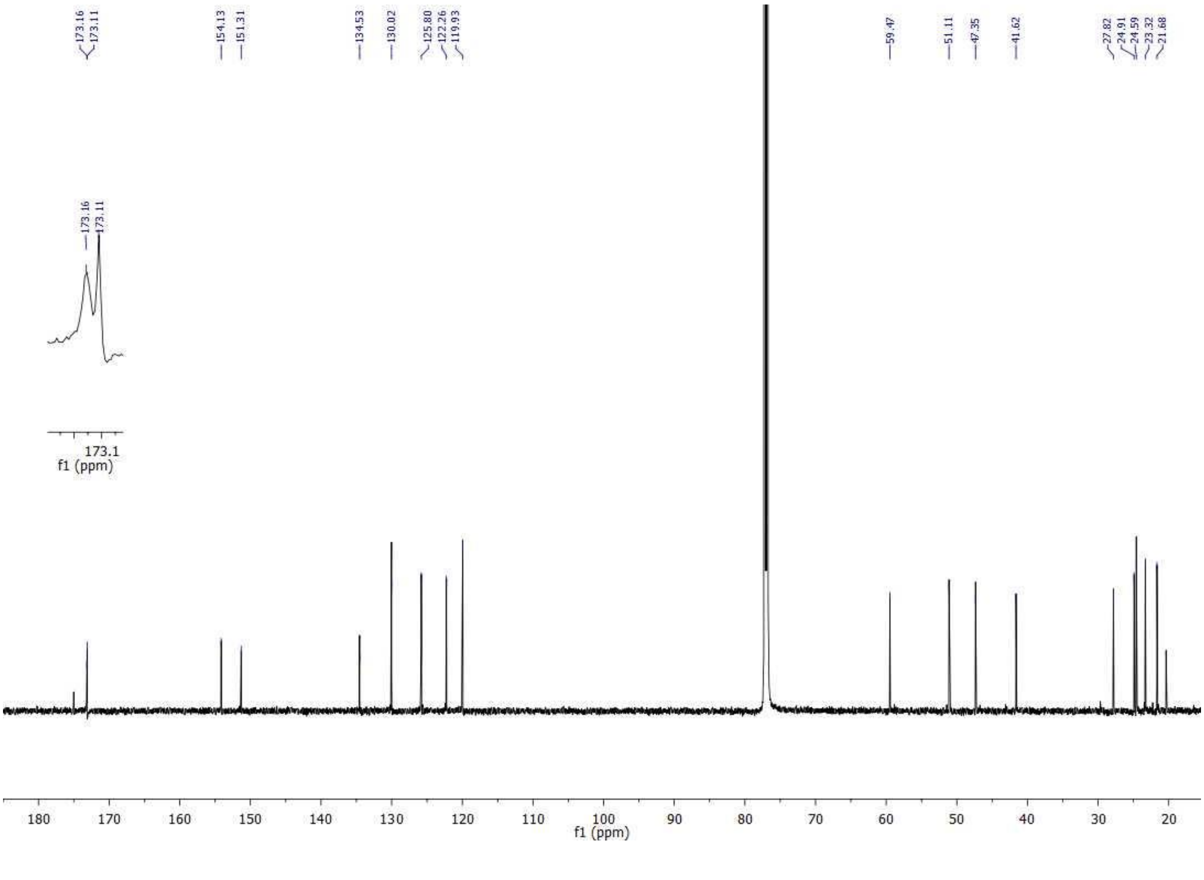

Compound **24**

^{1}H NMR

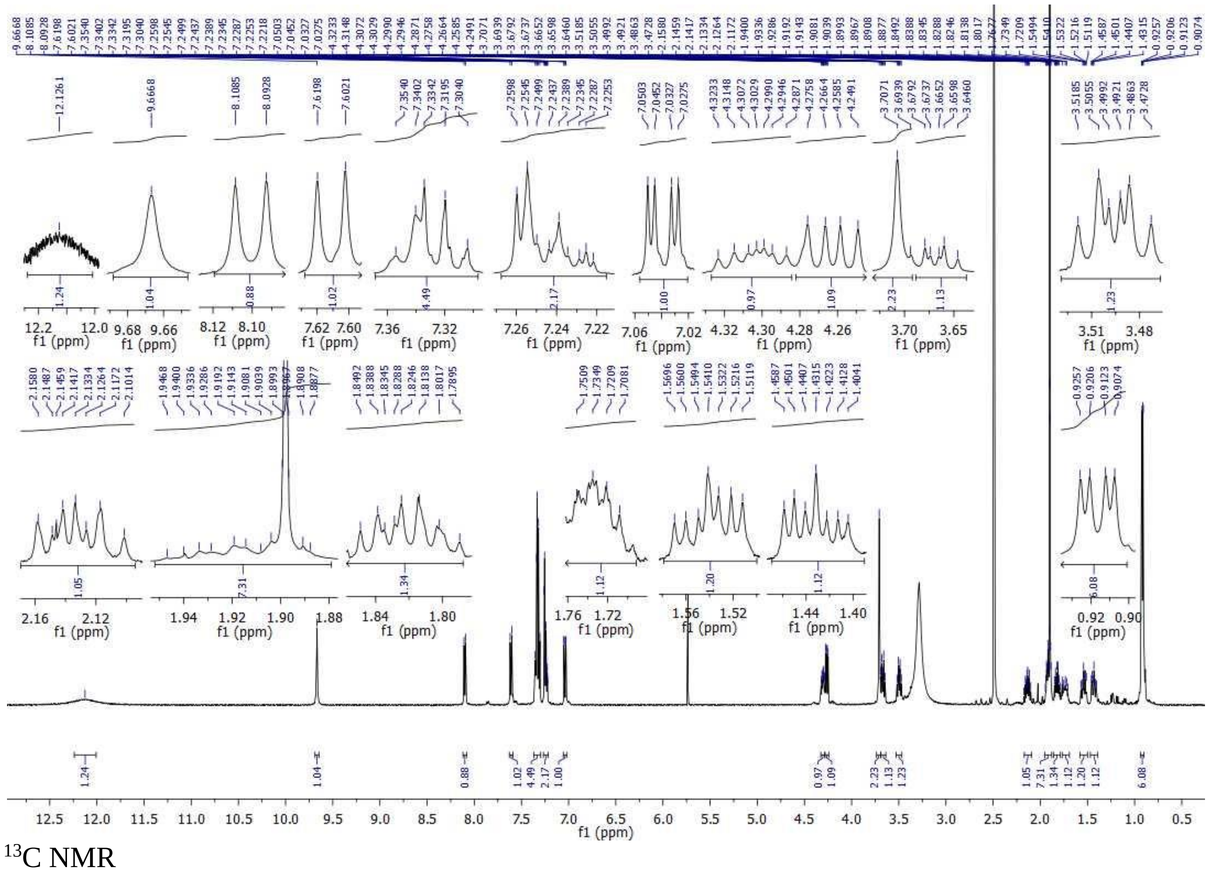

^{13}C NMR

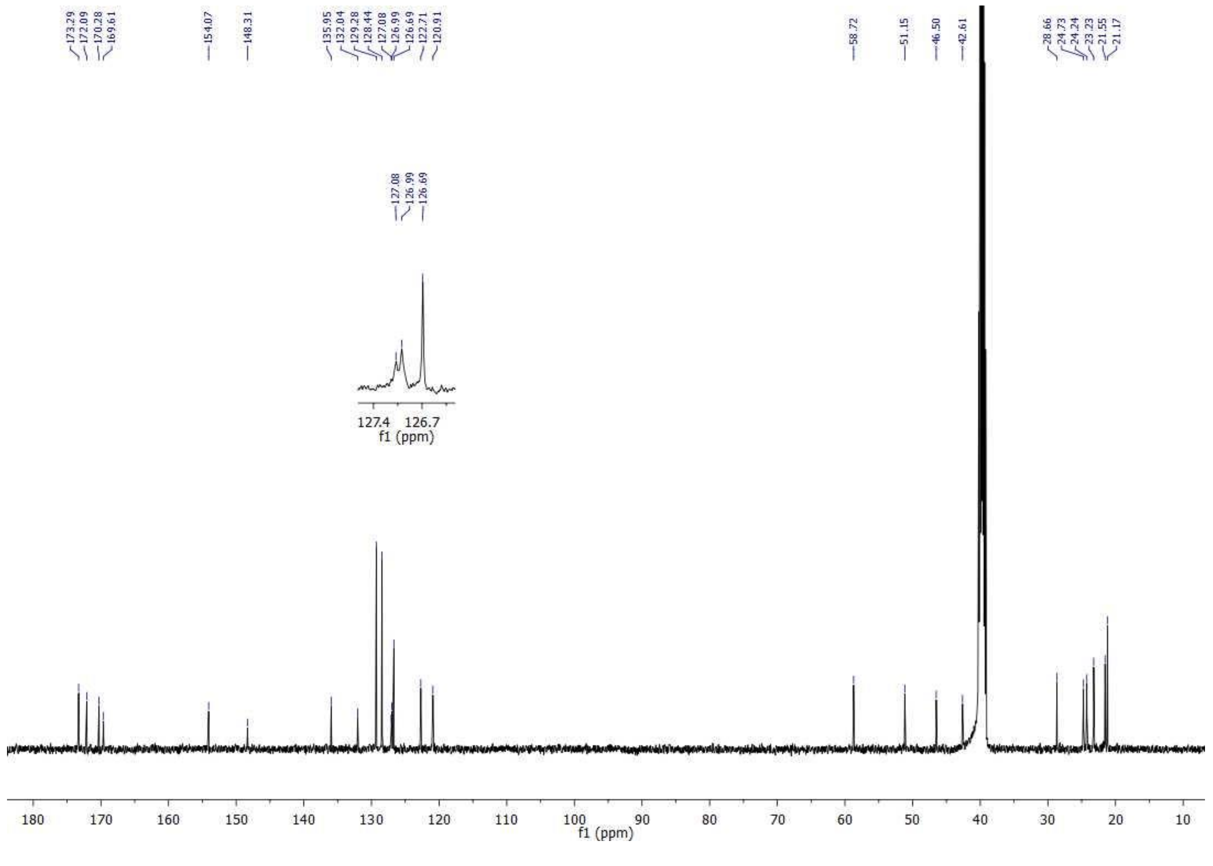

Compound **25**

^{1}H NMR

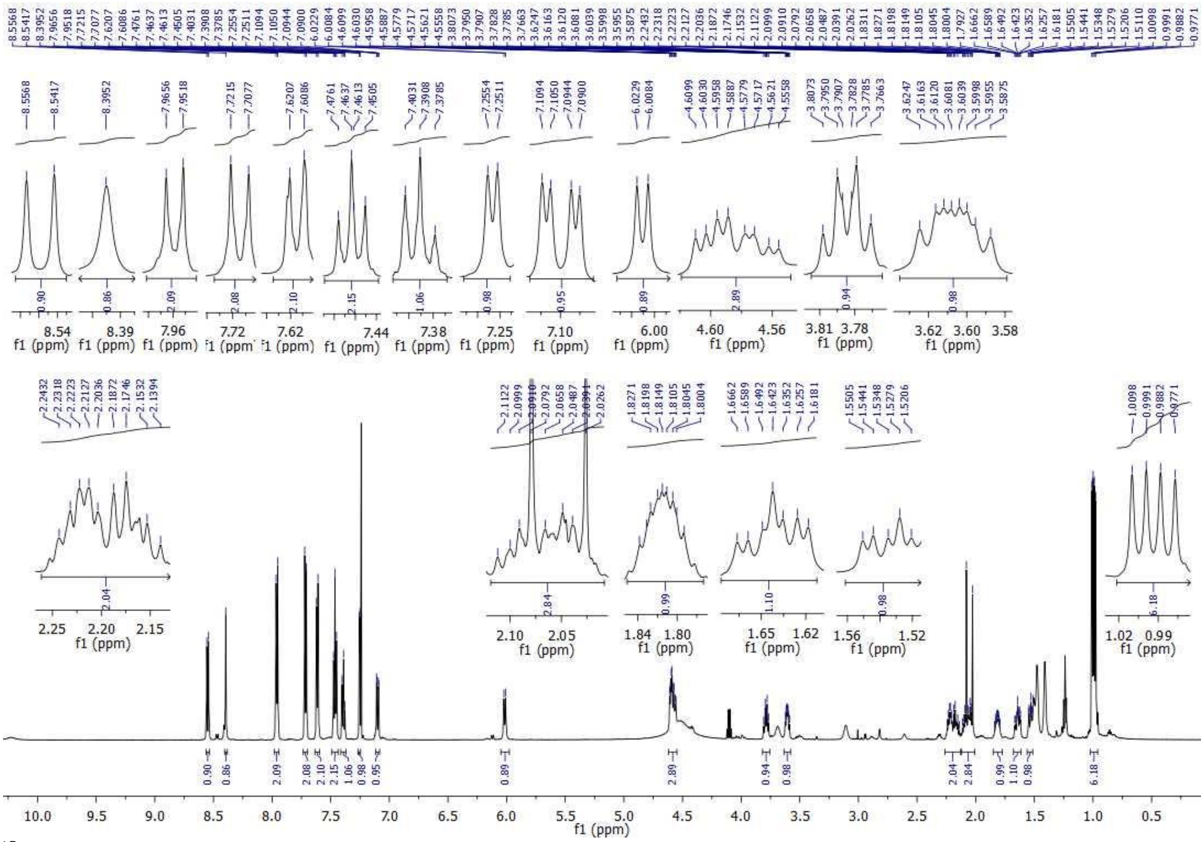

^{13}C NMR

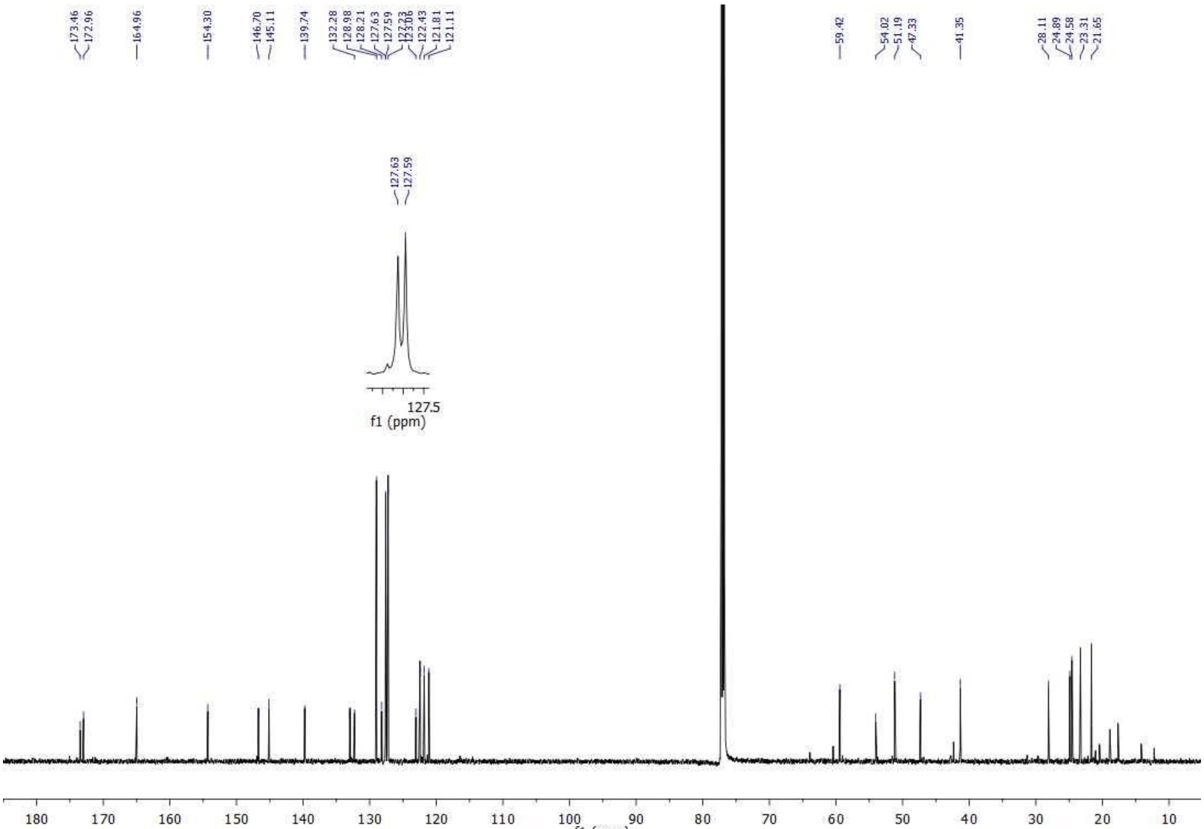

Compound **26**

^{1}H NMR

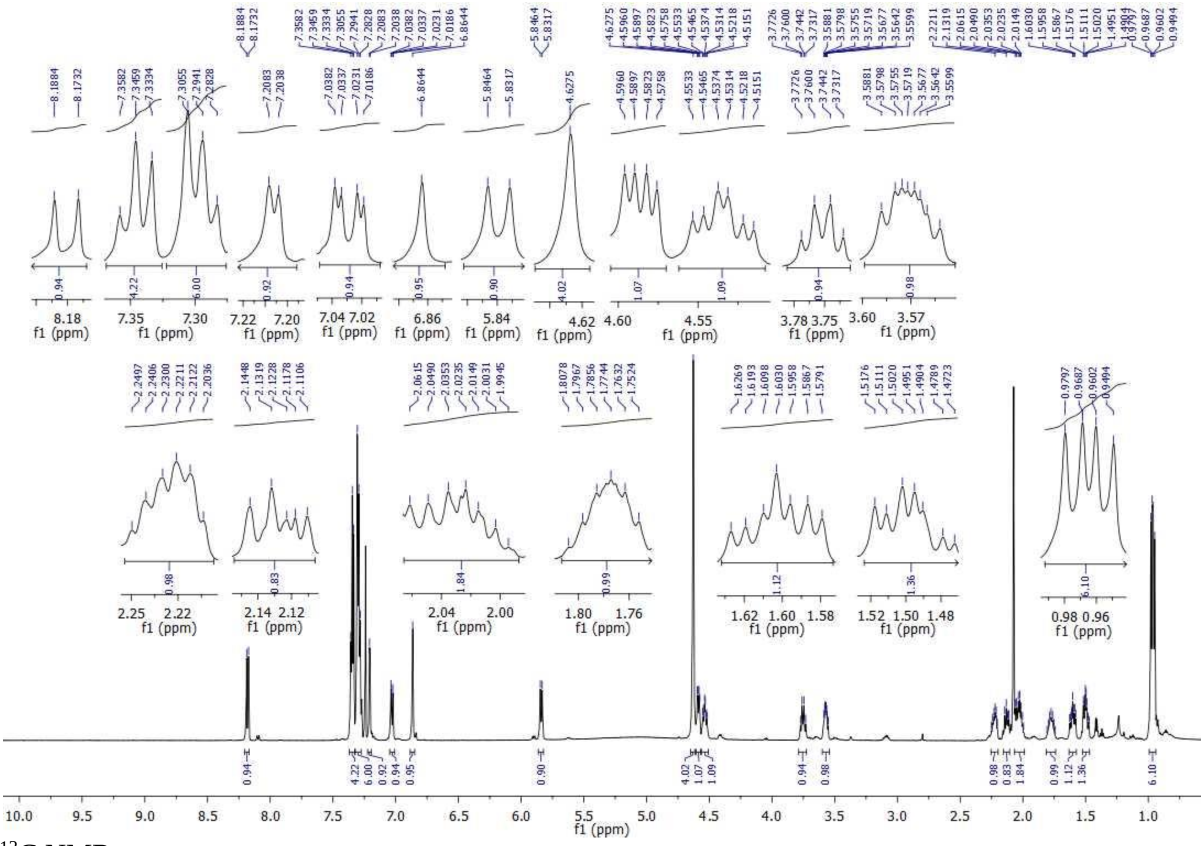

^{13}C NMR

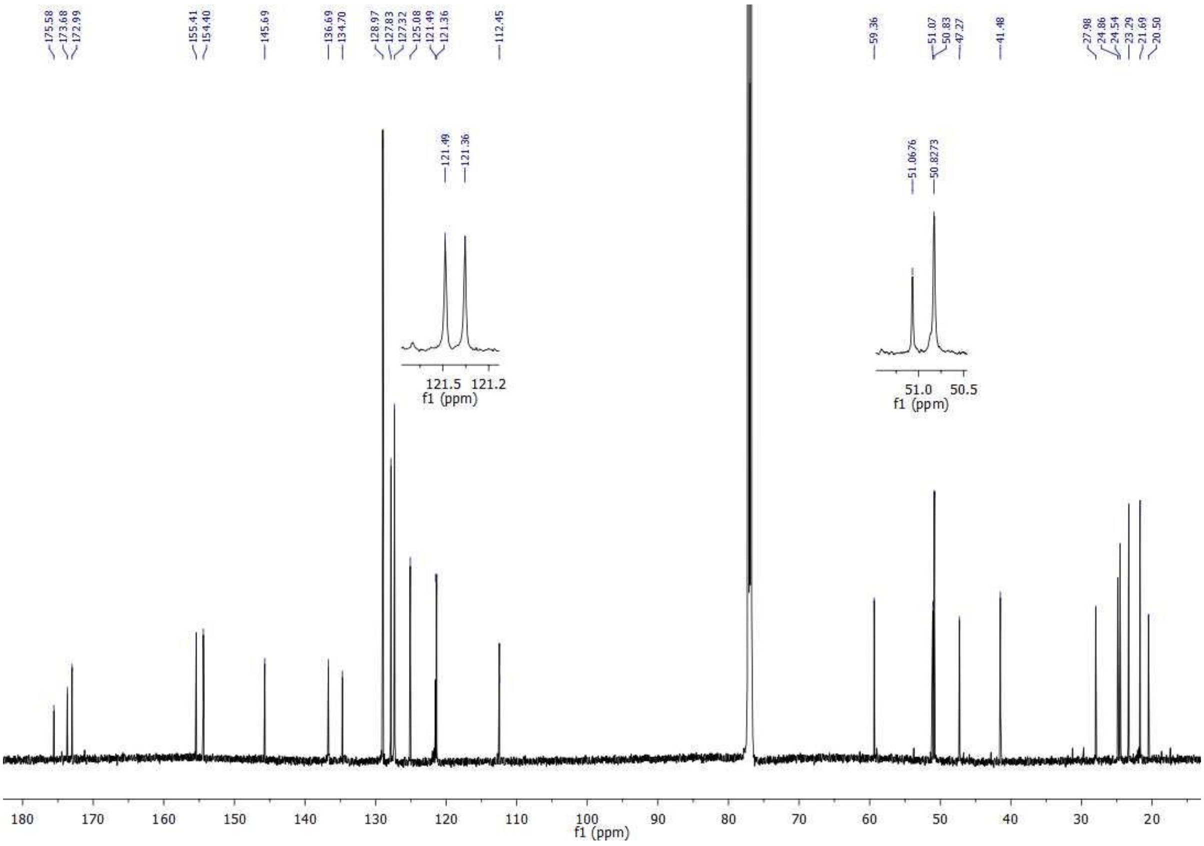

Compound **27**

^{1}H NMR

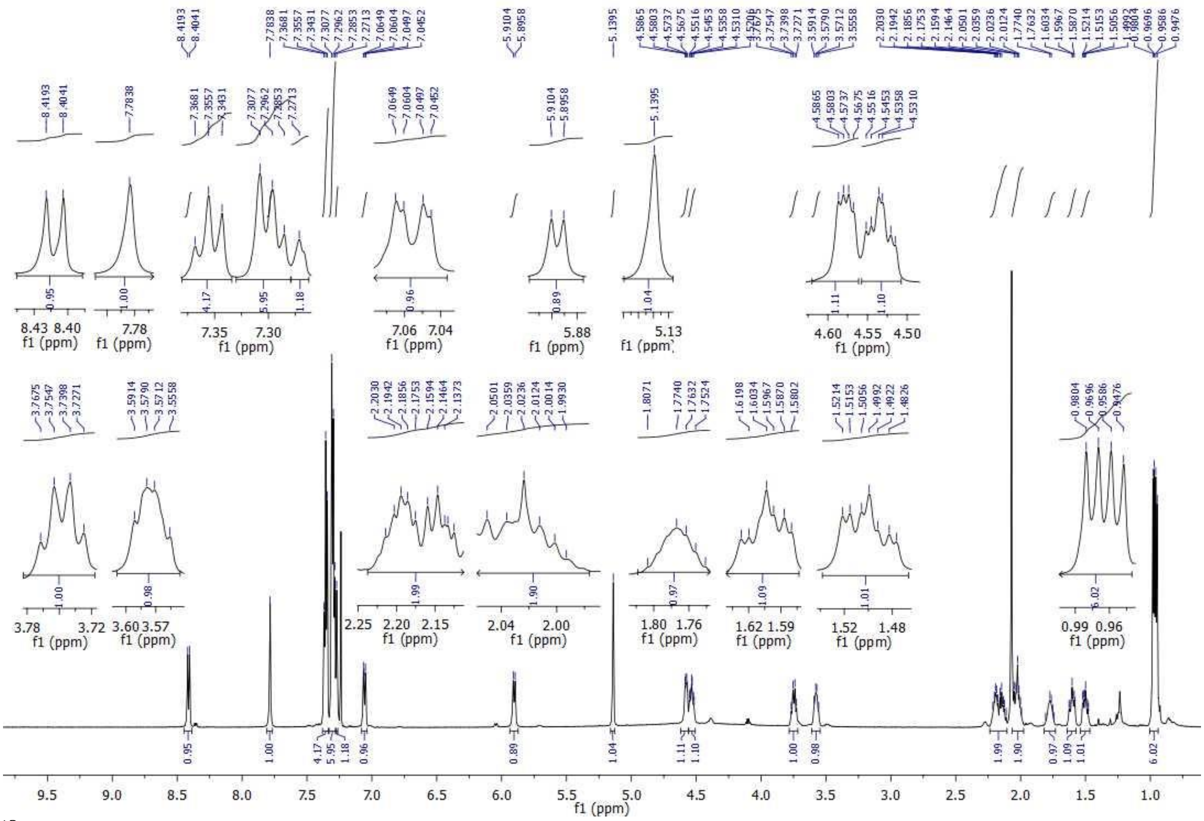

^{13}C NMR

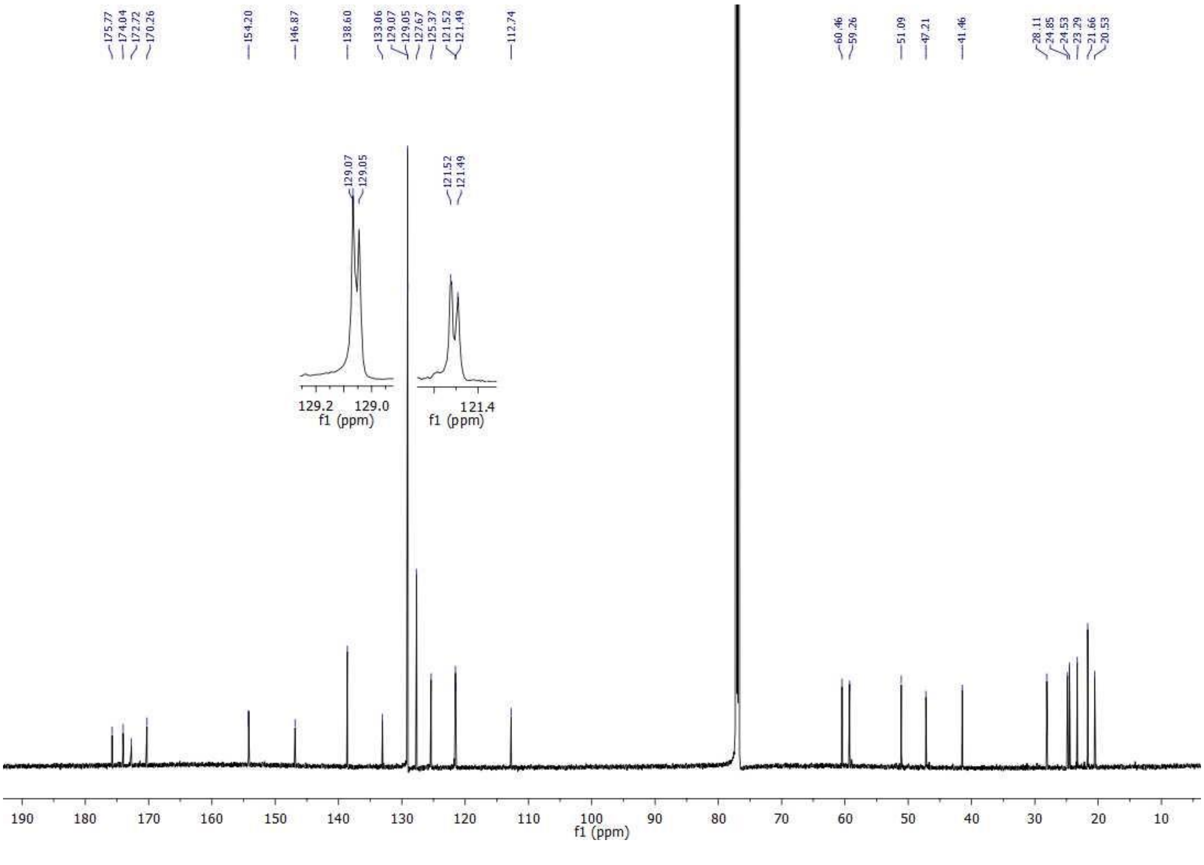

Compound **28**

^{1}H NMR

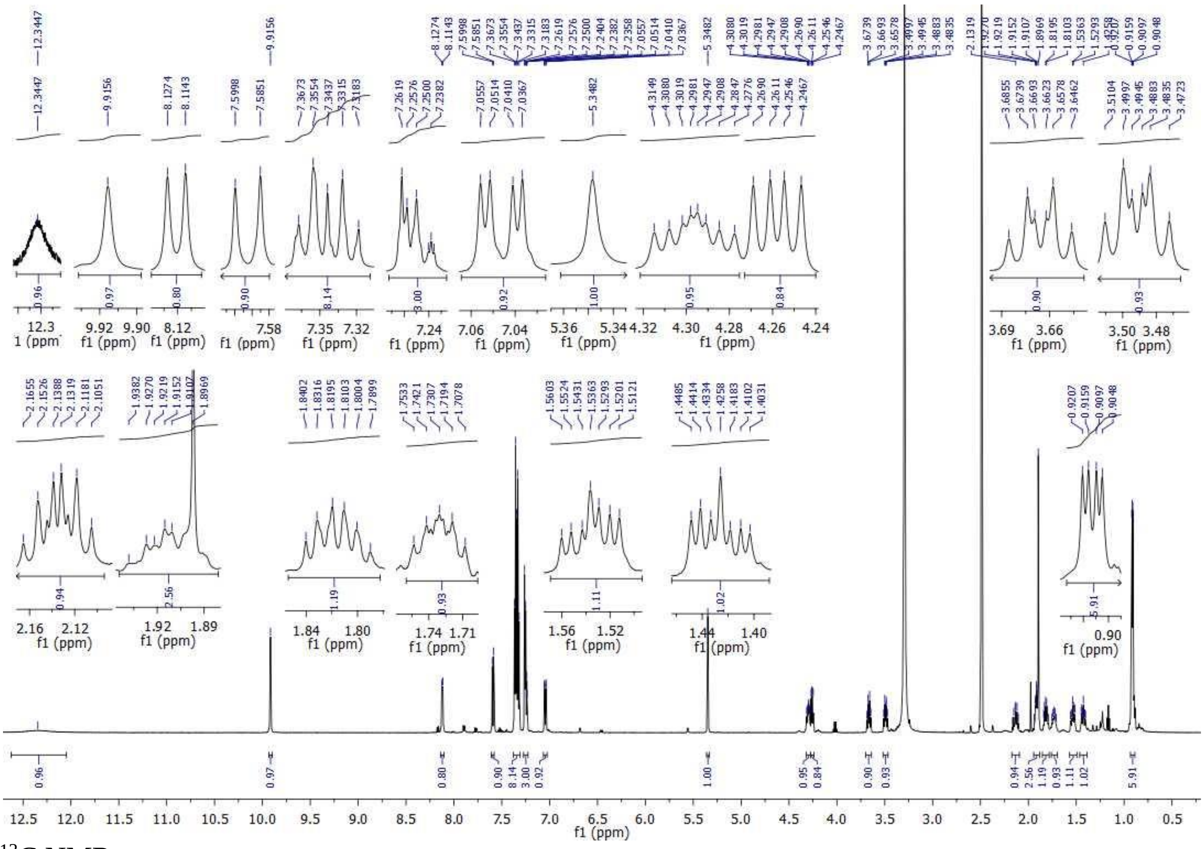

^{13}C NMR

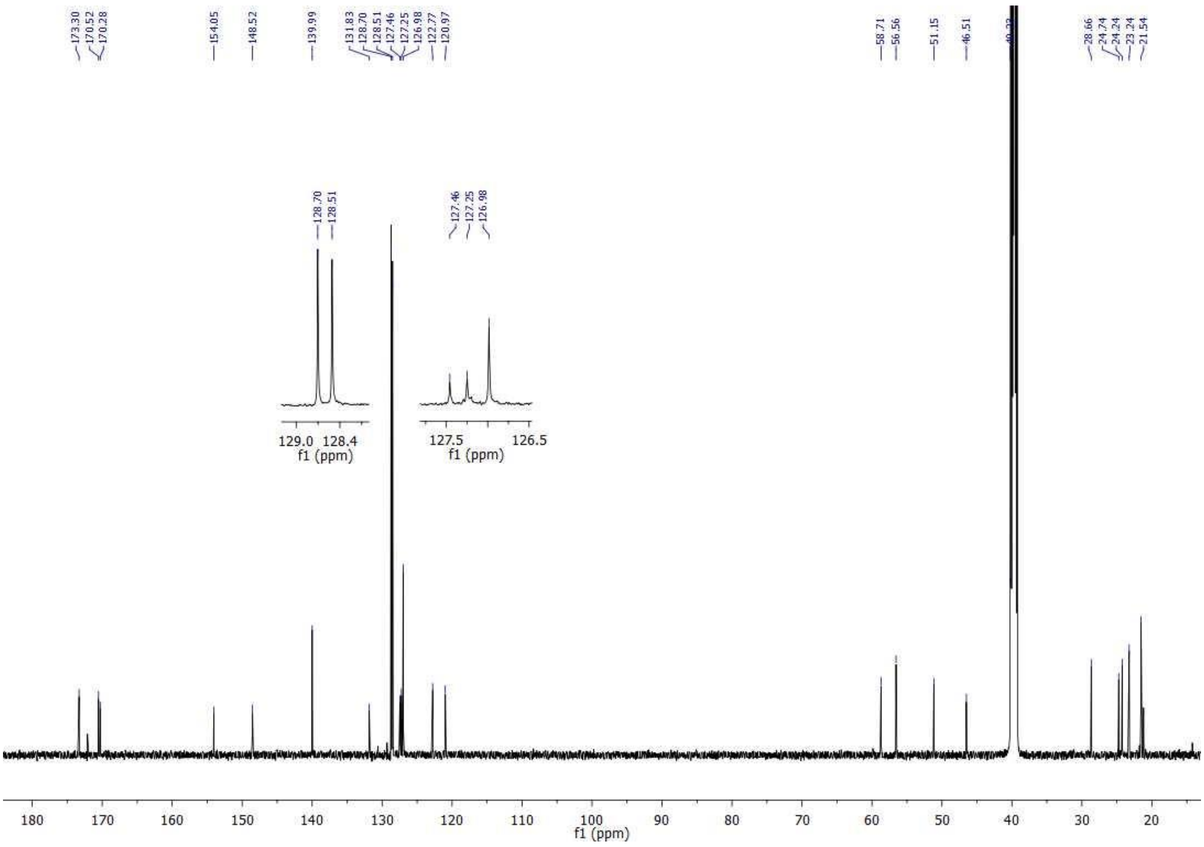

Compound **29**

^{1}H NMR

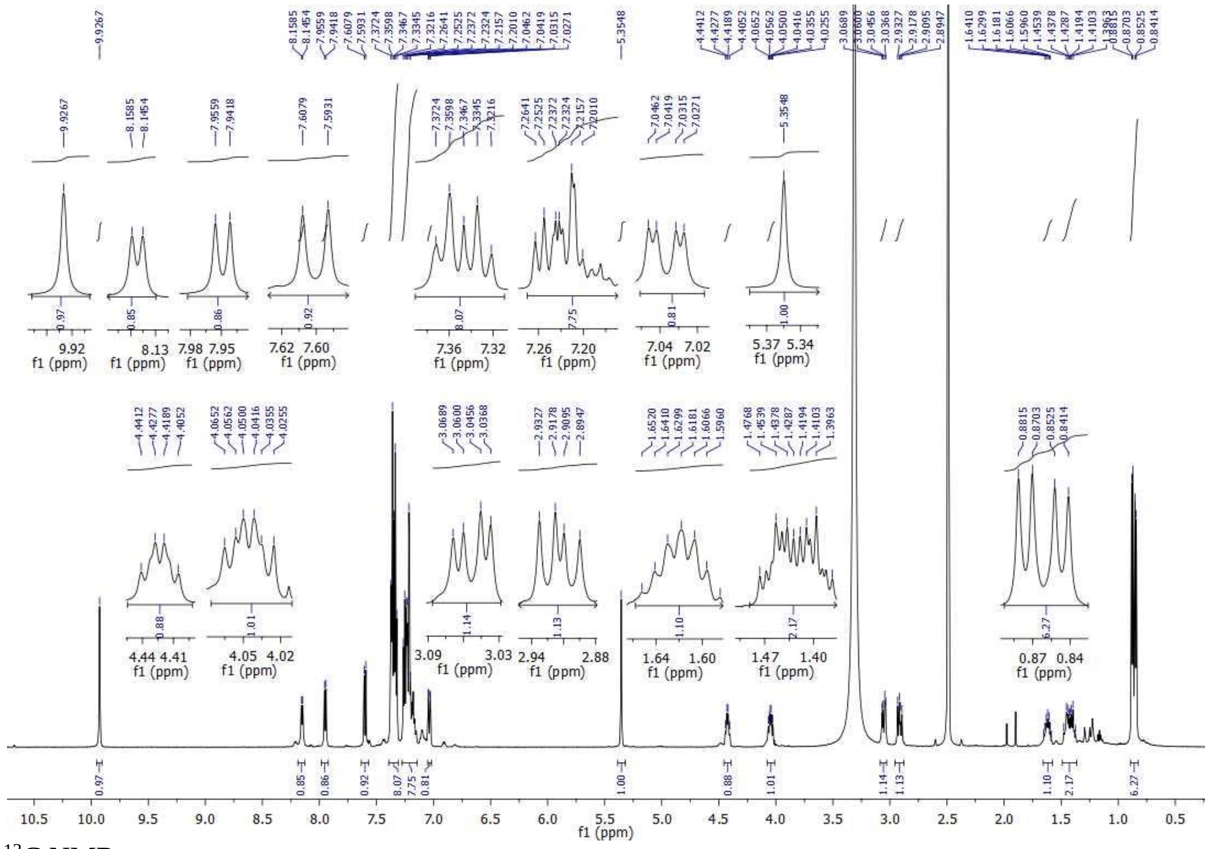

^{13}C NMR

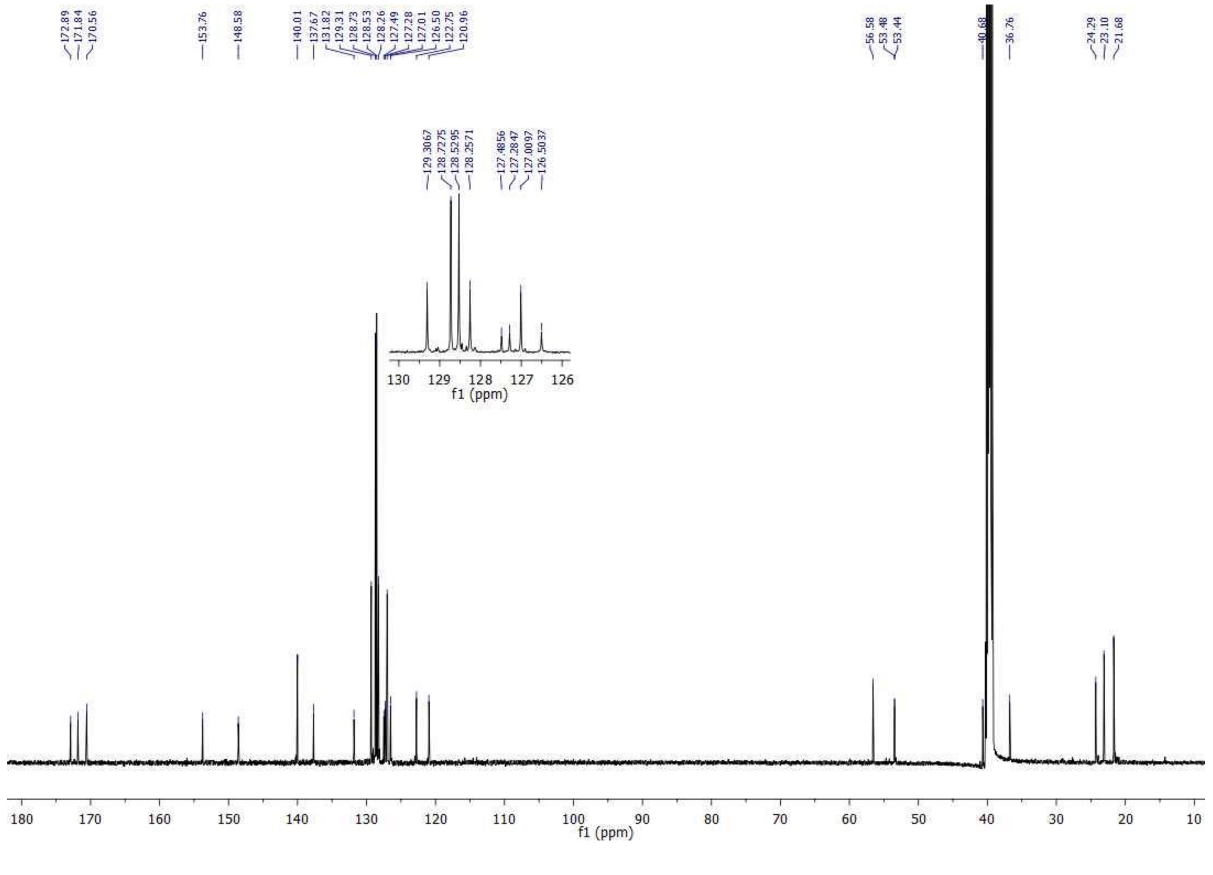

Compound **30**

^{1}H NMR

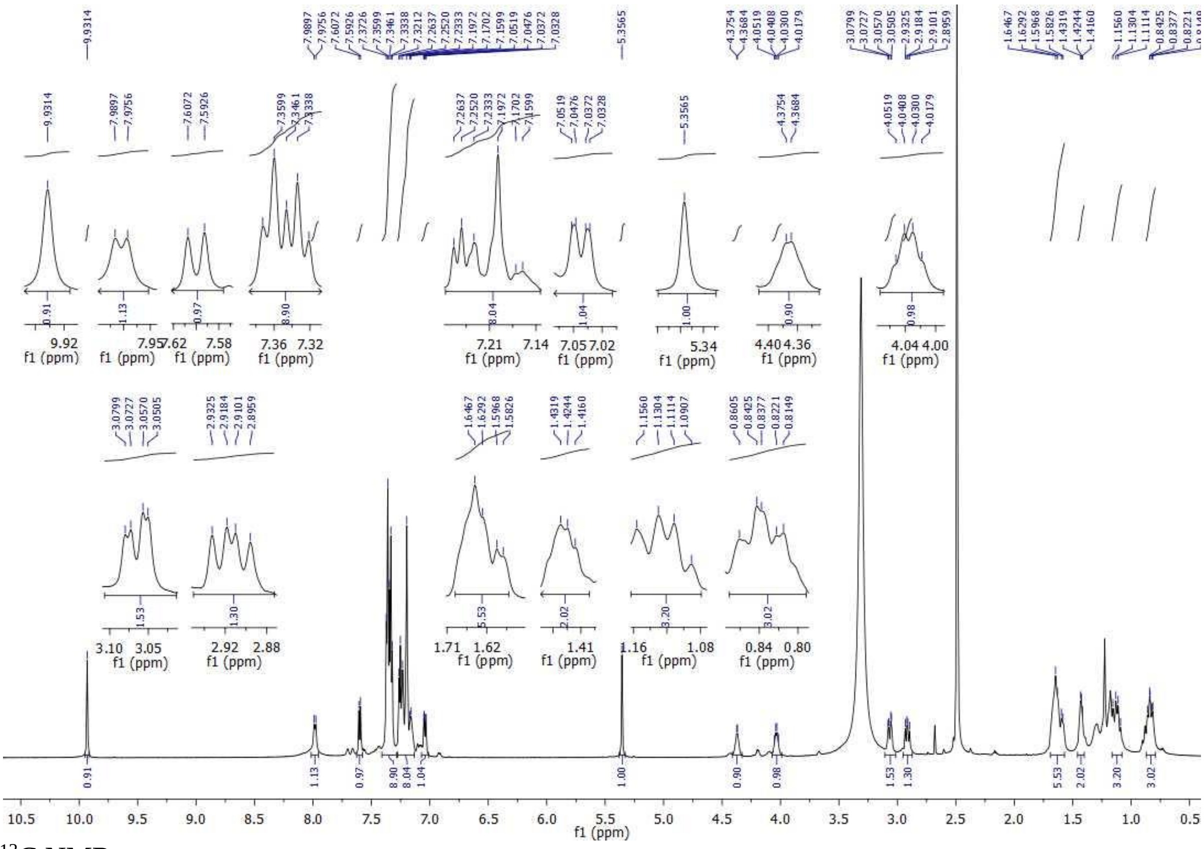

^{13}C NMR

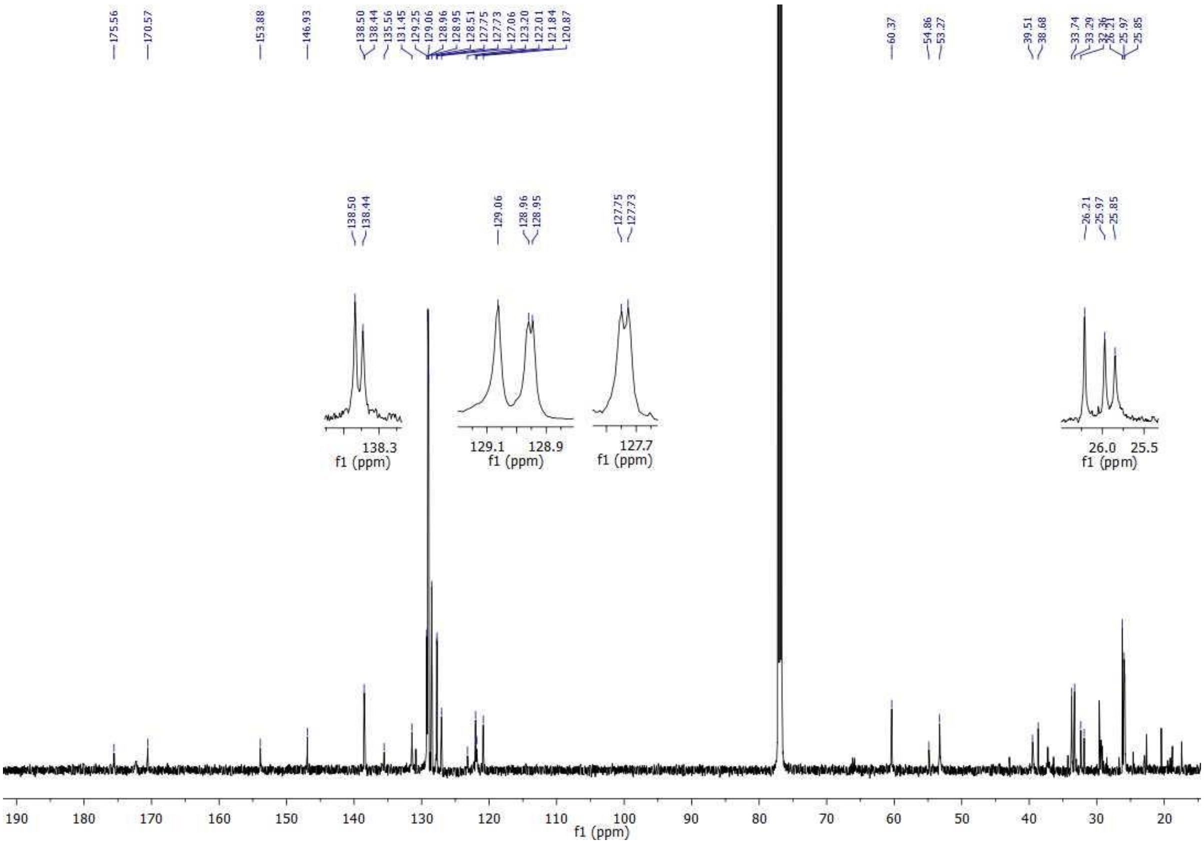

Compound **31**

^{1}H NMR

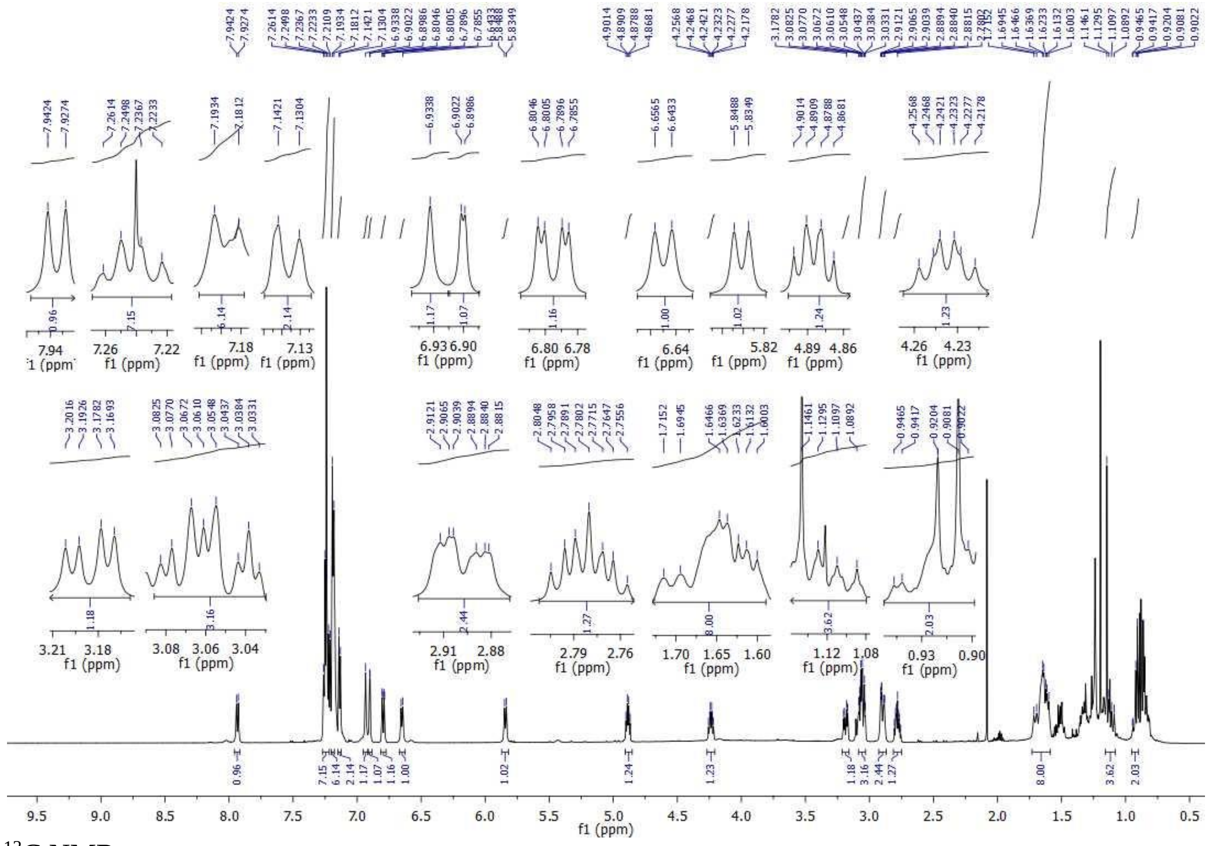

^{13}C NMR

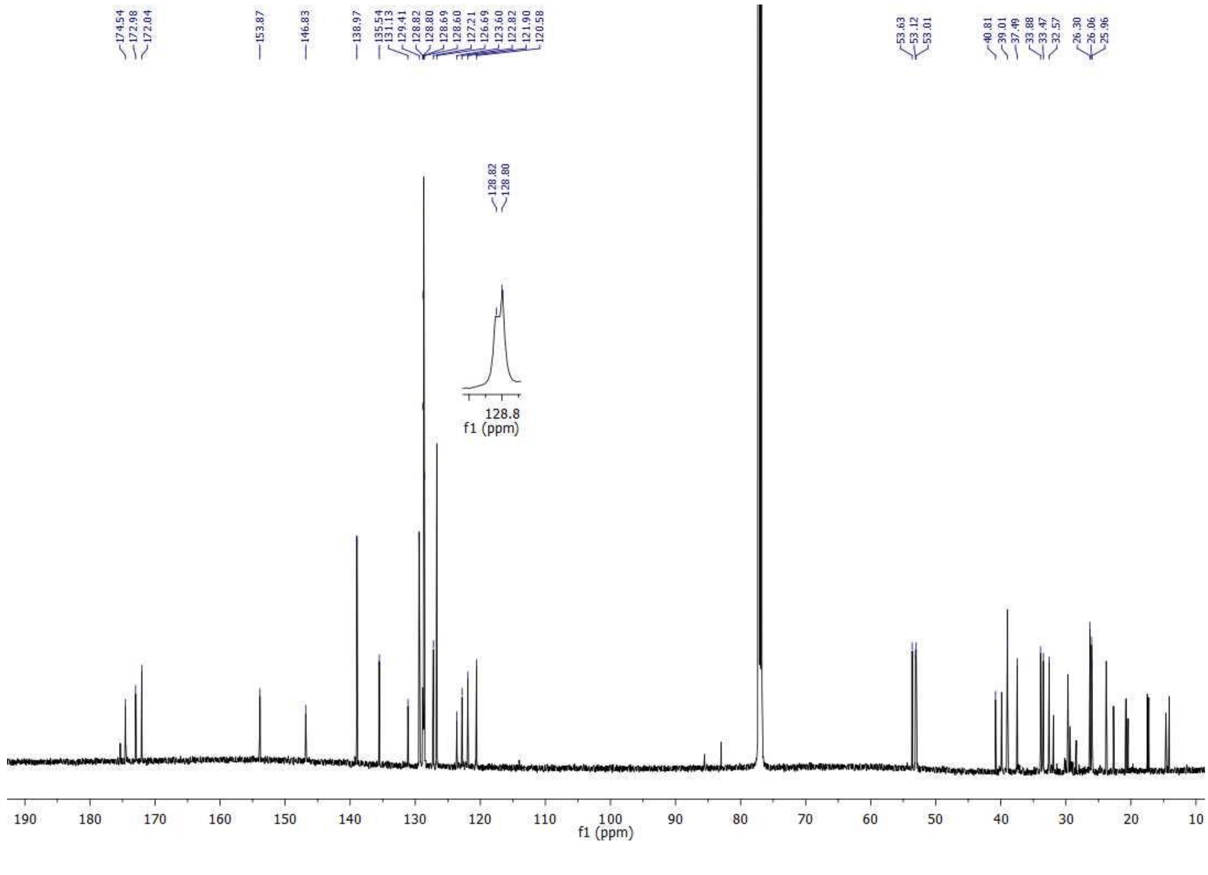

9. References

[1] M. Frizler, F. Lohr, N. Furtmann, J. Kläs, M. Gütschow, *J. Med. Chem.* **2011**, *54*, 396–400.

[2] M. D. Mertens, J. Schmitz, M. Horn, N. Furtmann, J. Bajorath, M. Mareš, M. Gütschow, *ChemBioChem* **2014**, *15*, 955–959.

[3] D. Turk, M. Podobnik, T. Popovic, N. Katunuma, W. Bode, R. Huber, V. Turk, *Biochemistry* **1995**, *34*, 4791−4797.

[4] *RCSB Protein Data Bank.* http://www.rcsb.org (accessed December 5th, 2018).

[5] *Molecular Operating Environment (MOE)*, 2018.01; Chemical Computing Group ULC, 1010 Sherbooke St. West, Suite #910, Montreal, QC, Canada, H3A 2R7, **2018**.

[6] D. Musil, D. Zucic, D. Turk, R. A. Engh, I. Mayr, R. Huber, T. Popovic, V. Turk, T. Towatari, N. Katunuma, W. Bode, *Embo J.* **1991**, *10*, 2321–2330.

[7] D. A. Case, V. Babin, J. T. Berryman, R. M. Betz, Q. Cai, D. S. Cerutti, T. E. Cheatham, T. A. Darden, R. E. Duke, H. Gohlke, A. W. Goetz, S. Gusarov, N. Homeyer, P. Janowski, J. Kaus, I. Kolossvary, A. Kovalenko, T. S. Lee, S. LeGrand, T. Luchko, R. Luo, B. Madej, K. M. Merz, F. Paesani, D. R. Roe, A. Roitberg, C. Sagui, R. Salomon-Ferrer, G. Seabra, C. L. Simmerling, W. Smith, J. Swails, R. C. Walker, J. Wang, R. M. Wolf, X. Wu, P. A. Kollman, AMBER, 14, **2014**.

[8] V. Hornak, R. Abel, A. Okur, B. Strockbine, A. Roitberg, C. Simmerling, *Proteins* **2006**, *65*, 712–725.

[9] J. A. Maier, C. Martinez, K. Kasavajhala, L. Wickstrom, K. E. Hauser, C. Simmerling, *J. Chem. Theory Comput.* **2015**, *11*, 3696−3713.

[10] J. M. Wang, R. M. Wolf, J. W. Caldwell, P. A. Kollman, D. A. Case, *J. Comput. Chem.* **200**5, *26*, 114–114.

[11] J. M. Wang, W. Wang, P. A. Kollman, D. A. Case, *J. Mol. Graph. Model.* **2006**, *25*, 247–260.

[12] W. L. Jorgensen, J. Chandrasekhar, J. D. Madura, R. W. Impey, M. L. Klein, *J. Chem. Phys.* **1983**, *79*, 926–935.

[13] M. von Arnim, R. Ahlrichs, *J. Comput. Chem.* **1999**, *19*, 1746–1757.

[14] A. Klamt, G. Schuurmann, *J. Chem. Soc. Perkin Trans. 2*, **1993**, 799–805.

[15] W. Thiel, MNDO2005 V7.0, Max-Planck-Institut für Kohlenforschung, Mühlheim, Germany, **2005**.

[16] ChemShell, a Computational Chemistry Shell, see www.chemshell.org.

[17] J. Kästner, J. M. Carr, T. W. Keal, W. Thiel, A. Wander, P. Sherwood, *J. Phys. Chem. A* **2009**, *113*, 11856–11865.

[18] Origin, OriginLab, Northampton, MA.

[19] W. Humphrey, A. Dalke, K. Schulten, K. *J. Mol. Graph.* **1996**, *14*, 33–38.

[20] C. Illy, O. Quraishi, J. Wang, E. Purisima, T. Vernet, J. S. Mort, *J. Biol. Chem.* **1997**, *272*, 1197–1202.

[21] J. Benýšek, M. Buša, P. Rubešová, J. Fanfrlík, M. Lepšík, J. Brynda, Z. Matoušková, U. Bartz, M. Horn, M. Gütschow, M. Mareš, *J. Enzyme Inhib. Med. Chem.* **2022**, *37*, 515–526.

[22] A. Jílková, P. Rubešová, J. Fanfrlík, P. Fajtová, P. Řezáčová, J. Brynda, M. Lepšík, H. Mertlíková-Kaiserová, C. D. Emal, A. R. Renslo, W. R. Roush, M. Horn, C. R. Caffrey, M. Mareš, *ACS Infect. Dis.* **2021**, *7*, 1077–1088.

[23] M. Gerlach, U. Mueller, M. Weiss, *JLSRF* **2016**, *2*, A47.

[24] W. Kabsch, *Acta Crystallogr. D Biol. Crystallogr.* **2010**, *66*, 125–132.

[25] A. Vagin, A. Teplyakov, *Acta Crystallogr. D Biol. Crystallogr.* **2000**, *56*, 1622–1624.

[26] M. D. Winn, C. C. Ballard, K. D. Cowtan, E. J. Dodson, P. Emsley, P. R. Evans, R. M. Keegan, E. B. Krissinel, A. G. Leslie, A. McCoy, S. J. McNicholas, G. N. Murshudov, N. S. Pannu, E. A. Potterton, H. R. Powell, R. J. Read, A. Vagin, K. S. Wilson, *Acta Crystallogr. D Biol. Crystallogr.* **2011**, *67*, 235–242.

[27] P. Emsley, K. Cowtan, *Acta Crystallogr. D Biol. Crystallogr.* **2004**, *60*, 2126–2132.

[28] H. Berman, K. Henrick, H. Nakamura, *Nat. Struct. Biol.* **2003**, *10*, 980–980.

[29] S. C. Lovell, I. W. Davis, W. B. Arendall, 3rd, P. I. de Bakker, J. M. Word, M. G. Prisant, J. S. Richardson, D. C. Richardson, *Proteins* **2003**, *50*, 437–450.

[30] S. Salentin, S. Schreiber, V. J. Haupt, M. F. Adasme, M. Schroeder, *Nucleic Acids Res.* **2015**, *43*, W443–447.

[31] P. A. Karplus, K. Diederichs, *Science* **2012**, *336*, 1030–1033.

[32] A. T. Brünger, *Nature* **1992**, *355*, 472–475.

[33] M. Thorsen, T. P. Andersen, U. Pedersen, B. Yde, S. O. Lawesson, H. F. Hansen, *Tetrahedron*, **1985**, *41*, 5633–5636.

[34] R. L. Huguenin, S.Guttmann, *Helv. Chim. Acta*. **1965**, *48*, 1885–1898.

[35] C. Zeggaf, J. Poncet, P. Jouin, M. N. Dufour, B. Castro, *Tetrahedron*. **1989**, *45*, 5039–5050.

[36] C. A. Wartchow, P. Wang, M. D. Bednarski, M. R. Callstrom, *J. Org. Chem.* **1995**, *60*, 2216–2226.

[37] M. J. Yang, J. Wu, Z. D. Yang, Y. M. Zhang, *Chinese Chem. Lett*. **2009**, *20*, 527–530.

[38] D. Blanot, J. Martinez, G. Auger, E. Bricas, *Int. J. Peptide Protein Res*. **1979**, *14*, 41–56.

[39] D. Seebach, S. Abele, K. Gademann, G. Guichard, T. Hintermann, B. Jaun, J. L. Matthews, J. V. Schreiber, *Helv. Chim. Acta* **1998**, *81*, 932–982.

[40] G. Reyes-Rangel, E. Jiménez-González, J. L. Olivares-Romero, E. Juaristi, *Tetrahedron: Asymmetry* **2008**, *19*, 2839–2849.
